# Ring‐Opening Arylation of Cyclic Diaryliodoniums via Cyclic Triaryliodanes as Aryne Precursors and Dynamic Aryl Reservoirs

**DOI:** 10.1002/anie.6935149

**Published:** 2026-05-01

**Authors:** Daichi Ikeshita, Mao Atarashi, Naohiko Yoshikai

**Affiliations:** ^1^ Graduate School of Pharmaceutical Sciences, Tohoku University Sendai Japan

**Keywords:** arynes, Grignard reagents, hypervalent iodine compounds, oligoaryls, reaction mechanism

## Abstract

Cyclic diaryliodonium salts are widely employed as linchpins for constructing functionalized biaryls and as precursors to arynes under basic conditions. However, their synthetically practical engagement with strongly nucleophilic organometallic reagents has remained largely unexplored. Here we disclose a reaction between these salts and aryl Grignard reagents that delivers iodine‐capped teraryls with predominant *meta*‐selectivity. In contrast to ligand coupling at iodine(III) or direct base‐induced aryne formation, the present transformation is initiated by the formation of a cyclic triaryliodane, which serves as both an aryne precursor and a dynamic aryl reservoir within a self‐propagating carbomagnesiation sequence. Spectroscopic analyses and control experiments support the involvement of the triaryliodane and reversible aryl exchange with aryl Grignard reagents, while trapping experiments intercept both aryne and terarylmagnesium intermediates. The reaction tolerates sterically and electronically diverse aryl Grignard reagents with consistent *meta*‐selectivity, whereas strongly perturbing substituents on the iodonium salts can modulate the regioselectivity. The resulting iodo‐teraryls retain a versatile C─I bond amenable to further π‐extension or recyclization into cyclic iodonium architectures.

## Graphical abstract

Cyclic diaryliodonium salts engage aryl Grignard reagents to trigger a self‐propagating aryne–arylation cycle. Transient cyclic triaryliodanes serve as aryne precursors and dynamic aryl reservoirs, delivering iodine‐capped teraryls with predominant *meta*‐selectivity. The preserved C─I bond enables outward π‐extension or inward recyclization.

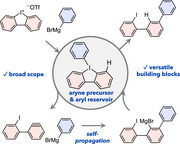

## 1 Introduction

**SCHEME 1:**
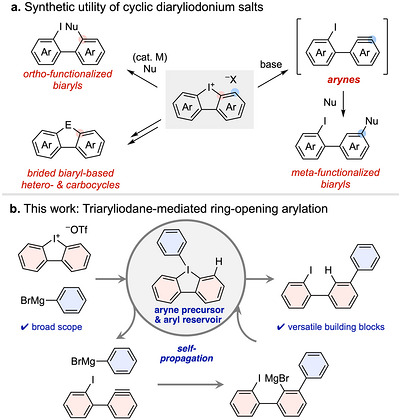
Reactivity of cyclic diaryliodonium salts.

Cyclic diaryliodonium salts, particularly the five‐membered variants, uniquely combine the electrophilic character of iodine(III) centers [1, 2] with intrinsic ring strain [3, 4, 5]. Together with their ready accessibility from the corresponding 2‐iodobiaryls using established synthetic protocols [6, 7, 8, 9, 10], this combination of electrophilicity and strain has rendered them powerful linchpins in synthetic chemistry. Ring‐opening substitutions with heteroatom nucleophiles deliver versatile 2‐iodo‐2′‐functionalized biaryl scaffolds, often with control over axial chirality where applicable (Scheme 1a, left) [8, 11, 12, 13, 14, 15]. Alternatively, both C─I bonds of cyclic diaryliodoniums can participate in annulative displacement, affording 2,2′‐bridged biaryl‐based hetero‐ and carbocycles. In parallel, cyclic diarylhalonium salts (halogen = Br or Cl) have emerged as competent aryne precursors, enabling *meta*‐functionalization through nucleophilic trapping [16, 17, 18, 19]. In this regard, cyclic diaryliodonium salts have been far less explored: although a few recent studies demonstrated their capacity to generate arynes at elevated temperature (Scheme 1a, right) [20, 21, 22], the overwhelming emphasis in the field has remained on *ortho*‐directed transformations. These two reactivity modes nonetheless position cyclic diaryliodonium salts as privileged platforms for the direct construction of valuable biaryl frameworks, wherein the retained C─I bond provides a versatile handle for further derivatization. Despite these advances, organometallic nucleophiles—particularly arylmetal reagents—have remained largely unexplored in this context. Realizing such ring‐opening arylation while preserving the C─I bond would provide direct access to iodoarylene motifs, key building blocks for oligoarylenes and polycyclic aromatic hydrocarbons (PAHs) [23, 24].

Here, we address this long‐standing gap by realizing the ring‐opening arylation of cyclic diaryliodonium salts with aryl Grignard reagents as carbon nucleophiles (Scheme 1b). Rather than proceeding through the conventional ligand coupling pathway to *ortho*‐teraryls, the reaction delivers iodoteraryl frameworks with predominant *meta* connectivity in high yields across a broad substrate range. Mechanistically, the transformation does not arise from direct base‐induced aryne formation. Instead, it is initiated by the formation of a cyclic triaryliodane, which undergoes deprotonative generation of aryne and release of aryl Grignard as part of a self‐propagating carbomagnesiation sequence. In this cascade, the Grignard reagent serves dual roles—as initiator and aryne trap—while regeneration of an arylmagnesium species sustains the reaction. This mechanistic design contrasts sharply with previously reported Pd‐catalyzed coupling with arylboronic acids, which furnishes *ortho*‐tetraaryls through sequential arylation of the two aryl–iodine bonds [25], thereby establishing a distinct mechanistic mode within hypervalent iodine chemistry.

**SCHEME 2:**
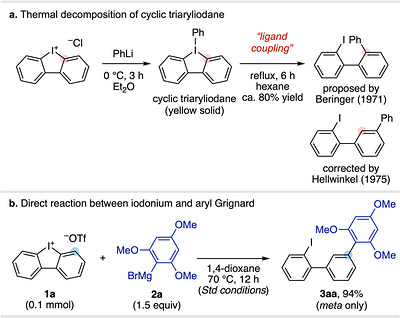
Reaction of cyclic diaryliodoniums with arylmetal reagents.

Over half a century ago, Beringer and Chang investigated the chemistry of cyclic triaryliodane, generated from a cyclic diaryliodonium salt and phenyllithium [26, 27]. At room temperature, this compound underwent gradual decomposition to give a complex mixture containing benzene, iodobenzene, biphenyl, 2‐iodobiphenyl, and 2,2′‐diiodobiphenyl. Upon heating, however, the product distribution shifted dramatically: thermolysis in refluxing hexane afforded a single high‐boiling product in approximately 80% yield, which was assigned—on the basis of mass spectrometry, elemental analysis, and melting point data—as an iodinated *ortho*‐terphenyl derivative (Scheme 2a). The product was described as an oil that gradually solidified (mp 53°C–54°C), consistent with the previously reported melting point [28]. Notably, a subsequent study by Hellwinkel and coworkers in 1975 reassigned the major thermolysis product to the *meta* isomer on the basis of further chemical transformations, thereby correcting the original structural assignment [29]. Around the same period, Reich and Cooperman rigorously examined the structural identity and dynamic stereochemistry of cyclic triaryliodane species through detailed NMR analyses, consolidating their structural framework within main‐group chemistry [30]. Although these studies clarified the structural identity and thermolytic behavior of cyclic triaryliodanes, their reactivity was largely viewed as unimolecular ligand rearrangement, and no connection to aryne‐type processes or synthetically practical intermolecular transformations was established. We envisioned that their in situ generation and controlled reactivity might provide direct access to iodine‐capped teraryl architectures under operationally simple conditions.

## 2 Results and Discussion

Building on these precedents, we examined the reactivity of cyclic diaryliodonium salts toward arylmetal reagents. Whereas earlier studies had employed phenyllithium, we selected aryl Grignard reagents as more practical and broadly accessible nucleophiles, reasoning that they would generate triaryliodane intermediates in situ. Treatment of dibenzo[*b*,*d*]iodol‐5‐ium triflate (**1a**) with 1,3,5‐trimethoxyphenylmagnesium bromide (**2a**, 1.5 equiv) in 1,4‐dioxane at 70°C furnished the *meta*‐arylated product **3aa** in 94% yield as a single regioisomer (Scheme 2b; see Tables S1 and S2 for additional data). The reaction was sluggish at 25°C and barely proceeded at 0°C. No trace of the corresponding *ortho*‐teraryl was observed by ^1^H NMR analysis. Notably, upon addition of the aryl Grignard reagent to a suspension of **1a** in 1,4‐dioxane, the mixture instantaneously turned bright yellow, even at low temperature (−78°C to 0°C), suggesting rapid formation of an iodine(III)‐containing intermediate, consistent with the characteristic coloration reported for triaryliodane species [26, 27]. Under the optimized thermal conditions, this coloration gradually faded as the reaction progressed (Figure S2).

**SCHEME 3:**
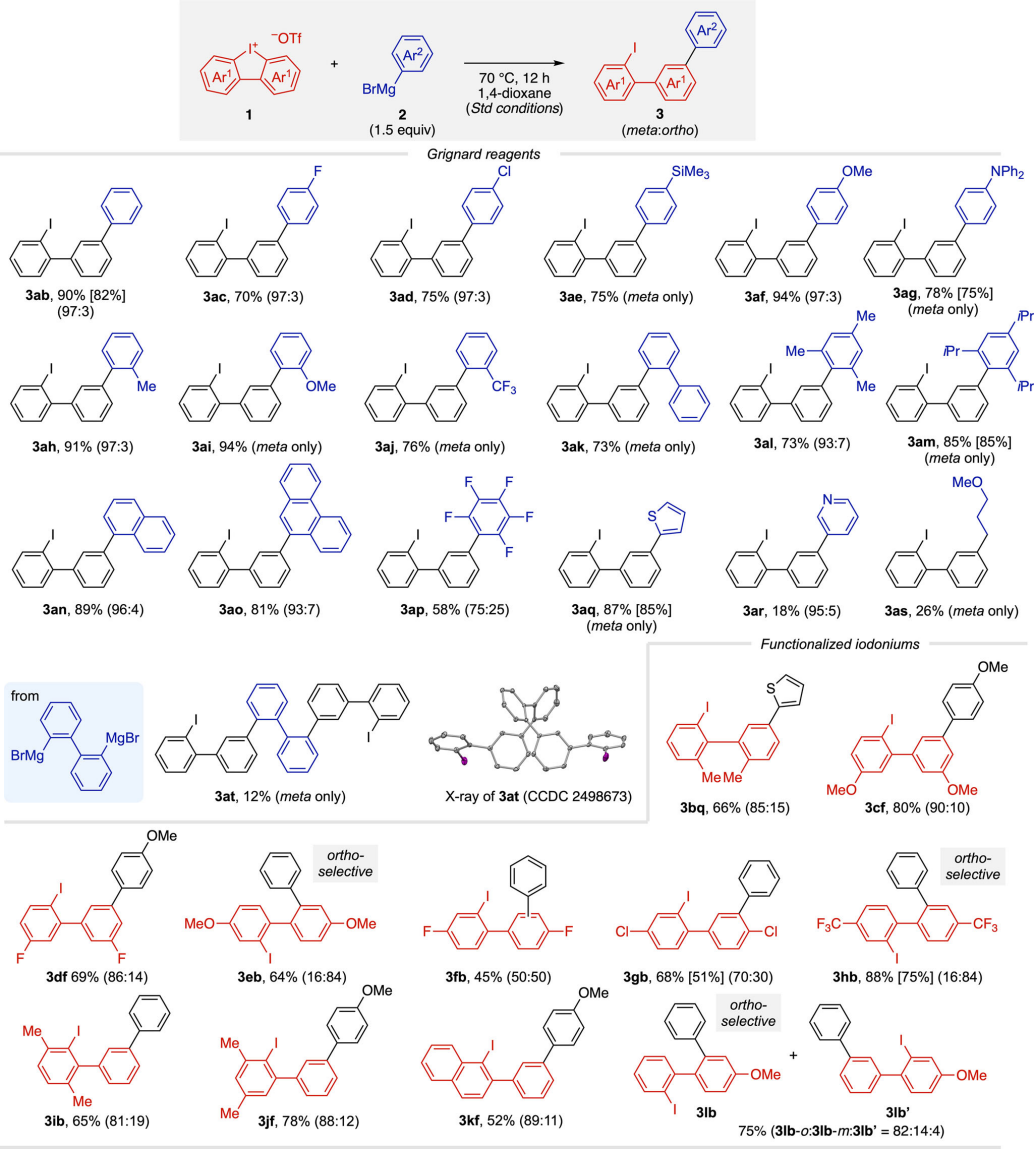
Scope of ring‐opening arylation of diaryliodonium salts with aryl Grignard reagents. The reaction was performed on a 0.1 mmol scale except for **3as** (0.2 mmol scale). Yields refer to the combined isolated yields of *meta*‐ and *ortho* isomers. In the brackets are shown the yields of 1 mmol‐scale reactions. See the Supporting Information for further technical details.

Next, we explored the substrate scope under the optimized conditions (Scheme 3). The reaction accommodated a wide range of aryl Grignard reagents with diverse electronic and steric features. Parent phenylmagnesium bromide and *para*‐substituted reagents bearing halogen (F, Cl), silyl, methoxy, or diphenylamino groups furnished the corresponding *meta*‐teraryl derivatives **3ab**–**3ag** in moderate to good yields with high regioselectivity (≥ 97:3). Sterically demanding nucleophiles, including *ortho*‐substituted aryl Grignard reagents (**3ah**–**3ak**), highly hindered mesityl and 2,4,6‐triisopropylphenyl derivatives (**3al** and **3am**), and polyaromatic species such as 1‐naphthyl and 9‐phenanthryl reagents (**3an** and **3ao**), were likewise tolerated, affording the desired products in good yields and with exclusive or high *meta*‐selectivity. The reaction also accommodated a strongly electron‐deficient pentafluorophenyl Grignard reagent, albeit with a somewhat diminished level of regioselectivity (**3ap**; 75:25). Heteroaryl nucleophiles, including 2‐thienyl and 3‐pyridyl Grignard reagents, underwent *meta*‐selective ring‐opening arylation, though the latter furnished the product **3ar** in low yield. An alkylmagnesium bromide bearing a methoxy substituent also participated, affording the corresponding product **3as** in modest yield. 2,2′‐Dimagnesiobiphenyl enabled bidirectional ring‐opening, affording hexaphenylene derivative **3at** featuring a *meta*, *ortho*, *ortho*, *meta* connectivity pattern. The structure of **3at** was confirmed by single‐crystal X‐ray diffraction, revealing the *C*_2_‐symmetric nature of the molecule [31].

The scope was next examined with respect to substituted cyclic iodonium salts. The 2,2′‐dimethyl‐substituted derivative **1b** reacted with 2‐thienyl Grignard reagent to afford the corresponding teraryl **3bq** with slightly diminished *meta*‐selectivity. Substituents at the 3,3′‐positions were likewise compatible, maintaining the hallmark *meta*‐selectivity in arylative ring‐opening (**3cf** and **3df**). In contrast, the 4,4′‐substituted derivatives exhibited variable regioselectivity, reflecting the sensitivity of the aryne carbomagnesiation step to electronic and geometric perturbations. Thus, the dimethoxy and bis(trifluoromethyl) derivatives preferentially afforded the *ortho*‐isomers (**3eb** and **3hb**), whereas the difluoro and dichloro analogues displayed attenuated or even reversed selectivity, favoring the *meta*‐isomer to varying extents (**3fb** and **3gb**). This substituent effect, together with the consistent *meta*‐selectivity independent from the steric and electronic profiles of the Grignard reagents, is reminiscent of those observed in nucleophilic additions to arynes derived from related cyclic diarylhalonium salts [16, 17, 18, 19, 20, 21] and may be qualitatively rationalized by the aryne‐distortion model [32], together with a possible chelating influence of the methoxy group (as in **3eb**). An unsymmetrical derivative possessing a single site of deprotonation and lacking substituents proximal to the aryne‐forming position restored the characteristic *meta*‐selectivity (**3ib**, **3jf**, and **3kf**), consistent with an inherent bias in the carbomagnesiation step. Another unsymmetrical iodonium salt, the 4‐methoxy derivative, underwent selective phenylation on the methoxy‐bearing ring to give the *ortho*‐isomer **3lb** as the major product along with other minor regioisomers; in this case, the methoxy group governed both the site of deprotonation and the regioselectivity of carbomagnesiation. The reaction was readily scalable: 1 mmol‐scale reactions delivered representative products (**3ab**, **3ag**, **3am**, **3aq**, **3gb**, and **3hb**) in yields comparable to those obtained on a smaller scale, underscoring the robustness of the transformation.

**SCHEME 4:**
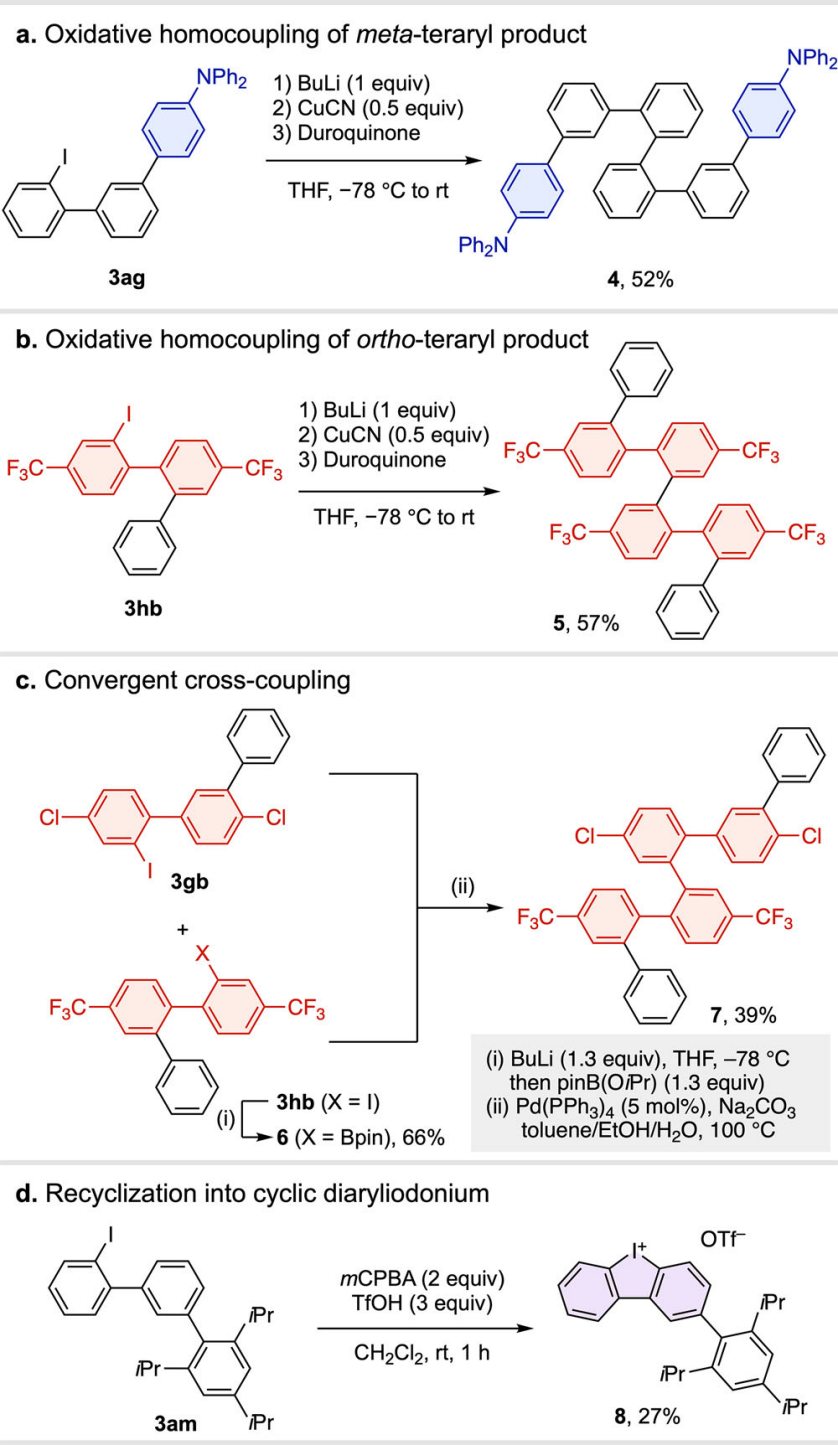
Transformations of iodine‐capped teraryls.

**FIGURE 1:**
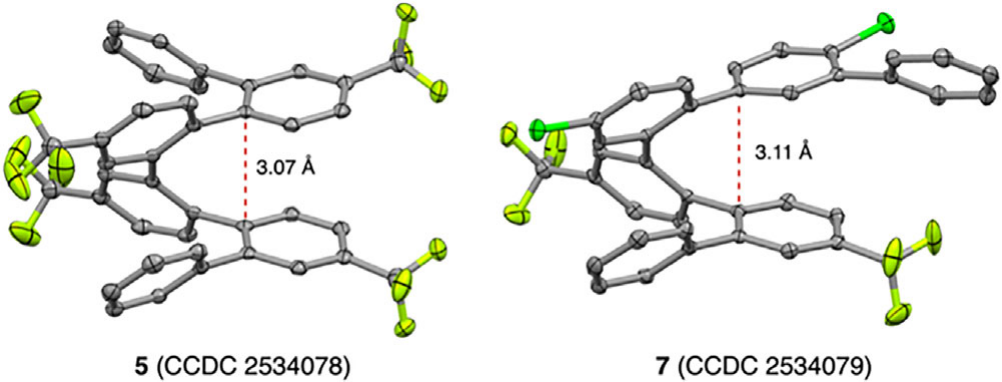
Solid‐state structures of **5** and **7** with thermal ellipsoids at the 50% probability level.

The C─I bond embedded within the teraryl framework provides a versatile handle for further structural elaboration (Scheme 4). Halogen–lithium exchange of *meta*‐teraryl **3ag**, followed by transmetalation to copper and oxidative homocoupling, furnished the terminally diphenylamino‐functionalized hexaarylene **4** with the *meta*, *ortho*, *ortho*, *meta* connectivity (Scheme 4a). Likewise, *ortho*‐teraryl **3hb** was converted to the internally trifluoromethyl‐substituted *ortho*‐hexaarylene **5** (Scheme 4b). Meanwhile, a Suzuki–Miyaura coupling of *meta*‐teraryl **3gb** with boronic ester **6**, prepared from *ortho*‐teraryl **3hb**, enabled convergent assembly of hexaarylene **7**, featuring predominant *ortho* linkage with a *meta*‐connected terminus (Scheme 4c). The ^1^H NMR spectra (CDCl_3_) of hexaarylenes **4**, **5**, and **7** each displayed a single dominant set of signals. In particular, **5** and **7** exhibited a characteristic upfield‐shifted aromatic resonance around 6 ppm, consistent with a helically folded structure [23]. Single‐crystal X‐ray diffraction analysis of **5** and **7** revealed helically twisted conformations in the solid state (Figure 1). Beyond outward chain extension, the residual C─I bond could also be directed inward, effecting re‐cyclization of **3am** to afford aryl‐substituted cyclic diaryliodonium salt **8** (Scheme 4d). Together, these transformations underscore the synthetic flexibility of the iodoteraryl scaffold.

**FIGURE 2:**
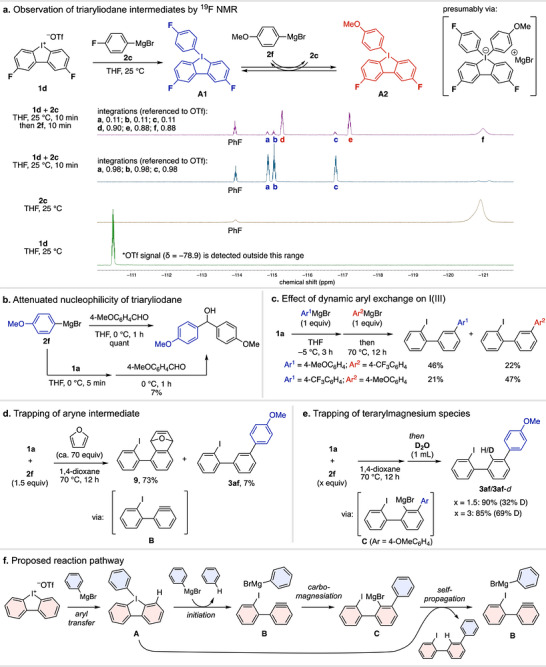
Mechanistic experiments and proposed reaction pathway.

The immediate yellow coloration, followed by gradual fading, suggested the transient formation and subsequent consumption of a discrete iodine‐containing intermediate. The nature of this species was examined by ^19^F NMR spectroscopy using a symmetric difluoro‐substituted iodonium salt **1d** and 4‐FC_6_H_4_MgBr (**2c**; Figures 2a and S3–S6). When combined in a 1:1 ratio in THF at room temperature, the starting materials largely disappeared from the ^19^F NMR spectrum, and three new signals of equal intensity emerged. This pattern is consistent with the formation of a T‐shaped cyclic triaryliodane **A1** in which the originally equivalent fluorine atoms on the iodonium become inequivalent [30]. Addition of one equivalent of 4‐MeOC_6_H_4_MgBr (**2f**) led to partial regeneration of the 4‐FC_6_H_4_MgBr signal, attenuation of the original triaryliodane signals, and appearance of two new resonances assignable to another triaryliodane **A2**. These observations are consistent with aryl ligand exchange between the initially formed triaryliodane and the added Grignard reagent, which likely occurs through a tetracoordinated iodine(III) ate species [33]. The signals of **A1** and **A2** remained well resolved even at 60°C (Figure S7), indicating that both isomerization of the T‐shaped triaryliodanes (via a Y‐shaped geometry) [30] and Grignard‐mediated aryl ligand exchange between distinct triaryliodanes are slow on the ^19^F NMR timescale, while gradual decomposition via ring‐opening arylation was observed upon heating.

The formation of cyclic triaryliodane was further substantiated by complementary computational and experimental evidence. A model DFT calculation indicated that conversion of the parent cyclic diaryliodonium triflate (**1a**) and PhMgBr•dioxane to the corresponding cyclic triaryliodane and MgBrOTf•dioxane is exergonic by approximately 10 kcal/mol (Scheme S13), supporting the thermodynamic feasibility of this transformation. Consistent with this picture, a 1:1 mixture of **1a** and **2f** exhibited markedly diminished nucleophilicity relative to the free Grignard reagent, as demonstrated by trapping experiments with 4‐methoxybenzaldehyde (Figure 2b). Furthermore, ring‐opening arylation employing two distinct aryl Grignard reagents afforded similar product distributions irrespective of the order of addition (Figure 2c and Table S3), in line with dynamic aryl ligand exchange between cyclic triaryliodane and excess Grignard reagents.

Having established the formation and dynamic behavior of cyclic triaryliodane, we next examined its role in aryne generation. The addition of furan to the reaction between **1a** and **2f** afforded the [4+2] cycloadduct **9**, along with a minor amount of the arylative ring‐opening product **3af** (Figure 2d), thereby confirming the in situ generation of 3‐(2‐iodophenyl)benzyne (**B**). This observation indicates that the aryl Grignard reagent serves not only as a nucleophile but also as a base that initiates aryne formation. Notably, the arylation of **1a** proceeded in 68% yield with only 1.1 equiv of the Grignard reagent **2f**. Because one equivalent is consumed in forming the poorly nucleophilic triaryliodane, the remaining 0.1 equiv cannot account for this conversion unless a regenerative process is operative.

To probe the origin of this high conversion, deuterium‐labeling experiments were conducted (Figure 2e). Quenching the standard reaction of **1a** with **2f** (1.5 equiv) with D_2_O afforded **3af** bearing 32% deuterium incorporation at the central benzene ring, consistent with formation of a terarylmagnesium intermediate **C**. Increasing the loading of **2f** to 3 equiv raised the deuterium incorporation to 69%, supporting the role of **C** as a base that sustains aryne generation under limiting Grignard conditions. In either case, deprotonative aryne formation regenerates an arylmagnesium species capable of subsequent carbomagnesiation, thereby enabling a self‐propagating sequence.

Taken together, these findings, along with established precedents for cyclic triaryliodanes [27, 29, 30, 33], support a self‐propagating mechanism (Figure 2f). Nucleophilic aryl transfer from ArMgBr to the cyclic diaryliodonium salt rapidly furnishes a cyclic triaryliodane **A**. Although this species predominantly undergoes reversible aryl exchange with excess Grignard reagent, occasional irreversible deprotonation generates ArH and 3‐(2‐iodophenyl)benzyne **B**, concomitantly regenerating ArMgBr. The overall transformation is best viewed as deprotonation followed by a halogen–metal exchange–like reorganization within a magnesium‐associated aggregate, rather than as a discrete departure of a phenyl anion [34]. The aryne then undergoes carbomagnesiation with either newly formed or pre‐existing ArMgBr to give an *ortho*‐diarylphenylmagnesium intermediate, which in turn acts as a base to sustain further aryne formation and thereby closes the propagation cycle. Self‐propagating aryne formation has precedent in reactions of *ortho*‐dihalobenzenes with aryllithium or Grignard reagents via halogen–metal exchange [35, 36, 37, 38, 39]. In contrast, the present system operates through deprotonation of a cyclic triaryliodane, representing a mechanistic variant of this broader concept. In light of the structural reassignment by Hellwinkel and co‐workers [29], we briefly reexamined the thermolysis of the corresponding triaryliodane under lithium conditions. Treatment of **1a** with phenyllithium, followed by thermolysis, furnished the *meta*‐teraryl **3ab** (23%), with no detectable formation of the *ortho*‐isomer. Given the revised characterization of the authentic *ortho*‐isomer (mp 80°C–83°C) [40], the originally reported product (mp 53°C–54°C) is consistent with the *meta*‐isomer. While earlier interpretations invoked direct ligand coupling under thermolytic conditions [28, 29], the possibility of aryne involvement cannot be excluded.

**SCHEME 5:**
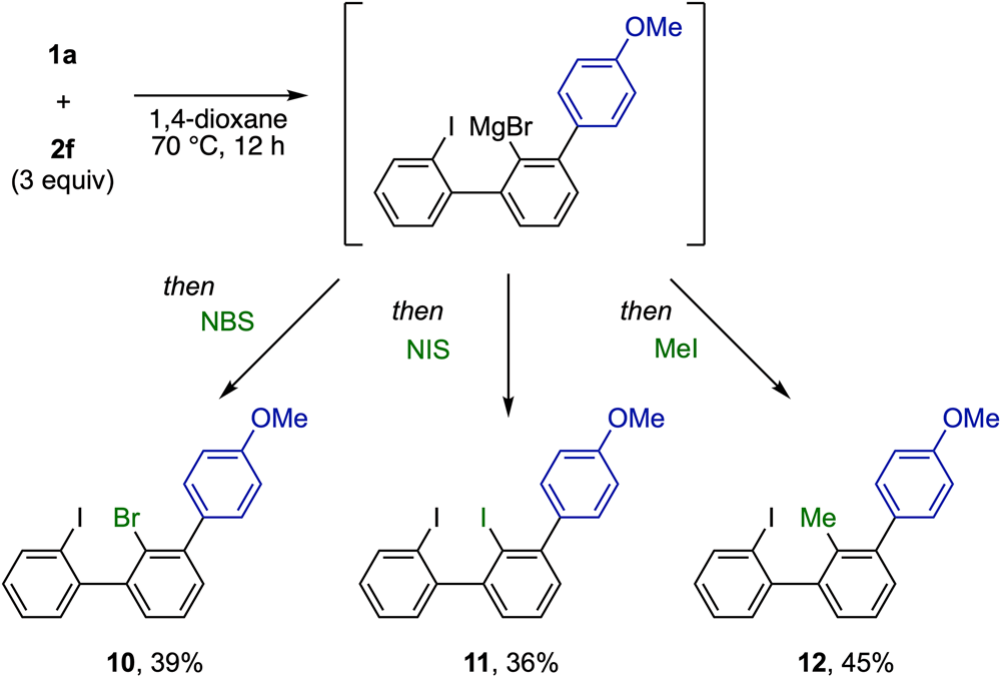
Harnessing terarylmagnesium species for electrophilic trapping.

Beyond serving as a propagating intermediate, the terarylmagnesium species could be preserved under appropriately tuned conditions and directly engaged with external electrophiles (Scheme 5). Guided by the deuterium‐labeling results, the reaction was conducted with 3 equiv of Grignard reagent **2f**, and subsequent trapping with different electrophiles furnished the brominated (**10**), iodinated (**11**), and methylated (**12**) derivatives. Importantly, all of these products retained the original C─I bond, providing a versatile synthetic handle for downstream diversification. These findings demonstrate that the present transformation not only enables self‐propagating aryne generation but also provides a platform for the modular assembly of structurally diverse and highly functionalized oligoarylene architectures.

## 3 Conclusions

In summary, we have established a broadly applicable ring‐opening arylation of cyclic diaryliodonium salts with diverse aryl Grignard reagents. Central to this transformation is the in situ formation of a cyclic triaryliodane intermediate that engages in a self‐propagating cycle of aryne generation and carbometalation, representing a distinct propagation mode in aryne chemistry. Although the chemistry of cyclic triaryliodanes has been explored for decades, its potential as a practical and selective synthetic platform has remained largely unrealized. The present work revisits this classical main‐group reactivity from a modern perspective, demonstrating that controlled triaryliodane formation can be harnessed to efficiently access iodoteraryl frameworks. The intact C─I bond enables both outward extension into oligoarylene architectures and inward re‐cyclization into novel cyclic iodonium salts, while the preserved terarylmagnesium intermediates can be diverted toward further electrophilic functionalization. Taken together, these findings expand the conceptual and synthetic scope of hypervalent iodine chemistry and open new avenues for the construction of structurally complex aryl frameworks.

## Conflicts of Interest

The authors declare no conflicts of interest.

## Supporting information




**Supporting File 1**: anie72419‐sup‐0001‐Data.zip.

**Supporting File 2**: anie72419‐sup‐0001‐SuppMat.pdf.

## Data Availability

The data that supports the findings of this study are available in the Supporting Information of this article

## References

[1] A. Yoshimura and V. V. Zhdankin , “Recent Progress in Synthetic Applications of Hypervalent Iodine(III) Reagents,” Chemical Reviews 124 (2024): 11108–11186, 10.1021/acs.chemrev.4c00303. 39269928 PMC11468727

[2] A. Yoshimura and V. V. Zhdankin , “Advances in Synthetic Applications of Hypervalent Iodine Compounds,” Chemical Reviews 116 (2016): 3328–3435, 10.1021/acs.chemrev.5b00547. 26861673

[3] X. Peng , A. Rahim , W. Peng , F. Jiang , Z. Gu , and S. Wen , “Recent Progress in Cyclic Aryliodonium Chemistry: Syntheses and Applications,” Chemical Reviews 123 (2023): 1364–1416, 10.1021/acs.chemrev.2c00591. 36649301 PMC9951228

[4] H.‐C. Cheng , J.‐L. Ma , and P.‐H. Guo , “Cyclic Diaryliodonium Salts: Eco‐Friendly and Versatile Building Blocks for Organic Synthesis,” Advanced Synthesis & Catalysis 365 (2023): 1112–1139, 10.1002/adsc.202201326.

[5] R. Singhal , S. P. Choudhary , B. Malik , and M. Pilania , “Cyclic Diaryliodonium Salts: Applications and Overview,” Organic & Biomolecular Chemistry 21 (2023): 4358–4378, 10.1039/D3OB00134B. 37161758

[6] D. Zhu , Q. Liu , B. Luo , et al., “Synthesis of Carbazoles via One‐Pot Copper‐Catalyzed Amine Insertion into Cyclic Diphenyleneiodoniums as a Strategy to Generate a Drug‐like Chemical Library,” Advanced Synthesis & Catalysis 355 (2013): 2172–2178, 10.1002/adsc.201300271.

[7] Y. Wu , X. Peng , B. Luo , et al., "Palladium Catalyzed Dual C‐H Functionalization of Indoles With Cyclic Diaryliodoniums, an Approach to Ring Fused Carbazole Derivatives," Organic & Biomolecular Chemistry 12 (2014): 2777–9780, 10.1039/C4OB02170C. 25357010

[8] B. Wu and N. Yoshikai , “Conversion of 2‐Iodobiaryls into 2,2′‐Diiodobiaryls via Oxidation‐Iodination Sequences: A Versatile Route to Ladder‐Type Heterofluorenes,” Angewandte Chemie International Edition 54 (2015): 8736–8739, 10.1002/anie.201503134. 26058358

[9] K. Zhu , Z. Song , Y. Wang , and F. Zhang , “Synthesis of 2, 2′‐dihalobiaryls via Cu‐Catalyzed Halogenation of Cyclic Diaryliodonium Salts,” Organic Letters 22 (2020): 9356–9359, 10.1021/acs.orglett.0c03614. 33215922

[10] M. Ding , W. Hua , M. Liu , and F. Zhang , “Pd‐Catalyzed C(sp_3_)–H Biarylation via Transient Directing Group Strategy,” Organic Letters 22 (2020): 7419–7423, 10.1021/acs.orglett.0c02353. 32946696

[11] K. Zhao , L. Duan , S. Xu , J. Jiang , Y. Fu , and Z. Gu , “Enhanced Reactivity by Torsional Strain of Cyclic Diaryliodonium in Cu‐Catalyzed Enantioselective Ring‐Opening Reaction,” Chem 4 (2018): 599–612, 10.1016/j.chempr.2018.01.017.

[12] X. Zhang , K. Zhao , and Z. Gu , “Transition Metal‐Catalyzed Biaryl Atropisomer Synthesis via a Torsional Strain Promoted Ring‐Opening Reaction,” Accounts of Chemical Research 55 (2022): 1620–1633, 10.1021/acs.accounts.2c00175. 35647705

[13] S. Yang , T. Zheng , L. Duan , X. Xue , and Z. Gu , “Atroposelective Three‐Component Coupling of Cyclic Diaryliodoniums and Sodium Cyanate Enabled by the Dual‐Role of Phenol,” Angewandte Chemie International Edition 62 (2023): e202302749. 36947004 10.1002/anie.202302749

[14] W. Fang , Y.‐D. Meng , S.‐Y. Ding , et al., “Asymmetric S ‐Arylation of Sulfenamides to Access Axially Chiral Sulfilimines Enabled by Anionic Stereogenic‐at‐Cobalt(III) Complexes,” Angewandte Chemie International Edition 64 (2025): e202419596, 10.1002/anie.202419596. 39625341

[15] H. Yang , X. Sun , Z. Chen , et al., “Coplanar Atropochiral 5*H*‐Cyclopenta[2,1‐*b*:3,4‐*b*′]dipyridine Ligands: Synthesis and Applications in Asymmetric Ring‐Opening Reaction,” Angewandte Chemie International Edition 137 (2025): e202416839, 10.1002/anie.202416839. 39377116

[16] M. Lanzi , Q. Dherbassy , and J. Wencel‐Delord , “Cyclic Diaryl λ^3^ ‐Bromanes as Original Aryne Precursors,” Angewandte Chemie International Edition 60 (2021): 14852–14857, 10.1002/anie.202103625. 33901330

[17] M. Lanzi , R. A. A. Abdine , M. De Abreu , and J. Wencel‐Delord , “Cyclic Diaryl λ^3^‐Bromanes: A Rapid Access to Molecular Complexity via Cycloaddition Reactions,” Organic Letters 23 (2021): 9047–9052, 10.1021/acs.orglett.1c03278. 34806390

[18] M. Lanzi , T. Rogge , T. S. Truong , K. N. Houk , and J. Wencel‐Delord , “Cyclic Diaryl λ^3^ ‐Chloranes: Reagents and Their C–C and C–O Couplings with Phenols via Aryne Intermediates,” Journal of the American Chemical Society 145 (2023): 345–358, 10.1021/jacs.2c10090. 36535642 PMC9837845

[19] M. Lanzi and J. Wencel‐Delord , “Diaryl Hypervalent Bromines and Chlorines: Synthesis, Structures and Reactivities,” Chemical Science 15 (2024): 1557–1569, 10.1039/D3SC05382B. 38303936 PMC10829020

[20] M. Liu , H. Jiang , J. Tang , Z. Ye , F. Zhang , and Y. Wu , “Meta‐Selective O‐Arylation of Cyclic Diaryliodonium Salts With Phenols via Aryne Intermediates,” Organic Letters 25 (2023): 2777–2781, 10.1021/acs.orglett.3c00600. 37058147

[21] H. Jiang , M. Liu , Z. Ye , Z. Song , Y. Wu , and F. Zhang , “Metal‐Free Meta‐Halogenation of Cyclic Diaryliodonium Salts via Aryne Intermediates,” Organic Chemistry Frontiers 10 (2023): 3856–3860, 10.1039/D3QO00570D.

[22] K. Liu , L. Bai , S. Deng , X. Qi , and X. Jiang , “Divergent Aryne Regulation for Polycyclic Aromatic Hydrocarbons,” Science Bulletin 70 (2025): 2967–2976, 10.1016/j.scib.2025.07.026. 40744788

[23] C. S. Hartley , “Folding of *ortho*‐Phenylenes,” Accounts of Chemical Research 49 (2016): 646–654, 10.1021/acs.accounts.6b00038. 26954326

[24] H. Ito , Y. Segawa , K. Murakami , and K. Itami , “Polycyclic Arene Synthesis by Annulative π‐Extension,” Journal of the American Chemical Society 141 (2019): 3–10, 10.1021/jacs.8b09232. 30395456

[25] Y. Zhang , J. Han , and Z.‐J. Liu , “Palladium‐Catalyzed Double‐Suzuki–Miyaura Reactions Using Cyclic Dibenziodoniums: Synthesis of o‐Tetraaryls,” Journal of Organic Chemistry 81 (2016): 1317–1323, 10.1021/acs.joc.5b02255. 26608348

[26] K. Clauss , “Über Diphenylen‐phenyl‐jod,” Chemische Berichte 88 (1955): 268–270, 10.1002/cber.19550880217.

[27] F. M. Beringer and L. L. Chang , “Electrophilic and Homolytic Cleavage of 5‐aryl‐5H‐Dibenziodoles,” Journal of Organic Chemistry 36 (1971): 4055–4060, 10.1021/jo00825a011.

[28] G. Wittig and D. Hellwinkel , “Über Bildungsweisen und Verhalten Pentaarylierter Spiroarsene,” Chemische Berichte 97 (1964): 769–788, 10.1002/cber.19640970320.

[29] D. Hellwinkel , M. Haltmeier , and G. Reiff , “Thermische Zersetzung von 2,2'‐Biphenylylen(phenyl)Jod und Ähnlichen Verbindungen,” Justus Liebigs Annalen Der Chemie 1975 (1975): 249–254, 10.1002/jlac.197519750209.

[30] H. J. Reich and C. S. Cooperman , “Structure and Stereolability of Triaryliodine (III) Compounds. Degenerate Isomerization of 5‐Phenyl‐5*H*‐Dibenziodole,” Journal of the American Chemical Society 95 (1973): 5077–5078, 10.1021/ja00796a065.

[31] CCDC 2498673 (**3as**), 2534078 (**5**), and 2534079 (**7**) contain the Supplementary Crystallographic Data for this Paper. These data are provided free of charge by the Joint Cambridge Crystallographic Data Center and Fachinformationszentrum Karlsruhe Access Structures Service, https://www.ccdc.cam.ac.uk/structures.

[32] J. M. Medina , J. L. Mackey , N. K. Garg , and K. N. Houk , “The Role of Aryne Distortions, Steric Effects, and Charges in Regioselectivities of Aryne Reactions,” Journal of the American Chemical Society 136 (2014): 15798–15805, 10.1021/ja5099935. 25303232 PMC4221504

[33] F. M. Beringer and L. L. Chang , “Exchange of Aryl Ligands to Polyvalent Iodine,” Journal of Organic Chemistry 37 (1972): 1516–1519, 10.1021/jo00975a011.

[34] H. J. Reich , “What's Going on With These Lithium Reagents?,” Journal of Organic Chemistry 77 (2012): 5471–5491, 10.1021/jo3005155. 22594379

[35] J. A. García‐López and M. F. Greaney , “Synthesis of Biaryls Using Aryne Intermediates,” Chemical Society Reviews 45 (2016): 6766–6798, 10.1039/C6CS00220J. 27752670

[36] N. Kim , M. Choi , S.‐E. Suh , and D. M. Chenoweth , “Aryne Chemistry: Generation Methods and Reactions Incorporating Multiple Arynes,” Chemical Reviews 124 (2024): 11435–11522, 10.1021/acs.chemrev.4c00296. 39383091

[37] F. Leroux and M. Schlosser , “The “Aryne” Route to Biaryls Featuring Uncommon Substituent Patterns,” Angewandte Chemie International Edition 41 (2002): 4272–4274, 10.1002/1521-3773(20021115)41:22<4272::AID-ANIE4272>3.0.CO;2-B. 12434360

[38] F. R. Leroux , A. Berthelot , L. Bonnafoux , A. Panossian , and F. Colobert , “Transition‐Metal‐Free Atropo‐Selective Synthesis of Biaryl Compounds Based on Arynes,” Chemical European Journal 18 (2012): 14232–14236, 10.1002/chem.201202739. 23019081

[39] A. Berthelot‐Bréhier , A. Panossian , F. Colobert , and F. R. Leroux , “Atroposelective Synthesis of Axially Chiral P,S‐Ligands Based on Arynes,” Organic Chemistry Frontiers 2 (2015): 634–644, 10.1039/C5QO00067J.

[40] S. Karmakar , T. Mandal , and J. Dash , “Ring Closing Metathesis Approach for the Synthesis of o‐Terphenyl Derivatives,” European Journal of Organic Chemistry 2019 (2019): 5916–5924, 10.1002/ejoc.201900988.

