# Supplementary material for: Ring‐Opening Arylation of Cyclic Diaryliodoniums via Cyclic Triaryliodanes as Aryne Precursors and Dynamic Aryl Reservoirs
Source: Angew Chem Int Ed Engl. 2026 May 1;65(24):e6935149. doi: 10.1002/anie.6935149 (PMC13245596; doi:10.1002/anie.6935149)

Supporting Information

Ring-Opening Arylation of Cyclic Diaryliodoniums via Cyclic Triaryliodanes as Aryne Precursors and Dynamic Aryl Reservoirs

Daichi Ikeshita, Mao Atarashi, and Naohiko Yoshikai*

Graduate School of Pharmaceutical Sciences, Tohoku University
6-3 Aoba, Aramaki, Aoba-ku, Sendai 980-8578, Japan
E-mail: naohiko.yoshikai.c5@tohoku.ac.jp

Contents

1. Materials and Methods S2
2. Ring-Opening Arylation of Cyclic Diaryliodonium Salts S5
3. Product Transformations S22
4. Mechanistic Experiments S27
5. Electrophilic Trapping of Terarylmagnesium Intermediate S38
6. X-Ray Crystallographic Analysis S40
7. DFT Calculations S46
8. References S51
9. ^{1}H and ^{13}C NMR Spectra S52

1. Materials and Methods

General. All reactions involving air- or moisture-sensitive compounds were performed using standard Schlenk techniques in oven-dried reaction vessels under an argon atmosphere. Analytical thin-layer chromatography (TLC) was performed on Merck 60 F254 silica gel plates. Column chromatography was performed using flash chromatography with 40–63 μm silica gel (Silica Gel 60N, Kanto Chemical Co., Inc.). ^{1}H, ^{13}C, and ^{19}F nuclear magnetic resonance (NMR) spectra were recorded on a Varian Mercury (400 MHz) or a JEOL-ECA600 (600 MHz) spectrometer. ^{1}H and ^{13}C NMR chemical shifts are reported in parts per million (ppm) downfield from an internal standard or the residual solvent peak: tetramethylsilane (0.00 ppm for ^{1}H in $CDCl_3$), $CHCl_3$ (77.1 ppm for ^{13}C in $CDCl_3$), and dimethyl sulfoxide (DMSO, 2.54 ppm for ^{1}H in DMSO-d_6, 39.5 ppm for ^{13}C in DMSO-d_6). ^{19}F NMR spectra are referenced to external standard (CF_3CO_2H, –76.6 ppm in $CDCl_3$). Melting points were determined with a MPA100 OptiMelt apparatus. High-resolution mass spectra (HRMS) were recorded on a JEOL JMS-DX-303, a JEOL JMS-700, or a JEOL JMS-T100GC spectrometer with a magnetic sector time-of-flight mass analyzer. Gel permeation chromatography (GPC) was performed using a Japan Analytical Industry LabACE LC-5060 [JAIGEL-2HR Plus (20 × 600 mm)] with $CHCl_3$ as the eluent at a flow rate of 7.0 mL/min.

Materials. Unless otherwise noted, commercial reagents were purchased from Tokyo Chemical Industry Co., Ltd., Kanto Chemical Co., Inc., Signa-Aldrich Japan, FUJIFILM Wako Pure Chemical Corporation, and other commercial suppliers and were used without further purification. Anhydrous 1,4-dioxane, toluene, and tetrahydrofuran (THF) were purchased from FUJIFILM Wako Pure Chemical Corporation (Wako). Figure S1 summarizes the cyclic diaryliodonium salts used in this study. The cyclic diaryliodonium salts were synthesized according to the general procedure described below.

Preparation of cyclic diaryliodonium salts

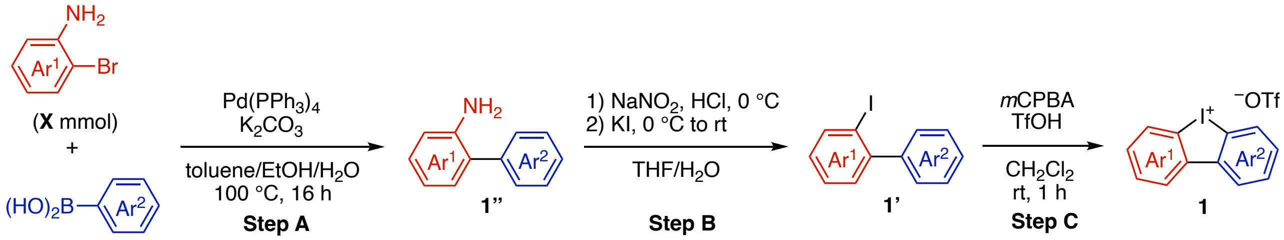

[Step A] general procedure for the preparation of 2-Aminobiaryl Derivative (1”): A three-necked flask equipped with a magnetic stir bar was charged with an arylboronic acid (1.5 equiv), K_2CO_3 (4 equiv), $Pd(PPh_3)_4$ (10 mol%), toluene, H_2O, and EtOH (0.13 M, 4/1/1). To the resulting solution was added a 2-bromoaniline derivative (1 equiv), and the reaction mixture was heated at 100 °C for 16 h. After cooling to room temperature, the biphasic mixture was diluted with saturated aqueous NH_4Cl and CH_2Cl_2. The aqueous phase was extracted with CH_2Cl_2, and the combined organic extracts were washed with water and saturated aqueous $NaHCO_3$, dried over $MgSO_4$, and concentrated under reduced pressure. The residue was purified by flash chromatography on silica gel to afford the desired 2-aminobiaryl derivative.

[Step B] general procedure for the preparation of 2-iodobiaryl (1’): To a stirred solution of 2-aminobiaryl 1’ (1 equiv) in THF (0.40 M) was added TsOH•H_2O (3 equiv), and the solution was cooled in an ice bath. A solution of $NaNO_2$ (1.2 equiv) in H_2O (2 mL) was added dropwise. After 20 min, a solution of KI (2.5 equiv) in H_2O (2 mL) was added, and upon additional stirring for 10 min, the reaction mixture was slowly warmed to room temperature and stirred for another 1 h. Then 1M aqueous $Na_2S_2O_3$ was added portionwise until the color of the mixture did not change further. The organic and aqueous phases were separated, and the latter was extracted with EtOAc. The combined organic layers were washed with H_2O and brine, dried over Na_2SO_4, and concentrated under reduced pressure. The residue was purified by flash chromatography on silica gel to afford the desired 2-iodobiaryl.

[Step C] general procedure for the preparation of cyclic diaryliodonium salts (1): To a stirred solution of 2-iodobiaryl (1 equiv) in anhydrous CH_2Cl_2 (0.25 M) was added *m*CPBA (1.5 equiv) and TfOH (3 equiv). The solution was stirred for 1 h at room temperature, followed by removal of CH_2Cl_2 by rotary evaporation. To the solid residue was added Et_2O, and the resulting mixture was stirred for 20 min. The solid precipitate was collected by vacuum filtration, washed with Et_2O for three times, and dried under vacuum to afford the cyclic diaryliodonium triflate.

Figure S1 summarizes the cyclic diaryliodonium salts used in this study and their isolated yields from the corresponding precursors. The spectral data of **1a**,[1] **1b**,[2] **1c**,[3] **1d**,[3] **1e**,[1] **1f**,[3] **1g**,[3] **1h**,[3] **1i**,[4] **1j**,[5] **1k**,[6] and **1l** [7] were in good agreement with the literature values.

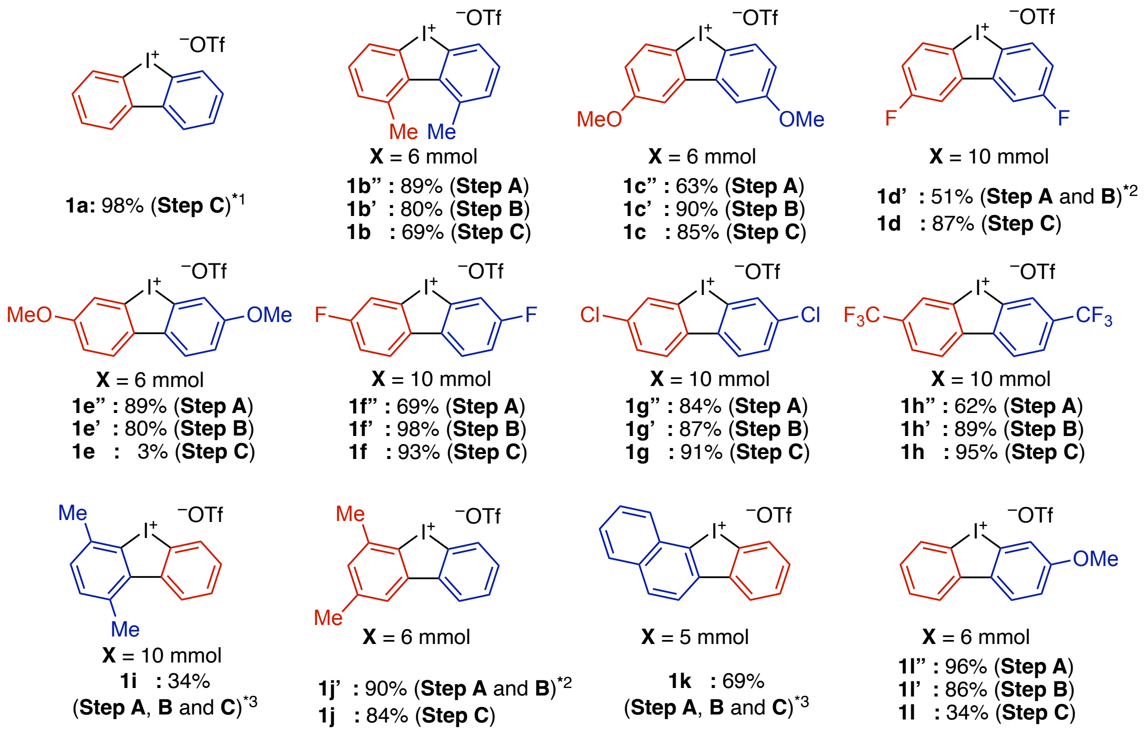

*1 2-Iodobiphenyl (**1a'**) was purchased commercially. A 20 mmol scale of **1a'** was used in Step C without further purification.
*2 The yields are shown over two steps because impurities could not be separated from **1"** after purification in **step A**.
*3 The yields are shown over three steps because impurities could not be separated from **1"** and **1'** after purification in **step A** and **step B**.

Figure S1. Cyclic diaryliodonium salts used in this study.

2. Ring-Opening Arylation of Cyclic Diaryliodonium Salts

Table S1. Ring-opening arylation of **1a** with **2a**: Effect of reaction conditions.

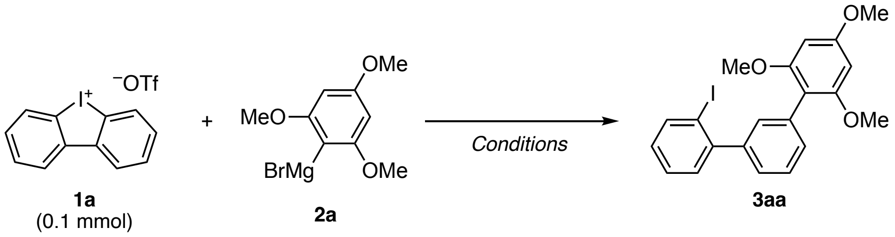

Entry	Conditions	Yield[a]
1	THF (2 mL), rt, 12 h<br>2 equiv of **2a**	46%
2	THF (2 mL), 70 °C, 5 h<br>2 equiv of **2a**	68%
3	THF (2 mL), 70 °C, 12 h<br>2 equiv of **2a**	94%
4	THF (2 mL), 70 °C, 12 h<br>1.5 equiv of **2a**	89%
5	1,4-dioxane (2 mL), 70 °C, 12 h<br>1.5 equiv of **2a**	98% (94%)[b]
6	1,4-dioxane (2 mL), 70 °C, 12 h<br>1.2 equiv of **2a**	65%

[a]Determined by ^{1}H NMR analysis using 1,1,2,2-tetrachloroethane as an internal standard. [b]Isolated yield.

Table S2. Ring-opening arylation of **1a** with PhMgBr (**2b**) or 4-MeOC_6H_4MgBr (**2f**): Effect of reaction conditions.

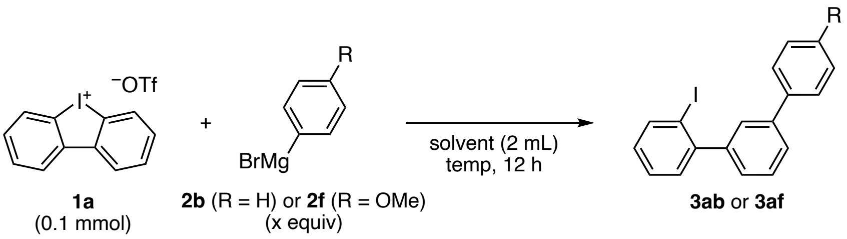

Entry	R	x	solvent	temp (°C)	yield[a]
1	H	1.2	THF	50	12%
2	H	1.2	THF	70	70%
3	H	1.2	dioxane	70	72%
4	H	1.2	hexane	70	66%
5	H	1.2	toluene	70	61%
6	H	1.5	dioxane	70	98%
7	OMe	1.2	dioxane	70	72%
8	OMe	1.1	dioxane	70	68%

[a]Determined by ^{1}H NMR analysis using 1,1,2,2-tetrachloroethane as an internal standard.

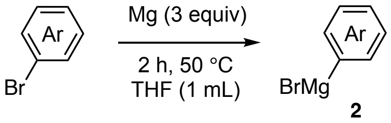

General procedure for the preparation of aryl Grignard reagents: Under an argon atmosphere, a flame-dried 10 mL Schlenk tube equipped with a magnetic stir bar was charged with magnesium turnings (3.0 mmol, 3.0 equiv). Solid aryl bromide (1.0 mmol) was added at this stage, whereas liquid aryl bromide (1.0 mmol) was introduced after the tube had been purged with argon, together with anhydrous THF (1.0 mL) and 1,2-dibromoethane (2 drops). The mixture was stirred at 50 °C for 2 h. The concentration of the resulting Grignard reagent was determined by titration with iodine in THF.

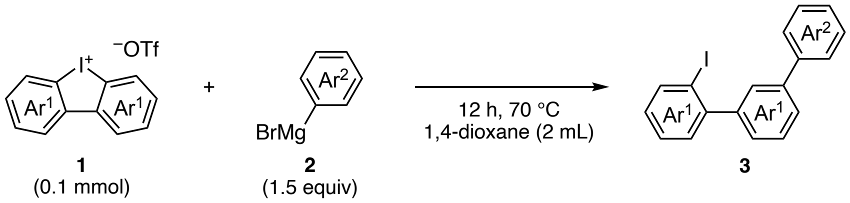

General procedure for the ring-opening arylation: A flame-dried 10 mL Schlenk tube equipped with a magnetic stir bar was charged with cyclic diaryliodonium salt **1** (0.10 mmol, 1.0 equiv). The tube was purged with argon, and anhydrous 1,4-dioxane (2 mL) was added. The tube was then placed in a preheated aluminum block (70 °C), and a THF solution of aryl Grignard reagent **2** (0.15 mmol, 1.5 equiv; typically ca. 1 M) was added dropwise via syringe. The reaction mixture was stirred at 70 °C for 12 h. After cooling to room temperature, saturated aqueous NH_4Cl solution was added, and the mixture was extracted with CH_2Cl_2 (5 mL × 3). The combined organic layers were dried over $MgSO_4$ and concentrated under reduced pressure. The residue was purified by preparative thin-layer chromatography to afford the desired product **3**. Unless otherwise noted, the regioisomeric ratio of products was determined by ^{1}H NMR analysis after purification.

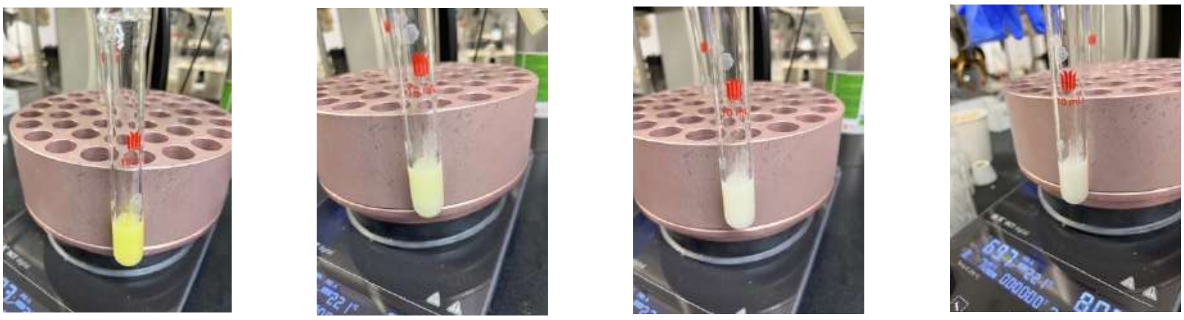

t = 0 h   *t* = 1 h   *t* = 5 h   *t* = 12 h

Figure S2. Color change of the reaction mixture for the arylation of **1a** with 4-methoxyphenylmagnesium bromide (**2f**).

Procedure for the 1 mmol-scale synthesis of 3ab, 3ag, 3am, 3aq, 3gb, and 3hb: A flame-dried 50 mL two-necked round-bottom flask equipped with a magnetic stir bar was charged with cyclic diaryliodonium salt **1** (1.0 mmol, 1.0 equiv). The flask was purged with argon, and anhydrous 1,4-dioxane (20 mL) was added. The flask was then placed in a preheated oil bath (70 °C), and a solution of aryl Grignard reagent **2** (1.5 mmol, 1.5 equiv; typically ca.1 M) was added dropwise via syringe. The reaction mixture was stirred at 70 °C for 12 h. After cooling to room temperature, saturated NH_4Cl aqueous solution was added, and the mixture was extracted with CH_2Cl_2 (20 mL × 3). The combined organic layers were dried over $MgSO_4$ and concentrated under reduced pressure. The residue was purified by silica-gel column chromatography to afford the desired product **3**.

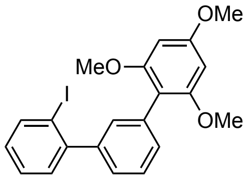

2''-Iodo-2,4,6-trimethoxy-1,1':3',1''-terphenyl (3aa): Pale brown oil (37.4 mg, 94% yield); R_f 0.5 (hexane/ethyl acetate = 3/1); **^{1}H NMR** (400 MHz, $CDCl_3$) δ 7.95 (d, J = 8.0 Hz, 1H), 7.43 (t, J = 7.7 Hz, 1H), 7.38−7.36 (m, 3H), 7.33 (t, J = 1.6 Hz, 1H), 7.24 (dt, J = 7.5, 1.6 Hz, 1H), 7.00 (ddd, J = 8.0, 5.2, 4.0 Hz, 1H), 6.23 (s, 2H), 3.85 (s, 3H), 3.73 (s, 6H); **^{13}C{^{1}H} NMR** (151 MHz, $CDCl_3$) δ 160.6, 158.5, 147.0, 143.3, 139.5, 133.7, 132.5, 130.6, 130.4, 128.6, 128.1, 127.2, 112.3, 98.8, 91.1, 56.1, 55.4; one aromatic resonance not resolved due to overlap; **HRMS** (EI^+) Calcd for $C_{21}H_{19}IO_3^+$ $[M]^+$ 446.0379, found 446.0388. The *meta*-connectivity was confirmed by the pair of triplet signals (one with a large J and another with a small J) characteristic of *meta*-disubstituted benzenes. This diagnostic triplet pair (or one of the pair) was also discernible for several other products, while in some cases not resolved due to signal overlap. The *meta*-connectivity of the remaining compounds, except those derived from 4,4'-disubstituted iodonium salts, was inferred by analogy and supported by the X-ray crystallographic analysis of **3at**.

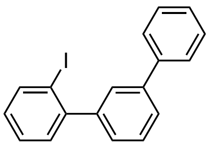

2-Iodo-1,1':3',1''-terphenyl (3ab): Colorless oil (32.1 mg, 90% yield on 0.1 mmol scale/292.6 mg, 82% yield on 1 mmol scale); R_f 0.4 (hexane/CH_2Cl_2 = 7/1); The regioisomeric ratio m:o = 97:3; **^{1}H NMR** (400 MHz, $CDCl_3$, major regioisomer) δ 7.98 (d, J = 8.0 Hz, 1H), 7.66−7.59 (m, 4H), 7.49 (t, J = 7.6 Hz, 1H), 7.46−7.31 (m, 6H), 7.04 (app td, J = 7.5, 1.9 Hz, 1H); **^{13}C{^{1}H} NMR** (151 MHz, $CDCl_3$) δ 146.6, 144.6, 141.0, 140.9, 139.7, 130.2, 129.0, 128.9, 128.5, 128.4,

128.3, 128.2, 127.5, 127.3, 126.4, 98.7; **HRMS** (EI$^+$) Calcd for $C_{18}H_{13}I^+$ $[M]^+$ 356.0062, found 356.0057.

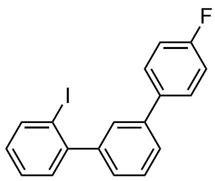

4''-Fluoro-2-iodo-1,1':3',1''-terphenyl (3ac): Colorless oil (28.1 mg, 70% yield); R_f 0.4 (hexane/CH_2Cl_2 = 8/1); The regioisomeric ratio *m*:*o* = 97:3; **^{1}H NMR** (600 MHz, $CDCl_3$, major regioisomer) δ 7.98 (d, *J* = 7.9 Hz, 1H), 7.61−7.59 (m, 2H), 7.56 (d, *J* = 7.9 Hz, 1H), 7.54 (d, *J* = 1.0 Hz, 1H), 7.49 (t, *J* = 7.7 Hz, 1H), 7.41 (app t, *J* = 7.4 Hz, 1H) , 7.35 (d, *J* = 7.4 Hz, 1H) , 7.32 (dt, *J* = 7.5, 1.1 Hz, 1H) , 7.13 (app t, *J* = 8.5 Hz, 2H), 7.05 (t, *J* = 7.6 Hz, 1H); **^{13}C{^{1}H} NMR** (151 MHz, $CDCl_3$) δ 162.6 (d, *J* = 246.4 Hz), 146.4, 144.7, 139.9, 139.7, 137.1 (d, *J* = 2.9 Hz), 130.2, 129.0, 128.9, 128.8, 128.6, 128.3 (d, *J* = 8.7 Hz), 128.2, 126.3, 115.8 (d, *J* = 21.3 Hz), 98.7; **^{19}F NMR** (376 MHz, $CDCl_3$): δ −115.5 (m); **HRMS** (EI$^+$) Calcd for $C_{18}H_{12}FI^+$ $[M]^+$ 373.9968, found 373.9966.

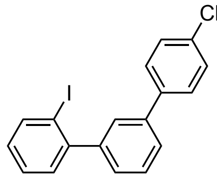

4''-Chloro-2-iodo-1,1':3',1''-terphenyl (3ad): Colorless oil (29.2 mg, 75% yield); R_f 0.5 (hexane/toluene = 8/1); The regioisomeric ratio *m*:*o* = 97:3; **^{1}H NMR** (400 MHz, $CDCl_3$, major regioisomer) δ 7.98 (dd, *J* = 7.9, 1.1 Hz, 1H), 7.59−7.54 (m, 4H), 7.49 (t, *J* = 7.6 Hz, 1H), 7.43−7.39 (m, 3H), 7.36−7.32 (m, 2H), 7.05 (ddd, *J* = 7.9, 7.2, 1.9 Hz, 1H); **^{13}C{^{1}H} NMR** (151 MHz, $CDCl_3$) δ 146.3, 144.8, 139.7, 139.6, 139.4, 133.6, 130.2, 129.1, 129.0, 128.7, 128.5, 128.4, 128.3, 128.2, 126.2, 98.7; **HRMS** (EI$^+$) Calcd for $C_{18}H_{12}ClI^+$ $[M]^+$ 389.9672, found 389.9680.

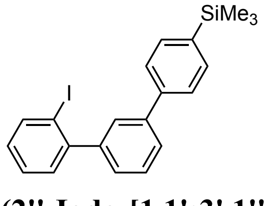

(2''-Iodo-[1,1':3',1''-terphenyl]-4-yl)trimethylsilane (3ae): Colorless oil (29.2 mg, 75% yield); R_f 0.5 (hexane/toluene = 8/1); **^{1}H NMR** (400 MHz, $CDCl_3$) δ 7.98 (dd, *J* = 8.0, 1.2 Hz, 1H), 7.65−7.59 (m, 6H), 7.49 (t, *J* = 7.4 Hz, 1H), 7.40 (td, *J* = 7.6, 1.2 Hz, 1H), 7.36 (dd, *J* = 7.6, 1.9 Hz, 1H), 7.32 (dt, *J* = 7.8, 1.4 Hz, 1H), 7.04 (td, *J* = 7.6, 1.7 Hz, 1H), 0.30 (s, 9H); **^{13}C{^{1}H} NMR** (151 MHz, $CDCl_3$) δ 146.6, 144.6, 141.3, 140.8, 139.7, 139.5, 133.9, 130.2,

129.0, 128.5, 128.35, 128.27, 128.2, 126.6, 126.4, 98.7, −1.0; **HRMS** (EI$^+$) Calcd for $C_{21}H_{21}ISi^+$ [M]$^+$ 428.0457, found 428.0459.

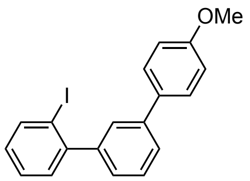

2-Iodo-4''-methoxy-1,1':3',1''-terphenyl (3af): Colorless oil (36.6 mg, 94% yield); R_f 0.6 (hexane/ethyl acetate = 9/1); The regioisomeric ratio *m*:*o* = 97:3; **^{1}H NMR** (400 MHz, $CDCl_3$, major regioisomer) δ 7.97 (d, *J* = 8.0 Hz, 1H), 7.60−7.57 (m, 3H), 7.55 (m, 1H), 7.46 (t, *J* = 7.6 Hz, 1H), 7.42−7.34 (m, 2H), 7.27 (d, *J* = 7.6 Hz, 1H), 7.04 (td, *J* = 7.5, 1.9 Hz, 1H), 6.98 (app d, *J* = 8.8 Hz, 2H), 3.85 (s, 3H); **^{13}C{^{1}H} NMR** (151 MHz, $CDCl_3$) δ 159.3, 146.7, 144.6, 140.4, 139.7, 133.5, 130.2, 128.9, 128.5, 128.3, 128.2, 127.9, 127.6, 126.0, 114.3, 98.7, 55.4; **HRMS** (EI$^+$) Calcd for $C_{19}H_{15}IO^+$ [M]$^+$ 386.0168, found 386.0166.

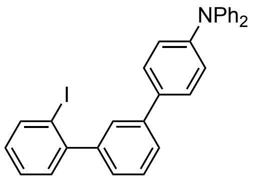

2''-Iodo-*N*,*N*-diphenyl-[1,1':3',1''-terphenyl]-4-amine (3ag): White oil (40.9 mg, 78% yield on 0.1 mmol scale; 391.1 mg, 75% yield on 1 mmol scale); R_f 0.5 (hexane/ethyl acetate = 9/1); **^{1}H NMR** (400 MHz, $CDCl_3$) δ 7.94 (dd, *J* = 7.9, 0.9 Hz, 1H), 7.58−7.54 (m, 2H), 7.50 (td, *J* = 8.6, 2.3 Hz, 2H), 7.43 (t, *J* = 7.7 Hz, 1H), 7.37−7.31 (m, 2H), 7.26−7.20 (m, 5H), 7.13−7.09 (m, 6H), 7.02−6.97 (m, 3H); **^{13}C{^{1}H} NMR** (151 MHz, $CDCl_3$) δ 147.7, 147.4, 146.7, 144.6, 140.3, 139.6, 134.8, 130.2, 129.4, 128.9, 128.5, 128.2, 127.9, 127.8, 127.7, 125.9, 124.5, 123.9, 123.0, 98.7; **HRMS** (EI$^+$) Calcd for $C_{30}H_{22}IN^+$ [M]$^+$ 523.0797, found 523.0800.

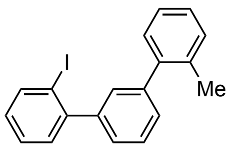

2-Iodo-2''-methyl-1,1':3',1''-terphenyl (3ah): Colorless oil (33.6 mg, 91% yield); R_f 0.5 (hexane/CH_2Cl_2 = 7/1); The regioisomeric ratio *m*:*o* = 97:3; **^{1}H NMR** (600 MHz, $CDCl_3$, major regioisomer) δ 7.96 (dd, *J* = 8.0, 1.1 Hz, 1H), 7.47 (t, *J* = 7.8 Hz, 1H), 7.39 (td, *J* = 7.4, 1.2 Hz, 1H), 7.35 (dt, *J* = 7.8, 2.0 Hz, 2H), 7.31−7.24 (m, 6H), 7.03 (td, *J* = 7.6, 1.8 Hz, 1H), 2.34 (s, 3H); **^{13}C{^{1}H} NMR** (151 MHz, $CDCl_3$) δ 146.6, 144.1, 141.7, 139.6, 135.6, 130.4, 130.3, 130.2, 129.9, 128.9, 128.5, 128.2, 127.8, 127.6, 127.4, 125.8, 98.7, 20.8; one aromatic resonance not resolved due to overlap; **HRMS** (EI$^+$) Calcd for $C_{19}H_{15}I^+$ [M]$^+$ 370.0218, found 370.0214.

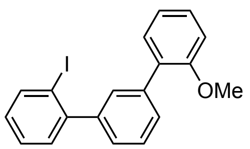

2-Iodo-2''-methoxy-1,1':3',1''-terphenyl (3ai): Colorless oil (36.4 mg, 94% yield); R_f 0.6 (hexane/ethyl acetate = 9/1); **^{1}H NMR** (400 MHz, $CDCl_3$) δ 7.97 (d, J = 7.8 Hz, 1H), 7.58 (dt, J = 7.8, 1.5 Hz, 1H), 7.53 (t, J = 1.6 Hz, 1H), 7.46 (t, J = 7.7 Hz, 1H), 7.40 (dt, J = 7.4, 1.8 Hz, 2H), 7.37−7.37 (m, 1H), 7.33−7.28 (m, 2H), 7.06−7.01 (m, 2H), 6.99 (d, J = 8.4 Hz, 1H), 3.83 (s, 3H); **^{13}C{^{1}H} NMR** (151 MHz, $CDCl_3$) δ 156.6, 146.8, 143.8, 139.6, 138.2, 131.1, 130.7, 130.4, 130.3, 128.9, 128.82, 128.79, 128.2, 127.8, 127.7, 121.0, 111.4, 98.8, 55.7; **HRMS** (EI^+) Calcd for $C_{19}H_{15}IO^+$ $[M]^+$ 386.0168, found 386.0163.

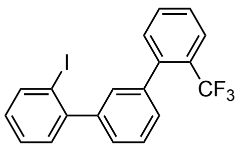

2-Iodo-2''-(trifluoromethyl)-1,1':3',1''-terphenyl (3aj): Colorless oil (31.9 mg, 76% yield); R_f 0.5 (hexane/CH_2Cl_2 = 8/1); **^{1}H NMR** (600 MHz, $CDCl_3$) δ 7.95 (dd, J = 7.9, 0.7 Hz, 1H), 7.75 (d, J = 7.9 Hz, 1H), 7.56 (t, J = 7.5 Hz, 1H), 7.48–7.44 (m, 2H), 7.41 (d, J = 7.6 Hz, 1H), 7.39–7.34 (m, 4H), 7.30 (s, 1H), 7.03 (td, J = 7.6, 1.7 Hz, 1H); **^{13}C{^{1}H} NMR** (151 MHz, $CDCl_3$) δ 146.4, 143.8, 141.2, 139.6, 132.2, 131.4, 130.2, 130.0, 129.0, 128.61 (q, J = 30.1 Hz), 128.57, 128.27, 128.26, 128.2, 127.5, 127.4, 126.2 (q, J = 5.3 Hz), 124.3 (q, J = 274.1 Hz), 98.6; **^{19}F NMR** (376 MHz, $CDCl_3$): δ −56.7; **HRMS** (EI^+) Calcd for $C_{19}H_{12}F_3I^+$ $[M]^+$ 423.9936, found 423.9938.

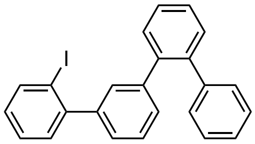

2'''-Iodo-1,1':2',1'':3'',1'''-quaterphenyl (3ak): Pale yellow oil (31.2 mg, 73% yield); R_f 0.2 (hexane/CH_2Cl_2 = 8/1); **^{1}H NMR** (400 MHz, $CDCl_3$) δ 7.90 (dd, J = 7.9, 1.0 Hz, 1H), 7.51−7.48 (m, 1H), 7.45−7.41 (m, 3H), 7.31 (td, J = 7.5, 1.1 Hz, 1H), 7.28−7.19 (m, 6H), 7.18−7.15 (m, 2H), 7.12 (t, J = 1.8 Hz, 1H), 7.03 (dd, J = 7.6, 1.7 Hz, 1H), 6.98 (td, J = 7.7, 1.7 Hz, 1H); **^{13}C{^{1}H} NMR** (151 MHz, $CDCl_3$) δ 146.6, 143.8, 141.6, 141.3, 140.8, 140.4, 139.4, 131.1, 130.6, 130.2, 130.1, 129.1, 128.7, 128.14, 128.05, 127.7, 127.55, 127.49, 126.5, 98.6; two aromatic resonances not resolved due to overlap; **HRMS** (EI^+) Calcd for $C_{24}H_{17}I^+$ $[M]^+$ 432.0375, found 432.0384.

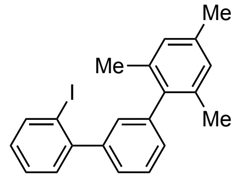

2''-Iodo-2,4,6-trimethyl-1,1':3',1''-terphenyl (3al): Pale yellow oil (32.8 mg, 73% yield); R_f 0.5 (hexane/CH_2Cl_2 = 9/1); The regioisomeric ratio *m*:*o* = 93:7; **^{1}H NMR** (600 MHz, $CDCl_3$, major regioisomer) δ 7.94 (dd, *J* = 7.9, 1.0 Hz, 1H), 7.46 (t, *J* = 7.7 Hz, 1H), 7.38 (dd, *J* = 7.1, 1.1 Hz, 1H), 7.35 (dd, *J* = 7.6, 1.9 Hz, 1H), 7.27 (dt, *J* = 7.8, 1.5 Hz, 1H), 7.16 (dt, *J* = 7.8, 1.4 Hz, 1H), 7.13 (t, *J* = 1.6 Hz, 1H), 7.02 (app td, *J* = 7.4, 1.9 Hz, 1H), 6.94 (s, 2H), 2.33 (s, 3H) , 2.07 (s, 6H); **^{13}C{^{1}H} NMR** (151 MHz, $CDCl_3$) δ 146.8, 144.5, 140.9, 139.6, 138.8, 136.7, 136.1, 130.3, 130.1, 128.8, 128.6, 128.2, 128.15, 128.10, 127.3, 98.8, 21.11, 21.09; **HRMS** (EI^+) Calcd for $C_{21}H_{19}I^+$ $[M]^+$ 398.0531, found 398.0536.

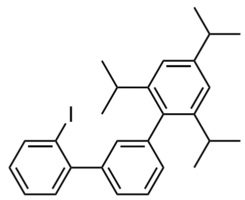

2''-Iodo-2,4,6-triisopropyl-1,1':3',1''-terphenyl (3am): White solid (40.7 mg, 85% yield on 0.1 mmol scale/411.4 mg, 85% yield on 1 mmol scale); R_f 0.3 (hexane/CH_2Cl_2 = 9/1); m.p. 111.1−113.2 °C; **^{1}H NMR** (400 MHz, $CDCl_3$) δ 7.94 (dd, *J* = 8.0, 0.9 Hz, 1H), 7.44 (t, *J* = 7.6 Hz, 1H), 7.39 (td, *J* = 7.4, 1.2 Hz, 1H), 7.35 (dd, *J* = 7.6, 1.9 Hz, 1H), 7.27 (dt, *J* = 7.9, 1.5 Hz, 1H), 7.20 (dt, *J* = 7.6, 1.4 Hz, 1H), 7.18 (t, *J* = 1.6 Hz, 1H), 7.06 (s, 2H), 7.02 (td, *J* = 7.6, 1.9 Hz, 1H), 2.94 (sept, *J* = 6.9 Hz, 1H), 2.75 (sept, *J* = 6.9 Hz, 2H), 1.31 (d, *J* = 6.9 Hz, 6H), 1.11 (d, *J* = 6.7 Hz, 6H), 1.10 (d, *J* = 7.0 Hz, 6H); **^{13}C{^{1}H} NMR** (151 MHz, $CDCl_3$) δ 148.0, 146.8, 146.7, 144.1, 140.6, 139.5, 136.8, 130.8, 130.1, 129.1, 128.8, 128.1, 127.7, 127.2, 120.6, 98.7, 34.4, 30.4, 24.4, 24.2; **HRMS** (EI^+) Calcd for $C_{27}H_{31}I^+$ $[M]^+$ 482.1470, found 482.1484.

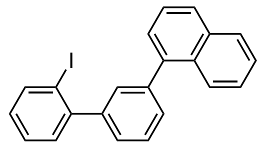

1-(2'-Iodo-[1,1'-biphenyl]-3-yl)naphthalene (3an): Colorless oil (36.1 mg, 89% yield); R_f 0.3 (hexane/CH_2Cl_2 = 9/1); The regioisomeric ratio *m*:*o* = 93:7; **^{1}H NMR** (400 MHz, $CDCl_3$, major regioisomer) δ 8.04 (d, *J* = 8.4 Hz, 1H), 7.97 (d, *J* = 7.9 Hz, 1H), 7.90 (d, *J* = 7.8 Hz, 1H), 7.86 (d, *J* = 8.1 Hz, 1H), 7.56−7.48 (m, 6H), 7.45 (td, *J* = 7.6, 1.4 Hz, 1H),7.41−7.39 (m, 3H), 7.05−7.01 (m, 1H); **^{13}C{^{1}H} NMR** (151 MHz, $CDCl_3$) δ 146.5, 144.3, 140.5, 140.0, 139.6,

133.9, 131.7, 131.1, 130.2, 129.4, 129.0, 128.3, 128.2, 128.11, 128.05, 127.8, 127.1, 126.3, 126.2, 125.9, 125.5, 98.8; **HRMS** (EI^+) Calcd for $C_{22}H_{15}I^+$ $[M]^+$ 406.0218, found 406.0220.

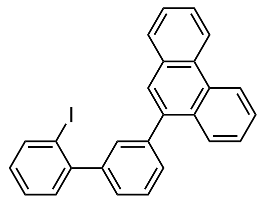

9-(2'-Iodo-[1,1'-biphenyl]-3-yl)phenanthrene (3ao): Colorless oil (36.9 mg, 81% yield); R_f 0.5 (hexane/CH_2Cl_2 = 8/1); The regioisomeric ratio *m*:*o* = 96:4; **^{1}H NMR** (600 MHz, $CDCl_3$, major regioisomer) δ 8.77 (d, *J* = 8.3 Hz, 1H), 8.71 (d, *J* = 8.3 Hz, 1H), 8.06 (dd, *J* = 8.2, 0.8 Hz, 1H), 7.96 (app d, *J* = 8.6 Hz, 1H), 7.89 (app d, *J* = 7.8 Hz, 1H), 7.75 (s, 1H), 7.68−7.64 (m, 2H), 7.62−7.53 (m, 5H), 7.43−7.37 (m, 3H), 7.02 (ddd, *J* = 8.0, 6.6, 2.5 Hz, 1H); **^{13}C{^{1}H} NMR** (151 MHz, $CDCl_3$) δ 146.5, 144.3, 140.5, 139.6, 138.5, 131.6, 131.12, 131.11, 130.7, 130.2, 130.1, 129.4, 129.0, 128.8, 128.2, 128.1, 127.7, 127.2, 126.9, 126.7, 126.64, 126.57, 123.0, 122.6, 98.8; one aromatic resonance not resolved due to overlap; **HRMS** (EI^+) Calcd for $C_{26}H_{17}I^+$ $[M]^+$ 456.0375, found 456.0356.

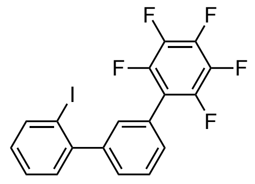

2,3,4,5,6-Pentafluoro-2''-iodo-1,1':3',1''-terphenyl (3ap)* : Colorless oil (25.9 mg, 58% yield); R_f 0.5 (hexane/CH_2Cl_2 = 8/1); The regioisomeric ratio *m*:*o* = 75:25; **^{1}H NMR** (600 MHz, $CDCl_3$, major regioisomer) δ 7.97 (dd, *J* = 8.0, 1.1 Hz, 1H), 7.56 (t, *J* = 7.8 Hz, 1H), 7.46−7.43 (m, 2H), 7.43−7.39 (m, 2H), 7.34 (dd, *J* = 7.6, 1.7 Hz, 1H), 7.06 (td, *J* = 7.7, 1.6 Hz, 1H); **^{13}C{^{1}H} NMR** (151 MHz, $CDCl_3$, major regioisomer) δ 145.7, 144.7, 139.8, 131.3, 131.23, 131.17, 130.3, 130.2, 129.4, 129.3, 128.5, 128.3, 128.2, 127.7, 126.2, 98.4; **^{19}F NMR** (376 MHz, $CDCl_3$, major regioisomer): δ −142.6 (dd, *J* = 22.9, 8.1 Hz), −155.2 (t, *J* = 21.0 Hz), −142.6 (dt, *J* = 22.0, 7.7 Hz); **HRMS** (EI^+) Calcd for $C_{18}H_8F_5I^+$ $[M]^+$ 445.9591, found 445.9595.
*__NOTE__: The Grignard reagent was prepared according to the conditions shown below. A flame-dried 10 mL Schlenk tube equipped with a magnetic stir bar was purged with argon, after which anhydrous THF (1.0 mL) and aryl bromide (247.2 mg, 1.0 mmol, 1.0 equiv) were added. A solution of *i*-PrMgCl•LiCl in THF (1.3 M, 808 μL, 1.05 equiv) was then added dropwise at room temperature, and the mixture was stirred for 30 min. The concentration of the resulting Grignard reagent was approximately 0.35 M.

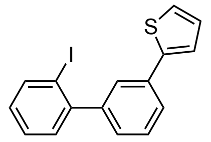

2-(2'-Iodo-[1,1'-biphenyl]-3-yl)thiophene (3aq): White oil (31.3 mg, 87% yield on 0.1 mmol scale/308.2 mg, 85% yield on 1 mmol scale); R_f 0.4 (hexane/CH_2Cl_2 = 9/1); **^{1}H NMR** (600 MHz, $CDCl_3$) δ 7.97 (dd, *J* = 7.9, 1.0 Hz, 1H), 7.63 (ddd, *J* = 7.8, 1.9, 1.1 Hz, 1H), 7.60 (t, *J* = 1.7 Hz, 1H), 7.43 (t, *J* = 7.8 Hz, 1H), 7.40 (dd, *J* = 7.6, 1.2 Hz, 1H), 7.36−7.34 (m, 2H), 7.28 (dd, *J* = 5.1, 1.1 Hz, 1H), 7.26−7.24 (m, 1H), 7.08 (dd, *J* = 5.3, 3.6 Hz, 1H), 7.05 (td, *J* = 7.7, 1.7 Hz, 1H); **^{13}C{^{1}H} NMR** (151 MHz, $CDCl_3$) 146.2, 144.8, 144.2, 139.7, 134.2, 130.1, 129.1, 128.6, 128.4, 128.3, 128.1, 127.0, 125.2, 125.1, 123.4, 98.6; **HRMS** (EI^+) Calcd for $C_{16}H_{11}IS^+$ $[M]^+$ 361.9626, found 361.9623.

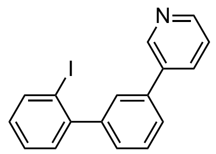

3-(2'-Iodo-[1,1'-biphenyl]-3-yl)pyridine (3ar): White oil (6.2 mg, 18% yield); R_f 0.2 (hexane/ethyl acetate = 4/1); The regioisomeric ratio *m*:*o* = 95:5; **^{1}H NMR** (400 MHz, $CDCl_3$, major regioisomer) δ 8.92 (d, *J* = 2.1 Hz, 1H), 8.61 (dd, *J* = 4.5, 1.7 Hz, 1H), 7.99 (dd, *J* = 7.9, 1.2 Hz, 1H), 7.94 (ddd, *J* = 7.8, 2.3, 1.7 Hz, 1H), 7.66−7.61 (m, 1H), 7.59 (t, *J* = 1.5 Hz, 1H), 7.55 (t, *J* = 7.7 Hz, 1H), 7.45−7.36 (m, 4H), 7.07 (ddd, *J* = 7.9, 7.3, 1.9 Hz, 1H); **^{13}C{^{1}H} NMR** (151 MHz, $CDCl_3$) δ 148.7, 148.5, 146.1, 145.0, 139.8, 137.6, 134.5, 130.2, 129.2, 129.1, 128.9, 128.38, 128.36, 127.3, 126.4, 123.7, 98.6; **HRMS** (EI^+) Calcd for $C_{17}H_{12}IN^+$ $[M]^+$ 357.0014, found 357.0013.

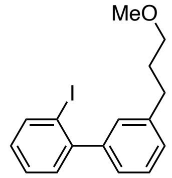

2-Iodo-3'-(3-methoxypropyl)-1,1'-biphenyl (3as): Colorless oil (18.2 mg, 26% yield on 0.2 mmol scale); R_f 0.4 (hexane/ethyl acetate = 9/1); **^{1}H NMR** (600 MHz, $CDCl_3$) δ 7.93 (dd, *J* = 8.1, 1.0 Hz, 1H), 7.38 (td, *J* = 7.4, 1.2 Hz, 1H), 7.35 (td, *J* = 7.5, 1.3 Hz, 1H), 7.31 (app d, *J* = 7.6 Hz, 1H), 7.26 (s, 1H), 7.24 (dd, *J* = 7.5, 1.6 Hz, 1H), 7.06 (dd, *J* = 7.8, 1.4 Hz, 1H), 7.04 (td, *J* = 7.7, 1.6 Hz, 1H), 3.24 (t, *J* = 6.5 Hz, 2H), 3.23 (s, 3H), 2.52 (ddd, *J* = 14.1, 9.5, 6.0 Hz, 1H), 2.39 (ddd, *J* = 14.1, 9.3, 6.4 Hz, 1H), 1.78−1.65 (m, 2H); **^{13}C{^{1}H} NMR** (151 MHz, $CDCl_3$) δ 146.4, 144.2, 139.4, 139.0, 130.1, 129.9, 129.2, 128.8, 128.2, 127.9, 125.7, 100.6, 72.2, 58.4, 30.3, 29.7; **HRMS** (EI^+) Calcd for $C_{16}H_{17}IO^+$ $[M]^+$ 352.0324, found 352.0330.

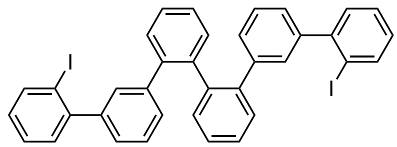

2,2'''''-Diiodo-1,1':3',1'':2'',1''':2''',1'''':3'''',1'''''-sexiphenyl (3at)*: White solid (43.5 mg, 12% yield); R_f 0.2 (hexane/CH_2Cl_2 = 5/1); m.p. 197.8 °C (decomp.) °C; **^{1}H NMR** (400 MHz, $CDCl_3$) δ 7.86 (d, J = 7.6 Hz, 2H), 7.42−7.41 (m, 2H), 7.37−7.30 (m, 6H), 7.27 (t, J = 7.5 Hz, 2H), 7.10−7.05 (m, 4H), 6.95 (td, J = 7.6, 1.4 Hz, 2H), 7.49 (d, J = 7.5 Hz, 2H), 6.71 (s, 2H), 6.70 (d, J = 7.4 Hz, 2H); **^{13}C{^{1}H} NMR** (151 MHz, $CDCl_3$) δ 146.4, 142.9, 140.8, 140.7, 140.2, 139.5, 131.9, 130.5, 130.3, 128.64, 128.58, 128.0, 127.6, 127.5, 127.3, 127.2, 98.4; one aromatic resonance not resolved due to overlap; **HRMS** (EI^+) Calcd for $C_{36}H_{24}I_2^+$ $[M]^+$ 709.9967, found 709.9957.

*__NOTE__: The Grignard reagent was prepared by stirring 2,2′-dibromobiphenyl (156.2 mg, 0.5 mmol, 1.0 equiv) with magnesium turnings (4.0 equiv) in anhydrous THF (1.0 mL) at 65 °C for 2 h, affording a white suspension. Separately, a flame-dried 100 mL Schlenk tube equipped with a magnetic stir bar was charged with a solution of iodonium salt **1a** (471.0 mg, 1.1 mmol, 2.2 equiv) in anhydrous 1,4-dioxane (20 mL) and heated in an oil bath at 70 °C. The entire suspension of the freshly prepared Grignard reagent was added to the solution of **1a** via syringe, and the resulting mixture was stirred at 70 °C for 12 h. The yield of **3at** was calculated based on the initial amount of 2,2′-dibromobiphenyl.

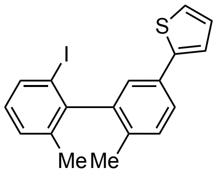

2-(2'-Iodo-6,6'-dimethyl-[1,1'-biphenyl]-3-yl)thiophene (3bq): Colorless oil (22.8 mg, 66% yield); R_f 0.4 (hexane/CH_2Cl_2 = 6/1); The regioisomeric ratio m:o = 85:15; **^{1}H NMR** (600 MHz, $CDCl_3$, major regioisomer) δ 7.80 (d, J = 8.1 Hz, 1H), 7.56 (dd, J = 7.9, 1.9 Hz, 1H), 7.31−7.29 (m, 2H), 7.27−7.24 (m, 3H), 7.06 (dd, J = 5.0, 3.6 Hz, 1H), 6.97 (t, J = 7.8 Hz, 1H), 2.07 (s, 3H), 2.00 (s, 3H); **^{13}C{^{1}H} NMR** (151 MHz, $CDCl_3$) δ 145.4, 144.44, 144.40, 137.8, 136.7, 135.0, 132.4, 130.8, 129.9, 129.1, 128.0, 126.2, 125.3, 124.5, 122.8, 101.0, 21.9, 19.3; **HRMS** (EI^+) Calcd for $C_{18}H_{15}IS^+$ $[M]^+$ 389.9939, found 389.9940.

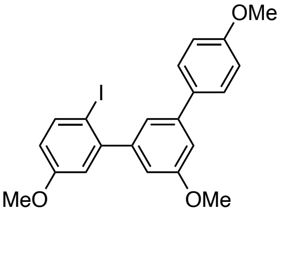

2-Iodo-4'',5,5'-trimethoxy-1,1':3',1''-terphenyl (3cf): Yellow oil (35.7 mg, 80% yield); R_f 0.2 (hexane/ethyl acetate = 8/1); The regioisomeric ratio *m*:*o* = 90:10; **^{1}H NMR** (400 MHz, $CDCl_3$, major regioisomer) δ 7.81 (d, J = 8.8 Hz, 1H), 7.58 (app d, J = 9.0 Hz, 2H), 7.13 (t, J = 1.4 Hz, 1H), 7.11 (t, J = 2.0 Hz, 1H), 6.97 (app d, J = 8.8 Hz, 2H), 6.94 (d, J = 2.9 Hz, 1H), 6.82 (dd, J = 2.3, 1.6 Hz 1H), 6.66 (dd, J = 8.7, 3.0 Hz, 1H), 3.89 (s, 3H), 3.84 (s, 3H), 3.79 (s, 3H); **^{13}C{^{1}H} NMR** (151 MHz, $CDCl_3$) δ 159.8, 159.5, 159.4, 147.4, 145.6, 141.9, 140.1, 133.4, 128.3, 120.5, 115.9, 115.4, 114.3, 113.0, 111.9, 87.0, 55.52, 55.51, 55.4; **HRMS** (EI^+) Calcd for $C_{21}H_{19}IO_3^+$ $[M]^+$ 446.0379, found 446.0372.

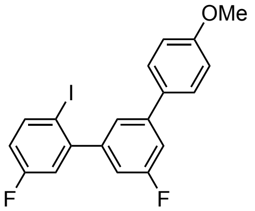

5,5'-Difluoro-2-iodo-4''-methoxy-1,1':3',1''-terphenyl (3df): Yellow oil (28.9 mg, 69% yield); R_f 0.4 (hexane/ethyl acetate = 9/1); The regioisomeric ratio *m*:*o* = 86:14; **^{1}H NMR** (600 MHz, $CDCl_3$, major regioisomer) δ 7.90 (dd, J = 8.7, 5.6 Hz, 1H), 7.56 (app d, J = 9.0 Hz, 2H), 7.30−7.28 (m, 2H), 7.09 (dd, J = 9.1, 3.1 Hz, 1H), 6.99 (app d, J = 8.8 Hz, 2H), 6.97−6.95 (m, 1H), 7.84 (dt, J = 8.3, 3.1 Hz, 1H), 3.86 (s, 3H); **^{13}C{^{1}H} NMR** (151 MHz, $CDCl_3$) δ 162.9 (d, J = 248.9 Hz), 162.8 (d, J = 246.4 Hz), 159.8, 147.3 (d, J = 7.2 Hz), 145.2 (d, J = 8.3 Hz), 142.9 (d, J = 8.3 Hz), 140.9 (d, J = 8.0 Hz), 128.3, 123.4 (d, J = 2.5 Hz), 117.4 (d, J = 22.4 Hz), 116.7 (d, J = 21.7 Hz), 114.5, 114.3 (d, J = 22.4 Hz), 113.4, 113.1 (d, J = 22.0 Hz), 91.2 (d, J = 3.3 Hz), 55.5; **^{19}F NMR** (376 MHz, $CDCl_3$): δ −112.9 (t, J = 5.4 Hz), −114.0 (ddd, J = 9.0, 8.1, 5.7 Hz); **HRMS** (EI^+) Calcd for $C_{19}H_{13}F_2IO^+$ $[M]^+$ 421.9979, found 421.9997.

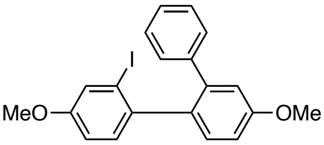

2-Iodo-4,4'-dimethoxy-1,1':2',1''-terphenyl (3eb)*: Colorless oil (26.5 mg, 64% yield); R_f 0.5 (hexane/ethyl acetate = 7/1); The regioisomeric ratio *m*:*o* = 16:84; **^{1}H NMR** (600 MHz, $CDCl_3$, major regioisomer) δ 7.33 (d, J = 2.6 Hz, 1H), 7.20−7.16 (m, 6H), 6.96−6.93 (m, 2H), 6.91 (d, J = 8.4 Hz, 1H), 6.71 (dd, J = 8.5, 2.7 Hz, 1H), 3.88 (s, 3H), 3.74 (s, 3H); **^{13}C{^{1}H} NMR** (151 MHz, $CDCl_3$) δ 159.2, 158.4, 142.4, 141.2, 138.4, 135.2, 132.5, 131.7, 129.6, 127.8, 126.7, 123.7, 115.3, 113.9, 112.5, 101.2, 55.5, 55.4; **HRMS** (EI^+) Calcd for $C_{20}H_{17}IO_2^+$ $[M]^+$ 416.0273, found 416.0270.

*__NOTE__: The *ortho*-connectivity of the major regioisomer was confirmed by a Suzuki–Miyaura coupling of the regioisomeric mixture with phenylboronic acid, which furnished a symmetric

(and thus spectroscopically simplified) tetraaryl product expected for the *ortho* isomer as the major component (vide infra).

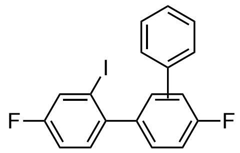

4,4'-Difluoro-2-iodo-1,1':3',1''-terphenyl (3fb)*: Colorless oil (17.6 mg, 45% yield); R_f 0.6 (hexane/CH_2Cl_2 = 8/1); The regioisomeric ratio *m*:*o* = 50:50; **^{1}H NMR** (600 MHz, $CDCl_3$) δ 7.68 (dd, *J* = 8.2, 2.7 Hz, 1H), 7.62−7.60 (m, 2H), 7.53 (dd, *J* = 8.1, 2.6 Hz, 1H), 7.46 (app t, *J* = 7.6 Hz, 2H), 7.41 (dd, *J* = 7.4, 2.4 Hz, 1H), 7.40−7.37 (m, 1H), 7.30 (dd, *J* = 8.5, 5.9 Hz, 1H), 7.26 (s, 1H), 7.25−7.19 (m, 5H), 7.15 (dd, *J* = 9.6, 2.6 Hz, 1H), 7.14−7.09 (m, 4H), 6.99 (dd, *J* = 8.5, 5.9 Hz, 1H), 6.91 (td, *J* = 8.4, 2.6 Hz, 1H); **^{13}C{^{1}H} NMR** (151 MHz, $CDCl_3$) δ 162.5 (d, *J* = 247.8 Hz), 161.4 (d, *J* = 252.2 Hz), 160.9 (d, *J* = 251.4 Hz), 159.4 (d, *J* = 249.3 Hz), 143.2 (d, *J* = 8.3 Hz), 141.8 (d, *J* = 3.3 Hz), 141.5 (d, *J* = 3.6 Hz), 139.8 (d, *J* = 1.4 Hz), 139.5 (d, *J* = 4.0 Hz), 137.8 (d, *J* = 3.3 Hz), 135.5 (d, *J* = 1.4 Hz), 132.7 (d, *J* = 8.3 Hz), 132.1 (d, *J* = 3.6 Hz), 131.9 (d, *J* = 8.0 Hz), 130.7 (d, *J* = 8.0 Hz), 129.9 (d, *J* = 8.3 Hz), 129.5, 129.2 (d, *J* = 3.3 Hz), 128.7 (d, *J* = 14.1 Hz), 128.6, 128.01, 127.95, 127.3, 126.5 (d, *J* = 23.5 Hz), 125.9 (d, *J* = 23.5 Hz), 116.9 (d, *J* = 22.0 Hz), 115.9 (d, *J* = 23.1 Hz), 115.4 (d, *J* = 21.0 Hz), 115.0 (d, *J* = 21.0 Hz), 114.0 (d, *J* = 21.3 Hz), 100.0 (d, *J* = 7.6 Hz), 98.2 (d, *J* = 7.6 Hz); **^{19}F NMR** (376 MHz, $CDCl_3$): δ −113.6 (m), −113.7 (m), −113.8 (m), −119.2 (m); **HRMS** (EI^+) Calcd for $C_{18}H_{11}F_2I^+$ $[M]^+$ 391.9874, found 391.9868.

*__NOTE__: Due to indistinguishable NMR signals, assignment of individual regioisomers was not possible. Therefore, all representative peaks in the ^{1}H, ^{13}C, and ^{19}F NMR spectra are listed above.

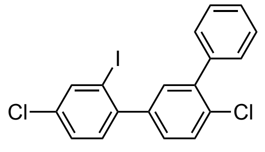

4,4'-Dichloro-2-iodo-1,1':3',1''-terphenyl (3gb)*: Colorless oil (28.9 mg, 68% yield on 0.1 mmol scale/214.6 mg, 51% yield on 1 mmol scale); R_f 0.4 (hexane/CH_2Cl_2 = 8/1); The regioisomeric ratio *m*:*o* = 70:30; **^{1}H NMR** (600 MHz, $CDCl_3$, major regioisomer) δ 7.96 (d, *J* = 2.1 Hz, 1H), 7.53−7.50 (m, 3H), 7.46−7.44 (m, 2H), 7.41−7.39 (m, 1H), 7.38 (dd, *J* = 5.8, 2.3 Hz, 1H), 7.32 (d, *J* = 2.2 Hz, 1H), 7.25−7.21 (m, 2H); **^{13}C{^{1}H} NMR** (151 MHz, $CDCl_3$, major regioisomer) δ 143.8, 141.7, 140.2, 139.0, 138.4, 132.3, 130.5, 129.8, 129.6, 129.5, 129.3, 128.6, 128.2, 128.1, 127.9, 98.4; **HRMS** (EI^+) Calcd for $C_{18}H_{11}Cl_2I^+$ $[M]^+$ 423.9283, found 423.9278.

*NOTE: The *ortho*-connectivity of the major regioisomer was confirmed by a Suzuki–Miyaura coupling of the regioisomeric mixture with phenylboronic acid, which furnished a symmetric (and thus spectroscopically simplified) tetraaryl product expected for the *ortho* isomer as the major component (vide infra).

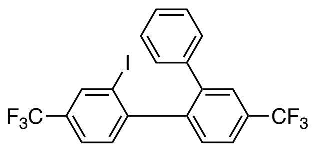

2-Iodo-4,4'-bis(trifluoromethyl)-1,1':2',1''-terphenyl (3hb)*: Colorless oil (43.4 mg, 88% yield on 0.1 mmol scale/371.3 mg, 75% yield on 1 mmol scale); R_f 0.6 (hexane/CH_2Cl_2 = 6/1); The regioisomeric ratio *m*:*o* = 16:84; **^{1}H NMR** (400 MHz, $CDCl_3$, major regioisomer) δ 8.08 (s, 1H), 7.73 (s, 1H), 7.69 (app d, *J* = 8.0 Hz, 1H), 7.47 (app d, *J* = 8.0 Hz, 1H), 7.42−7.40 (m, 1H), 7.24−7.22 (m, 3H), 7.15−7.13 (m, 3H); **^{13}C{^{1}H} NMR** (151 MHz, $CDCl_3$) δ 148.9, 144.9, 141.5, 139.0, 136.1 (q, *J* = 3.9 Hz), 131.2, 131.10, 131.06 (q, *J* = 32.5 Hz), 131.0 (q, *J* = 32.4 Hz), 129.5, 128.3, 127.7, 127.4 (q, *J* = 3.7 Hz), 124.7 (q, *J* = 3.6 Hz), 124.0 (q, *J* = 272.4 Hz), 123.9 (q, *J* = 3.6 Hz), 122.8 (q, *J* = 272.9 Hz), 99.3; **^{19}F NMR** (376 MHz, $CDCl_3$): δ −62.4, −62.6; **HRMS** (EI$^+$) Calcd for $C_{20}H_{11}F_6I^+$ [M]$^+$ 491.9810, found 491.9818.
*NOTE: The *ortho*-connectivity of the major regioisomer was confirmed by a Suzuki–Miyaura coupling of the regioisomeric mixture with phenylboronic acid, which furnished a symmetric (and thus spectroscopically simplified) tetraaryl product expected for the *ortho* isomer as the major component (vide infra).

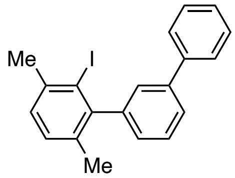

2-Iodo-3,6-dimethyl-1,1':3',1''-terphenyl (3ib): Colorless oil (25.1 mg, 65% yield); R_f 0.3 (hexane/CH_2Cl_2 = 8/1); The regioisomeric ratio *m*:*o* = 81:19; **^{1}H NMR** (600 MHz, $CDCl_3$, major regioisomer) δ 7.66−7.61 (m, 3H), 7.52 (t, *J* = 7.7 Hz 1H), 7.46−7.41 (m, 2H), 7.36 (t, *J* = 1.7 Hz, 1H), 7.21−7.12 (m, 3H), 7.08 (d, *J* = 7.6 Hz, 1H), 2.50 (s, 3H), 2.09 (s, 3H); **^{13}C{^{1}H} NMR** (151 MHz, $CDCl_3$) δ 147.0, 146.1, 141.2, 141.1, 139.7, 134.5, 129.5, 128.8, 128.7, 128.04, 128.02, 127.4, 127.2, 126.0, 125.9, 108.1, 29.8, 22.1; **HRMS** (EI$^+$) Calcd for $C_{20}H_{17}I^+$ [M]$^+$ 384.0375, found 384.0368.

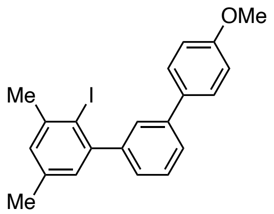

2-Iodo-4''-methoxy-3,5-dimethyl-1,1':3',1''-terphenyl (3jf): Colorless oil (32.3 mg, 78% yield); R_f 0.2 (hexane/CH_2Cl_2 = 4/1); The regioisomeric ratio *m*:*o* = 88:12; **^{1}H NMR** (400 MHz, $CDCl_3$, major regioisomer) δ 7.58 (app d, *J* = 8.8 Hz, 2H), 7.56 (ddd, *J* = 7.8, 1.8, 1.3 Hz, 1H), 7.50 (t, *J* = 1.6 Hz, 1H), 7.45 (t, *J* = 7.7 Hz, 1H), 7.23 (dt, *J* = 7.8, 1.4 Hz, 1H), 7.07 (d, *J* = 2.1 Hz, 1H), 6.98−6.96 (m, 3H), 3.84 (s, 3H), 2.52 (s, 3H), 2.30 (s, 3H); **^{13}C{^{1}H} NMR** (151 MHz, $CDCl_3$) δ 159.3, 147.6, 146.0, 142.3, 140.3, 137.5, 133.6, 130.7, 129.6, 128.3, 128.0, 127.6, 125.7, 114.3, 113.2, 102.0, 55.4, 30.0, 20.7; **HRMS** (EI^+) Calcd for $C_{21}H_{19}IO^+$ $[M]^+$ 414.0481, found 414.0472.

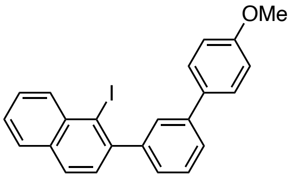

1-Iodo-2-(4'-methoxy-[1,1'-biphenyl]-3-yl)naphthalene (3kf): Colorless oil (22.7 mg, 52% yield); R_f 0.2 (hexane/CH_2Cl_2 = 4/1); The regioisomeric ratio *m*:*o* = 89:11; **^{1}H NMR** (400 MHz, $CDCl_3$, major regioisomer) δ 8.35 (d, *J* = 8.6 Hz, 1H), 7.85 (d, *J* = 8.3 Hz, 1H), 7.83 (d, *J* = 8.1 Hz, 1H), 7.62−7.60 (m, 5H), 7.55−7.53 (m, 1H), 7.51 (t, *J* = 7.6 Hz, 1H), 7.46 (d, *J* = 8.3 Hz, 1H), 7.32 (dt, *J* = 7.8, 1.4 Hz, 1H), 6.98 (app d, *J* = 8.8 Hz, 2H), 3.85 (s, 3H); **^{13}C{^{1}H} NMR** (151 MHz, $CDCl_3$) δ 159.4, 146.5, 146.3, 140.4, 135.1, 133.6, 133.5, 133.1, 128.6, 128.5, 128.34, 128.31, 128.21, 128.20, 127.8, 127.7, 126.7, 125.9 114.3, 104.2, 55.4; **HRMS** (EI^+) Calcd for $C_{23}H_{17}IO^+$ $[M]^+$ 436.0324, found 436.0310.

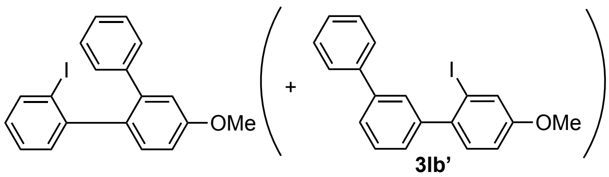

3lb'

2-Iodo-4'-methoxy-1,1':2',1''-terphenyl (3lb): White oil (29.0 mg, 75% yield); R_f 0.3 (hexane/ethyl acetate = 9/1); The regioisomeric ratio *meta*:*ortho*:**3lb'** = 14:82:4; **^{1}H NMR** (600 MHz, $CDCl_3$, major regioisomer) δ 7.98 (dd, *J* = 7.9, 1.2 Hz 1H), 7.20 (d, *J* = 8.4 Hz, 1H), 7.18−7.16 (m, 5H), 7.14 (dd, *J* = 7.4, 1.2 Hz, 1H), 7.03 (dd, *J* = 7.6, 1.7 Hz, 1H), 6.98−6.95 (m, 2H), 6.87 (td, *J* = 7.7, 1.7 Hz, 1H), 3.89 (s, 3H); **^{13}C{^{1}H} NMR** (151 MHz, $CDCl_3$) δ 159.3,

146.1, 142.1, 141.0, 139.0, 135.6, 132.0, 131.6, 129.6, 128.3, 127.8, 127.6, 126.8, 115.3, 112.5, 101.4, 55.4; **HRMS** (EI^+) Calcd for $C_{20}H_{17}I^+$ $[M]^+$ 384.0375, found 384.0368.
The regiochemistry of the major isomer was established as follows. The 4-methoxy-substituted iodonium salt could in principle generate two distinct arynes—one arising within the methoxy-substituted ring and the other within the unsubstituted phenyl ring. The former, under the directing influence of the methoxy group, is expected to undergo *ortho*-arylation to afford *o*-**3lb**, whereas the latter would behave analogously to other 3-arylbenzynes, leading to *meta*-arylation (**3lb'**). Notably, only the former product contains an *ortho*-disubstituted benzene ring. The presence of a diagnostic td signal (δ 6.87), characteristic of *ortho*-disubstitution, identifies the major isomer as *o*-**3lb**. The ratio of *o*-**3lb** to the minor regioisomers (*m*-**3lb** and **3lb'**) was estimated from the minor ^{1}H NMR signals, in analogy with the imperfect regioselectivity observed for **3eb** (*m*:*o* = 16:84).

Assignment of the regiochemistry of 3eb, 3gb, and 3hb: To unambiguously determine the regiochemistry of the major products arising from highly electronically perturbed 3,6-disubstituted arynes, we performed their Suzuki–Miyaura coupling with phenylboronic acid, expecting that ^{1}H and ^{13}C NMR patterns of the coupling products would be markedly different depending on the regiochemistry of the starting iodoteraryls. Thus, only when the *ortho*-isomer was the major product, should the spectral patterns be apparently simplified due to the molecular symmetry of the *ortho*-tetraarylene product.

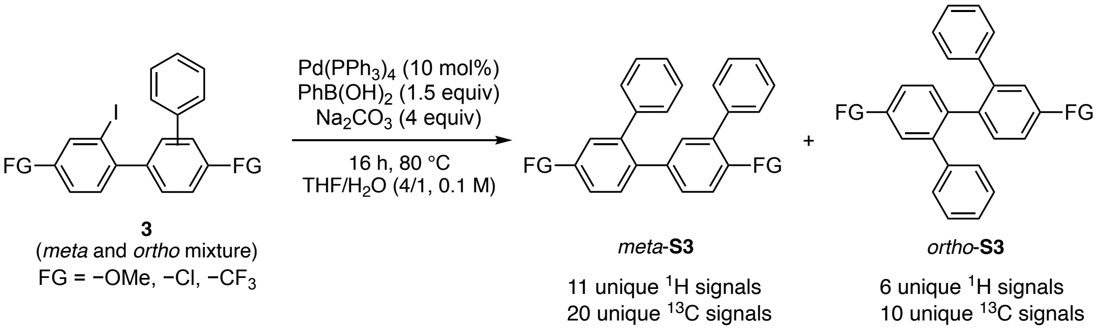

Procedure for the diagnostic Suzuki–Miyaura reaction: A flame-dried 10 mL Schlenk tube equipped with a magnetic stir bar was charged with **3** (mixture of regioisomers, 1.0 equiv), phenylboronic acid (1.5 equiv), tetrakis(triphenylphosphine)palladium (10 mol%), and sodium carbonate (4.0 equiv). After the tube was purged with argon, anhydrous THF and digassed water (THF/H_2O = 4/1, 0.1 M) were added. The tube was then placed in a preheated aluminum block (80 °C) and stirred for 16 h. After cooling to room temperature, water (5 mL) was added, and the mixture was extracted with EtOAc (5 mL × 3). The combined organic layers were dried over $MgSO_4$, filtered, and concentrated under reduced pressure. The residue was purified by preparative thin-layer chromatography followed by gel permeation chromatography to afford tetraalylenes *meta*-**S3** and *ortho*-**S3**. ^{1}H and ^{13}C NMR analyses of the mixture indicated the dominance of symmetric *ortho*-**S3**.

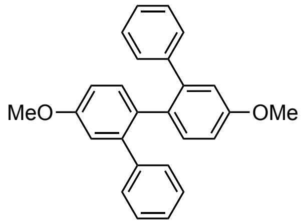

4'',5'-dimethoxy-1,1':2',1'':2'',1'''-quaterphenyl (S3eb): Compound **S3eb** was prepared from **3eb** (22.0 mg, 0.05 mmol); White oil (19.4 mg, quantative yield); R_f 0.5 (hexane/ethyl acetate = 7/1); The regioisomeric ratio *meta*-**S3eb**:*ortho*-**S3eb** = 16:84; **^{1}H NMR** (400 MHz, $CDCl_3$, major regioisomer) δ 7.28−7.26 (m, 2H), 7.09−7.06 (m, 2H), 7.03−6.98 (m, 4H), 6.88 (dd, J = 8.4, 2.6 Hz 2H), 6.70 (d, J = 2.8 Hz, 2H), 6.64−6.61 (m, 4H), 3.81 (s, 6H); **^{13}C{^{1}H} NMR** (151 MHz, $CDCl_3$) δ 142.3, 141.2, 132.9, 132.3, 129.2, 127.6, 126.0, 115.2, 112.7, 55.4; **HRMS** (EI^+) Calcd for $C_{26}H_{22}O_2^+$ $[M]^+$ 366.1620, found 366.1618.

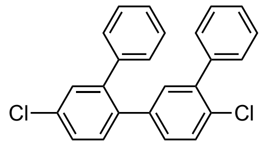

4'',5'-Dichloro-1,1':2',1'':3'',1'''-quaterphenyl (S3gb): Compound **S3gb** was prepared from **3gb** (37.3 mg, 0.09 mmol); Colorless oil (23.6 mg, 72% yield); R_f 0.5 (hexane/CH_2Cl_2 = 7/1); The regioisomeric ratio *meta*-**S3gb**:*ortho*-**S3gb** = 64:36; **^{1}H NMR** (400 MHz, $CDCl_3$, both regioisomers) δ 7.43−7.42 (m, 1H), 7.383−7.379 (m, 2H), 7.37−7.27 (m, 9.2H), 7.16−7.10 (m, 6.4H), 7.07 (dd, J = 8.3, 2.3 Hz 1H), 7.05−7.01 (m, 3H), 6.57 (app dd, J = 8.4, 1.3 Hz, 2.2H); **^{13}C{^{1}H} NMR** (151 MHz, $CDCl_3$, both regioisomers) δ 142.7, 142.3, 140.2, 140.0, 139.4, 139.1, 139.0, 137.6, 137.4, 133.7, 133.6, 133.1, 132.9, 131.6, 131.2, 130.6, 130.1, 129.84, 129.78, 129.6, 129.4, 129.1, 128.4, 128.0, 127.85, 127.75, 127.6, 127.28, 127.27, 126.7; Due to the moderate *m*:*o* ratio, ^{1}H and ^{13}C NMR signals could not be individually assigned to each regioisomer; **HRMS** (EI^+) Calcd for $C_{24}H_{16}Cl_2^+$ $[M]^+$ 374.0629, found 374.0622.

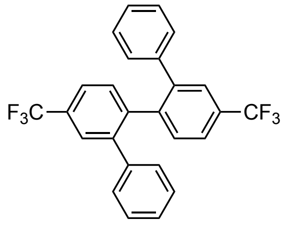

4'',5'-Bis(trifluoromethyl)-1,1':2',1'':2'',1'''-quaterphenyl (S3hb): Compound **S3hb** was prepared from **3hb** (86.7 mg, 0.2 mmol); White solid (62.1 mg, 80% yield); m.p. = 167.4−168.2 °C; R_f 0.5 (hexane/CH_2Cl_2 = 7/1); The regioisomeric ratio *meta*-**S3hb**:*ortho*-**S3hb** = 84:16; **^{1}H NMR** (400 MHz, $CDCl_3$) δ 7.62 (dd, J = 8.1, 1.3 Hz 2H), 7.51 (d, J = 8.0 Hz 2H),

7.44 (s, 2H), 7.15 (td, *J* = 7.3, 1.4 Hz 2H), 7.04 (app t, *J* = 7.8 Hz 4H), 6.59−6.57 (m, 4H); **^{13}C{^{1}H} NMR** (151 MHz, $CDCl_3$) δ 142.4, 141.8, 139.1, 132.0, 130.4 (q, *J* = 32.4 Hz), 129.1, 128.0, 127.12 (q, *J* = 3.6 Hz), 127.07, 124.1 (q, *J* = 272.3 Hz), 124.0 (q, *J* = 3.7 Hz); **HRMS** (EI^+) Calcd for $C_{26}H_{16}F_6^+$ $[M]^+$ 442.1156, found 442.1162.

3. Product Transformations

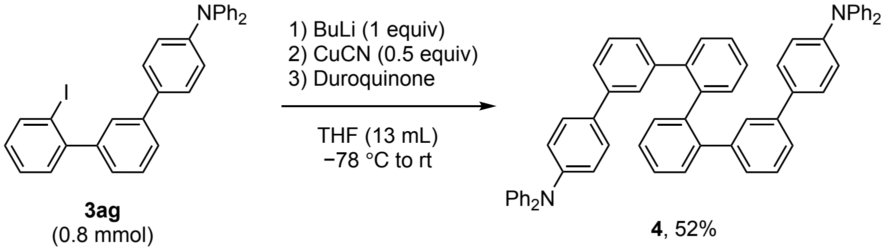

Oxidative homocoupling of 3ag: A flame-dried two-necked 30 mL round-bottom flask equipped with a magnetic stir bar was charged with **3ag** (391.1 mg, 0.8 mmol, 1.0 equiv). The flask was purged with argon, and anhydrous THF (13 mL) was added. The mixture was cooled to −78 °C in a dry ice–acetone bath, and a solution of BuLi (1.54 M in hexane × 485 μL, 0.75 mmol, 1.0 equiv) was added dropwise via syringe. After stirring at −78 °C for 30 min, CuCN (35.0 mg, 0.4 mmol, 0.52 equiv) was added in one portion. The reaction mixture was warmed to room temperature, and duroquinone (186.2 mg, 1.1 mmol, 1.52 equiv) was added in one portion. The mixture was stirred for 1.5 h, quenched with 10% aqueous NH_3, and extracted with EtOAc (15 mL × 3). The combined organic layers were dried over $MgSO_4$, filtered, and concentrated under reduced pressure. The residue was purified by silica gel column chromatography followed by gel permeation chromatography to afford the desired product **4**.

***N*^{4},*N*^{4},*N*$^{4'''''}$,*N*$^{4'''''}$-Tetraphenyl-[1,1':3',1'':2'',1''':2''',1'''':3'''',1'''''-sexiphenyl]-4,4'''''-diamine (4):** White solid (153.7 mg, 52% yield); R_f 0.5−0.6 (hexane/ethyl acetate = 6/1); m.p. 113.5 °C (decomp.); **^{1}H NMR** (600 MHz, $CDCl_3$) δ 7.52 (dd, J = 7.6, 1.2 Hz 2H), 7.41 (td, J = 7.5, 1.2 Hz 2H), 7.34 (td, J = 7.5, 1.2 Hz 2H), 7.24−7.21 (m, 12H), 7.08 (app d, J = 7.6 Hz, 8H), 7.04−6.96 (m, 14H), 6.84 (t, J = 1.7 Hz, 2H), 6.51 (d, J = 7.8 Hz, 2H); **^{13}C{^{1}H} NMR** (151 MHz, $CDCl_3$) δ 147.8, 146.9, 141.1, 140.9, 140.4, 139.6, 135.2, 131.8, 130.0, 129.3, 128.1, 127.9, 127.8, 127.7, 127.5, 124.4, 124.3, 123.9, 122.9; one aromatic resonance not resolved due to overlap; **HRMS** (EI^+) Calcd for $C_{60}H_{44}N_2^+$ $[M]^+$ 792.3504, found 792.3532.

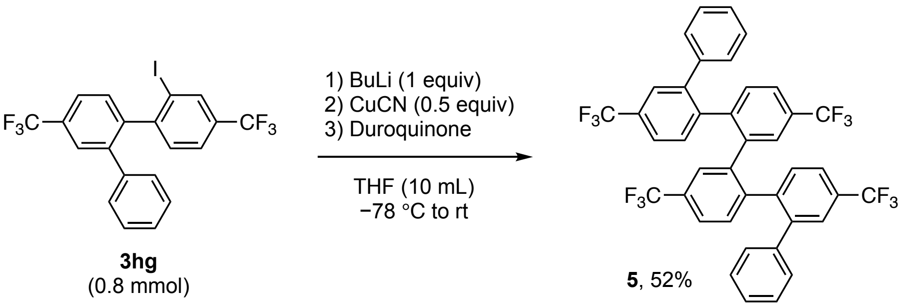

Oxidative homocoupling of 3hb: A flame-dried two-necked 30 mL round-bottom flask equipped with a magnetic stir bar was charged with **3hb** (221.2 mg, 0.45 mmol, 1.0 equiv). The flask was purged with argon, and anhydrous THF (10 mL) was added. The mixture was cooled to −78 °C in a dry ice–acetone bath, and a solution of BuLi (1.53 M in hexane × 294 μL, 0.45

mmol, 1.0 equiv) was added dropwise via syringe. After stirring at −78 °C for 30 min, CuCN (21.5 mg, 0.24 mmol, 0.52 equiv) was added in one portion. The reaction mixture was warmed to room temperature, and duroquinone (112.1 mg, 0.68 mmol, 1.52 equiv) was added in one portion. The mixture was stirred for 1.5 h, quenched with 10% aqueous NH_3, and extracted with EtOAc (15 mL × 3). The combined organic layers were dried over $MgSO_4$, filtered, and concentrated under reduced pressure. The residue was purified by preparative thin-layer chromatography to afford the desired product **5**.

4'',4'''',5',5'''-tetrakis(trifluoromethyl)-1,1':2',1'':2'',1''':2''',1'''':2'''',1'''''-sexiphenyl (5): White solid (93.3 mg, 57% yield); R_f 0.3 (hexane/CH_2Cl_2 = 7/1); m.p. 211.1−212.1 °C; **^{1}H NMR** (600 MHz, $CDCl_3$) δ 7.55 (d, J = 8.1 Hz, 2H), 7.32−7.31 (m, 4H), 7.21 (d, J = 7.9 Hz, 2H), 7.11 (t, J = 7.2 Hz, 2H), 6.96 (t, J = 7.3 Hz, 4H), 6.43 (d, J = 7.9 Hz, 2H), 6.36 (d, J = 8.1 Hz, 4H), 6.05 (s, 2H); **^{19}F NMR** (376 MHz, $CDCl_3$): δ −61.9 (s), −62.6 (s); **^{13}C{^{1}H} NMR** (151 MHz, $CDCl_3$) δ 141.8, 141.7, 140.2, 139.3, 138.9, 131.9, 131.4, 130.9 (q, J = 32.8 Hz), 129.9 (q, J = 32.8 Hz), 128.44, 128.41, 127.5, 126.8 (q, J = 3.8 Hz), 124.9 (q, J = 3.3 Hz), 123.6 (q, J = 272.5 Hz), 121.8 (q, J = 273.1 Hz); **HRMS** (EI^+) Calcd for $C_{40}H_{22}F_{12}^+$ $[M]^+$ 730.1530, found 730.1523.

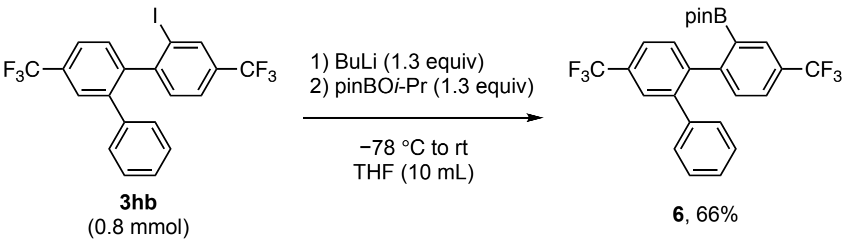

Borylation of 3ae: A flame-dried two-necked 50 mL round-bottom flask equipped with a magnetic stir bar was charged with **3hb** (412.6 mg, 0.8 mmol, 1.0 equiv). The flask was purged with argon, and anhydrous THF (10 mL) was added. The mixture was cooled to −78 °C in a dry ice–acetone bath, and a solution of BuLi (1.54 M in hexane, 708 µL, 1.1 mmol, 1.3 equiv) was added dropwise via syringe. After stirring at −78 °C for 1 h, 2-isopropoxy-4,4,5,5-tetramethyl-1,3,2-dioxaborolane (202.9 mg, 1.1 mmol, 1.3 equiv) was added dropwise via syringe. The reaction mixture was warmed to room temperature and stirred for 12 h. The reaction was then quenched with water and extracted with EtOAc (15 mL × 3). The combined organic layers were dried over $MgSO_4$, filtered, and concentrated under reduced pressure. The residue was purified by silica gel column chromatography to afford the desired product **6**.

2-(4,4'-bis(trifluoromethyl)-[1,1':2',1''-terphenyl]-2-yl)-4,4,5,5-tetramethyl-1,3,2-dioxaborolane (6): Yellow oil (271.9 mg, 66% yield); R_f 0.1 (hexane); **^{1}H NMR** (600 MHz, $CDCl_3$) δ 7.99 (s, 1H), 7.68 (s, 1H), 7.58 (d, J = 7.9 Hz, 1H), 7.45 (d, J = 7.9 Hz, 1H), 7.32 (d, J = 7.9 Hz, 1H), 7.20−7.15 (m, 5H), 7.04 (d, J = 7.9 Hz, 1H), 1,17−1.11 (m, 12H); **^{13}C{^{1}H} NMR** (151 MHz, $CDCl_3$) δ 150.2, 144.6, 141.5, 139.9, 131.6 (q, J = 3.6 Hz), 131.4, 130.7, 130.4, 130.1 (q, J = 32.6 Hz), 129.9, 128.7 (q, J = 32.4 Hz), 128.1, 127.3, 126.6 (q, J = 3.6 Hz), 126.5 (q, J = 3.6 Hz), 124.4 (q, J = 272.0 Hz), 124.3 (q, J = 272.3 Hz), 123.1 (q, J = 3.6 Hz), 84.2, 24.9; **^{19}F NMR** (376 MHz, $CDCl_3$): δ −62.4, −62.5; **HRMS** (EI^+) Calcd for $C_{26}H_{23}BF_6O_2^+$ $[M]^+$ 492.1695, found 492.1705.

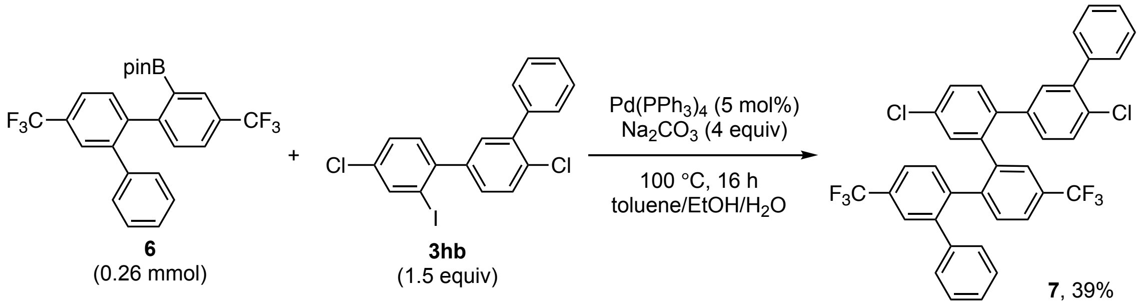

Cross-coupling of 3hb and 6: A flame-dried 10 mL Schlenk tube equipped with a magnetic stir bar was charged with **6** (125.9 mg, 0.3 mmol, 1.0 equiv), **3hb** (163.9 mg, 0.4 mmol, 1.5 equiv), tetrakis(triphenylphosphine)palladium (14.5 mg, 0.013 mmol, 5 mol%), and cesium carbonate (333.4 mg, 1.0 mmol, 4.0 equiv). The tube was purged with argon, and anhydrous toluene (0.5 mL), distilled ethanol (0.3 mL), and degassed water (0.2 mL) were added. The tube was then placed in a preheated oil bath (100 °C) and stirred for 16 h. After cooling to room temperature, water (5 mL) was added, and the mixture was extracted with EtOAc (5 mL × 3). The combined organic layers were dried over $MgSO_4$, filtered, and concentrated under reduced pressure. The residue was purified by silica gel column chromatography followed by gel permeation chromatography to afford the desired product **7**.

4'''',5'''-dichloro-4'',5'-bis(trifluoromethyl)-1,1':2',1'':2'',1''':2''',1'''':3'''',1'''''-sexiphenyl (7): White solid (95.6 mg, 39% yield); m.p. 93.9 °C (decomp.); **^{1}H NMR** (600 MHz, $CDCl_3$) δ 7.82 (d, J = 7.8 Hz, 1H), 7.50-7.49 (m, 2H), 7.35-7.32 (m, 4H), 7.26 (t, J = 7.3 Hz, 1H), 7.22 (d, J = 8.1 Hz, 1H), 7.20 (dd, J = 8.4, 2.2 Hz, 1H), 7.17−7.14 (m, 3H), 7.07-7.06 (m, 2H), 6.99 (d, J = 8.3 Hz, 1H), 6.55 (d, J = 7.4 Hz, 1H), 6.51 (d, J = 2.1 Hz, 1H), 7.36−7.33 (m, 2H), 5.97 (d, J = 2.1 Hz, 1H); **^{13}C{^{1}H} NMR** (151 MHz, $CDCl_3$) δ 143.4, 141.2, 140.9, 140.4, 139.7, 139.1, 138.7, 138.4, 138.1, 136.9, 133.8, 132.3, 132.2, 131.9, 131.2, 131.1, 131.0 (q, J = 33.1 Hz), 130.4, 130.11 (q, J = 32.5 Hz), 130.10, 129.2, 128.6, 128.5, 128.4, 128.3, 128.1, 127.9, 127.5, 126.5 (q, J = 3.6 Hz), 125.2 (q, J = 3.3 Hz), 124.0 (q, J = 272.4 Hz), 123.9 (q, J = 3.6 Hz); two aromatic resonances not resolved due to overlap; **^{19}F NMR** (376 MHz, $CDCl_3$): δ −62.2 (s), −62.5 (s); **HRMS** (EI^+) Calcd for $C_{38}H_{22}Cl_2F_6^+$ $[M]^+$ 662.1003, found 662.0985.

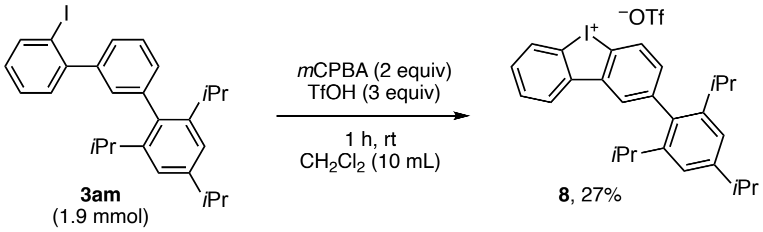

Synthesis of the arylated cyclicdiaryliodonium salt 8: A 100 mL round-bottom flask equipped with a magnetic stir bar was charged with **3am** (934.9 mg, 1.9 mmol, 1.0 equiv) and 3-chloroperoxybenzoic acid (669.4 mg, 3.9 mmol, 2.0 equiv). Dichloromethane (10 mL) was added to the flask. The mixture was cooled in an ice bath (0 °C) and stirred until the solid completely dissolved. Trifluoromethanesulfonic acid (510 μL, 5.8 mmol, 3.0 equiv) was then added dropwise via syringe at 0 °C. The reaction mixture was warmed to room temperature and stirred for 1 h. The solvent was evaporated under reduced pressure, and diethyl ether (20 mL) was added to the residue. The resulting suspension was kept at −20 °C for 5 h, and the precipitated solid was collected by filtration. The crude solid was washed with Et_2O (3 × 10 mL) and dried under vacuum to afford the desired product **8**.

2-(2,4,6-triisopropylphenyl)dibenzo[*b*,*d*]iodol-5-ium trifluoromethanesulfonate (8): Yellow solid (331.3 mg, 27% yield); m.p. 269.1−270.5 °C; **^{1}H NMR** (400 MHz, DMSO-d_6) δ 8.66 (d, J = 7.6 Hz, 1H), 8.45 (s, 1H), 8.28 (t, J = 8.9 Hz, 2H), 7.84 (t, J = 7.5 Hz, 1H), 7.75 (t, J = 7.7 Hz, 1H), 7.58 (d, J = 8.4 Hz, 1H), 7.18 (s, 2H), 2.98 (quin, J = 6.8 Hz, 1H), 2.51 (quin, J = 6.7 Hz, 2H), 1.30 (d, J = 6.8 Hz, 6H), 1.10 (d, J = 6.4 Hz, 12H); **^{13}C{^{1}H} NMR** (151 MHz, $CDCl_3$) δ 148.4, 145.8, 143.2, 141.8, 141.7, 134.9, 132.2, 131.2, 130.7, 130.5, 130.2, 127.8, 127.4, 122.0, 120.5, 119.9, 33.8, 30.1, 24.0, 23.9; **^{19}F NMR** (376 MHz, $CDCl_3$): δ −78.8 (s); **HRMS** (EI$^+$) Calcd for $C_{27}H_{30}I^+$ [M]$^+$ 481.1387, found 481.1385.

4. Mechanistic Experiments

^{19}F NMR studies (Figure 2a)

(a) ^{19}F NMR measurement of aryl Grignard reagent **2c**.

A solution of 4-fluorophenylmagnesium bromide (**2c**; 1.08 M in THF, 13 µL, 0.014 mmol) was added to anhydrous THF (0.5 mL) in an NMR tube. The resulting solution was subjected to ^{19}F NMR analysis (Figure S3).

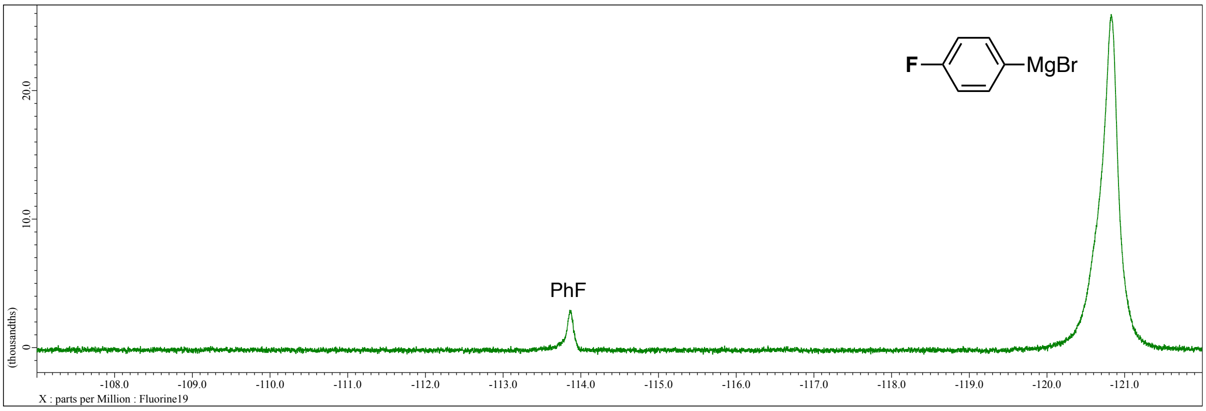

Figure S3. ^{19}F NMR spectrum (376 MHz, THF) of **2c**.

(b) ^{19}F NMR measurement of cyclic diaryliodonium salt **1d**.

Cyclic diaryliodonium salt **1d** (6.6 mg, 0.014 mmol) was dissolved in anhydrous THF (0.5 mL) in an NMR tube, and the resulting solution was subjected to ^{19}F NMR analysis (Figure S4). The observation of a single Ar–F resonance suggests that **1d** exists as a solvent-separated ion pair rather than a contact, T-shaped ion pair (which is common in the solid state).

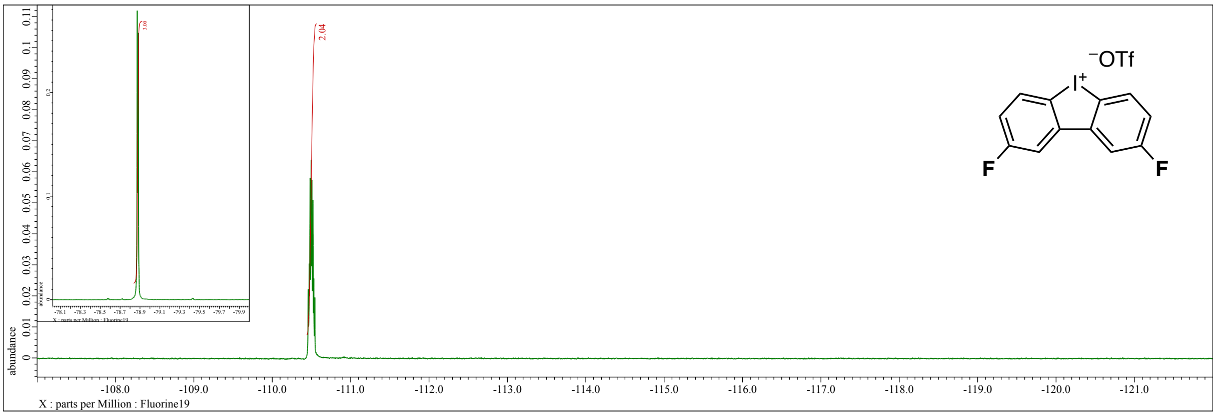

Figure S4. ^{19}F NMR spectrum (376 MHz, THF) of **1d**.

(c) ^{19}F NMR experiments with **1d** and **2c**.

To the NMR tube prepared in (b) was added a solution of Grignard reagent **2c** (1.08 M in THF, 13 µL, 0.014 mmol, 1.0 equiv). The mixture was allowed to stand at room temperature for 10 min and then analyzed by ^{19}F NMR spectroscopy (Figure S5). The emergence of three independent Ar–F resonances at –114.8, –115.0, and –116.8 ppm is consistent with the

formation of a T-shaped triaryliodane **A1**. Based on qualitative analysis of the ^{1}H–^{19}F spin-spin coupling patterns, the signal at –115.0 ppm can be assigned to the exocyclic 4-fluorophenyl group. Given that the 4-fluorophenyl ligand occupies the apical position of the T-shaped geometry, the signal proximal to this one (–114.8 ppm) likely originates from the other apical fluoroaryl moiety of the cyclic skeleton, whereas the distal signal (–116.8 ppm) is assignable to the equatorial fluoroaryl moiety.

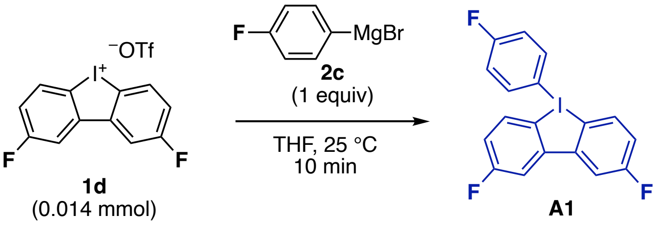

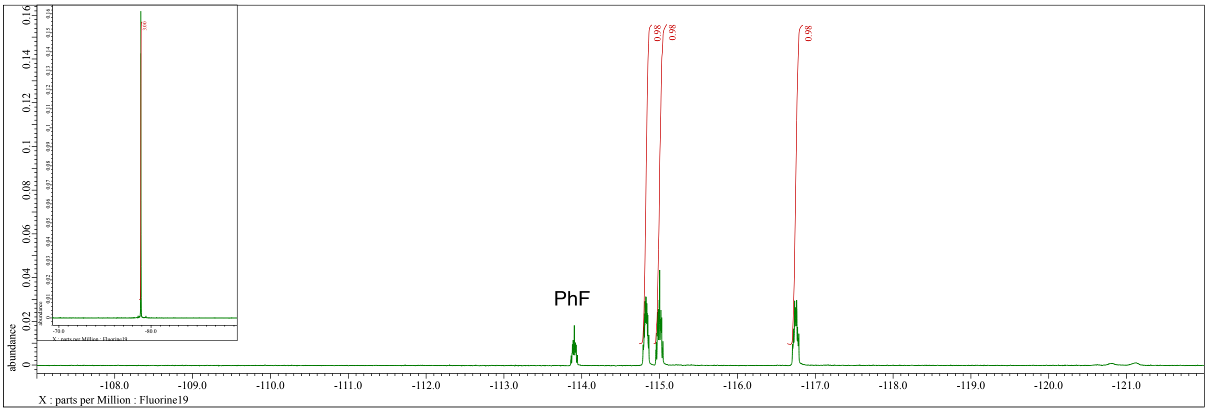

Figure S5. ^{19}F NMR spectrum (376 MHz, THF) of a mixture of **1d** and **2c**.

(d) ^{19}F NMR experiments with **1d**, **2c**, and **2f**.

To the NMR tube prepared in (c) was added a solution of 4-methoxyphenylmagnesium bromide (**2f**; 1.08 M in THF, 14 µL, 0.014 mmol, 1.0 equiv). The mixture was allowed to stand at room temperature for 10 min and then analyzed by ^{19}F NMR spectroscopy (Figure S6). The attenuation of the ^{19}F signals assigned to **A1**, the regeneration of the ^{19}F signal of 4-fluorophenyl Grignard, and the emergence of two new ^{19}F signals of equal intensity indicate the generation of a new triaryliodane **A2** via Grignard-mediated aryl ligand exchange on **A1**. As discussed for **A1**, the signals of **A2** at –115.2 and –117.2 ppm can be assigned to the apical and equatorial fluoroaryl moieties, respectively.

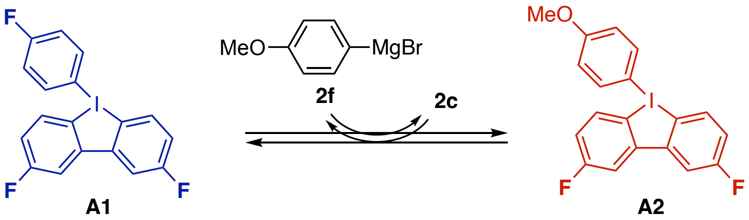

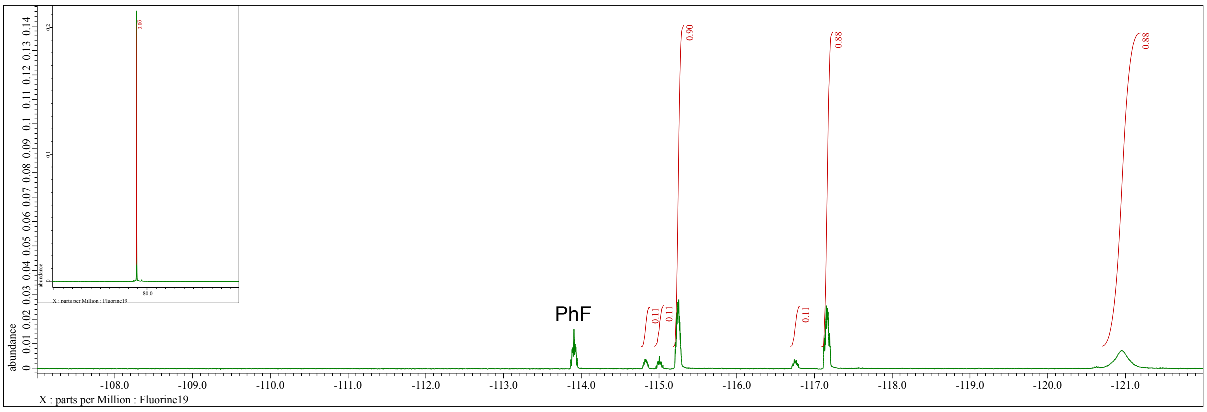

Figure S6. ^{19}F NMR spectrum (376 MHz, THF) of a mixture prepared by sequential addition of **2c** followed by **2f** to **1d**.

(e) ^{19}F NMR experiments with **1d**, **2c**, and **2f** at elevated temperature.

The NMR sample prepared in (d) was further subjected to variable-temperature ^{19}F NMR analysis at 60 °C (Figure S7), at which well-resolved signals were still observed, indicating that ligand exchange remains slow on the NMR timescale even at elevated temperature. Upon prolonged heating, decomposition via ring-opening arylation gradually complicated the spectral pattern.

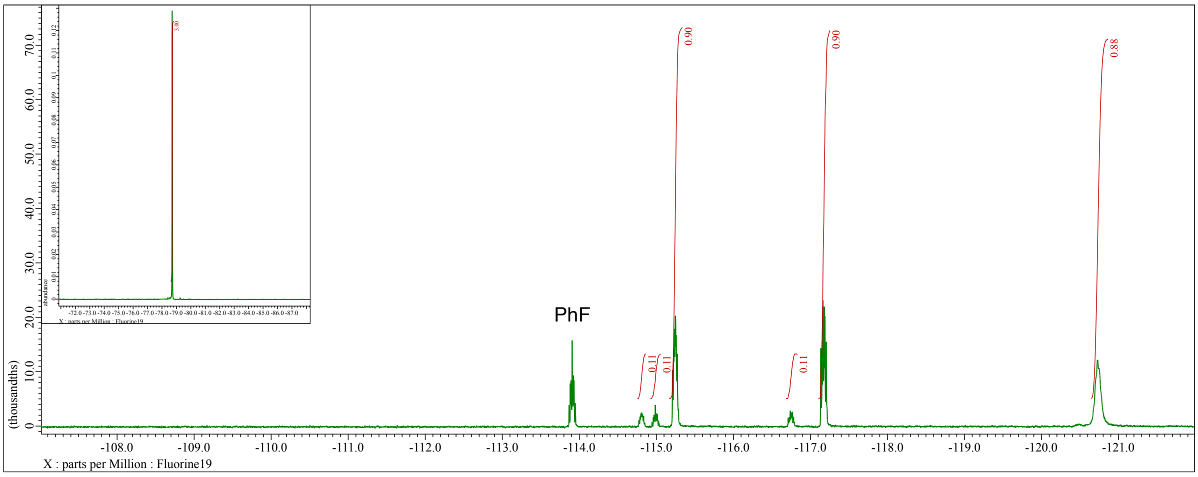

Figure S7. ^{19}F NMR spectrum (376 MHz, THF) of a mixture of **1d**, **2c**, and **2f** at 60 °C.

Electrophilic trapping of aryl Grignard reagent and cyclic diaryliodonium salt/Grignard mixture (Figure 2b)

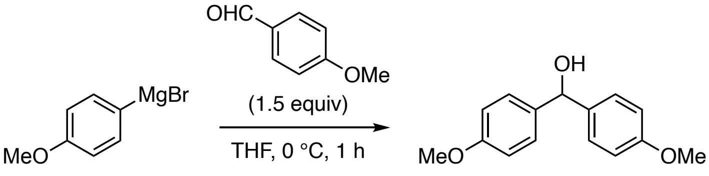

2f (0.1 mmol)   quant

A flame-dried 10 mL Schlenk tube equipped with a magnetic stir bar was purged with argon. Anhydrous THF (2 mL) and a solution of aryl Grignard reagent **2f** (0.99 M in THF, 101 μL, 0.10 mmol, 1.0 equiv) were added to the tube. The resulting solution was cooled to 0 °C, and 4-methoxybenzaldehyde (20.3 mg, 0.15 mmol, 1.5 equiv) was added dropwise via syringe under stirring. After stirring at 0 °C for 1 h, the reaction was quenched with saturated aqueous NH_4Cl. Water was then added, and the mixture was extracted with CH_2Cl_2 (3 × 5 mL). The combined organic layers were dried over anhydrous $MgSO_4$ and concentrated under reduced pressure. The ^1H NMR spectral data of the product were in good agreement with the reported values of bis(4-methoxyphenyl)methanol.[8] Quantitative formation of the product was confirmed by ^1H NMR analysis of the crude reaction mixture using 1,1,2,2-tetrachloroethane as an internal standard.

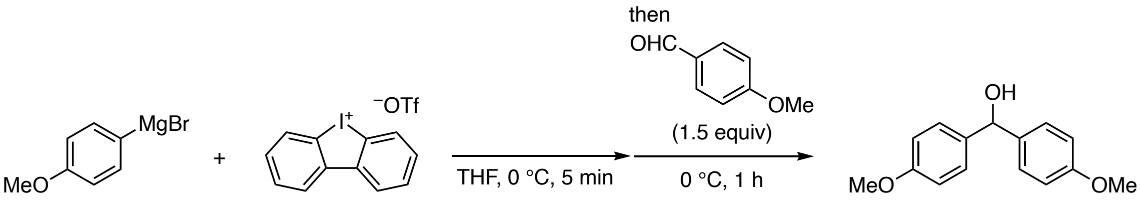

2f (0.1 mmol)   **2a** (1 equiv)   7%

A flame-dried 10 mL Schlenk tube equipped with a magnetic stir bar was charged with cyclic diaryliodonium salt **1a** (43.0 mg, 0.10 mmol, 1.0 equiv). The tube was purged with argon, and anhydrous THF (2 mL) was added. The tube was cooled to 0 °C, and a solution of aryl Grignard reagent **2f** (0.99 M in THF, 101 μL, 0.10 mmol, 1.0 equiv) was added dropwise via syringe under stirring. The mixture was stirred at 0 °C for 5 min, then 4-methoxybenzaldehyde (20.2 mg, 0.15 mmol, 1.5 equiv) was added dropwise via syringe. The reaction mixture was stirred at 0 °C for an additional 1 h and then quenched with saturated aqueous NH_4Cl. Water was added, and the mixture was extracted with CH_2Cl_2 (3 × 5 mL). The combined organic layers were dried over anhydrous $MgSO_4$ and concentrated under reduced pressure. The yield of the product was determined by ^1H NMR analysis of the crude mixture using 1,1,2,2-tetrachloroethane as an internal standard.

Ring-opening arylation using two distinct aryl Grignard reagents (Crossover experiments; Figure 2c)

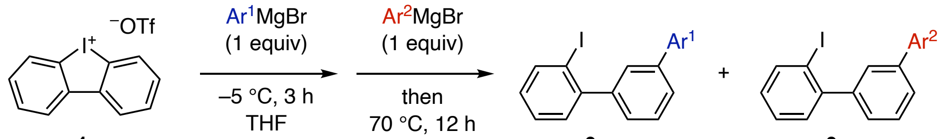

1a (0.1 mmol) 3ax 3ay

General procedure: A flame-dried 10 mL Schlenk tube equipped with a magnetic stir bar was charged with cyclic diaryliodonium salt **1a** (0.1 mmol, 1.0 equiv). The tube was purged with argon, and anhydrous THF (2 mL) was added. The tube was then cooled to −5 °C, and a solution of Ar^1MgBr (1.0 equiv) was added dropwise via syringe. After stirring at −5 °C for 3 h, a solution of Ar^2MgBr (1.0 equiv) was added dropwise via syringe. The tube was immediately transferred to a preheated aluminum block at 70 °C, and the reaction mixture was stirred at 70 °C for 12 h. After cooling to room temperature, saturated NH_4Cl aqueous solution was added, and the mixture was extracted with CH_2Cl_2 (3 × 5 mL). The combined organic layers were dried over anhydrous $MgSO_4$ and concentrated under reduced pressure. The yields of **3ax** and **3ay** was determined by ^1H NMR analysis of the crude mixture using 1,1,2,2-tetrachloroethane as an internal standard.

Table S3. Results of crossover experiments.

Ar[1]	Ar[2]	yield of **3ax**	yield of **3ay**	**3ax**:**3ay**
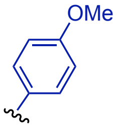	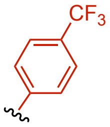	46%	22%	68:32
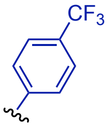	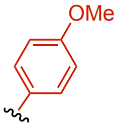	21%	47%	31:69
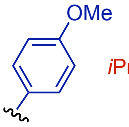	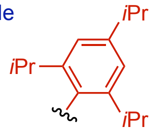	41%	43%	49:51
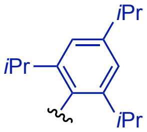	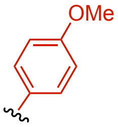	45%	42%	52:48
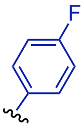	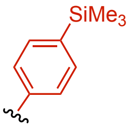	16%	53%	23:77
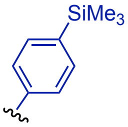	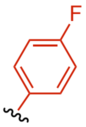	51%	11%	82:18
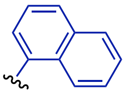	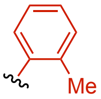	15%	66%	19:81
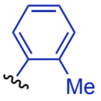	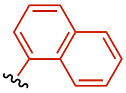	70%	16%	81:19

Trapping of aryne intermediate (Figure 2d)

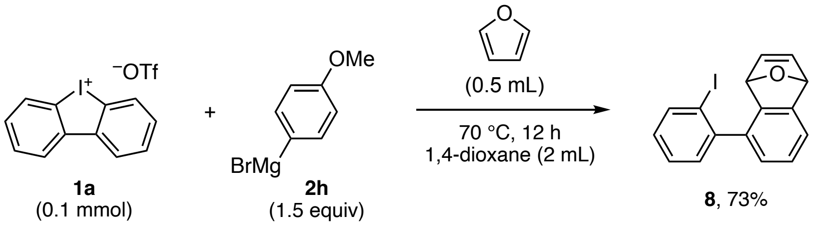

A flame-dried 10 mL Schlenk tube equipped with a magnetic stir bar was charged with cyclic diaryliodonium salt **1a** (43.3 mg, 0.1 mmol, 1.0 equiv). After purging the tube with argon, anhydrous 1,4-dioxane (2 mL) and furan (0.5 mL) were added. The tube was placed in a preheated aluminum block at 70 °C, and a solution of arylmagnesium reagent **2h** (0.58 M in THF, 256 μL, 1.5 equiv) was added dropwise via syringe. The reaction mixture was stirred at 70 °C for 12 h. After cooling to room temperature, saturated aqueous NH_4Cl was added, and the mixture was extracted with CH_2Cl_2 (3 × 5 mL). The combined organic layers were dried over anhydrous $MgSO_4$, concentrated under reduced pressure, and purified by preparative thin-layer chromatography (PTLC) to afford the desired product 5-(2-iodophenyl)-1,4-dihydro-1,4-epoxynaphthalene (**8**) as a yellow oil (25.7 mg, 73% yield). The spectral data were in good agreement with the literature values.[9]

Quenching with D_2O (Figure 2e)

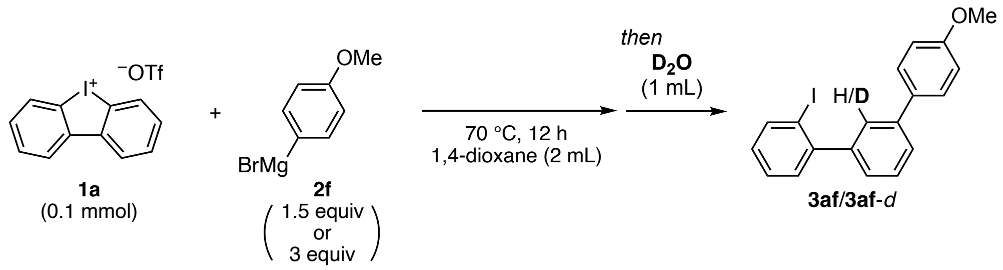

A flame-dried 10 mL Schlenk tube equipped with a magnetic stir bar was charged with cyclic diaryliodonium salt **1a** (0.1 mmol, 1.0 equiv). The tube was purged with argon, and anhydrous 1,4-dioxane (2 mL) was added. The tube was then placed in a preheated aluminum block at 70 °C, and a solution of aryl Grignard reagent **2f** (1.5 or 3.0 equiv) was added dropwise via syringe. The reaction mixture was stirred at 70 °C for 12 h. After cooling to room temperature, D_2O (1 mL) was added, and the mixture was stirred for 2 h. The reaction mixture was extracted with CH_2Cl_2 (3 × 5 mL). The combined organic layers were dried over anhydrous $MgSO_4$, concentrated under reduced pressure, and the residue was purified by preparative thin-layer chromatography to afford a mixture of the desired products **3af** and **3af**-*d*. The ratio of **3af**/**3af**-*d* was determined by ^{1}H NMR analysis after purification (Figure S8−S10).

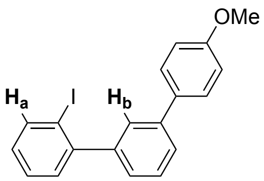

3af
(*meta*:*ortho* = 97:3)

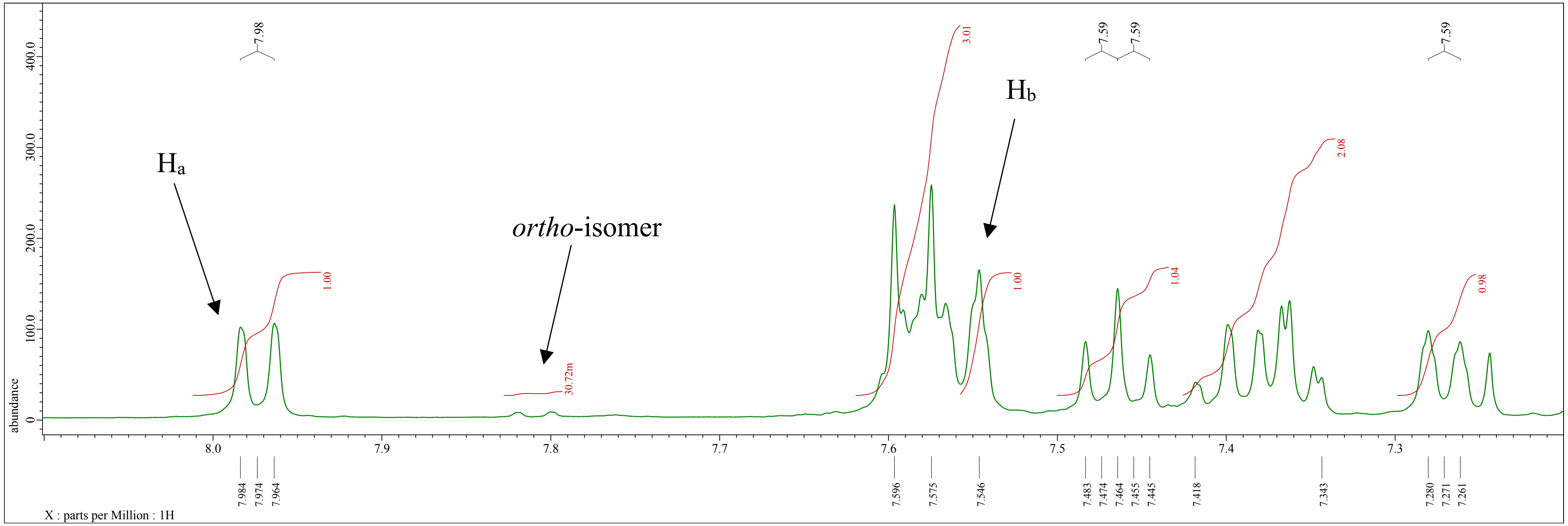

Figure S8. ^{1}H NMR spectra of **3af** (400 MHz, $CDCl_3$).

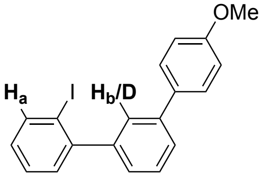

3af/3af-*d*
(H/D = 68/32)

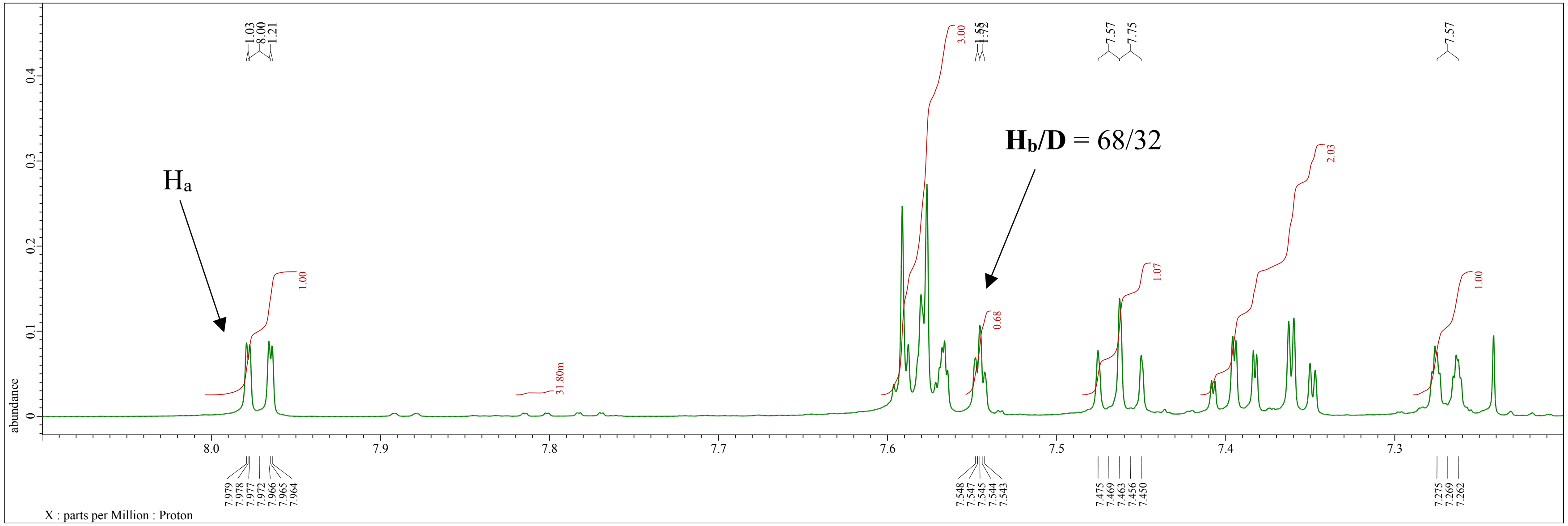

Figure S9. ^{1}H NMR analysis of mixture after purification using 1.5 equivalents of Grignard reagent (400 MHz, $CDCl_3$).

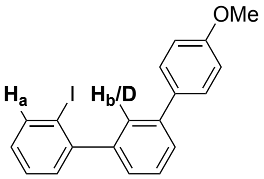

3af/**3af**-*d*
(H/D = 31/69)

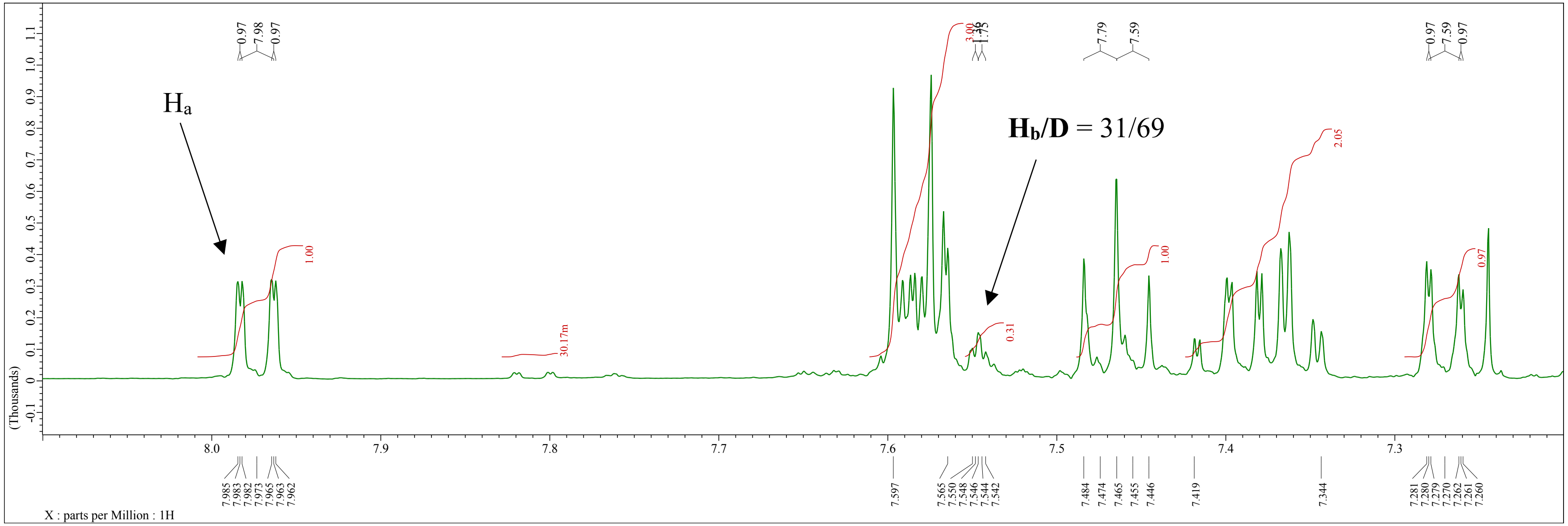

Figure S10. ^{1}H NMR analysis of mixture after purification using 3.0 equivalents of Grignard reagent (400 MHz, $CDCl_3$)

5. Electrophilic Trapping of Terarylmagnesium Intermediate

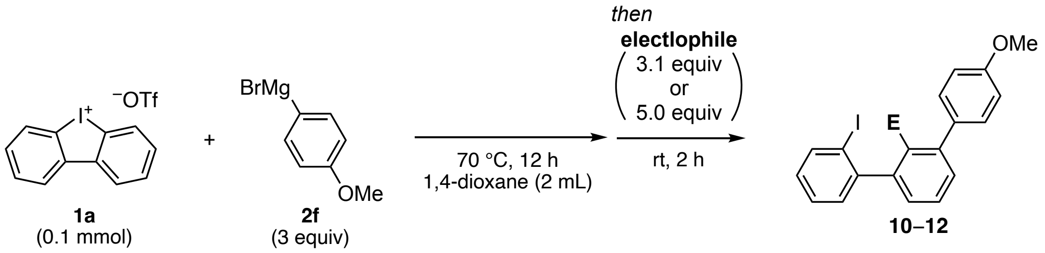

General procedure: A flame-dried 10 mL Schlenk tube equipped with a magnetic stir bar was charged with cyclic diaryliodonium salt **1a** (0.1 mmol, 1.0 equiv). The tube was purged with argon, and anhydrous 1,4-dioxane (2 mL) was added. The tube was then placed in a preheated aluminum block at 70 °C, and a solution of aryl Grignard reagent **2f** (3.0 equiv) was added dropwise via syringe. The reaction mixture was stirred at 70 °C for 12 h. After cooling to room temperature, *N*-bromosuccinimide (3.1 equiv) or *N*-iodosuccinimide (3.1 equiv) or iodomethane (5.0 equiv) was added, and the mixture was stirred for 2 h. Then, water was added to the mixture, and extracted with CH_2Cl_2 (3 × 5 mL). The combined organic layers were dried over anhydrous $MgSO_4$, concentrated under reduced pressure, and the residue was purified by preparative thin-layer chromatography to afford the desired products **10**, **11**, or **12**.

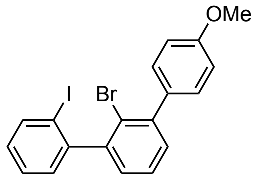

2'-Bromo-2-iodo-4''-methoxy-1,1':3',1''-terphenyl (10): White oil (17.9 mg, 39% yield); R_f 0.5 (hexane/ethyl acetate = 8/1); **^{1}H NMR** (600 MHz, $CDCl_3$) δ 7.95 (d, J = 7.9 Hz, 1H), 7.43-7.38 (m, 4H), 7.34 (dd, J = 7.6, 1.7 Hz, 1H), 7.29 (dd, J = 7.6, 1.7 Hz, 1H), 7.14 (dd, J = 7.4, 1.7 Hz, 1H), 7.08 (td, J = 7.7, 1.6 Hz, 1H), 6.97 (app d, J = 8.6 Hz, 2H), 3.86 (s, 3H); **^{13}C{^{1}H} NMR** (151 MHz, $CDCl_3$) δ 159.2, 147.1, 146.1, 143.3, 138.9, 134.1, 130.80, 130.79, 130.0, 129.5, 129.2, 128.1, 127.0, 124.2, 113.4, 99.8, 55.4; **HRMS** (EI^+) Calcd for $C_{19}H_{14}BrIO^+$ $[M]^+$ 463.9273, found 463.9267.

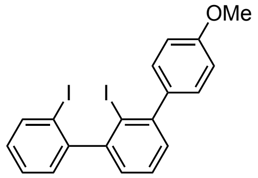

2,2'-Diiodo-4''-methoxy-1,1':3',1''-terphenyl (11): Brown oil (18.6 mg, 36% yield); R_f 0.5 (hexane/ethyl acetate = 8/1); **^{1}H NMR** (600 MHz, $CDCl_3$) δ 7.92 (dd, *J* = 7.9, 1.0 Hz, 1H), 7.44-7.40 (m, 2H), 7.32 (app d, *J* = 8.6, 2.5 Hz, 2H), 7.29-7.26 (m, 2H), 7.12 (dd, *J* = 7.6, 1.7 Hz, 1H), 7.09 (td, *J* = 7.7, 1.6 Hz, 1H), 6.96 (app d, *J* = 8.8 Hz, 2H), 3.86 (s, 3H); **^{13}C{^{1}H} NMR** (151 MHz, $CDCl_3$) δ 159.1, 150.2, 150.0, 147.5, 138.8, 137.7, 130.6, 130.0, 129.2, 129.1, 128.2, 128.0, 127.7, 113.2, 105.0, 100.0, 55.3; **HRMS** (EI^+) Calcd for $C_{19}H_{14}I_2O^+$ $[M]^+$ 511.9134, found 511.9139.

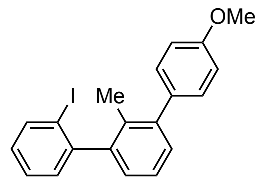

2-Iodo-4''-methoxy-2'-methyl-1,1':3',1''-terphenyl (12): White solid (16.5 mg, 42% yield); R_f 0.6 (hexane/ethyl acetate = 8/1); m.p. 115.7−117.1 °C; **^{1}H NMR** (600 MHz, $CDCl_3$) δ 7.94 (dd, *J* = 7.9, 1.0 Hz, 1H), 7.40 (td, *J* = 7.5, 1.2 Hz, 1H), 7.31-7.25 (m, 5H), 7.06-7.02 (m, 2H), 6.96 (app d, *J* = 8.7 Hz, 2H), 3.86 (s, 3H), 1.95 (s, 3H); **^{13}C{^{1}H} NMR** (151 MHz, $CDCl_3$) δ 158.6, 147.4, 145.2, 142.3, 139.0, 134.6, 133.4, 130.5, 129.8, 129.7, 128.7, 128.2, 128.1, 125.3, 113.6, 100.4, 55.4, 18.2; **HRMS** (EI^+) Calcd for $C_{20}H_{17}IO^+$ $[M]^+$ 400.0324, found 400.0306.

6. X-Ray Crystallographic Analysis

Experimental for compound 3at. Single crystals of **3at** suitable for X-ray diffraction were obtained by recrystallization from hexane/CH_2Cl_2. Diffraction data were collected on a Rigaku XtaLAB Synergy diffractometer equipped with a HyPix3000 hybrid pixel array detector and a Cu Kα source (λ = 1.54184 Å) with a mirror monochromator (PhotonJet (Cu), micro-focus sealed tube). The measurement was conducted at 100 K under a nitrogen stream using ω scans. Empirical multi-scan absorption correction (SCALE3 ABSPACK, CrysAlisPro 1.171.43.125a) was applied. Using Olex2,[10] the structure was solved with the SHELXT[11] structure solution program using Intrinsic Phasing and refined with the SHELXL[12] refinement package using Least Squares minimisation.

Crystallographic data of **3at** have been deposited on the Cambridge Crystallographic Data Center, deposition no. CCDC 2498673.

Table S4. Crystal data and structure refinements for 2,2'''''-Diiodo-1,1':3',1'':2'',1''':2''',1'''':3'''',1'''''-sexiphenyl (**3at**).

CCDC number	2498673
Empirical formula	$C_{36}H_{24}I_2$
Formula weight	710.40
Space system	Triclinic
Space group	P −1
a/Å	10.7107 (2)
b/Å	11.0491 (2)
c/Å	12.2054 (2)
a/°	76.7260 (10)
b/°	85.7920 (10)
g/°	84.8380 (10)
Volume/Å^3	1398.07
Z	2
Temperature/K	100(1)
2θ range for data collection/°	7.45 to 136.49
ρ_{calcd} g/cm^3	1.687
μ/mm^{-1}	17.832

F (000)	692
Crystal_size/mm^3	0.15 × 0.11 × 0.06
Radiation	Cu Kα (λ = 1.54184)
Reflections collected	13317
Independent	5087
Index ranges	$-12 \leqq h \leqq 12$, $-13 \leqq k \leqq 13$, $-14 \leqq l \leqq 14$
Data/restraints/parameters	5087/0/344
Final R indexes R [I>2σ(I)]gt	$R_1 = 0.0235$, $wR_2 = 0.0596$
Final R indexes R [all data]	$R_1 = 0.0244$, $wR_2 = 0.0602$
Goodness-of-fit on F^2	1.060
Largest peak/deepest hole eÅ^{-3}	+0.905/−0.852

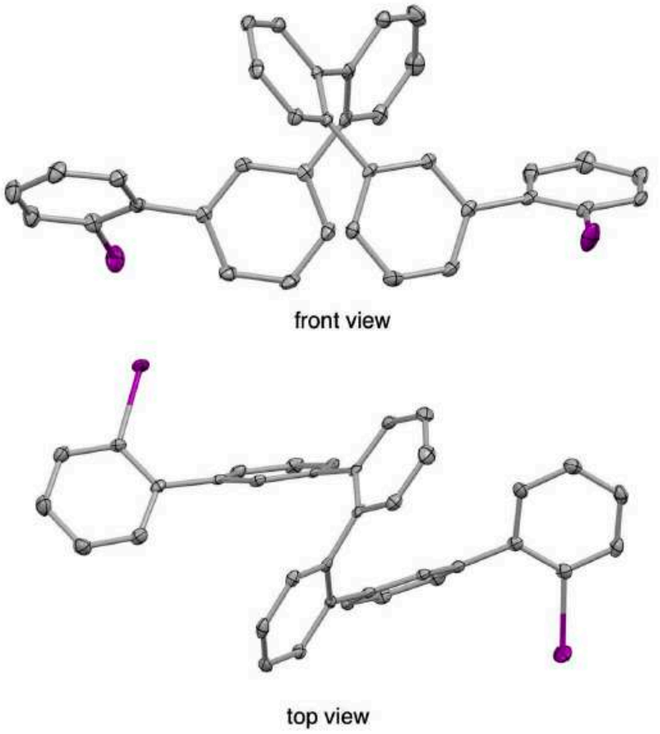

Figure S11. Thermal ellipsoid (50 % probability) plot of **3at**, CCDC No. 2498673. Color code of atoms: carbon, gray; iodine, purple.

Experimental for compound 5. Single clear colourless block-shaped crystals of **5** were obtained by recrystallisation from MeOH /CH_2Cl_2. Diffraction data were collected on a Rigaku XtaLAB Synergy diffractometer equipped with a HyPix3000 hybrid pixel array detector and a Cu Kα source (λ = 1.54184 Å) with a mirror monochromator (PhotonJet (Cu), micro-focus sealed tube). The crystal was kept at a steady T = 100.03(12) K during data collection. Using Olex2,[10] the structure was solved with the SHELXT[11] structure solution program using Intrinsic Phasing and refined with the SHELXL[12] refinement package using Least Squares minimisation.
Crystallographic data of **5** have been deposited on the Cambridge Crystallographic Data Center, deposition no. CCDC 2534078.

Table S5. Crystal data and structure refinements for 4'',4'''',5',5'''-tetrakis(trifluoromethyl)-1,1':2',1'':2'',1''':2''',1'''':2'''',1'''''-sexiphenyl (**5**).

CCDC number	2534078
Empirical formula	$C_{20}H_{11}F_6$
Formula weight	730.60
Space system	orthorhombic
Space group	*Pbcn*
a/Å	18.74846(13)
b/Å	10.78495(8)
c/Å	15.64900(12)
a/°	90
b/°	90
g/°	90
Volume/Å^3	3164.25(4)
Z	8
Temperature/K	100.03(12)
2θ range for data collection/°	9.43–136.51°
ρ_{calcd} g/cm^3	1.534
μ/mm^{-1}	1.212
F (000)	1480
Crystal_size/mm^3	0.10×0.10×0.05
Radiation	Cu Kα (λ = 1.54184)
Reflections collected	33188

Independent	2874
Index ranges	–22≦h≦22, –12≦k≦12, –18≦l≦13
Data/restraints/parameters	2874/0/236
Final R indexes R [I>2σ(I)]gt	R_1 = 0.0469, wR_2 = 0.1247
Final R indexes R [all data]	R_1 = 0.0490, wR_2 = 0.1267
Goodness-of-fit on F^2	1.049
Largest peak/deepest hole eÅ^{-3}	+0.634/−0.418

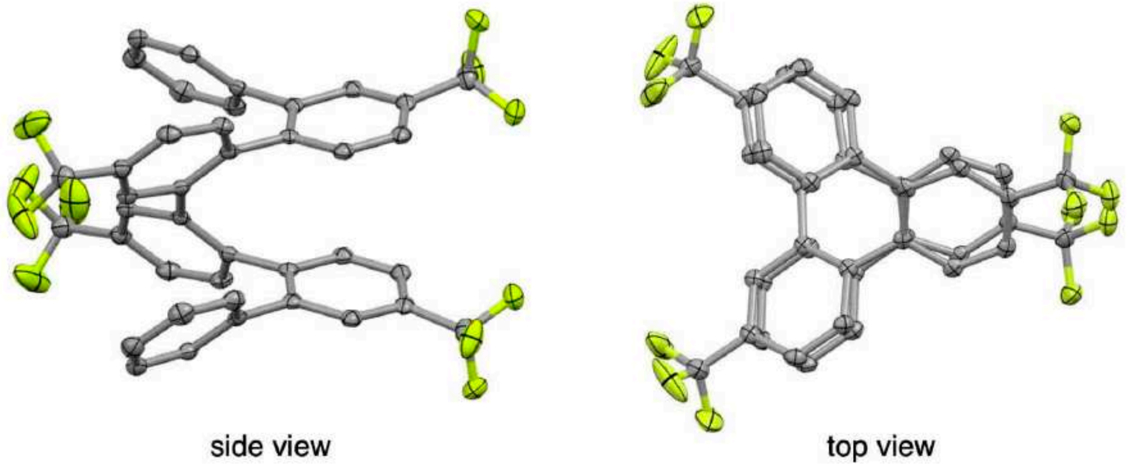

Figure S12. Thermal ellipsoid (50 % probability) plot of **5**, CCDC No. 2534078. Color code of atoms: carbon, gray; fluorine, yellow.

Experimental for compound 7. Single crystals of **7** were obtained by recrystallization from hexane/CH_2Cl_2. Diffraction data were collected on a Rigaku XtaLAB Synergy diffractometer equipped with a HyPix3000 hybrid pixel array detector and a Cu Kα source (λ = 1.54184 Å) with a mirror monochromator (PhotonJet (Cu), micro-focus sealed tube). The crystal was kept at 100.00(10) K during data collection. Using Olex2,[10] the structure was solved with the SHELXT[11] structure solution program using Intrinsic Phasing and refined with the SHELXL[12] refinement package using Least Squares minimisation.
Crystallographic data of **7** have been deposited on the Cambridge Crystallographic Data Center, deposition no. CCDC 2534079.

Table S6. Crystal data and structure refinements for 4'''',5'''-dichloro-4'',5'-bis(trifluoromethyl)-1,1':2',1'':2'',1''':2''',1'''':3'''',1'''''-sexiphenyl (**7**).

CCDC number	2534079
Empirical formula	$C_{38}H_{22}Cl_2F_6$
Formula weight	663.45
Space system	monoclinic
Space group	$P2_1/c$
a/Å	15.47557(12)
b/Å	9.75087(6)
c/Å	20.02468(17)
a/°	90
b/°	100.3720(8)
g/°	90
Volume/Å^3	2972.35(4)
Z	4
Temperature/K	100.00(10)
2θ range for data collection/°	5.806 to 136.644
ρ_{calcd} g/cm^3	1.483
μ/mm^{-1}	2.543
F (000)	1352.0
Crystal_size/mm^3	0.2 × 0.2 × 0.05
Radiation	Cu Kα (λ = 1.54184)
Reflections collected	29455
Independent	5406

Index ranges	-18 ≤ h ≤ 18, -11 ≤ k ≤ 7, -24 ≤ l ≤ 23
Data/restraints/parameters	5406/0/416
Final R indexes R [I>2σ(I)]gt	R_1 = 0.0382, wR_2 = 0.1022
Final R indexes R [all data]	R_1 = 0.0397, wR_2 = 0.1034
Goodness-of-fit on F^2	1.036
Largest peak/deepest hole eÅ^{-3}	+0.56/−0.55

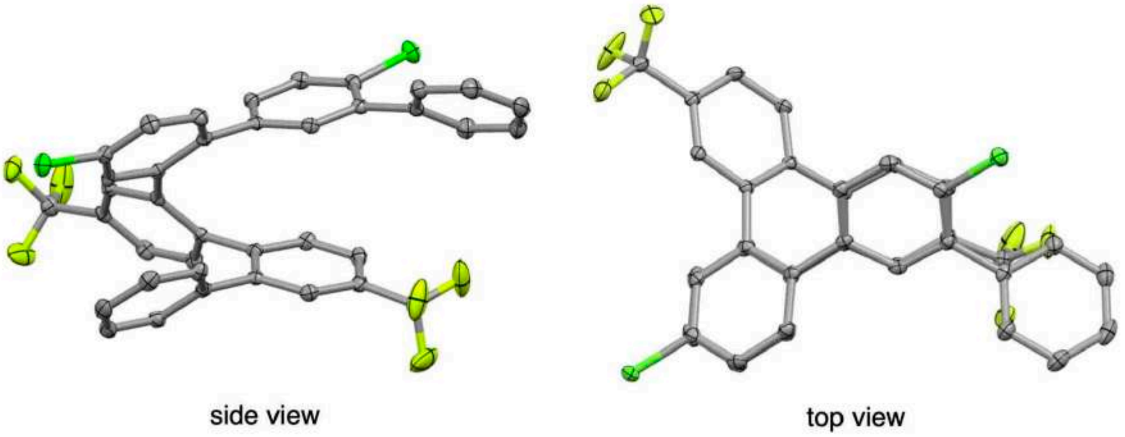

Figure S13. Thermal ellipsoid (50 % probability) plot of **7**, CCDC No. 2534079. Color code of atoms: carbon, gray; fluorine, yellow; chlorine, green.

7. DFT Calculations

Computational Method

All the density functional theory (DFT) calculations were carried out using the Gaussian 16 program.[13] Geometry optimizations were performed with the M06-2X functional[14-16] and a combined basis set B1 (the SDD effective core potential[17] for iodine and the 6-31G(d) basis set for all other atoms). Harmonic frequency calculations were performed for each stationary point to ensure that it is an energy minimum (no imaginary frequency). For the stationary points, single-point energy calculations were performed with the M06-2X functional and a combined basis set B2 (the SDD effective core potential for iodine and the 6-311++G(2df,2p) basis set for all other atoms) and the SMD model[18] with tetrahydrofuran as the solvent. The single-point energies corrected by the thermal correction to Gibbs free energies (TCG, obtained from frequency calculations) were used as the Gibbs free energies, corresponding to the reference state of 1 mol/L, 298.15 K. The 3-D structures were drawn using CYLView software.[19]

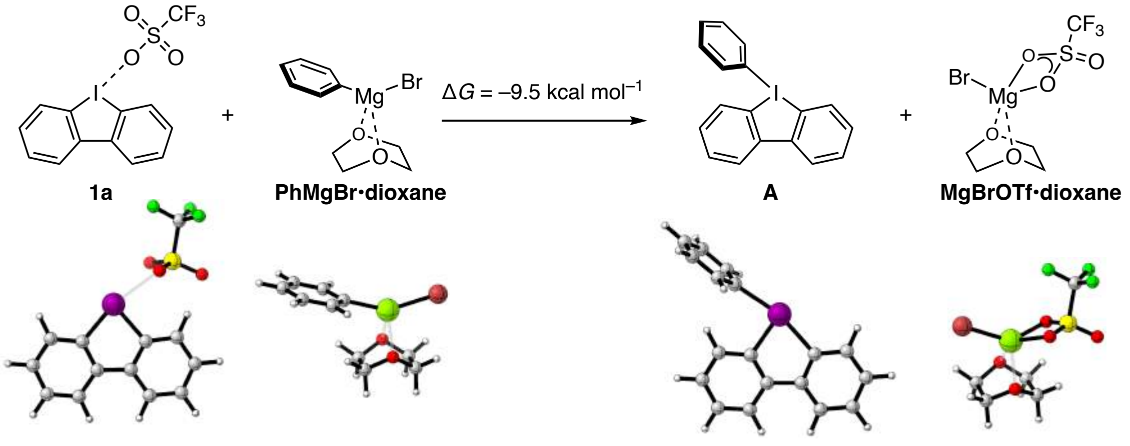

Figure S14. A model reaction between cyclic diaryliodonium triflate and PhMgBr•dioxane leading to cyclic triaryliodane and MgBrOTf•dioxane.

Table S7. Energy data for the optimized structures (Figure S14; hartrees).

Structure	E(M06-2X/B1)	TCG	E(M06-2X/B2)	TCG+ E(M06-2X/B2)
**1a**	-1434.43422999	0.139515	-1434.88646962	-1434.746955
**PhMgBr•dioxane**	-3003.46235023	0.057167	-3005.96263956	-3005.905473
**A**	-704.70012346	0.207342	-704.938923724	-704.7315817
**MgBrOTf•dioxane**	-3733.22137309	-0.007698	-3735.91675622	-3735.924454

Cartesian coordinates

1a

C	-2.141806	3.715631	-0.039122
C	-2.989125	2.618399	0.058720
C	-2.479181	1.316221	-0.016767
C	-1.098356	1.201530	-0.197160
C	-0.218928	2.261397	-0.301531
C	-0.769062	3.541280	-0.215831
H	-2.555361	4.716964	0.025901
H	-4.054905	2.769876	0.198424
H	0.846421	2.110668	-0.436594
H	-0.108919	4.399572	-0.285319
C	-3.276935	0.084991	0.080568
C	-2.586349	-1.125871	-0.011144
C	-4.664860	0.031293	0.257728
C	-3.214048	-2.360522	0.066976
C	-5.314248	-1.194637	0.337572
H	-5.239087	0.949327	0.334154
C	-4.595850	-2.388484	0.243490
H	-2.652084	-3.287021	-0.004229
H	-6.390407	-1.222125	0.475446
H	-5.109776	-3.342198	0.308431
I	-0.494826	-0.850126	-0.275284
O	1.793795	0.086780	-0.698673
S	2.713377	0.286927	0.479670
O	2.299461	-0.502137	1.636841
O	3.069858	1.683978	0.684682
C	4.255561	-0.526168	-0.127273
F	5.202456	-0.468763	0.806591
F	4.018655	-1.809064	-0.412504
F	4.704452	0.074656	-1.227534

PhMgBr•dioxane

C	1.749150	0.524893	0.001607
C	2.501464	0.535314	-1.190589

C	2.503438	0.527033	1.192562
C	3.898530	0.548272	-1.202800
H	1.988834	0.541403	-2.154391
C	3.900521	0.539976	1.202523
H	1.992402	0.526560	2.157233
C	4.604507	0.547234	-0.000713
H	4.435837	0.561751	-2.148355
H	4.439404	0.546916	2.147251
H	5.691181	0.559512	-0.001566
Mg	-0.350376	0.472029	0.001564
Br	-2.419993	1.659666	0.001290
O	-0.815189	-1.294053	1.211332
O	-0.814629	-1.290900	-1.213288
C	-2.114871	-1.717252	0.775263
H	-2.315145	-2.711085	1.191507
H	-2.824517	-0.995038	1.177810
C	0.168045	-2.242770	0.774819
H	1.124546	-1.902934	1.172930
H	-0.083823	-3.223926	1.192975
C	-2.114499	-1.715172	-0.778942
H	-2.823753	-0.991620	-1.179759
H	-2.314854	-2.707823	-1.197952
C	0.168648	-2.240452	-0.778839
H	-0.082138	-3.220501	-1.200232
H	1.125251	-1.898710	-1.175083

A

C	-0.985540	3.761471	-0.000697
C	-1.968945	2.779659	-0.000532
C	-1.631981	1.418348	-0.000279
C	-0.273419	1.105423	-0.000202
C	0.727965	2.059402	-0.000362
C	0.362633	3.405748	-0.000612
H	-1.271767	4.808925	-0.000892
H	-3.014481	3.071498	-0.000602
H	1.773243	1.770712	-0.000294

H	1.134168	4.169537	-0.000740
C	-2.622107	0.317140	-0.000092
C	-2.095048	-0.969045	0.000143
C	-4.012004	0.498583	-0.000136
C	-2.931439	-2.078765	0.000334
C	-4.850869	-0.610946	0.000054
H	-4.448734	1.493411	-0.000316
C	-4.315273	-1.901773	0.000289
H	-2.518479	-3.087302	0.000518
H	-5.927852	-0.470817	0.000021
H	-4.977959	-2.763097	0.000437
I	0.165525	-1.026493	0.000187
C	2.400052	-0.500073	0.000120
C	3.102532	-0.417262	1.202794
C	3.102583	-0.417818	-1.202563
C	4.489005	-0.246986	1.207034
C	4.489056	-0.247545	-1.206825
C	5.182674	-0.160970	0.000099
H	5.027382	-0.181807	2.149178
H	5.027471	-0.182802	-2.148976
H	6.260994	-0.029177	0.000091
H	2.571917	-0.480744	2.152594
H	2.572007	-0.481740	-2.152356

MgBrOTf•dioxane

Mg	-0.659072	0.212965	0.022429
Br	-1.817862	2.273466	0.039703
O	-1.757188	-1.240199	1.136432
O	-1.938364	-1.036832	-1.263584
C	-3.138676	-1.000231	0.823269
H	-3.731483	-1.837803	1.206759
H	-3.413115	-0.082809	1.344952
C	-1.328255	-2.483364	0.560951
H	-0.283801	-2.612702	0.854966
H	-1.921173	-3.295867	0.994310
C	-3.249915	-0.841332	-0.718514

H	-3.551416	0.167419	-0.999330
H	-3.930153	-1.574316	-1.165863
C	-1.500907	-2.372071	-0.981459
H	-2.247321	-3.074882	-1.367382
H	-0.557361	-2.507970	-1.507953
O	0.913900	-0.656583	-1.009023
S	1.799311	-0.712664	0.206360
O	0.992137	-0.069481	1.287183
O	2.431234	-1.978627	0.494177
C	3.148263	0.493970	-0.158008
F	2.613156	1.672115	-0.453709
F	3.863126	0.062631	-1.188563
F	3.930835	0.617096	0.904506

8. References

[1] Y. Wu, X. Peng, B. Luo, F. Wu, B. Liu, F. Song, P. Huang, S. Wen, *Org. Biomol. Chem.* **2014**, *12*, 9777–2780.

[2] K. Zhu, Z. Song, Y. Wang, F. Zhang, *Org. Lett.* **2020**, *22*, 9356−9359.

[3] M. Ding, W. Hua, M. Liu, F. Zhang, *Org. Lett.* **2020**, *22*, 7419−7423.

[4] M. Liu, H. Jiang, J. Tang, Z. Ye, F. Zhang, Y. Wu, *Org. Lett.* **2023**, *25*, 2777−2781.

[5] B. Wu, N. Yoshikai, *Angew. Chem. Int. Ed.* **2015**, 54, 8736–8739.

[6] W. Liang, Y. Yang, M. Zhang, C. Li, Y. Ran, J. Lan, Z. Bin, J. You, *Angew. Chem. Int. Ed.* **2021**, 60, 3493–3497.

[7] D. Zhu, Q. Liu, B. Luo, M. Chen, R. Pi, P. Huang, S. Wen, *Adv. Synth. Catal.* **2013**, *355*, 2172–2178.

[8] M. Jiang, J. Guo, B. Liu, Q. Tan, B. Xu, *Org. Lett.* **2019**, *21*, 8328−8333.

[9] M. Trose, F. Lazreg, T. Chang, F. Nahra, D. B. Cordes, A. M. Slawin, C. S. J. Cazin, *ACS Catal.* **2017**, 7, 238–242.

[10] O. V. Dolomanov, L. J. Bourhis, R. J. Gildea, J. A. K. Howard, H. Puschmann, *J. Appl. Cryst.* **2009**, *42*, 339-341.

[11] G. M. Sheldrick, *Acta Cryst.* **2015**, *A71*, 3-8.

[12] G. M. Sheldrick, *Acta Cryst.* **2015**, *C71*, 3-8.

[13] M. J. Frisch, G. W. Trucks, H. B. Schlegel, G. E. Scuseria, M. A. Robb, J. R. Cheeseman, G. Scalmani, V. Barone, G. A. Petersson, H. Nakatsuji, X. Li, M. Caricato, A. V. Marenich, J. Bloino, B. G. Janesko, R. Gomperts, B. Mennucci, H. P. Hratchian, J. V. Ortiz, A. F. Izmaylov, J. L. Sonnenberg, D. Williams-Young, F. Ding, F. Lipparini, F. Egidi, J. Goings, B. Peng, A. Petrone, T. Henderson, D. Ranasinghe, V. G. Zakrzewski, J. Gao, N. Rega, G. Zheng, W. Liang, M. Hada, M. Ehara, K. Toyota, R. Fukuda, J. Hasegawa, M. Ishida, T. Nakajima, Y. Honda, O. Kitao, H. Nakai, T. Vreven, K. Throssell, J. A. Montgomery, Jr., J. E. Peralta, F. Ogliaro, M. J. Bearpark, J. J. Heyd, E. N. Brothers, K. N. Kudin, V. N. Staroverov, T. A. Keith, R. Kobayashi, J. Normand, K. Raghavachari, A. P. Rendell, J. C. Burant, S. S. Iyengar, J. Tomasi, M. Cossi, J. M. Millam, M. Klene, C. Adamo, R. Cammi, J. W. Ochterski, R. L. Martin, K. Morokuma, O. Farkas, J. B. Foresman, Gaussian 16, Revision A.03; Gaussian, Inc.: Wallingford, CT (2016).

[14] Y. Zhao, D. G. Truhlar, *J. Phys. Chem. A* **2006**, *110*, 13126–13130.

[15] Y. Zhao, D. G. Truhlar, *Acc. Chem. Res.* **2008**, *41*, 157–167.

[16] Y. Zhao, D. G. Truhlar, *Chem. Phys. Lett.* **2011**, *502*, 1–13.

[17] M. Dolg, U. Wedig, H. Stoll, H. Preuss, *J. Chem. Phys.* **1987**, *86*, 866–872.

[18] A. V. Marenich, C. J. Cramer, D. G. Truhlar, *J. Phys. Chem. B* **2009**, *113*, 6378–6396.

[19] C. Y. Legault, *CYLview20*; Université de Sherbrooke, 2020 (http://www.cylview.org).

9. ¹H and ¹³C NMR Spectra

¹H NMR spectrum of **3aa** (600 MHz, $CDCl_3$).

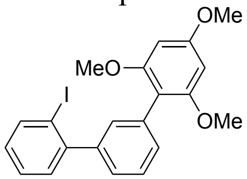

3aa
(*meta* only)

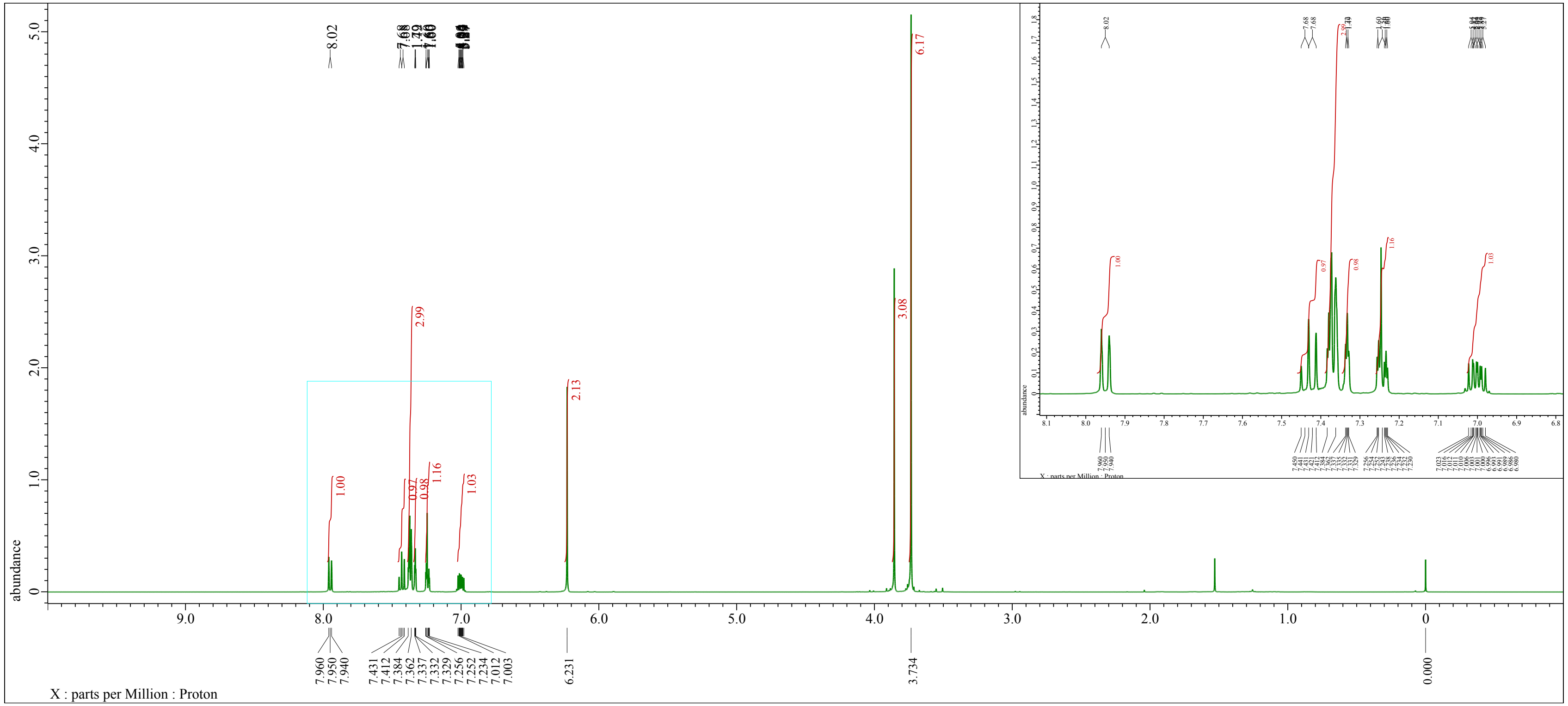

^{13}C NMR spectrum of **3aa** (151 MHz, $CDCl_3$).

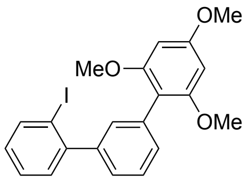

3aa
(*meta* only)

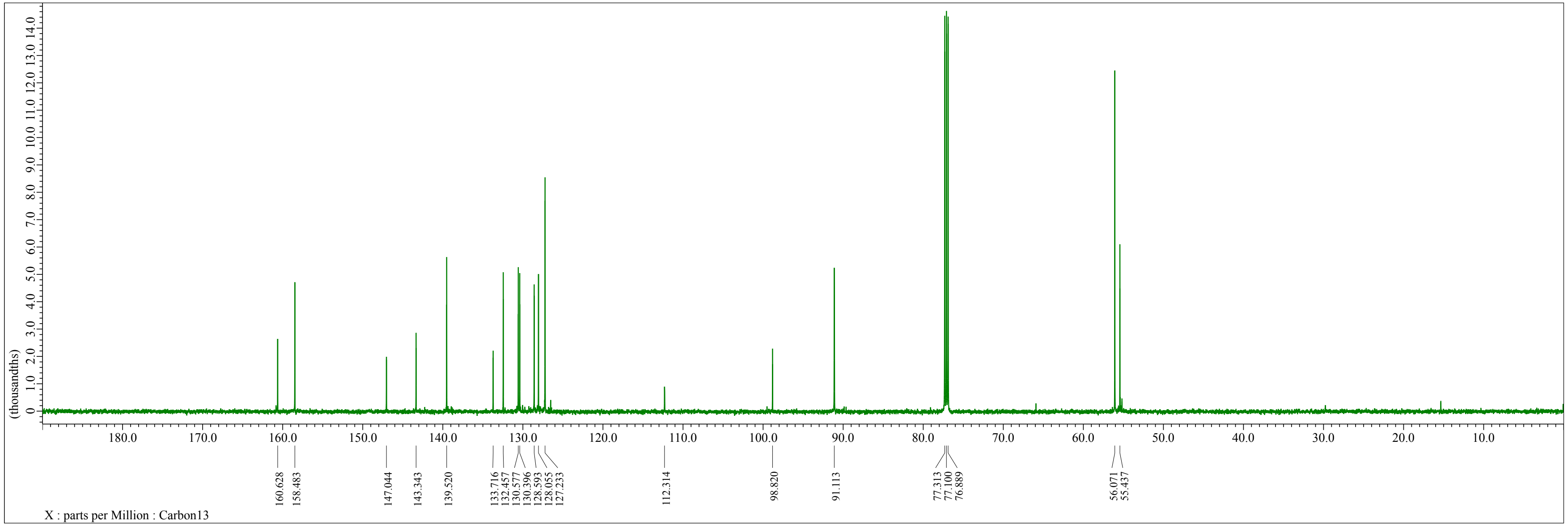

^{1}H NMR spectrum of **3ab** (400 MHz, $CDCl_3$).

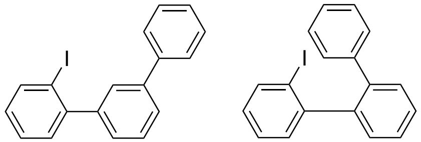

3ab
(*meta*:*ortho* = 97:3)

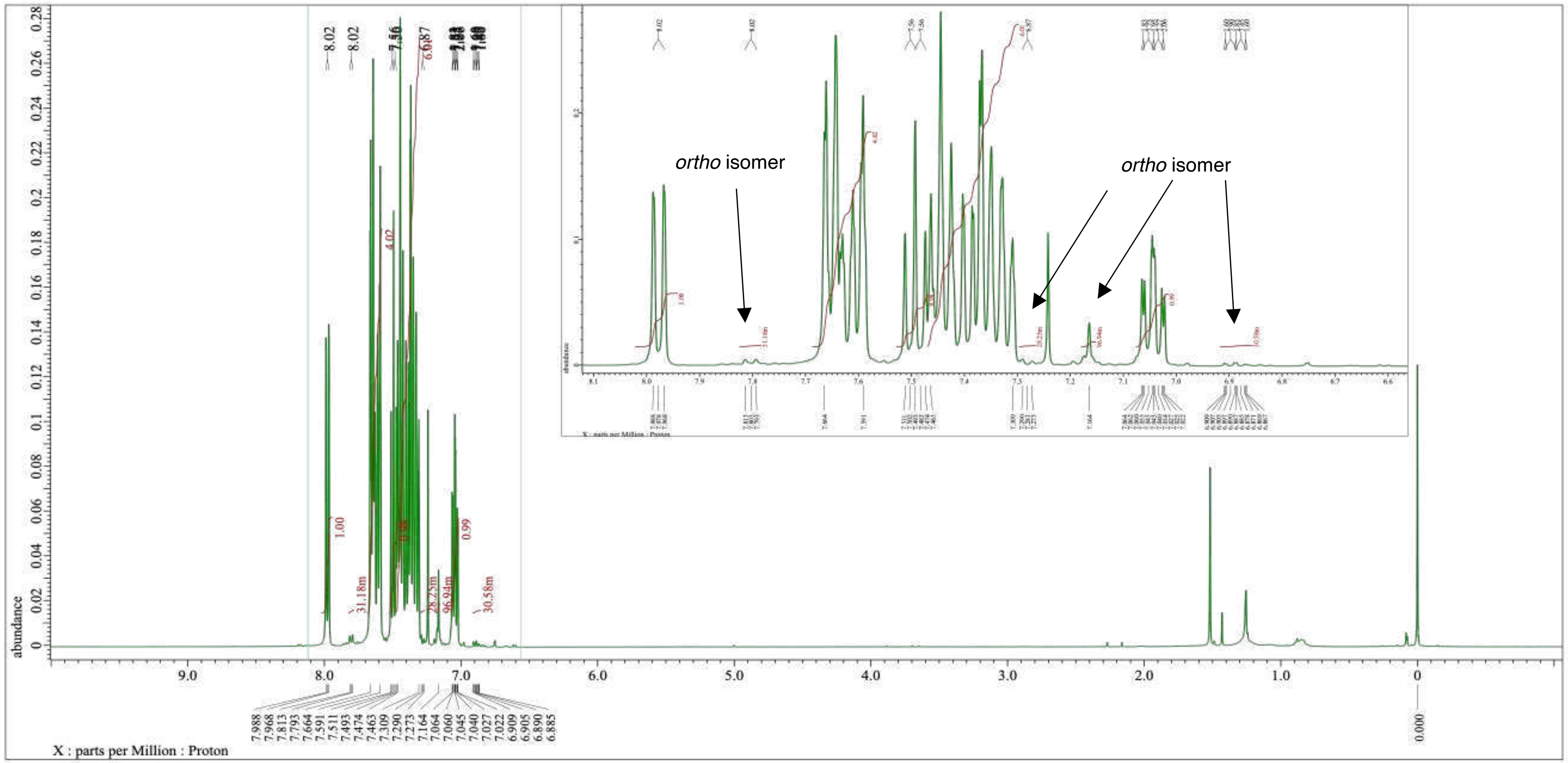

^{13}C NMR spectrum of **3ab** (151 MHz, $CDCl_3$).

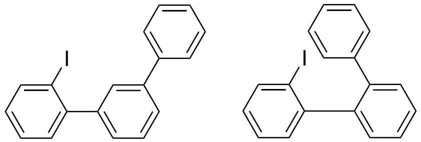

3ab
(*meta*:*ortho* = 97:3)

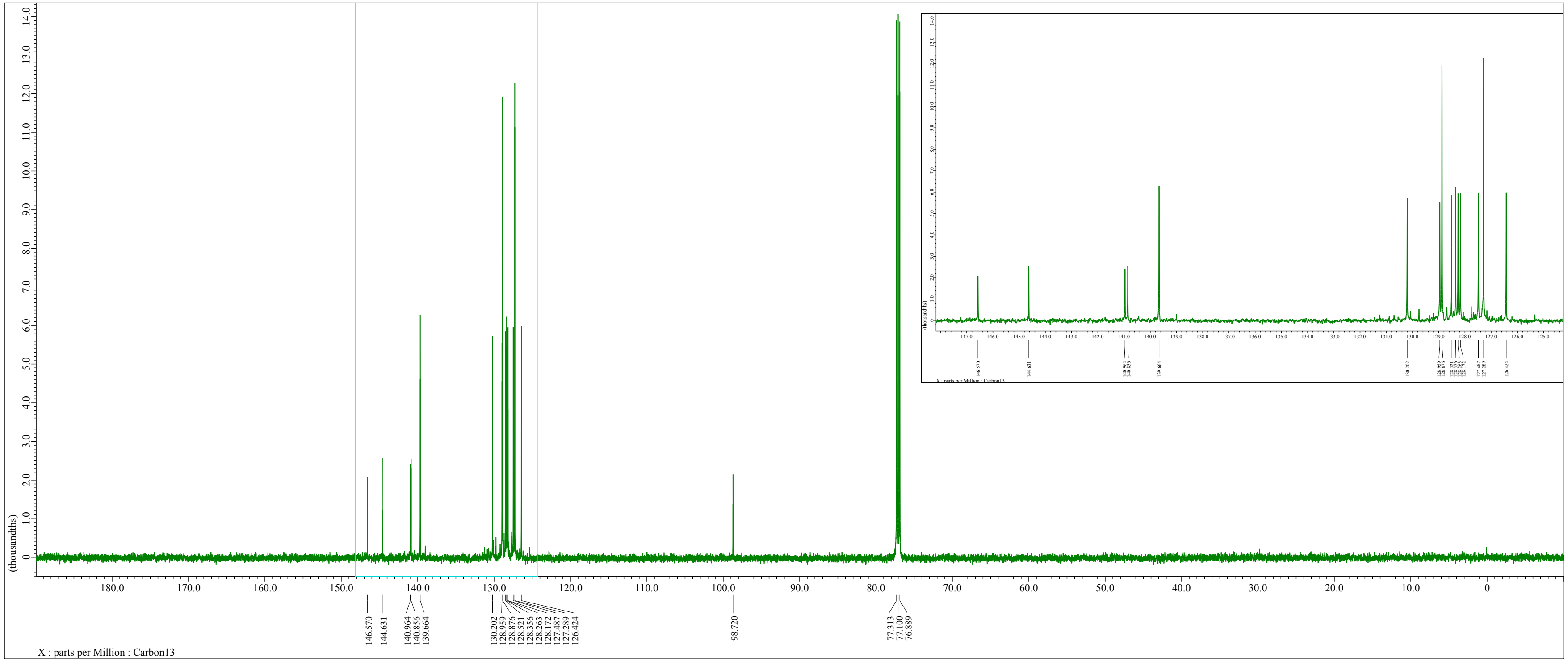

^{1}H NMR spectrum of **3ac** (600 MHz, $CDCl_3$).

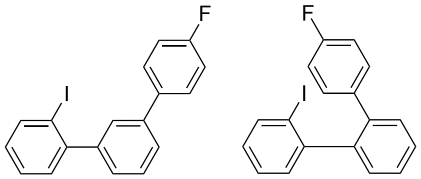

3ac
(*meta*:*ortho* = 97:3)

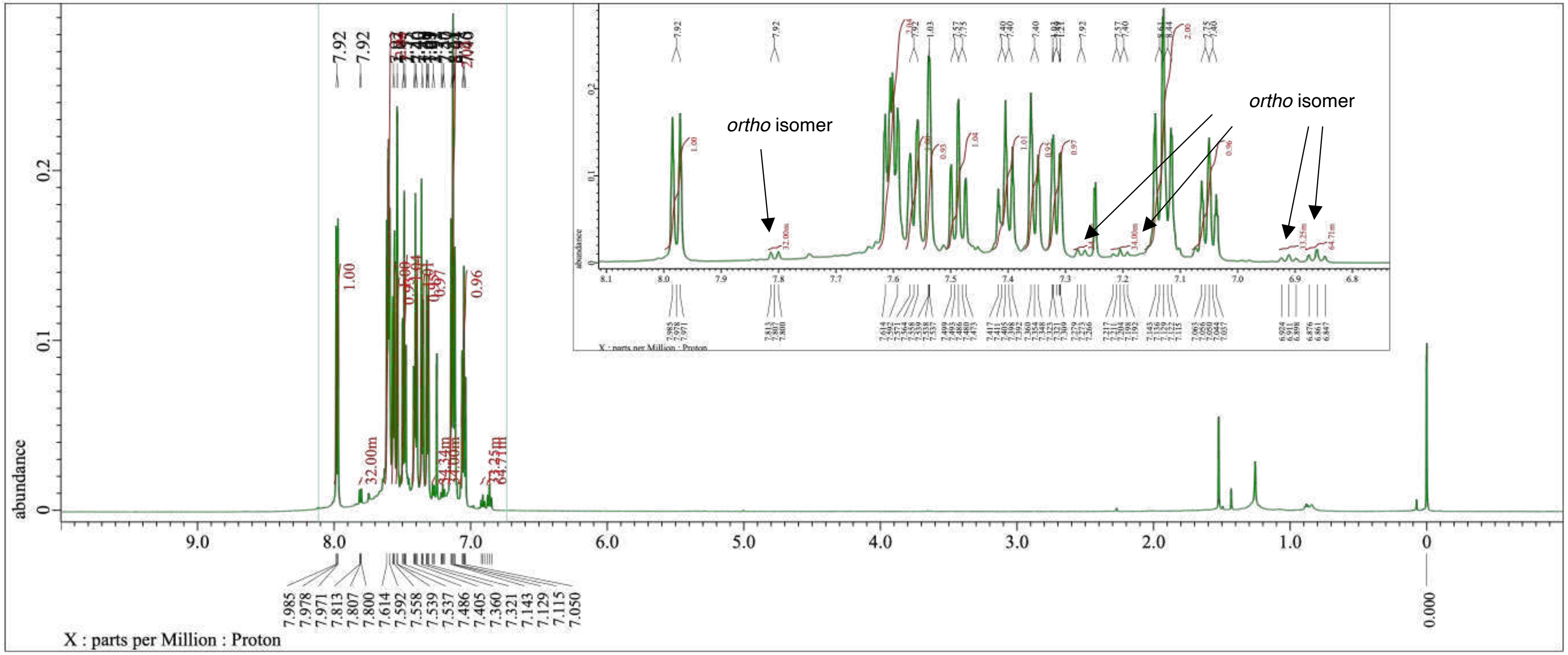

^{19}F NMR spectrum of **3ac** (376 MHz, $CDCl_3$).

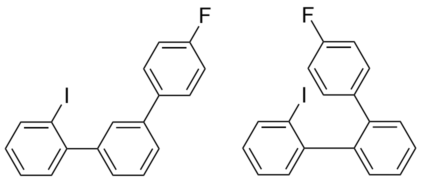

3ac
(*meta*:*ortho* = 97:3)

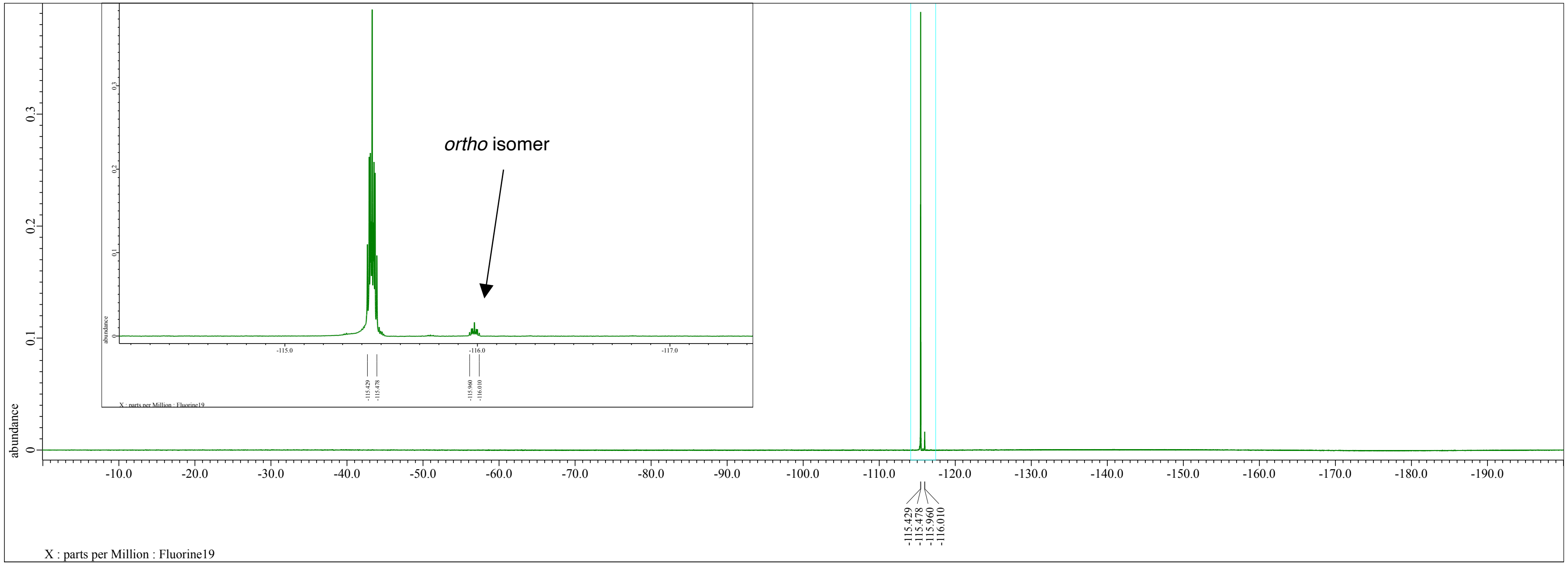

^{13}C NMR spectrum of **3ac** (151 MHz, $CDCl_3$).

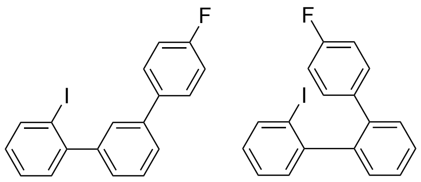

3ac
(*meta*:*ortho* = 97:3)

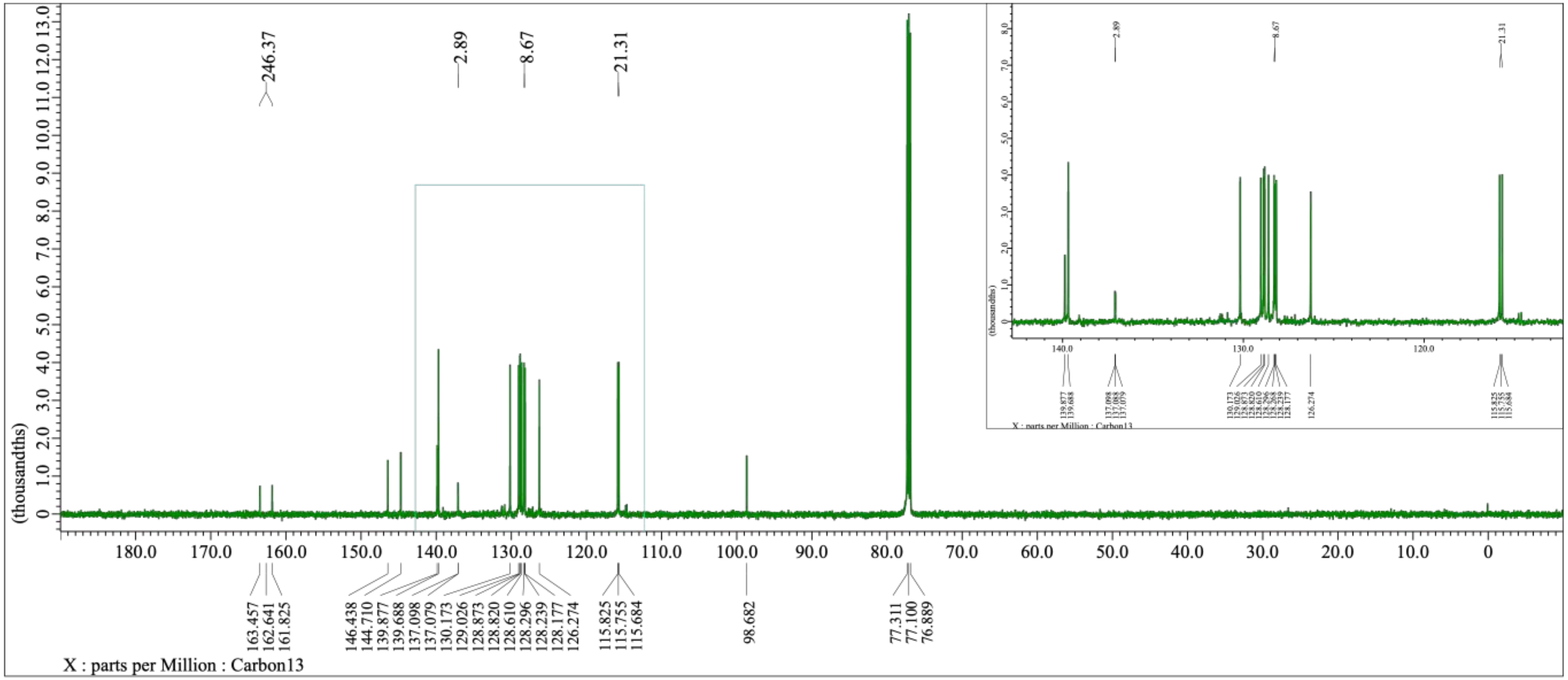

^{1}H NMR spectrum of **3ad** (400 MHz, $CDCl_3$).

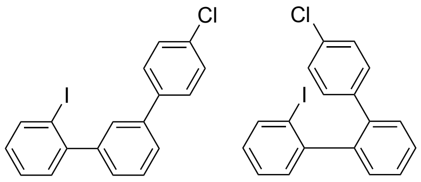

3ad
(*meta*:*ortho* = 97:3)

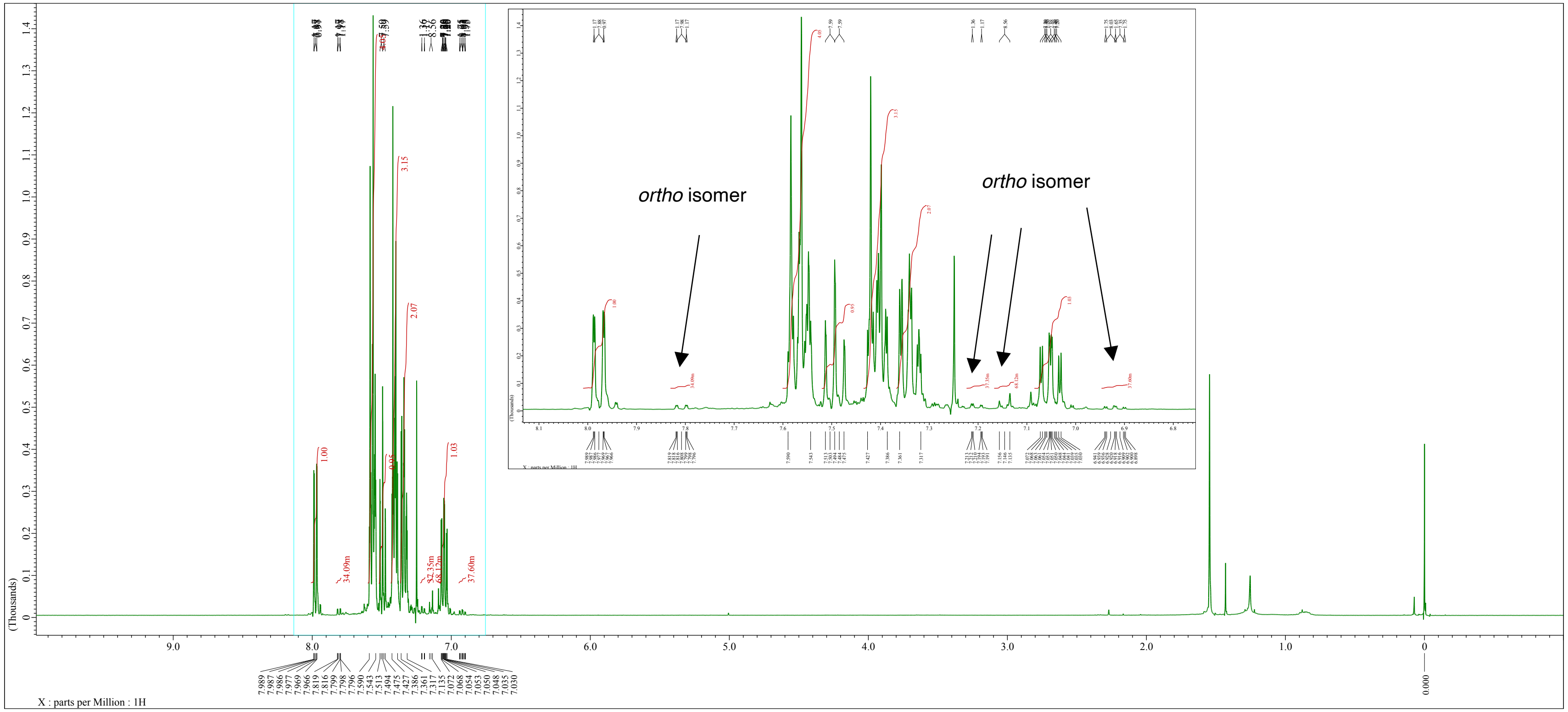

^{13}C NMR spectrum of **3ad** (151 MHz, $CDCl_3$).

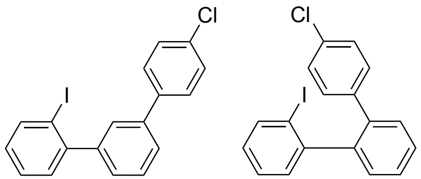

3ad
(*meta*:*ortho* = 97:3)

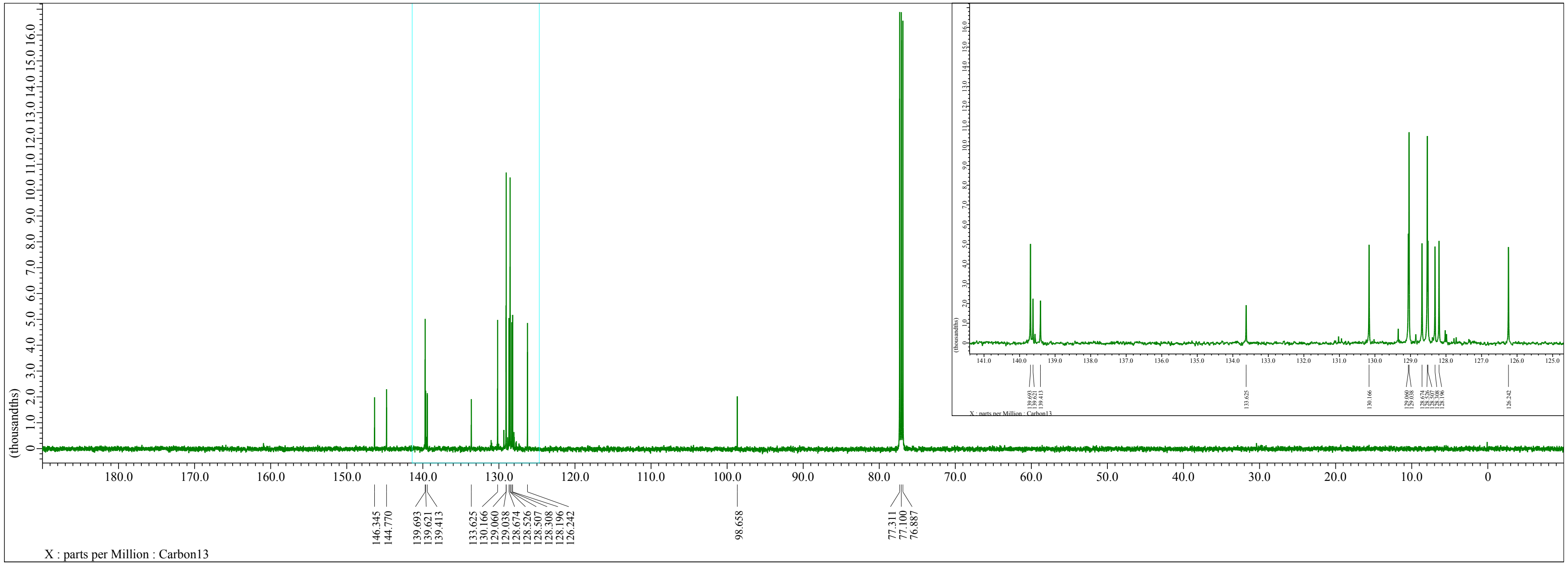

^{1}H NMR spectrum of **3ae** (400 MHz, $CDCl_3$).

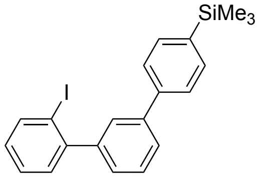

3ae
(*meta* only)

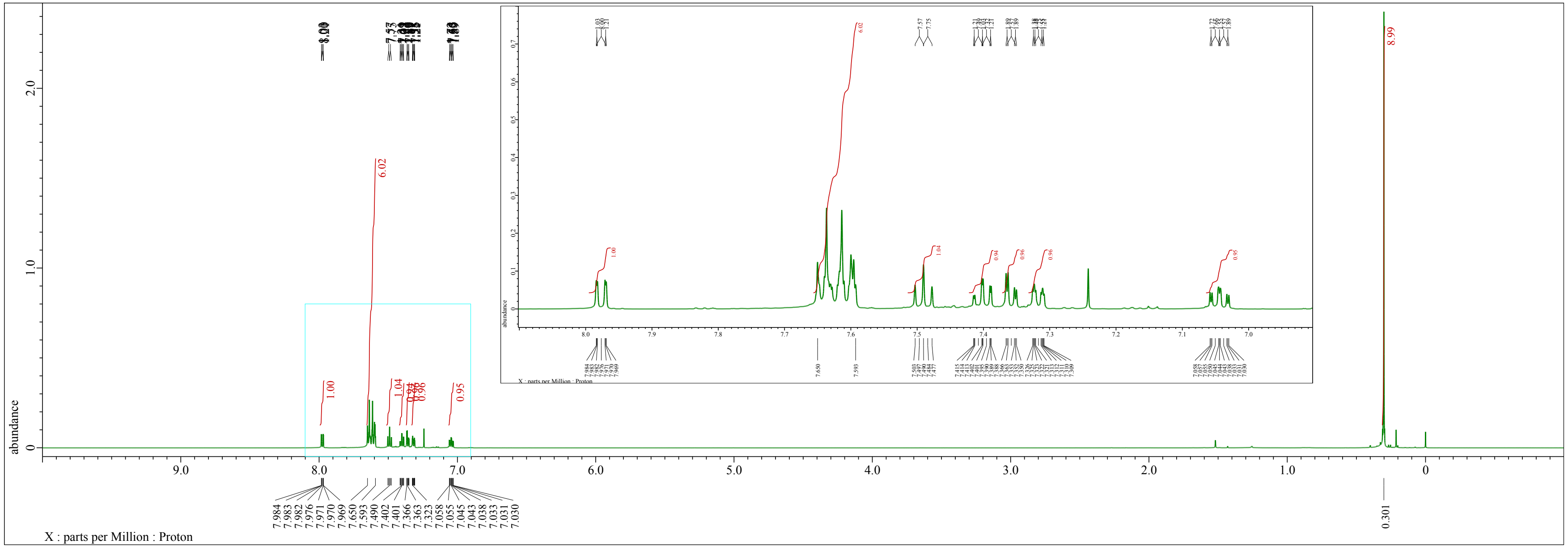

^{13}C NMR spectrum of **3ae** (151 MHz, $CDCl_3$).

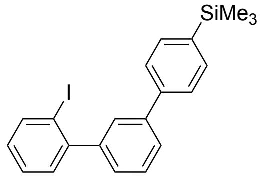

3ae
(*meta* only)

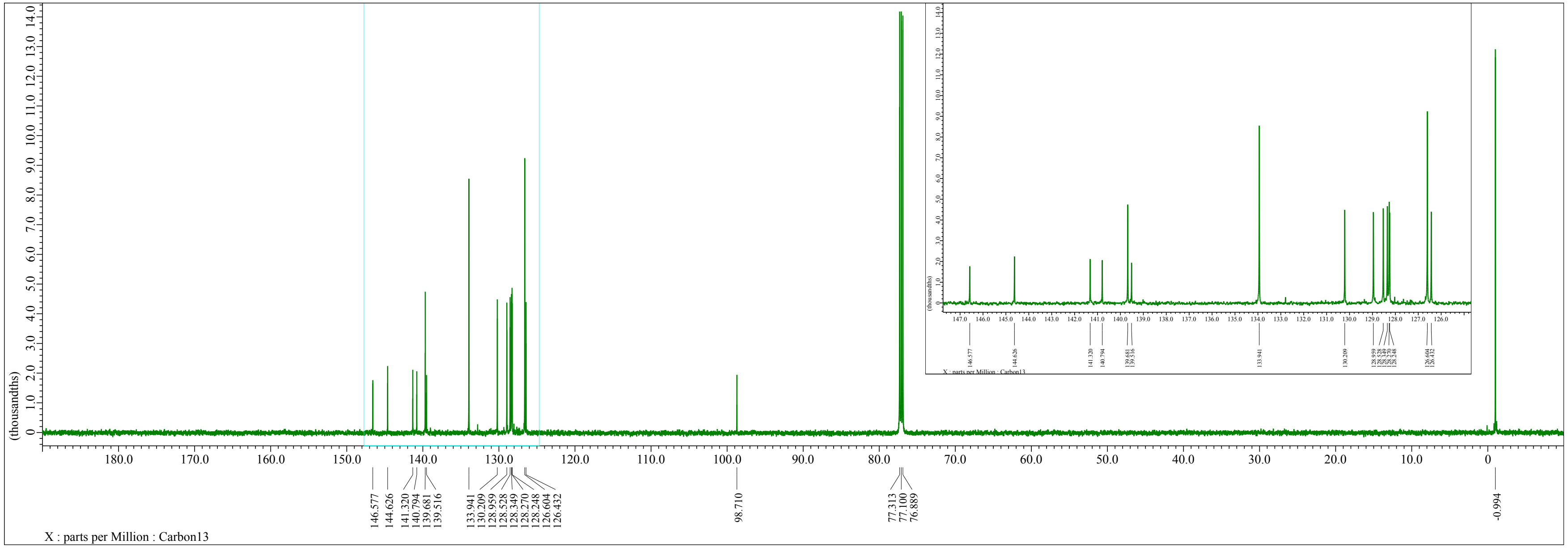

^{1}H NMR spectrum of **3af** (400 MHz, $CDCl_3$).

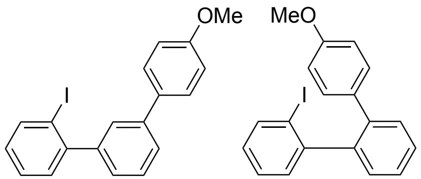

3af
(*meta*:*ortho* = 97:3)

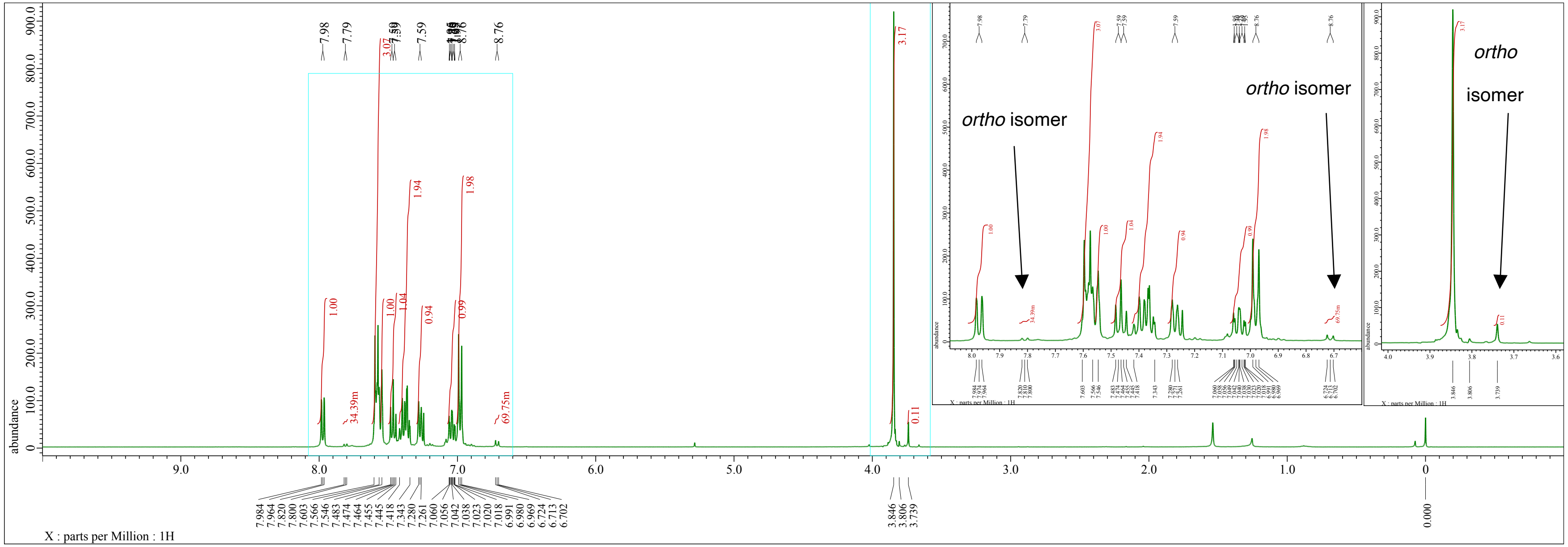

^{13}C NMR spectrum of **3af** (151 MHz, $CDCl_3$).

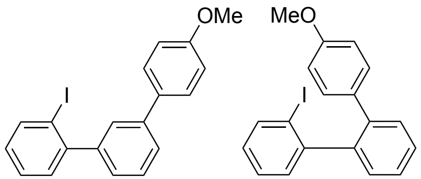

3af
(*meta*:*ortho* = 97:3)

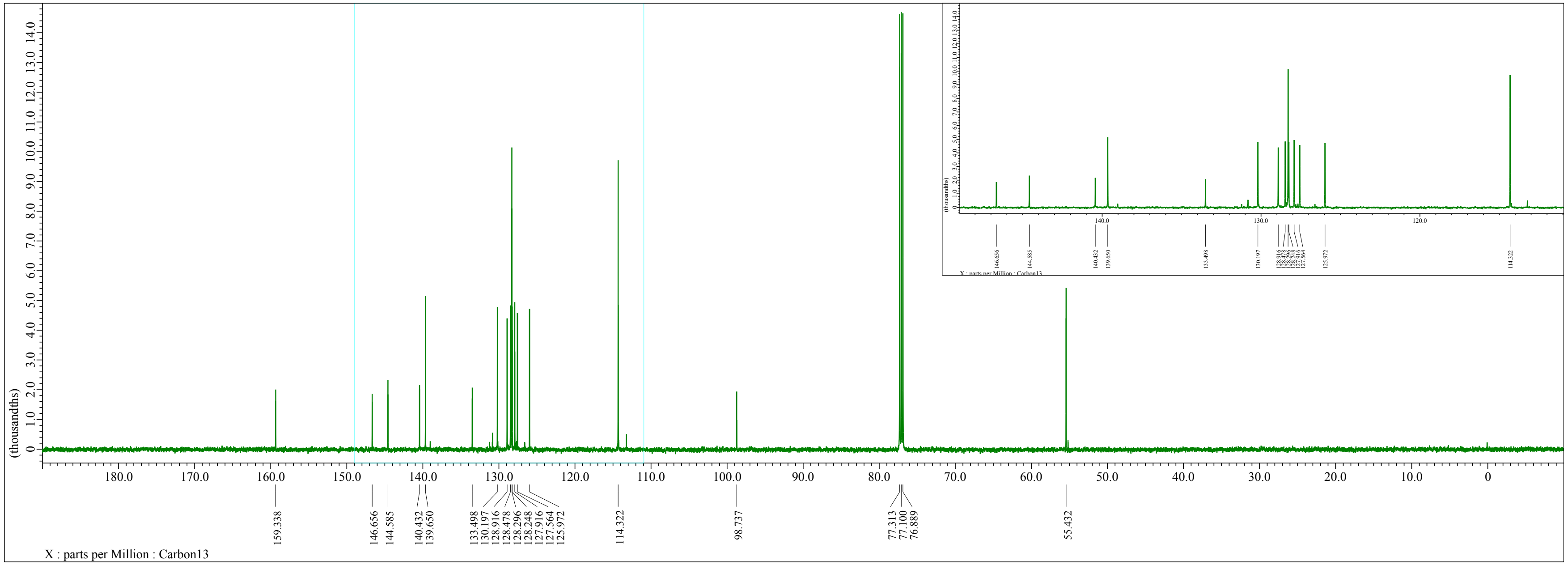

^{1}H NMR spectrum of **3ag** (400 MHz, $CDCl_3$).

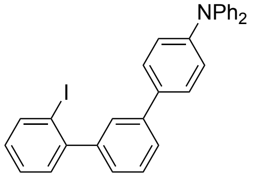

3ag
(*meta* only)

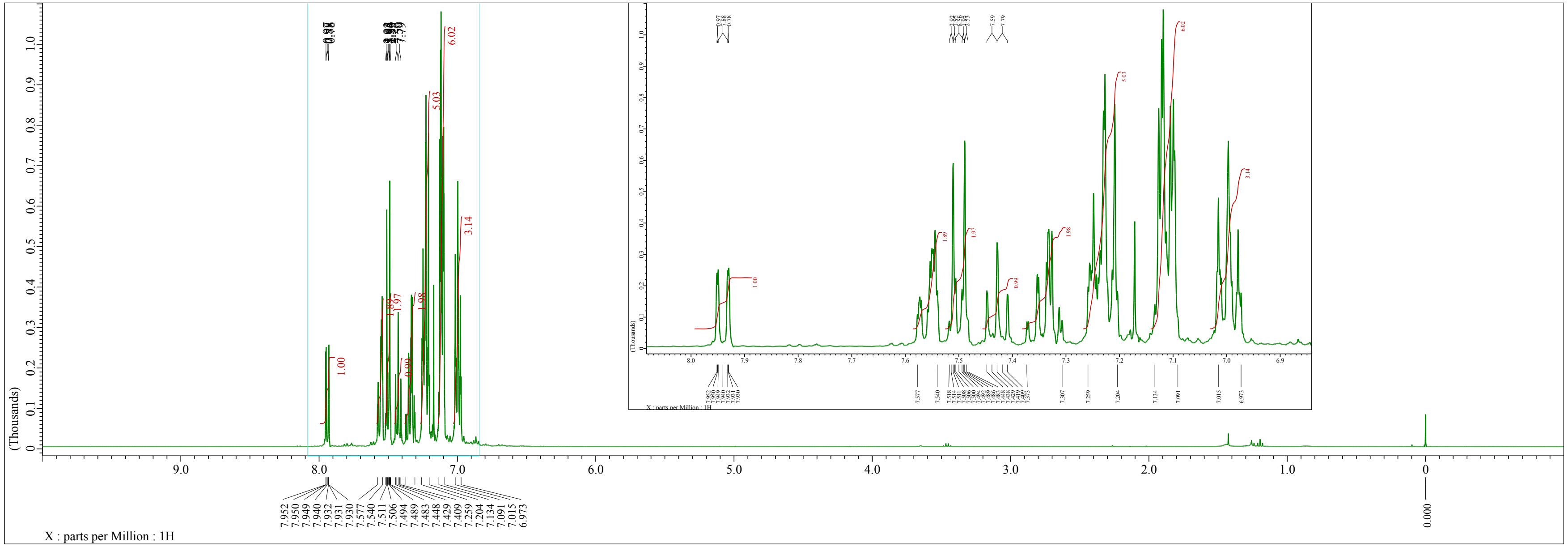

^{13}C NMR spectrum of **3ag** (151 MHz, $CDCl_3$).

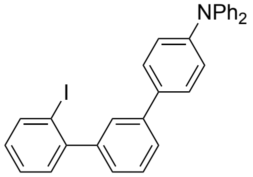

3ag
(*meta* only)

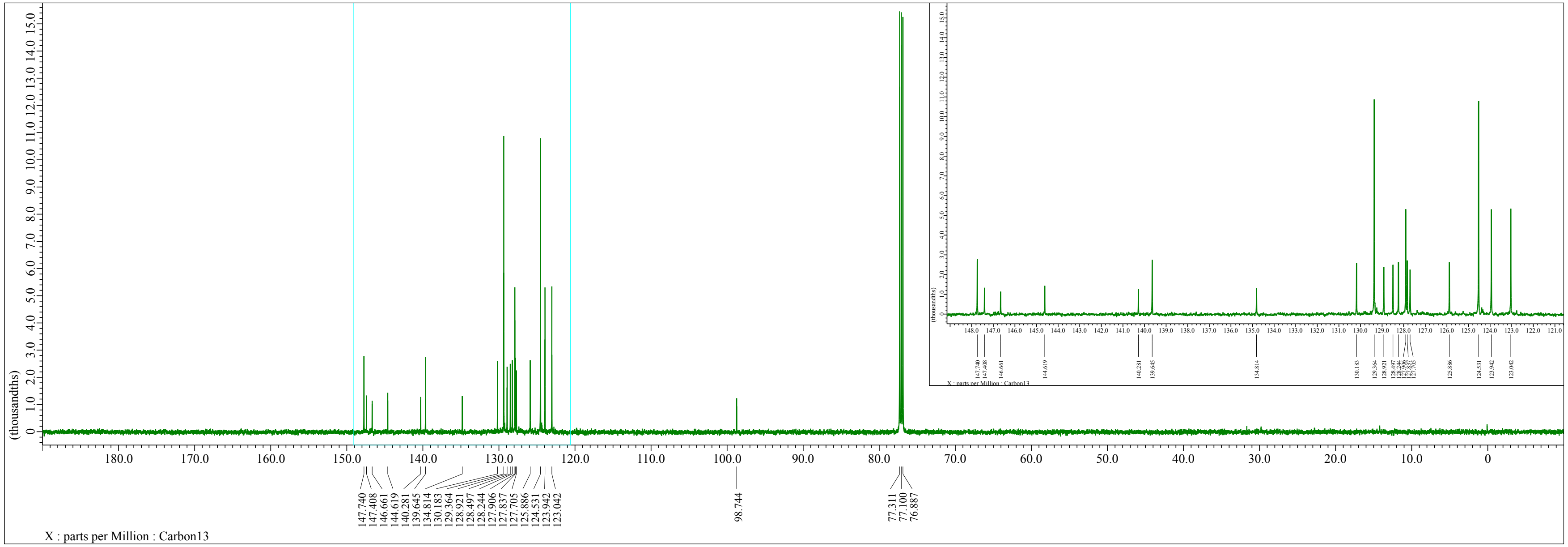

^{1}H NMR spectrum of **3ah** (600 MHz, $CDCl_3$).

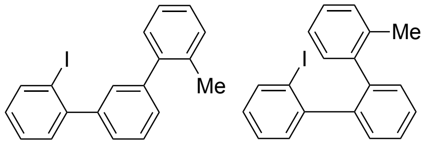

3ah
(*meta*:*ortho* = 97:3)

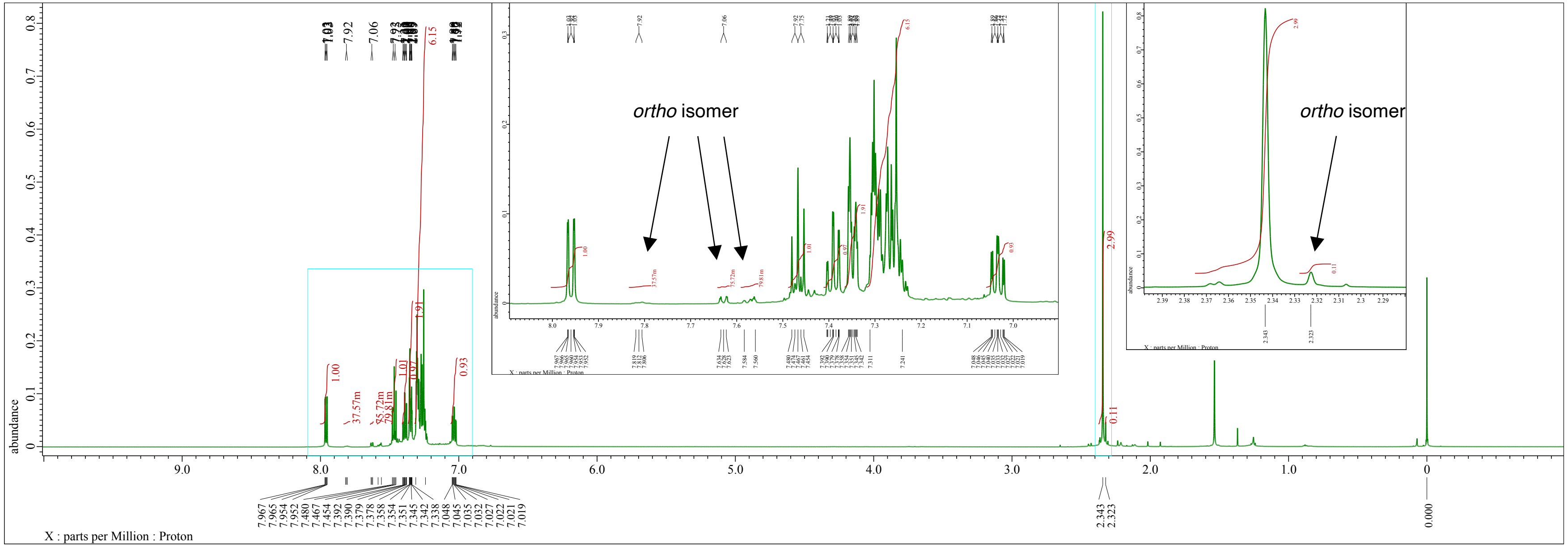

^{13}C NMR spectrum of **3ah** (151 MHz, $CDCl_3$).

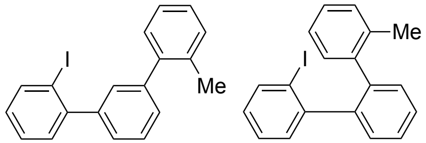

3ah
(*meta*:*ortho* = 97:3)

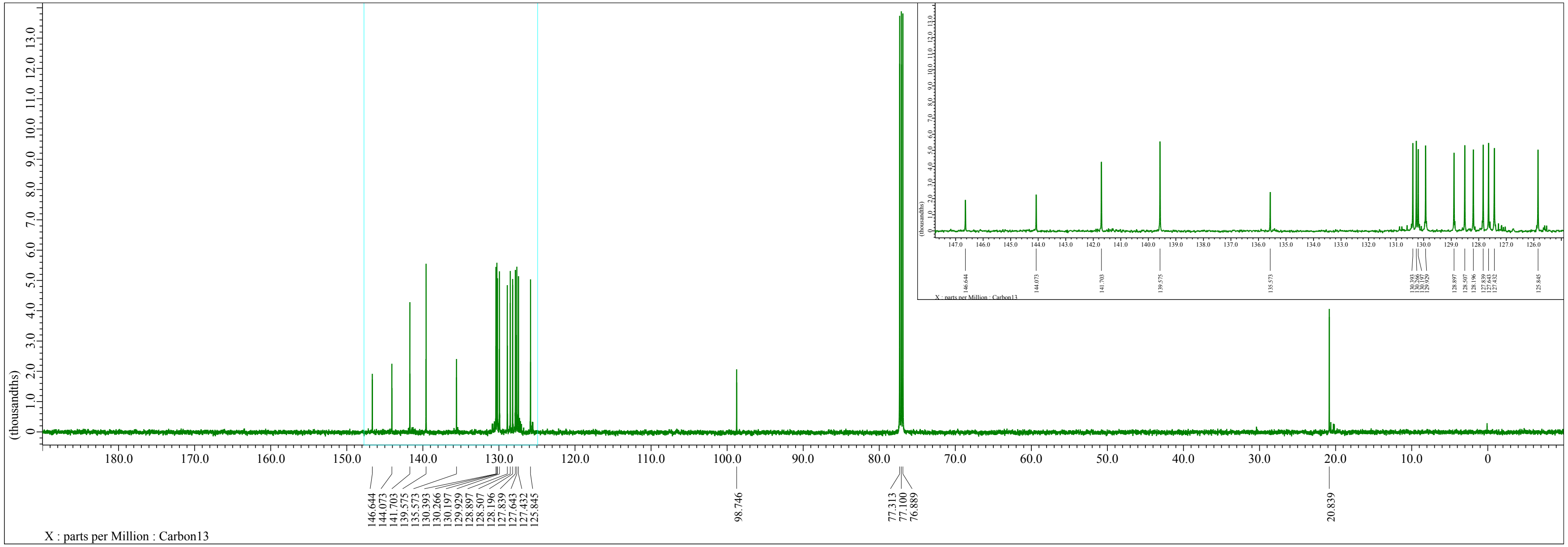

^{1}H NMR spectrum of **3ai** (400 MHz, $CDCl_3$).

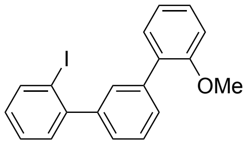

3ai
(*meta* only)

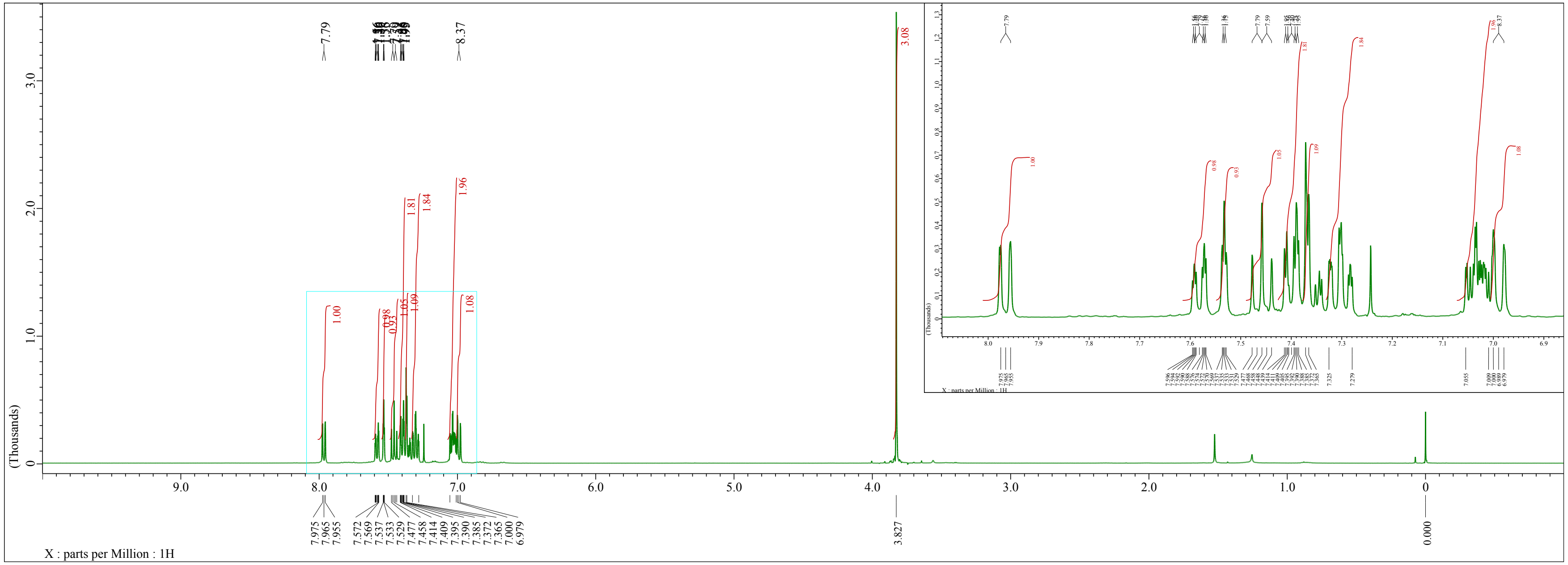

^{13}C NMR spectrum of **3ai** (151 MHz, $CDCl_3$).

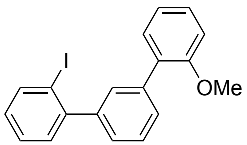

3ai
(*meta* only)

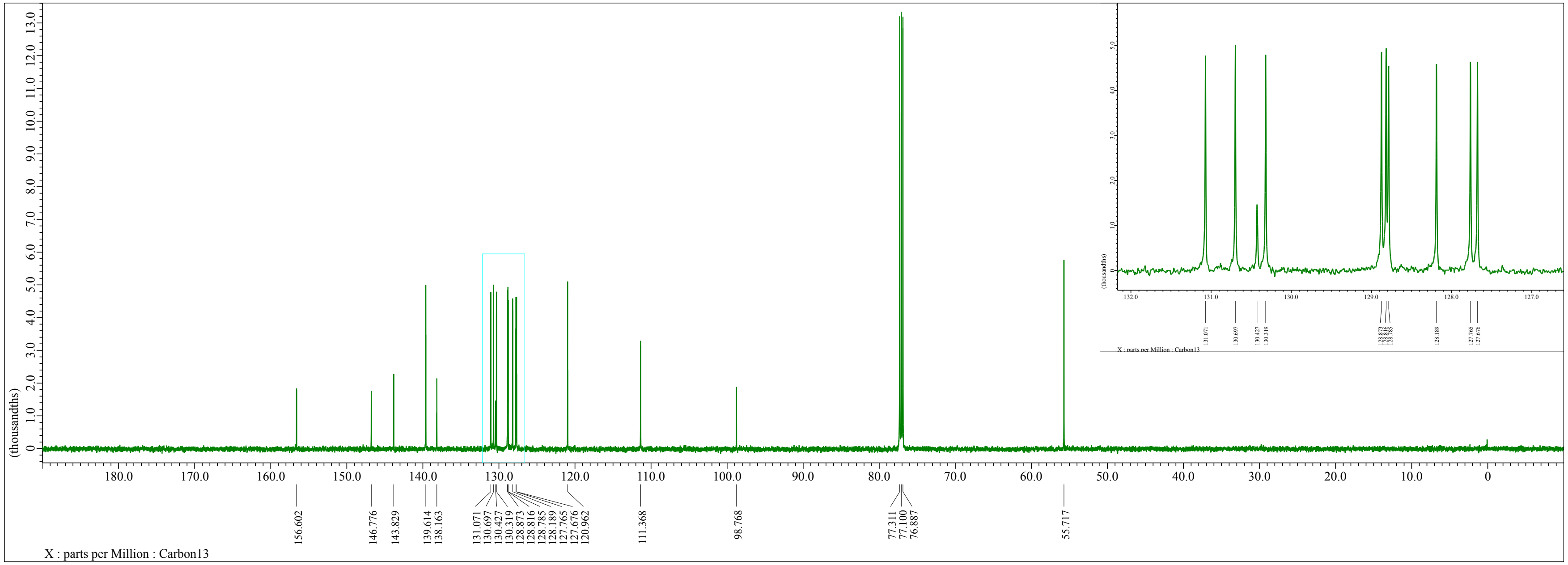

^{1}H NMR spectrum of **3aj** (600 MHz, $CDCl_3$).

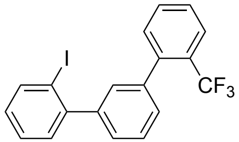

3aj
(*meta* only)

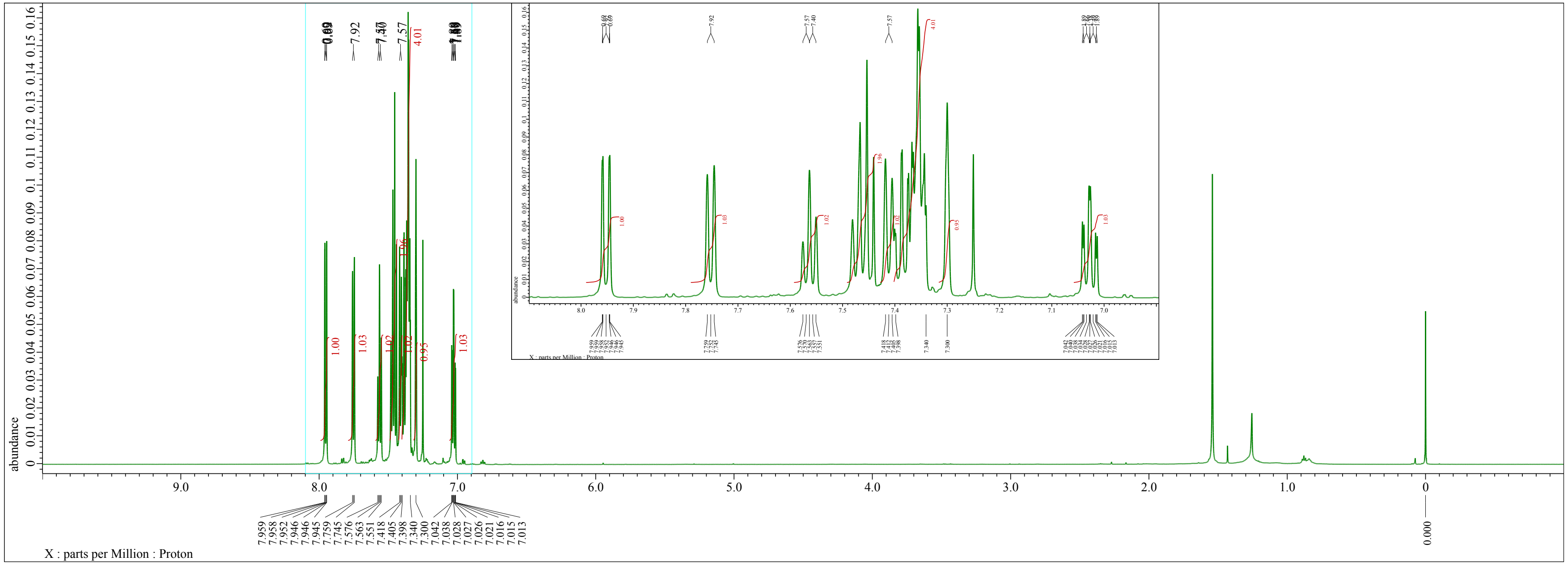

^{19}F NMR spectrum of **3aj** (376 MHz, $CDCl_3$).

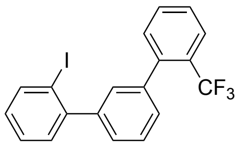

3aj
(*meta* only)

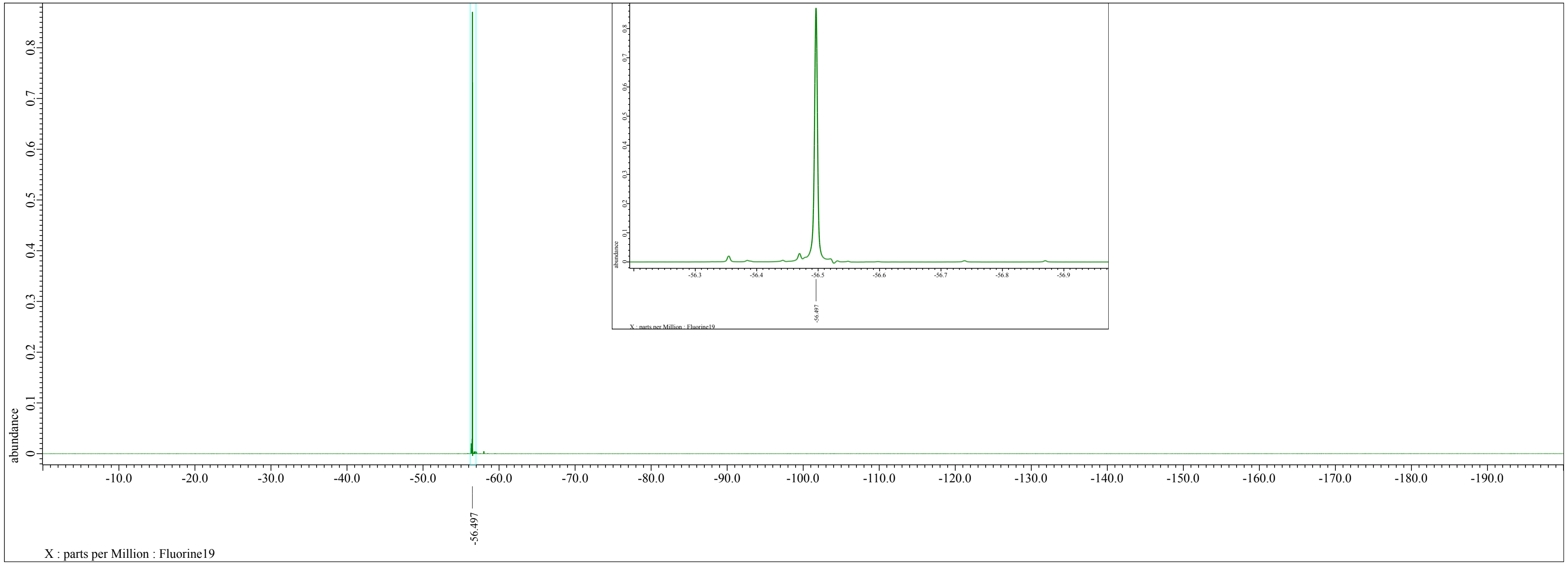

^{13}C NMR spectrum of **3aj** (151 MHz, $CDCl_3$).

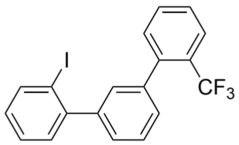

3aj
(*meta* only)

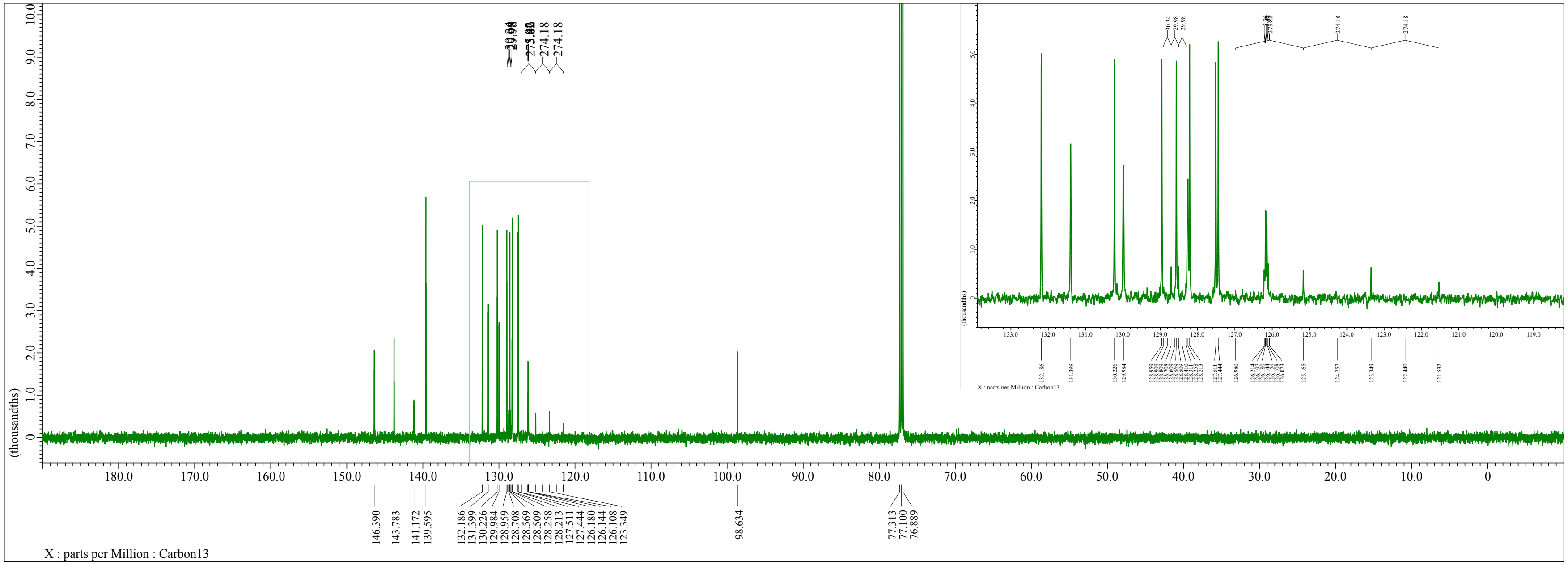

^{1}H NMR spectrum of **3ak** (400 MHz, $CDCl_3$).

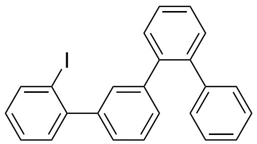

3ak
(*meta* only)

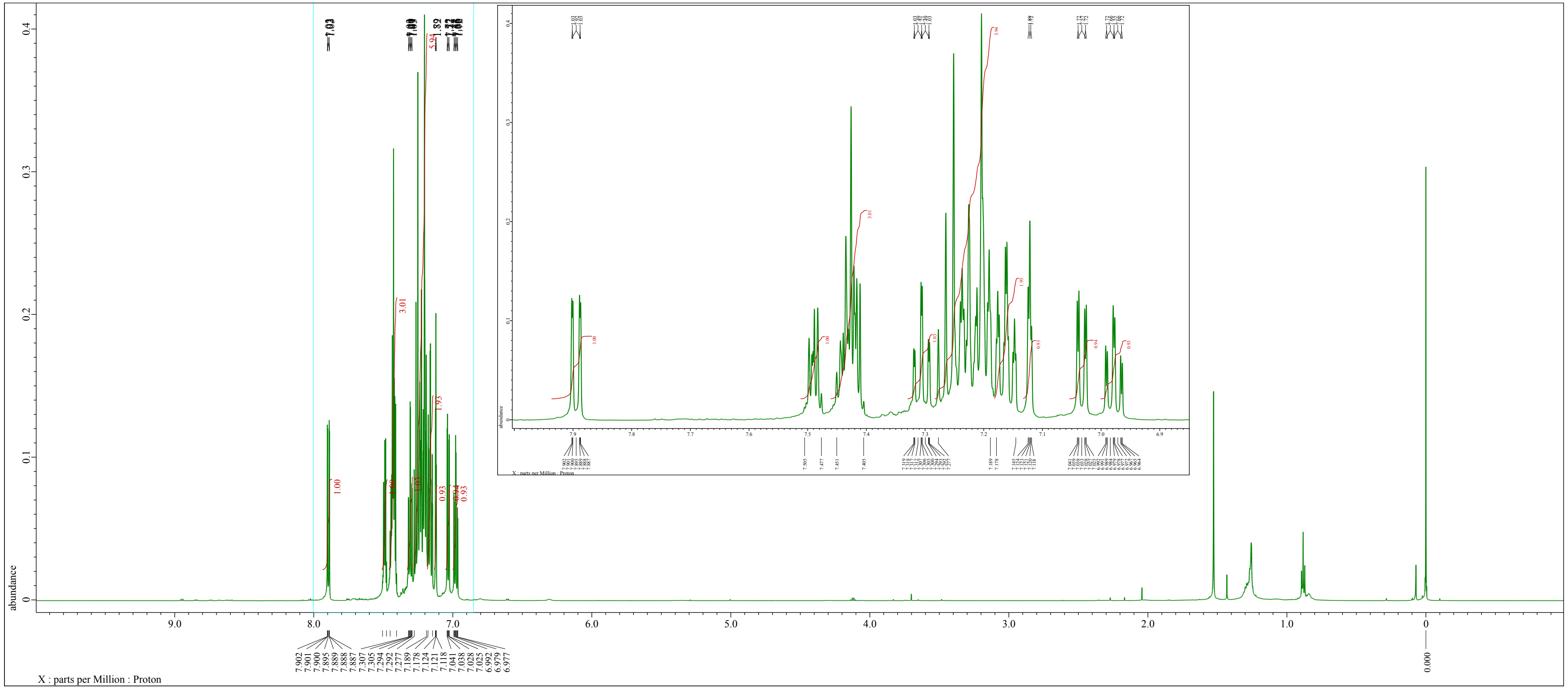

^{13}C NMR spectrum of **3ak** (151 MHz, $CDCl_3$).

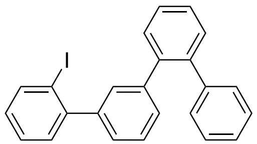

3ak
(*meta* only)

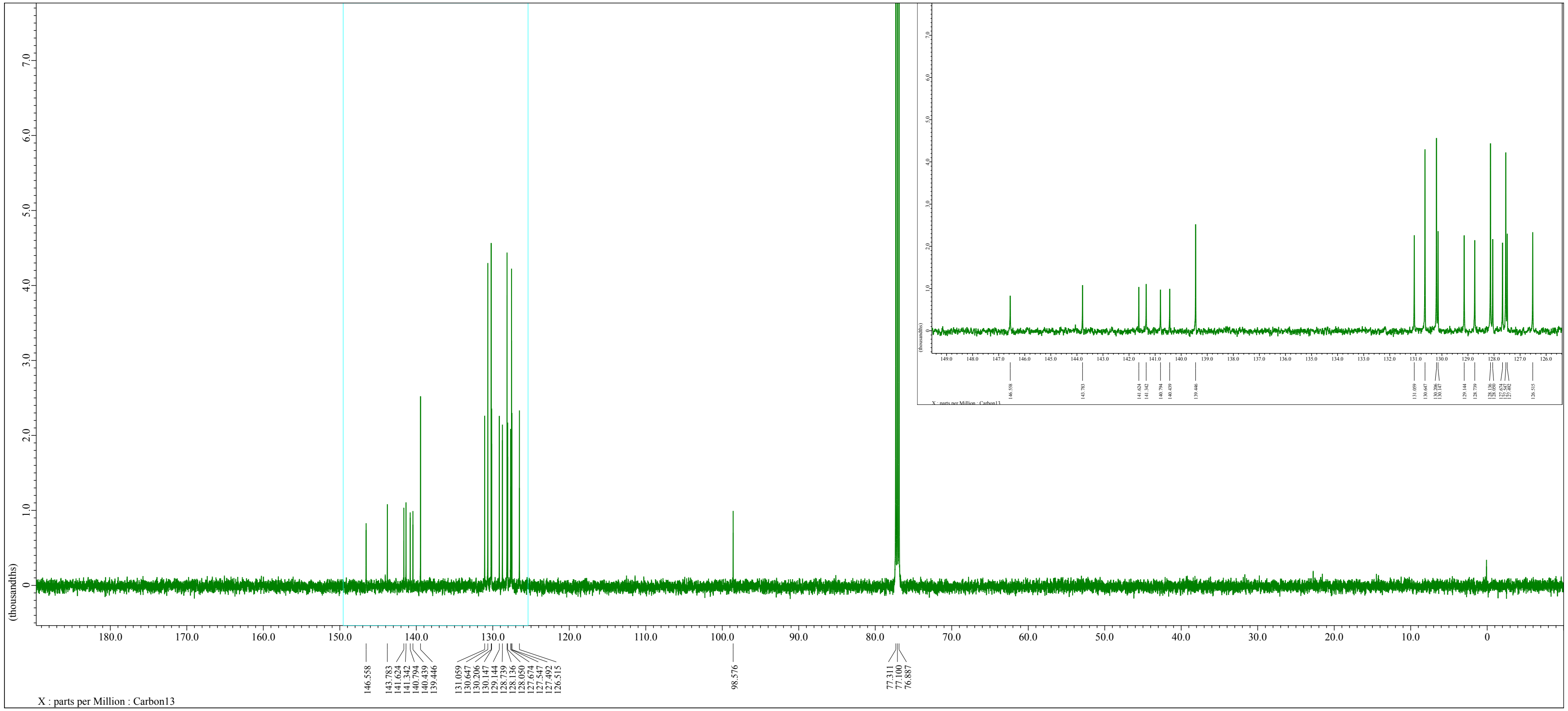

^{1}H NMR spectrum of **3al** (600 MHz, $CDCl_3$).

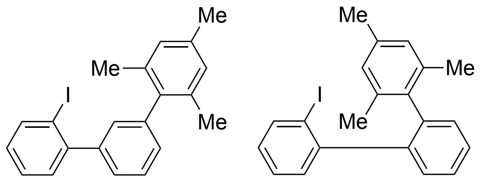

3al
(*meta*:*ortho* = 93:7)

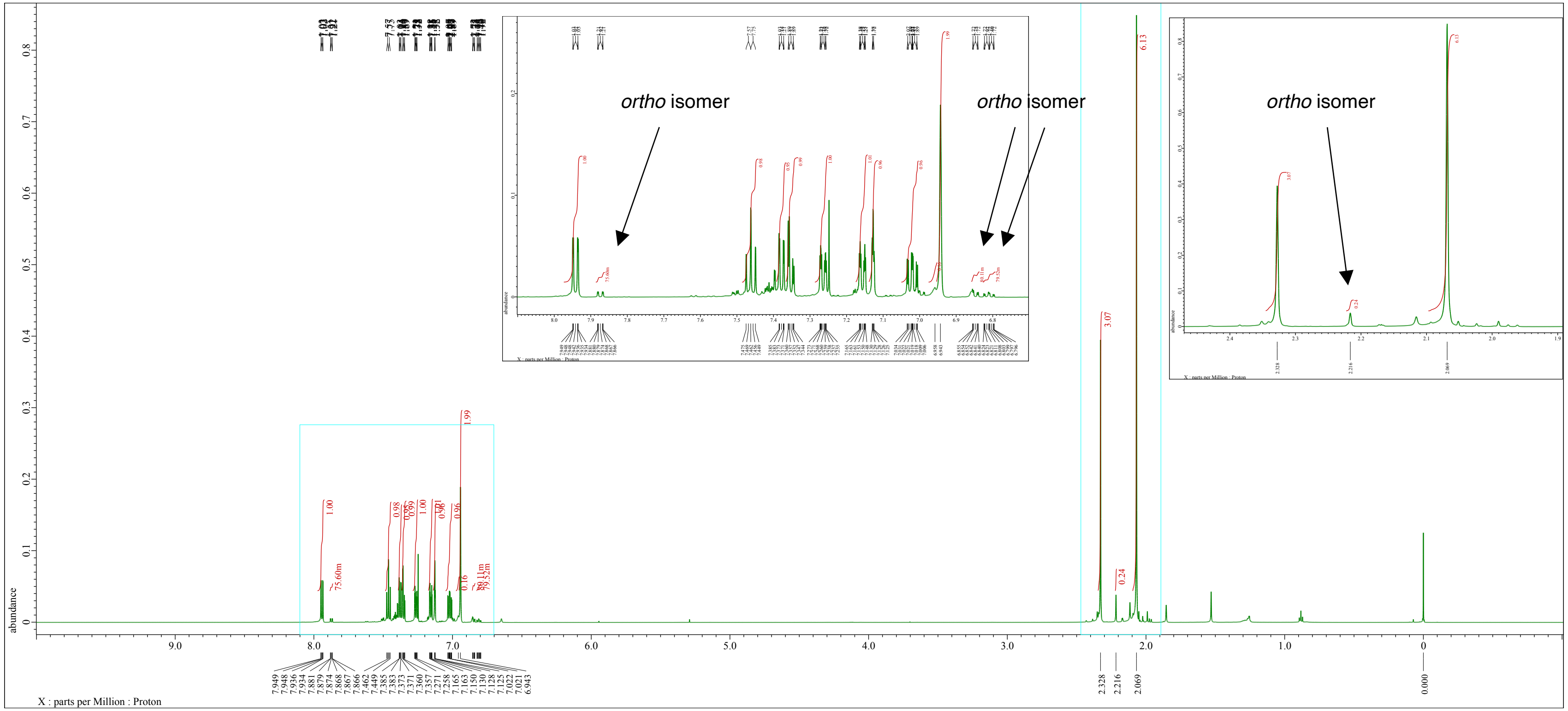

^{13}C NMR spectrum of **3al** (151 MHz, $CDCl_3$).

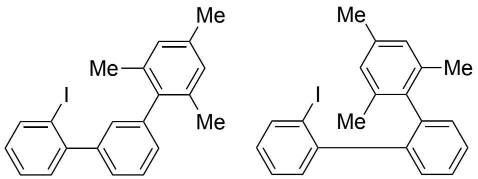

3al
(*meta*:*ortho* = 93:7)

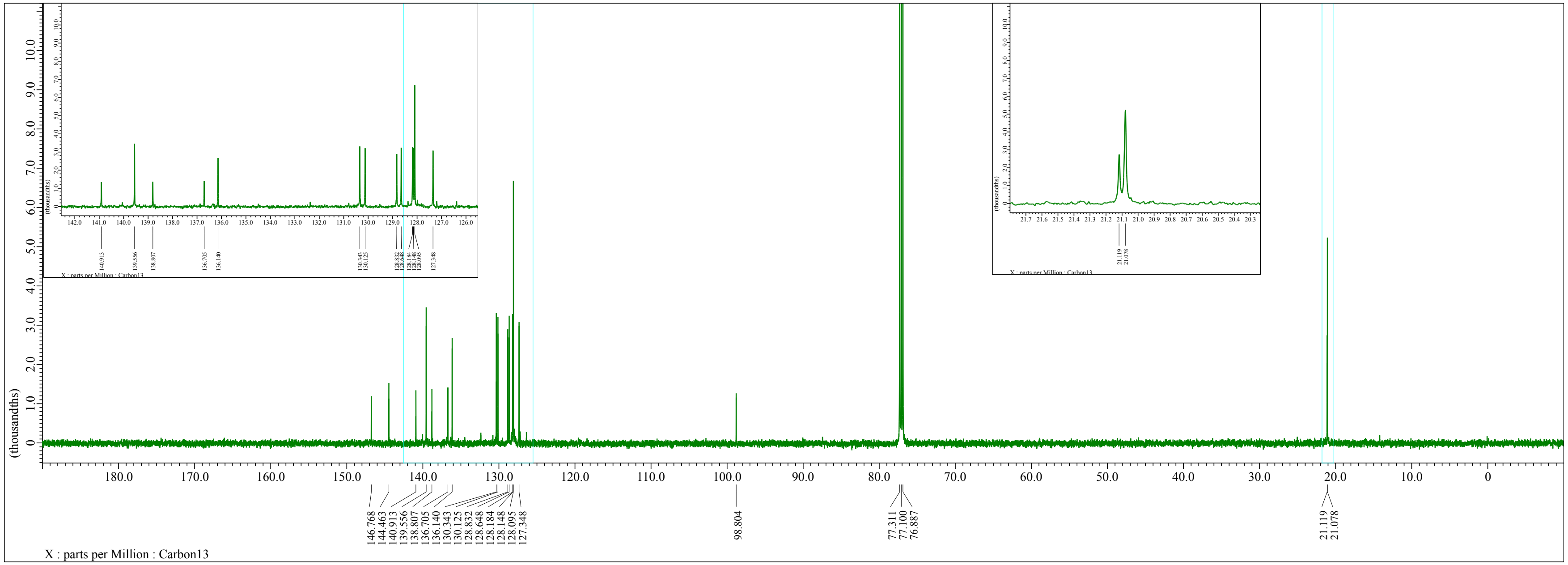

^{1}H NMR spectrum of **3am** (400 MHz, $CDCl_3$).

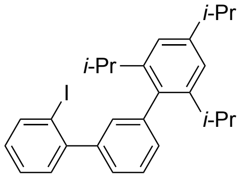

3am
(*meta* only)

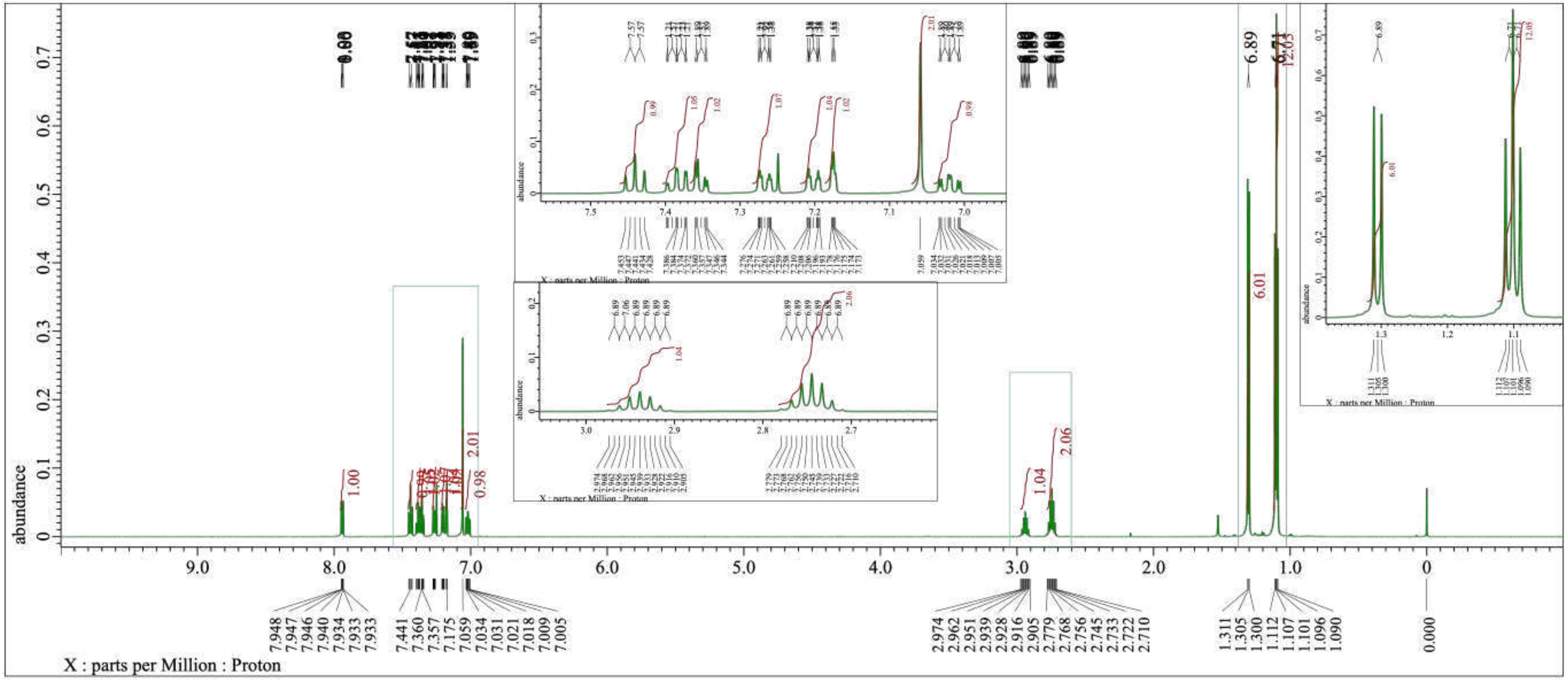

^{13}C NMR spectrum of **3am** (151 MHz, $CDCl_3$).

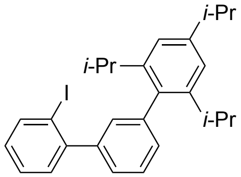

3am
(*meta* only)

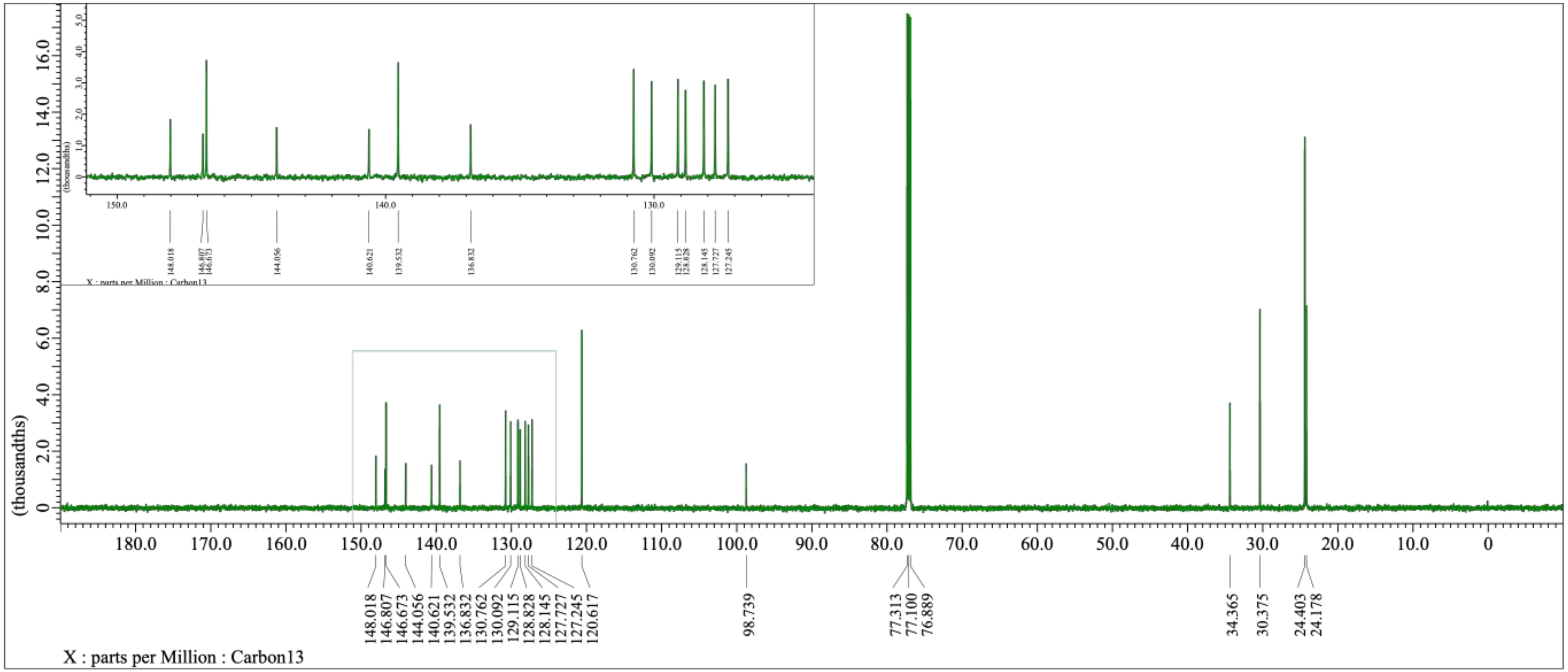

^{1}H NMR spectrum of **3an** (400 MHz, $CDCl_3$).

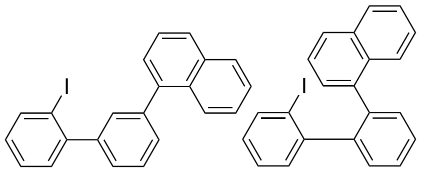

3an
(*meta*:*ortho* = 93:7)

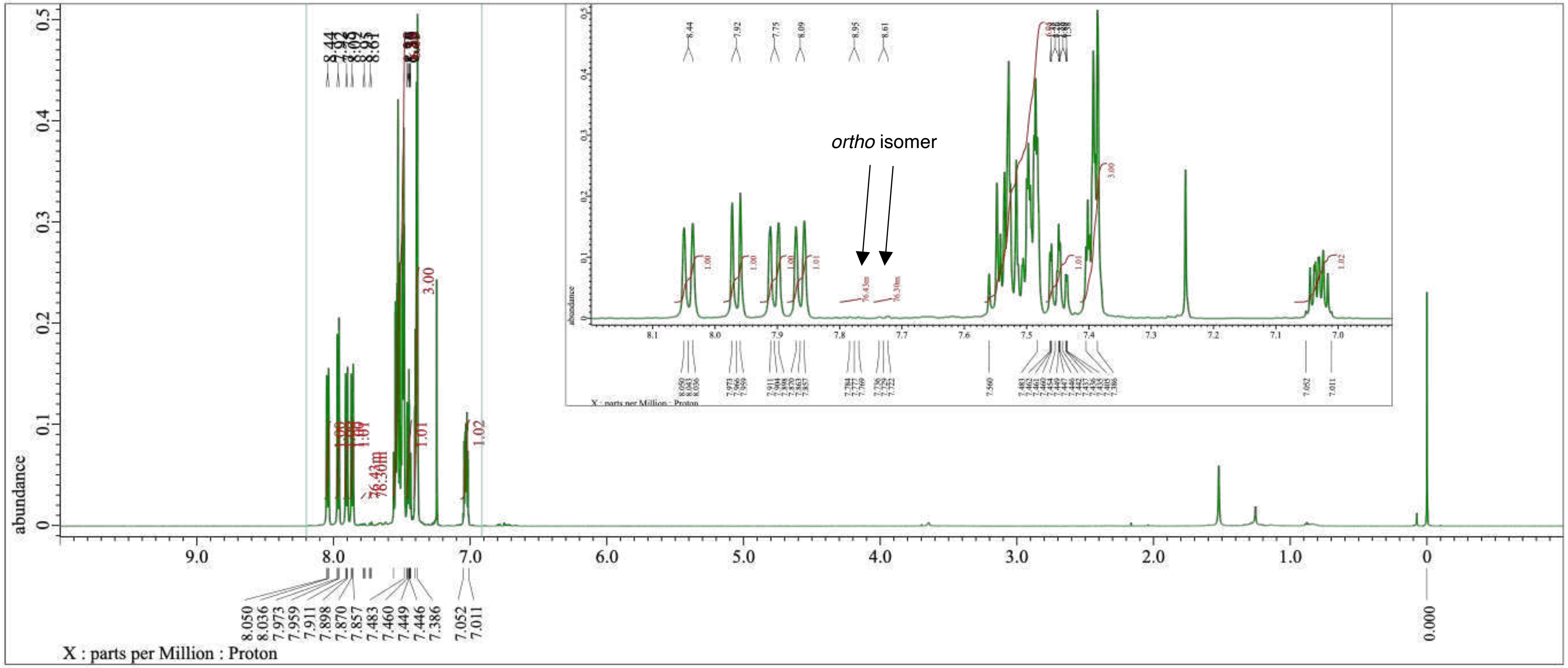

^{13}C NMR spectrum of **3an** (151 MHz, $CDCl_3$).

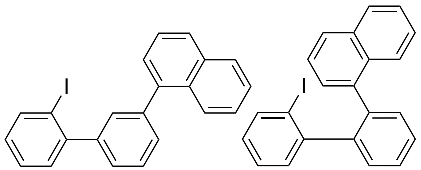

3an
(*meta*:*ortho* = 93:7)

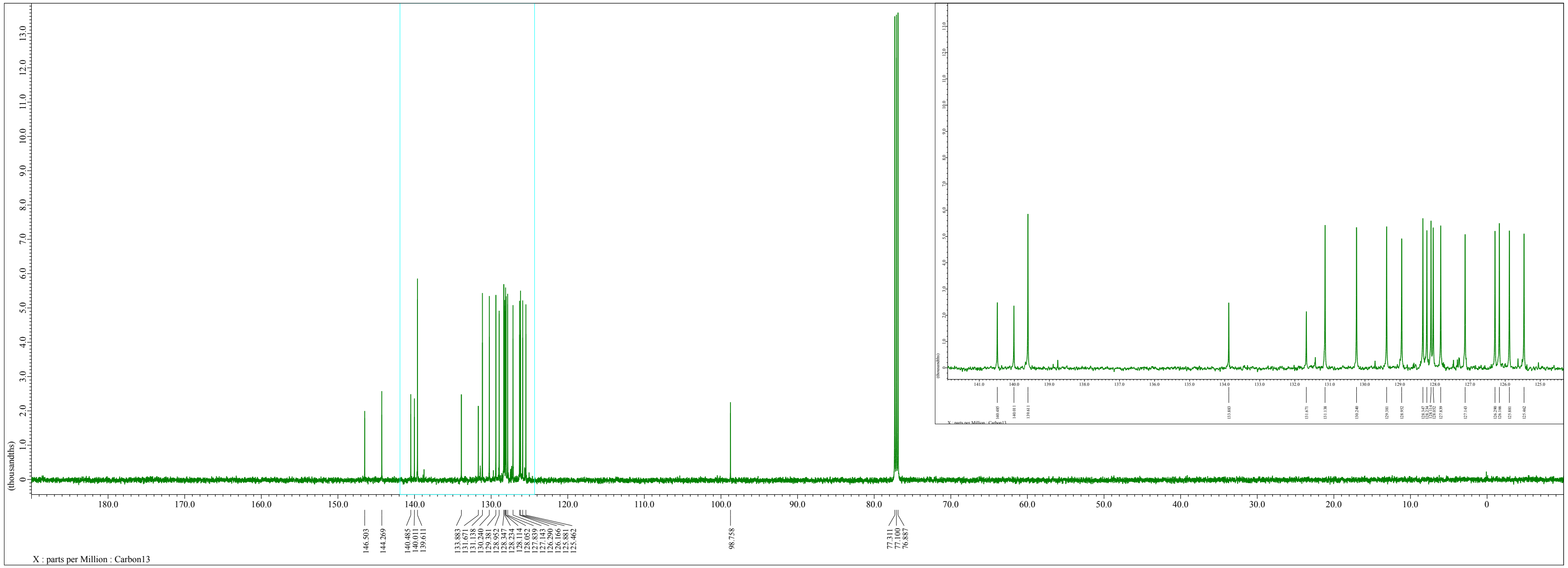

^{1}H NMR spectrum of **3ao** (600 MHz, $CDCl_3$).

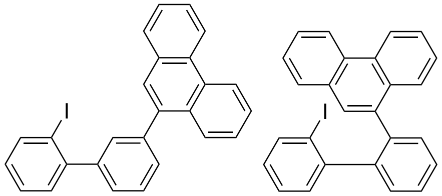

3ao
(*meta*:*ortho* = 96:4)

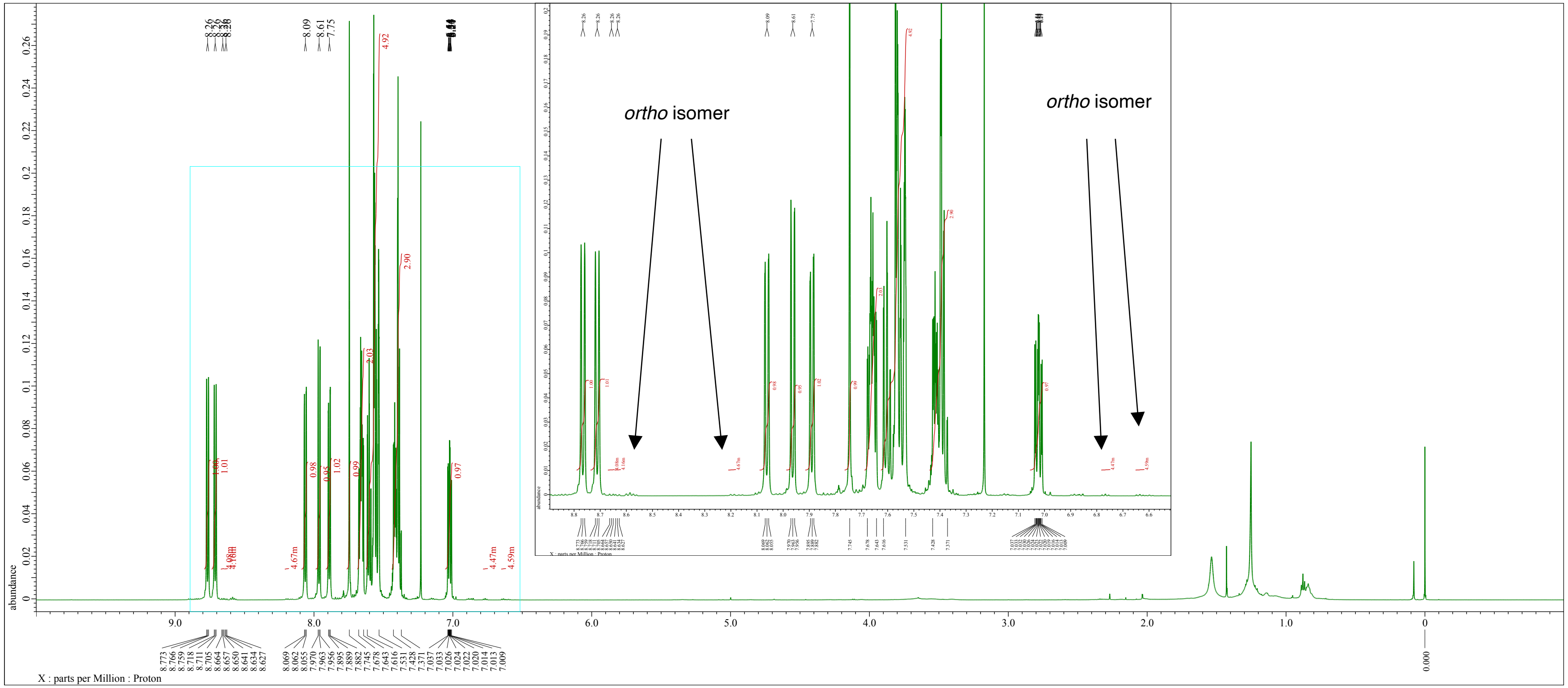

^{13}C NMR spectrum of **3ao** (151 MHz, $CDCl_3$).

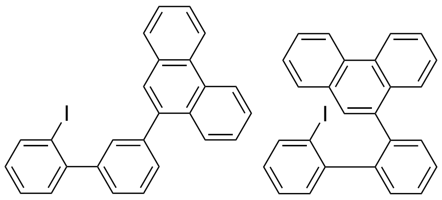

3ao
(*meta*:*ortho* = 96:4)

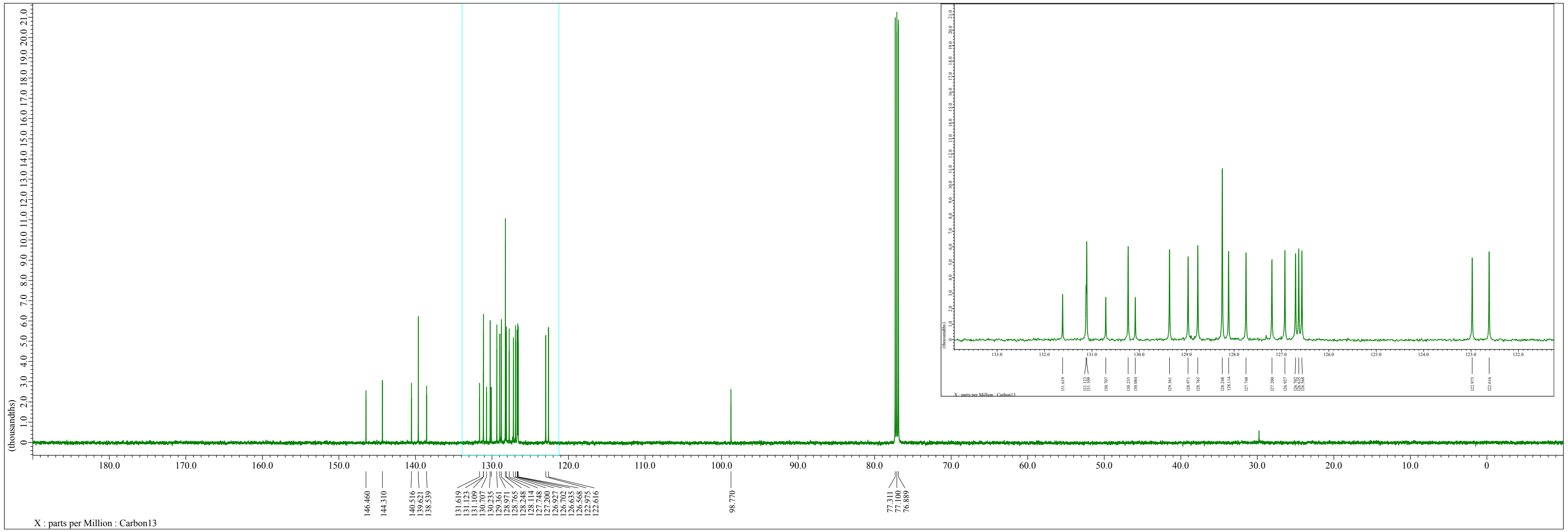

^{1}H NMR spectrum of **3ap** (600 MHz, $CDCl_3$).

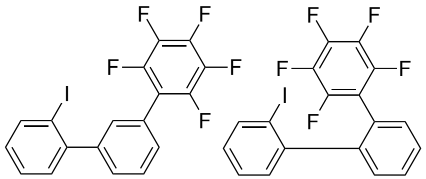

3ap
(*meta*:*ortho* = 75:25)

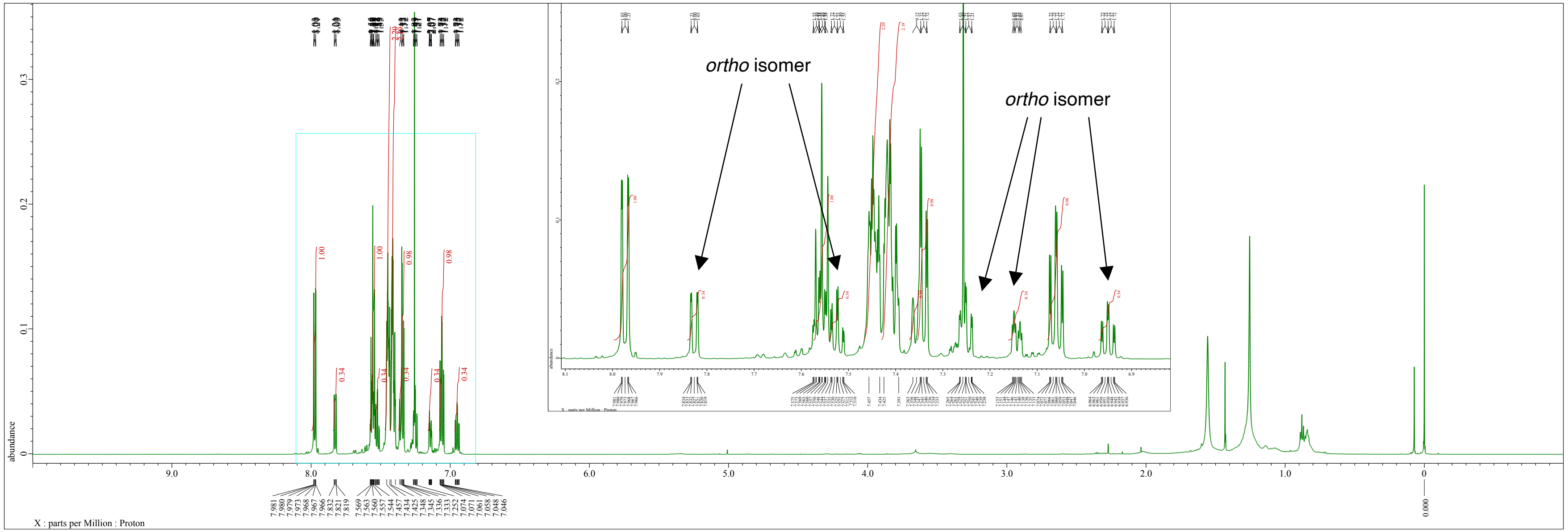

^{19}F NMR spectrum of **3ap** (376 MHz, $CDCl_3$).

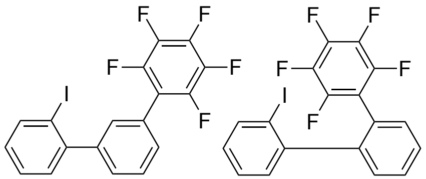

3ap
(*meta*:*ortho* = 75:25)

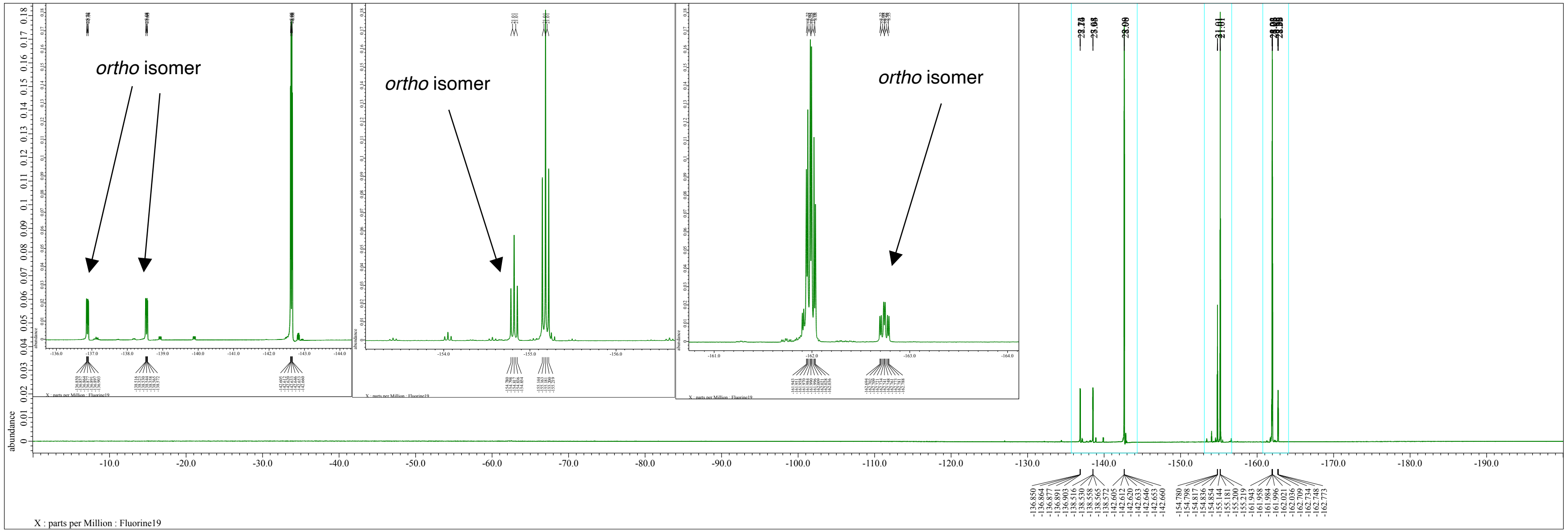

^{13}C NMR spectrum of **3ap** (151 MHz, $CDCl_3$).

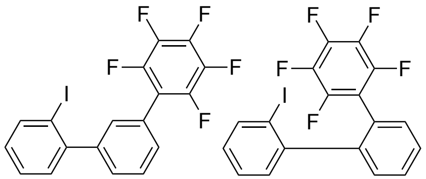

3ap
(*meta*:*ortho* = 75:25)

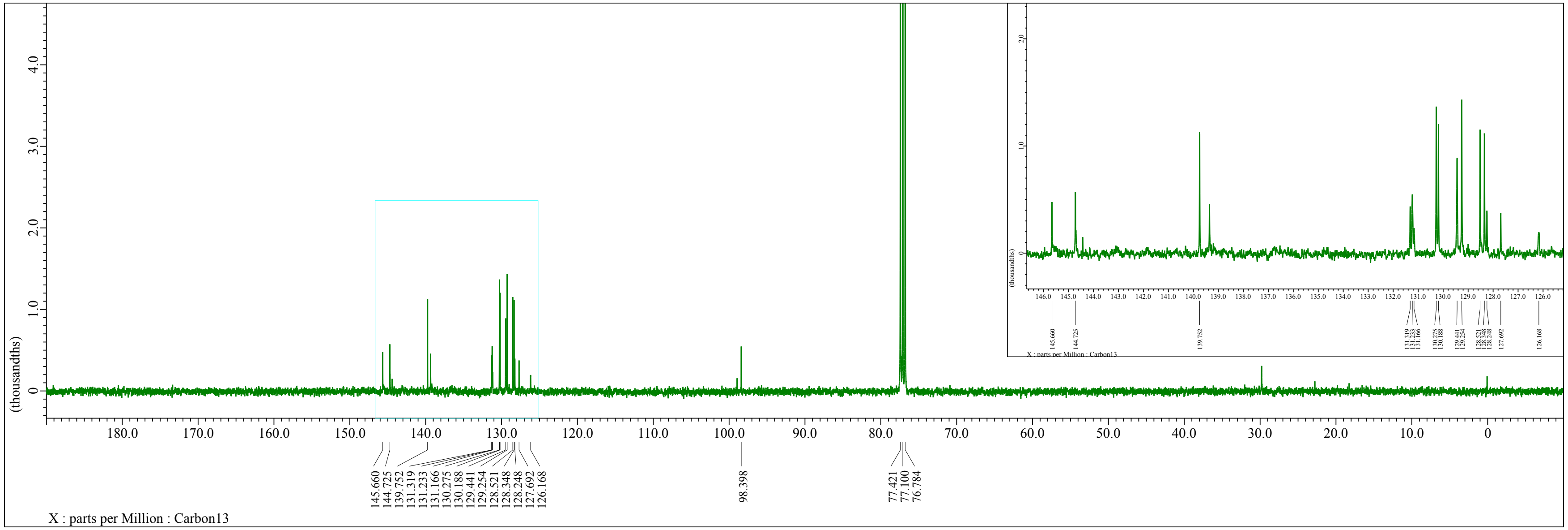

^{1}H NMR spectrum of **3aq** (600 MHz, $CDCl_3$).

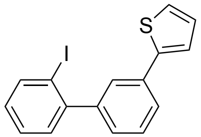

3aq
(*meta* only)

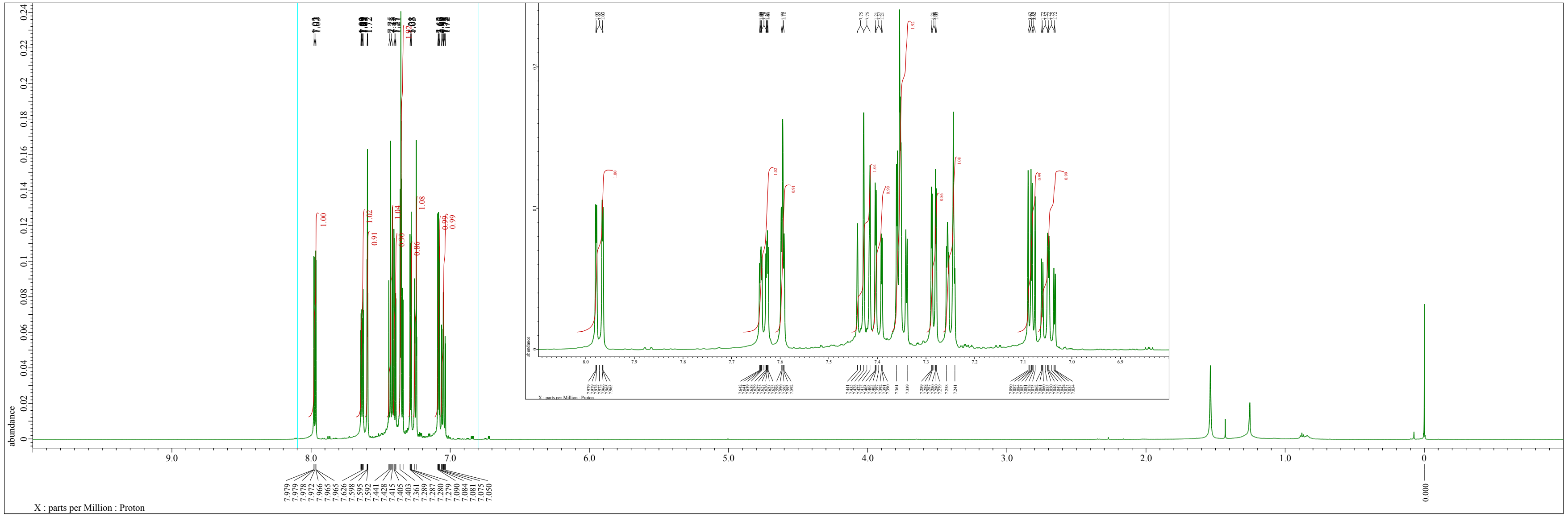

^{13}C NMR spectrum of **3aq** (151 MHz, $CDCl_3$).

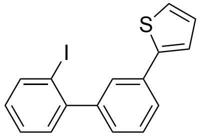

3aq
(*meta* only)

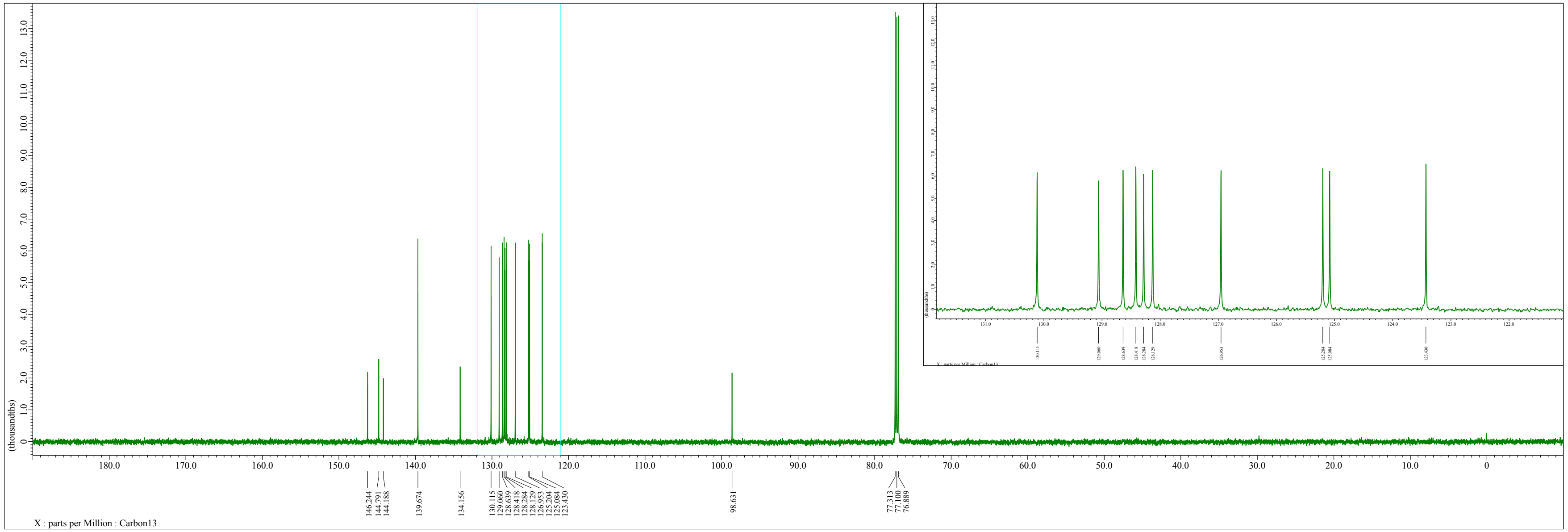

^{1}H NMR spectrum of **3ar** (400 MHz, $CDCl_3$).

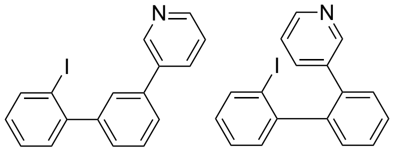

3ar
(*meta*:*ortho* = 95:5)

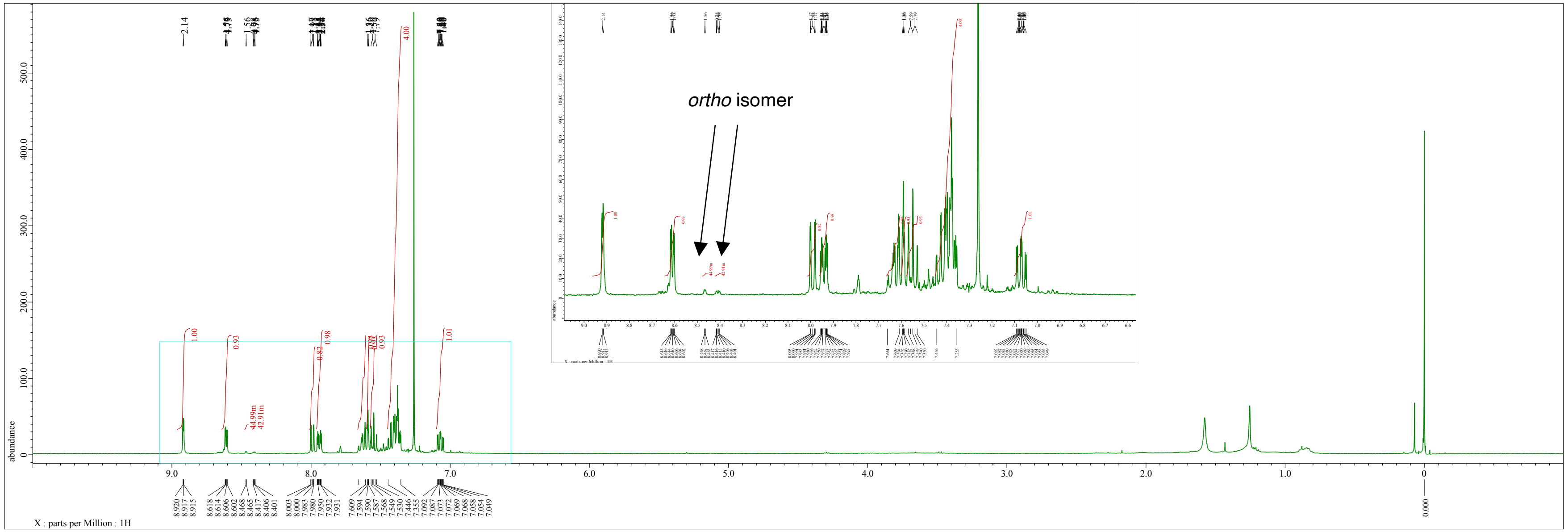

^{13}C NMR spectrum of **3ar** (151 MHz, $CDCl_3$).

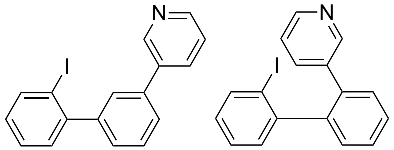

3ar
(*meta*:*ortho* = 95:5)

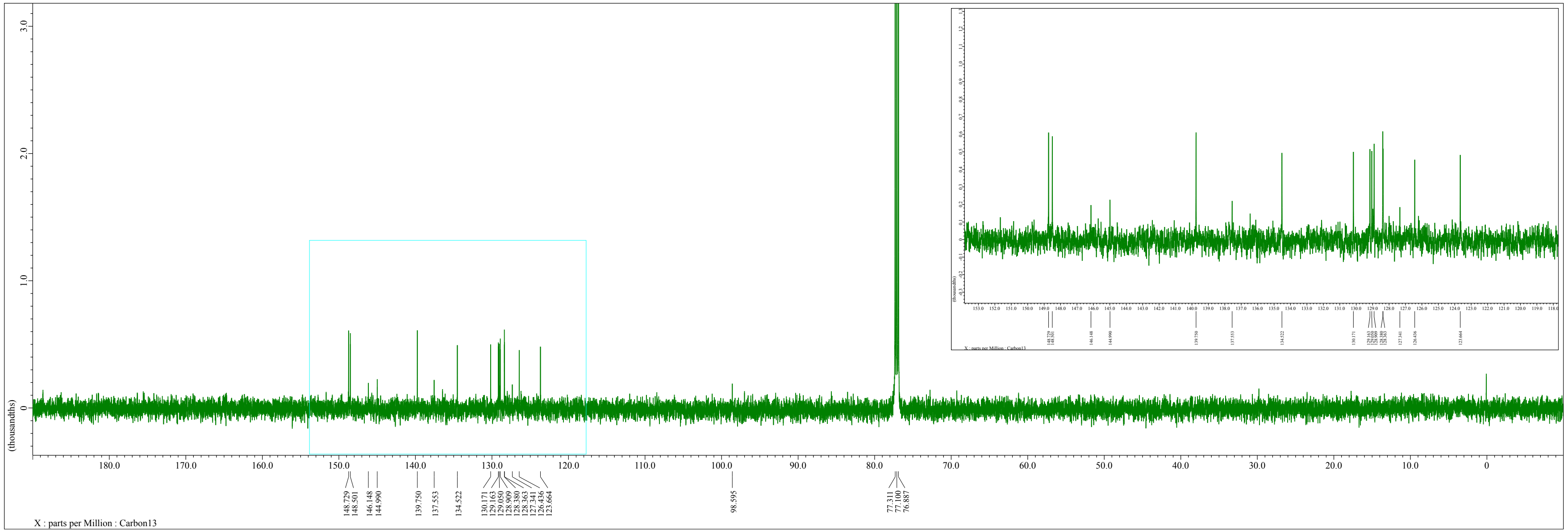

^{1}H NMR spectrum of **3as** (600 MHz, $CDCl_3$).

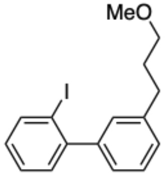

3as
(*meta* only)

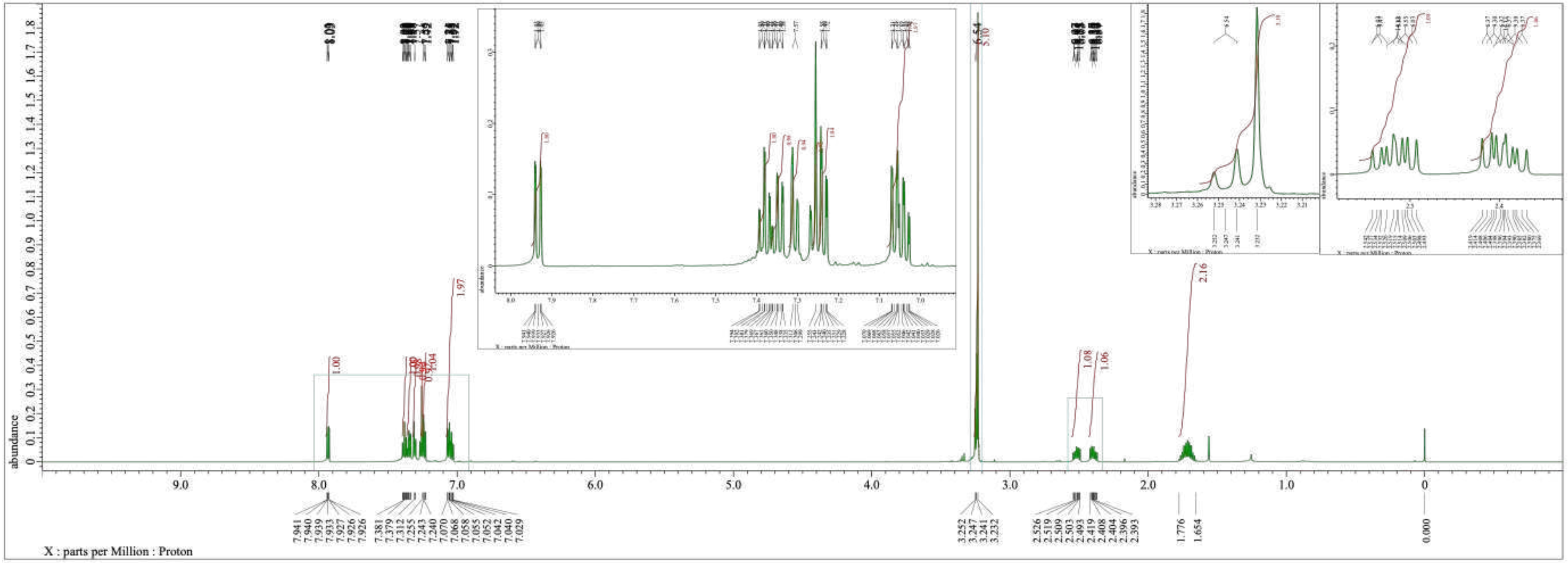

^{13}C NMR spectrum of **3as** (151 MHz, $CDCl_3$).

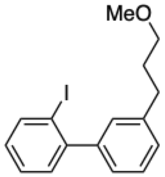

3as
(*meta* only)

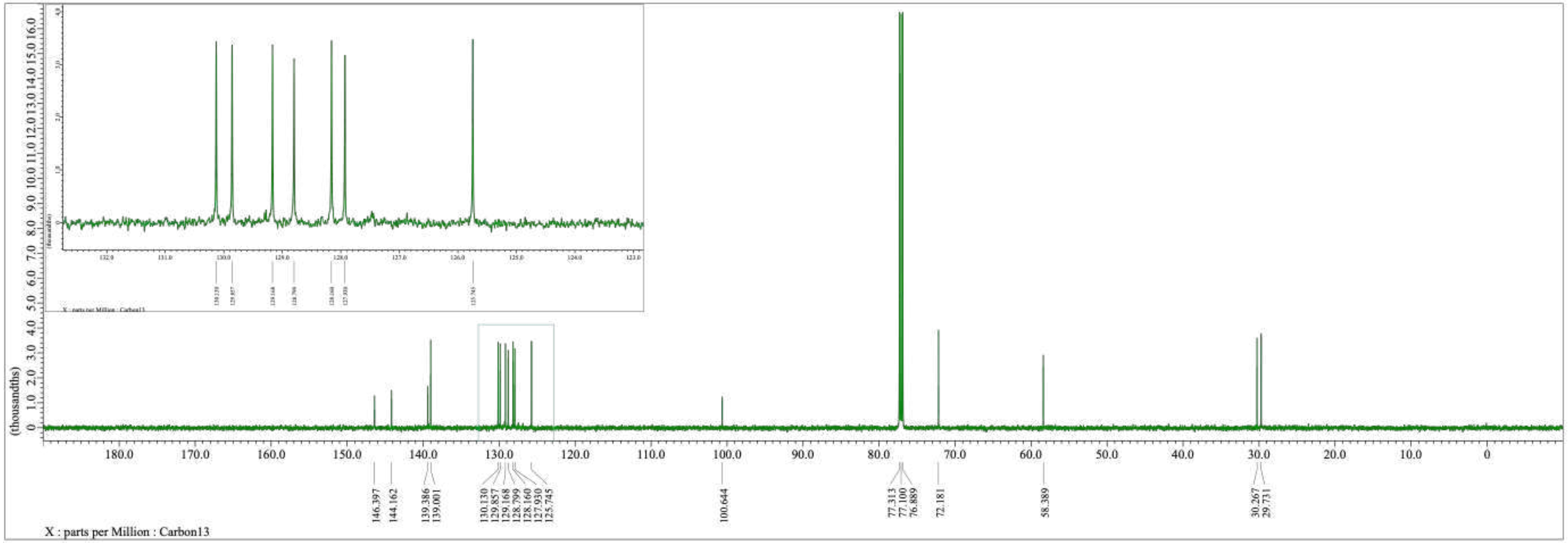

^{1}H NMR spectrum of **3at** (400 MHz, $CDCl_3$).

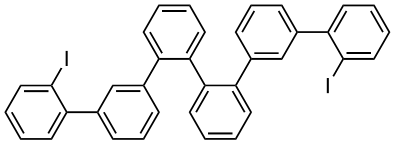

3at
(*meta* only)

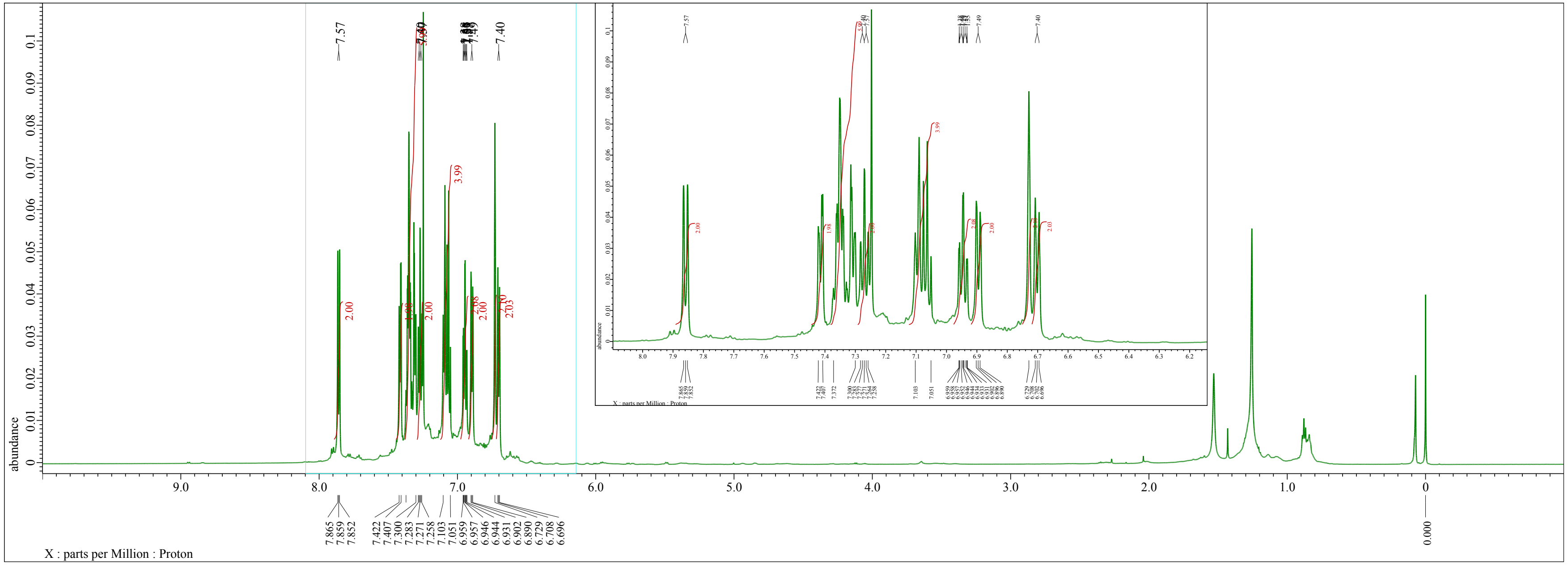

^{13}C NMR spectrum of **3at** (151 MHz, $CDCl_3$).

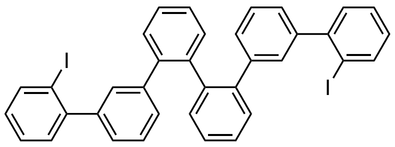

3at
(*meta* only)

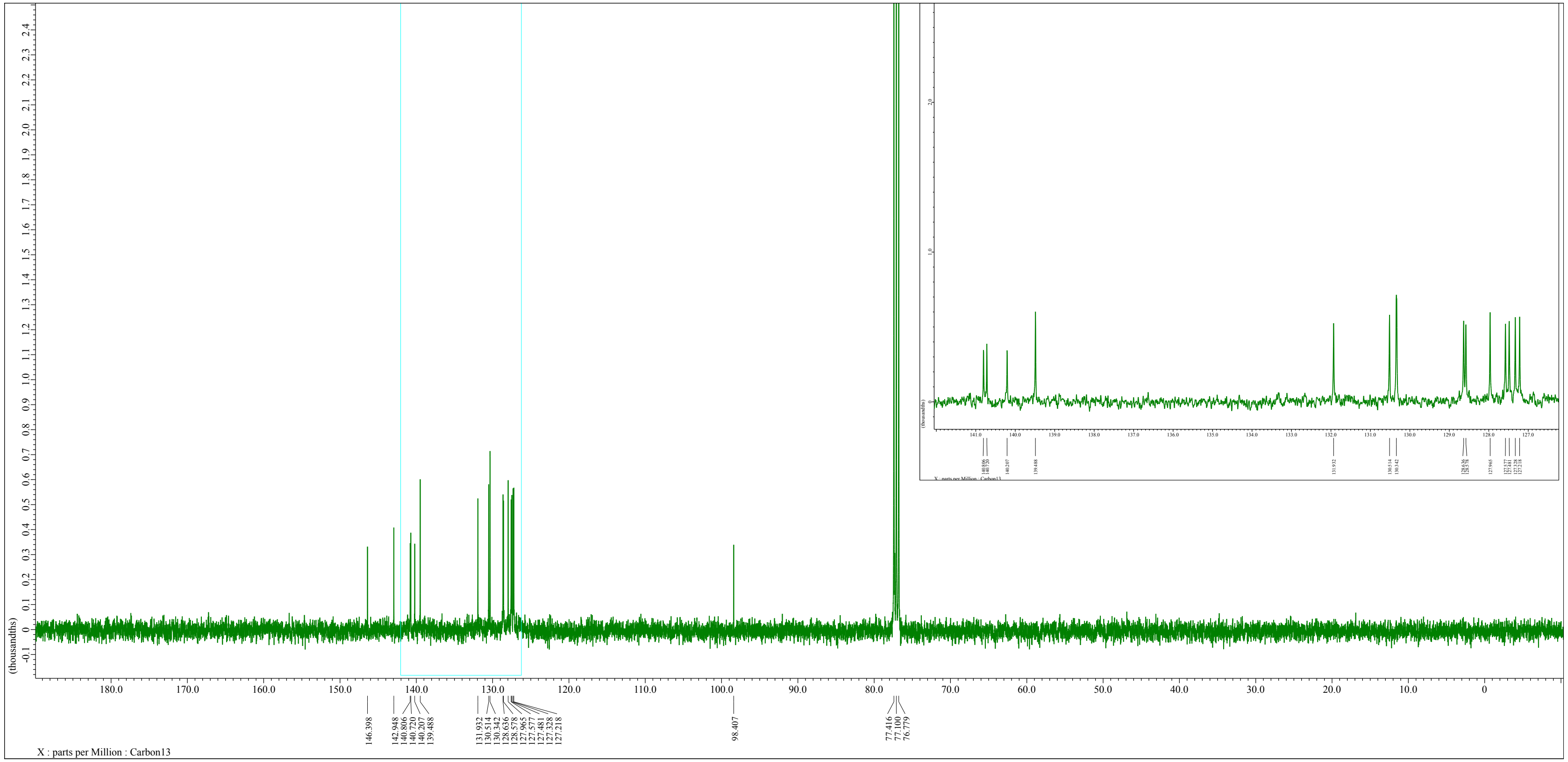

^{1}H NMR spectrum of **3cq** (600 MHz, $CDCl_3$).

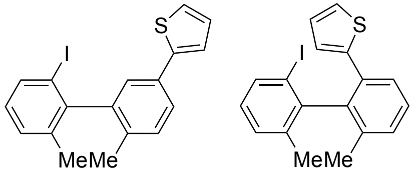

3cq
(*meta*:*ortho* = 85:15)

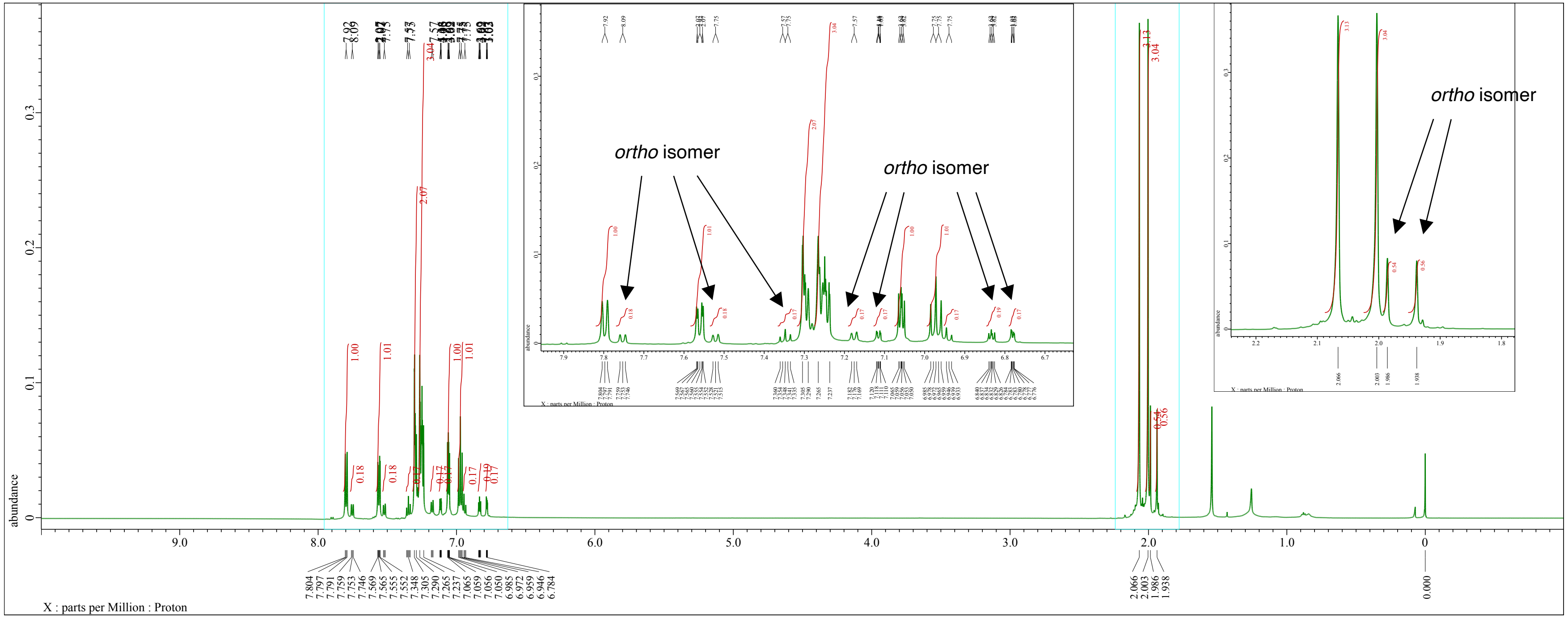

^{13}C NMR spectrum of **3cq** (151 MHz, $CDCl_3$).

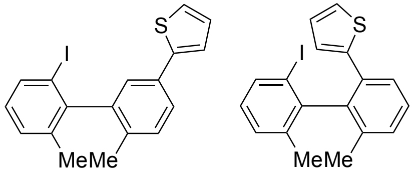

3cq
(*meta*:*ortho* = 85:15)

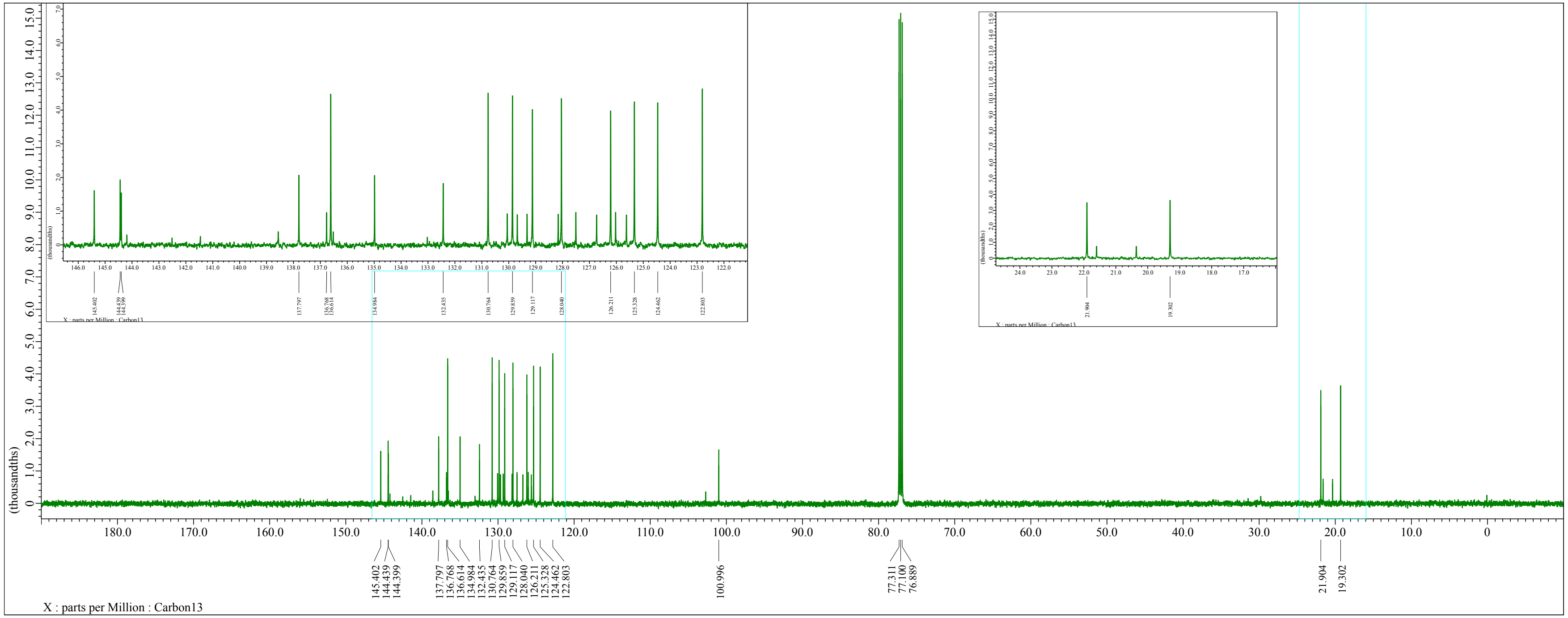

^{1}H NMR spectrum of **3cf** (400 MHz, $CDCl_3$).

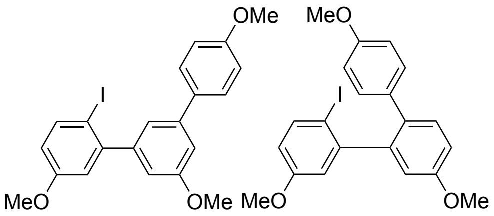

3cf
(*meta*:*ortho* = 90:10)

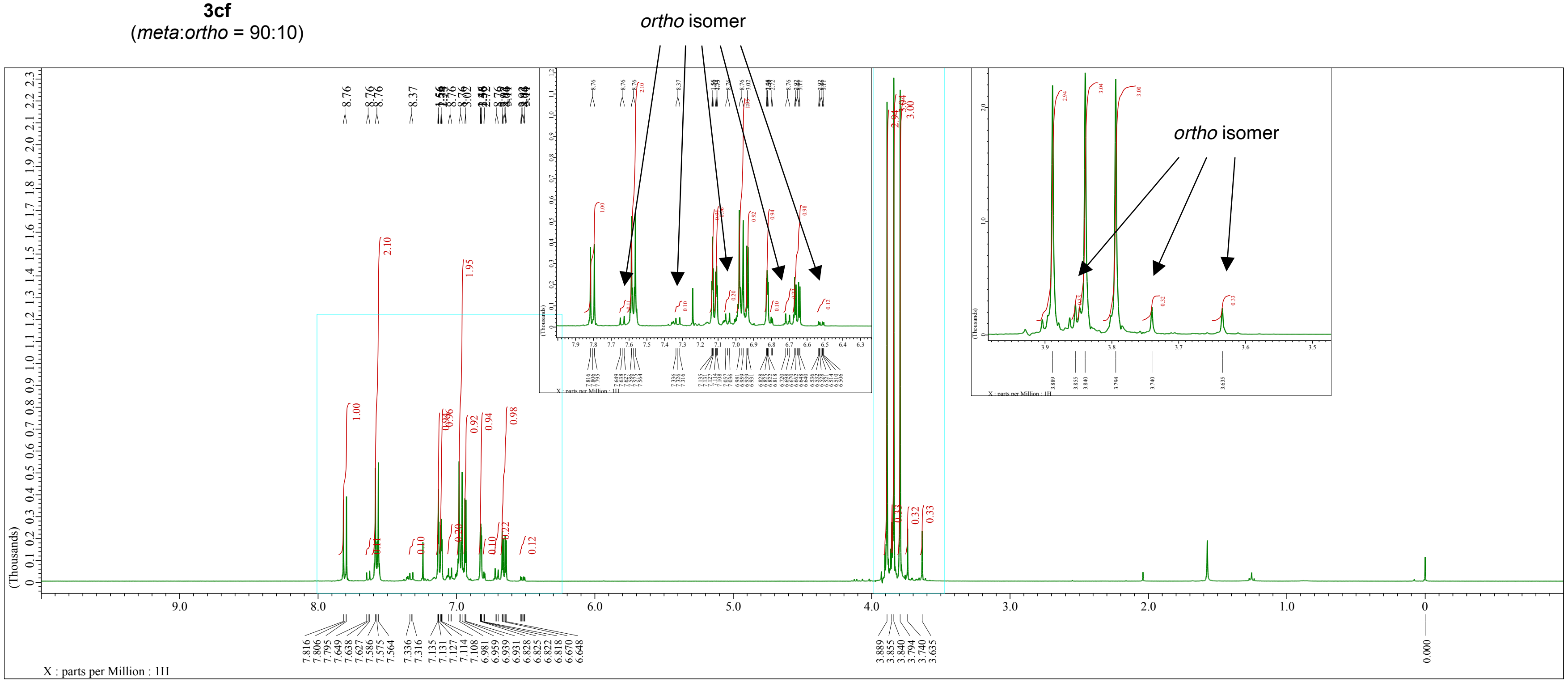

^{13}C NMR spectrum of **3cf** (151 MHz, CDCl₃).

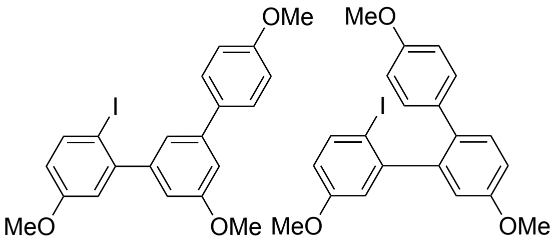

3cf
(*meta*:*ortho* = 90:10)

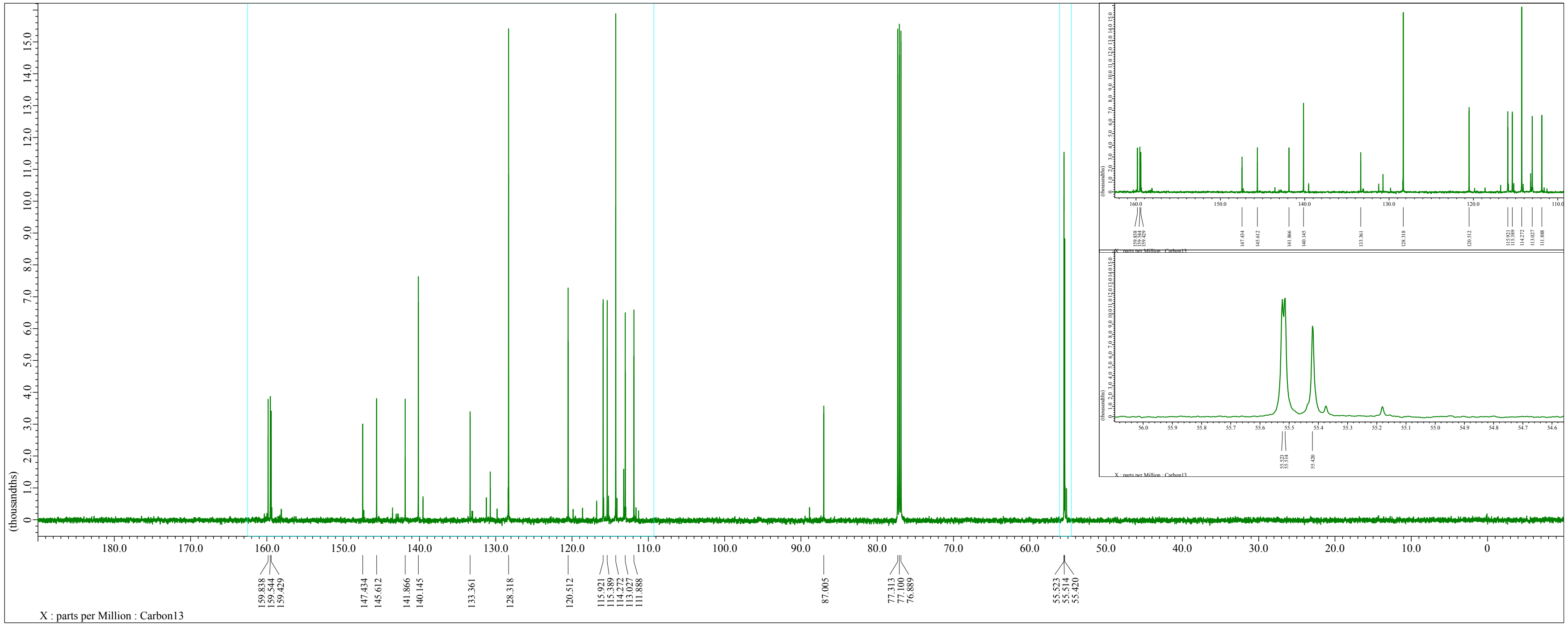

^{1}H NMR spectrum of **3df** (600 MHz, CDCl3).

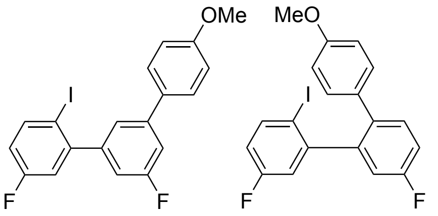

3df
(*meta*:*ortho* = 86:14)

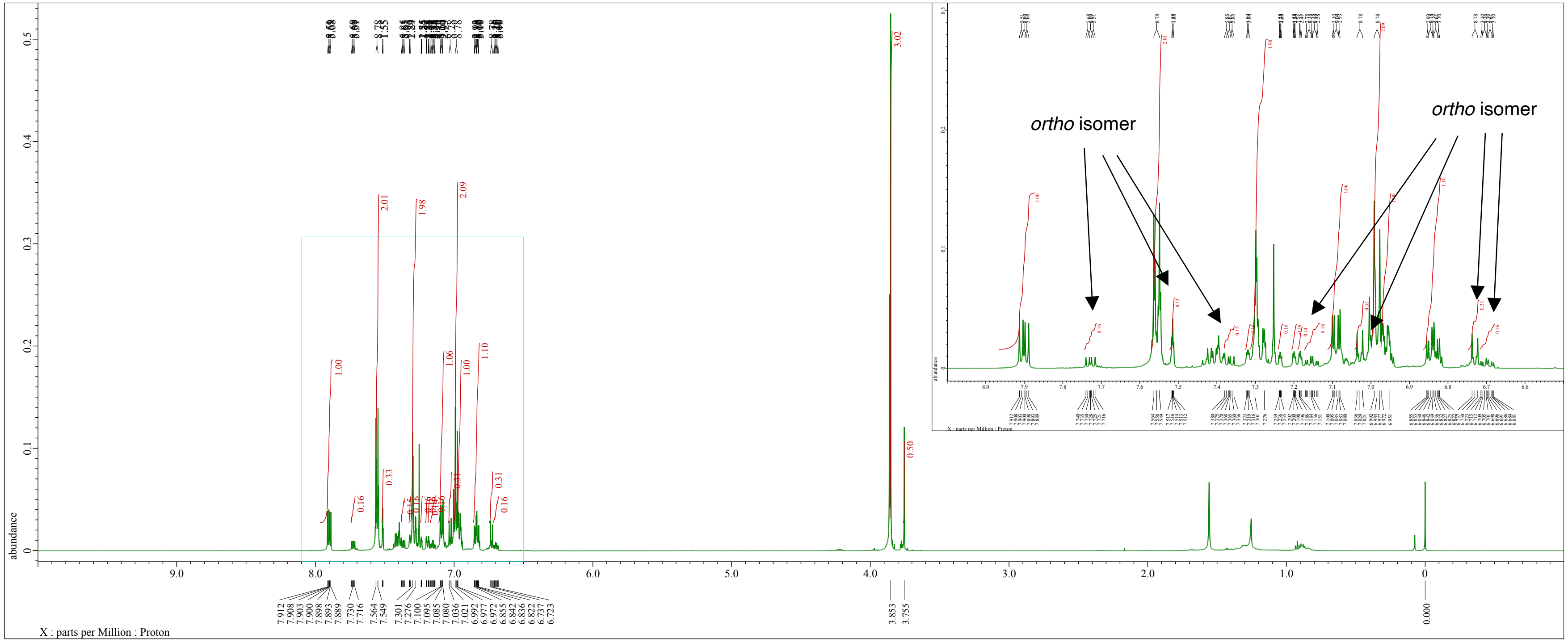

^{19}F NMR spectrum of **3df** (376 MHz, $CDCl_3$).

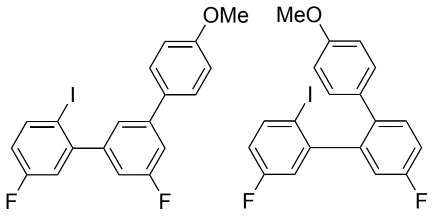

3df
(*meta*:*ortho* = 86:14)

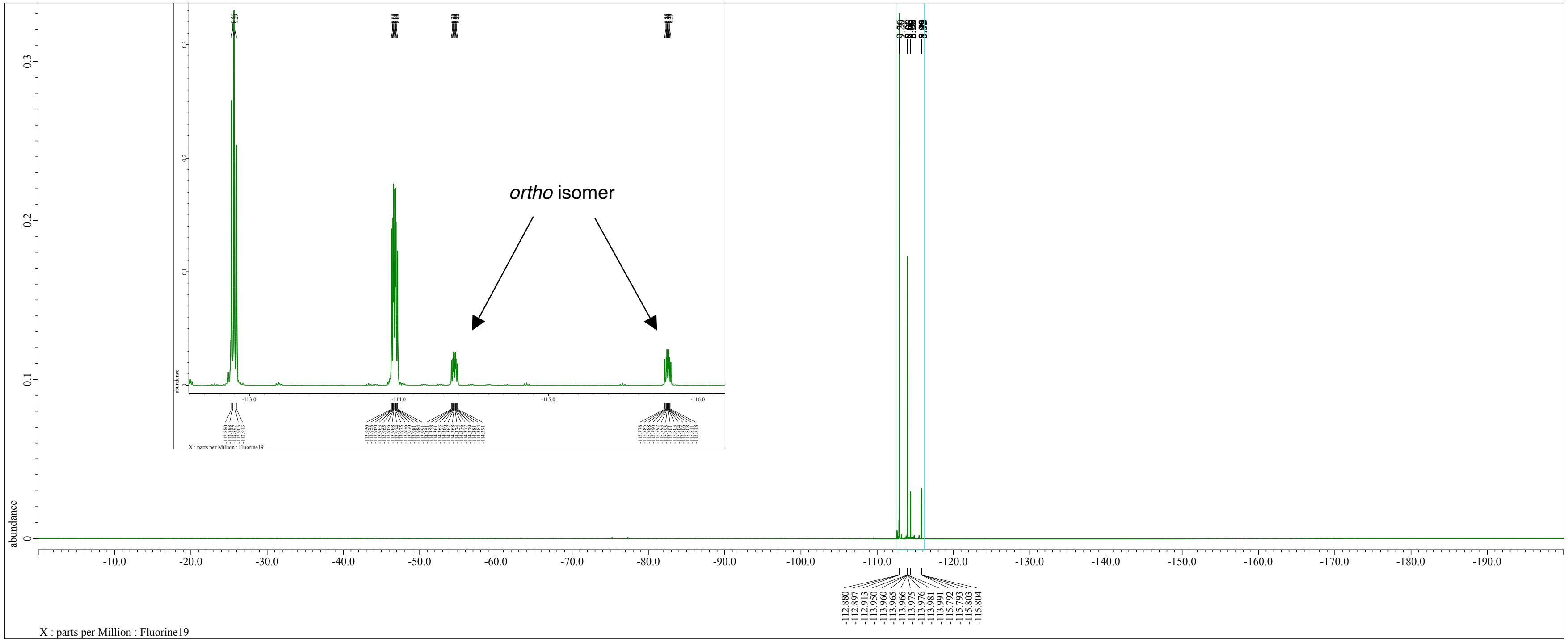

^{13}C NMR spectrum of **3df** (151 MHz, $CDCl_3$).

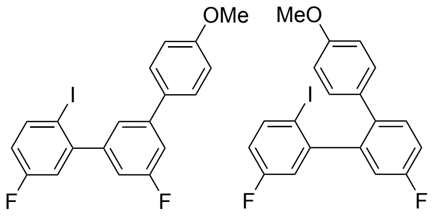

3df
(*meta*:*ortho* = 86:14)

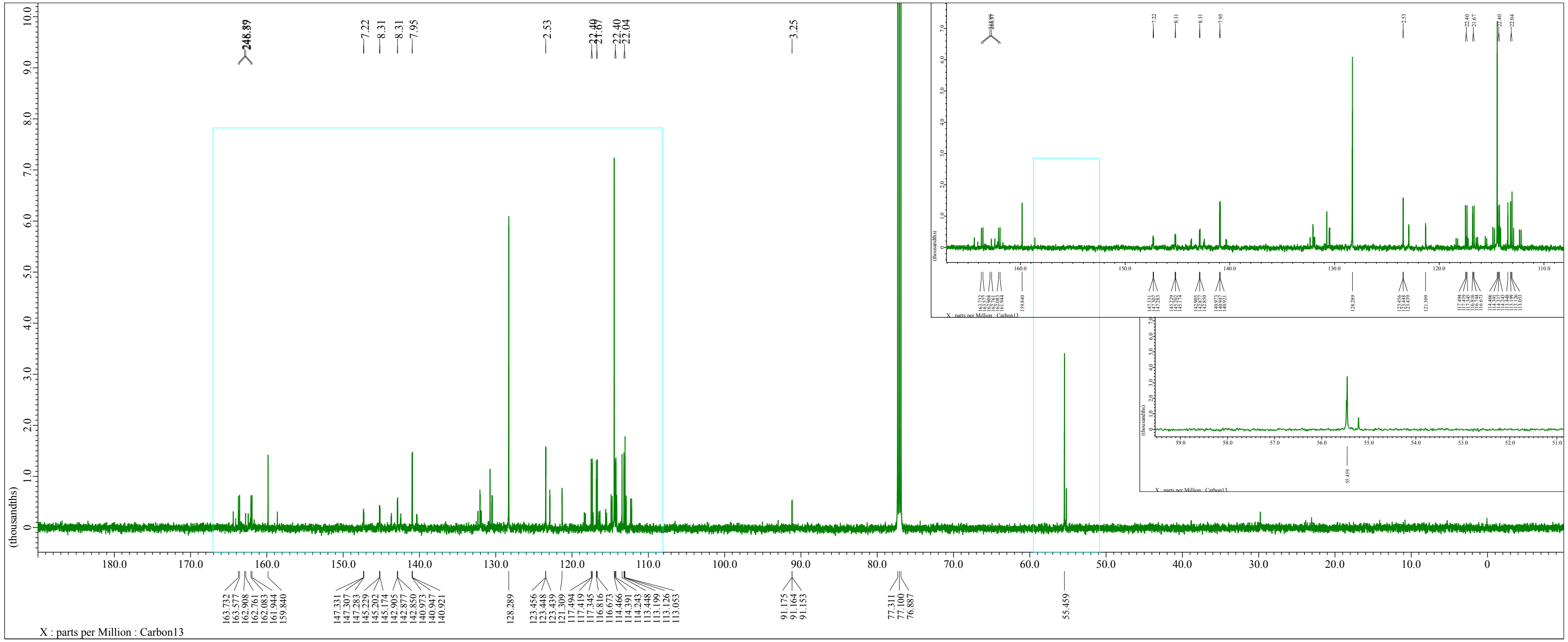

^{1}H NMR spectrum of **3eb** (600 MHz, $CDCl_3$).

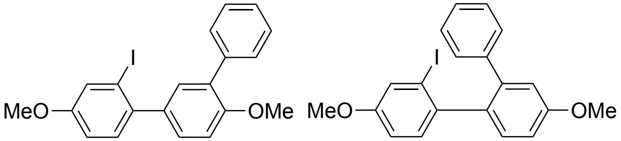

3eb
(*meta*:*ortho* = 16:84)

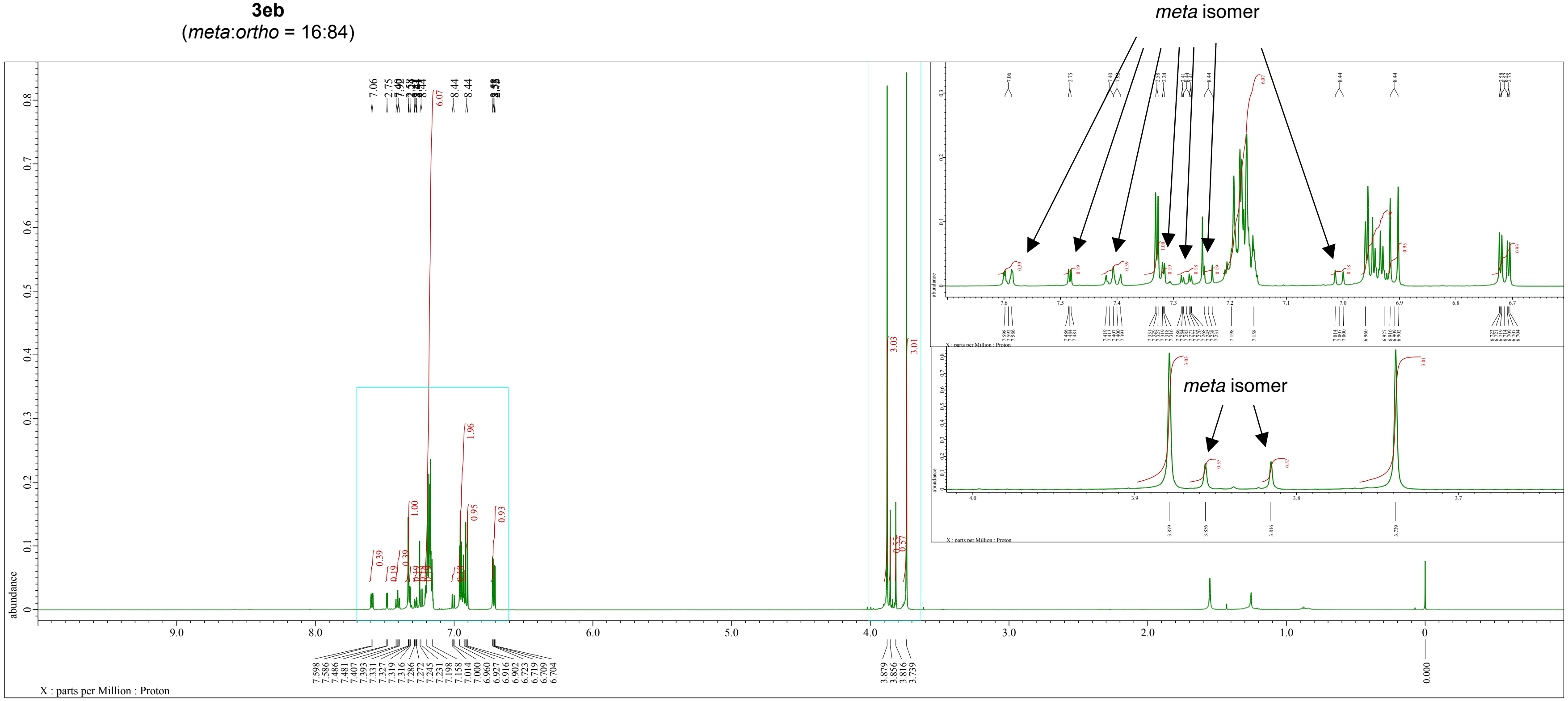

^{13}C NMR spectrum of **3eb** (151 MHz, CDCl$_3$).

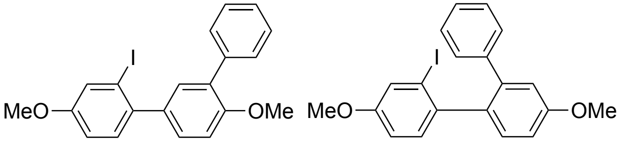

3eb
(*meta*:*ortho* = 16:84)

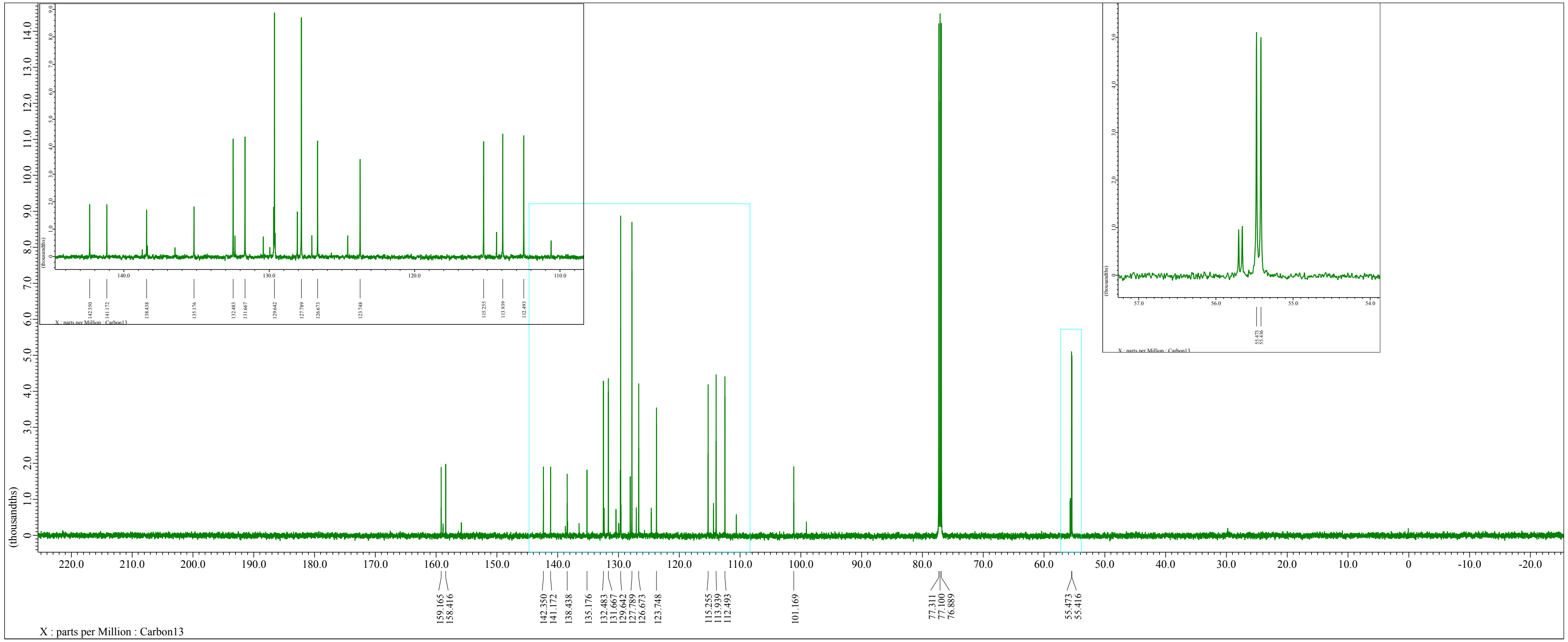

^{1}H NMR spectrum of **3fb** (600 MHz, $CDCl_3$).

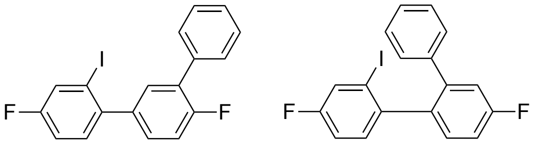

3fb
(*meta*:*ortho* = 50:50)

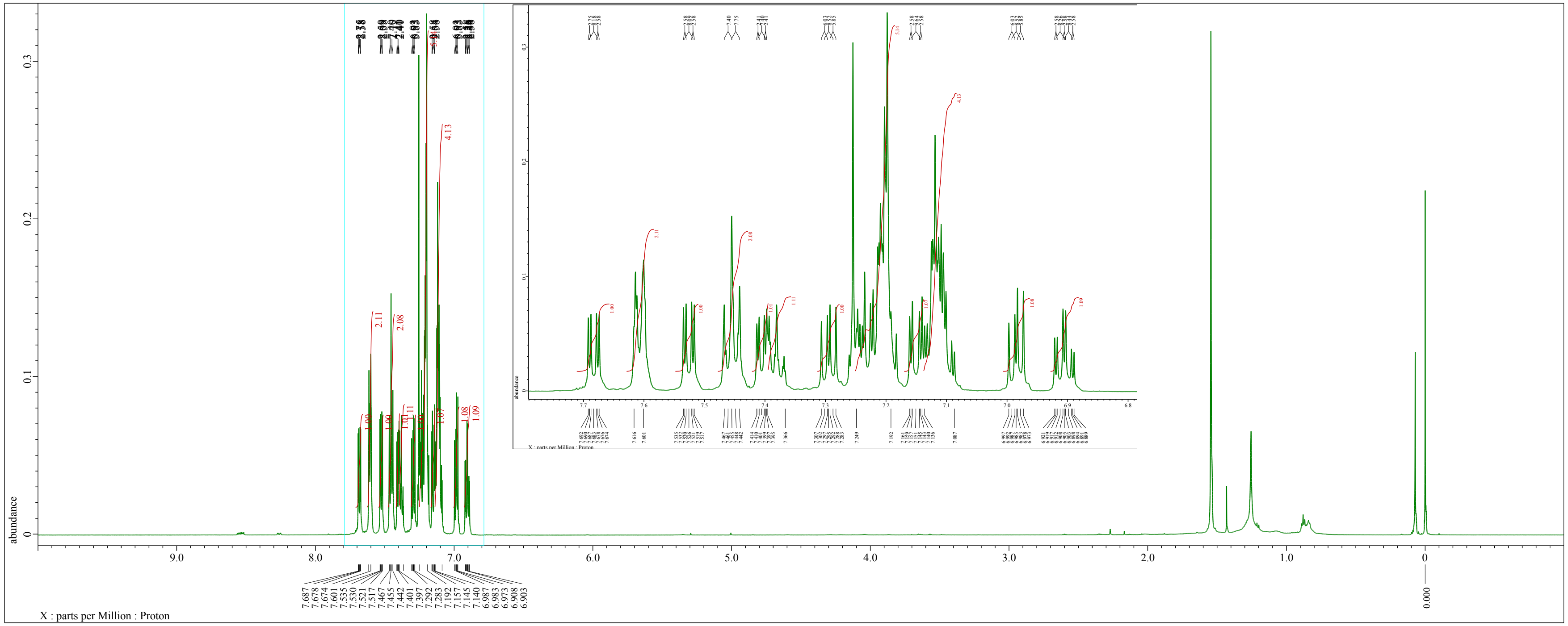

^{19}F NMR spectrum of **3fb** (376 MHz, $CDCl_3$).

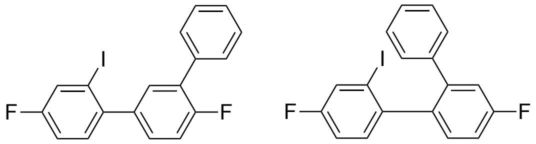

3fb
(*meta*:*ortho* = 50:50)

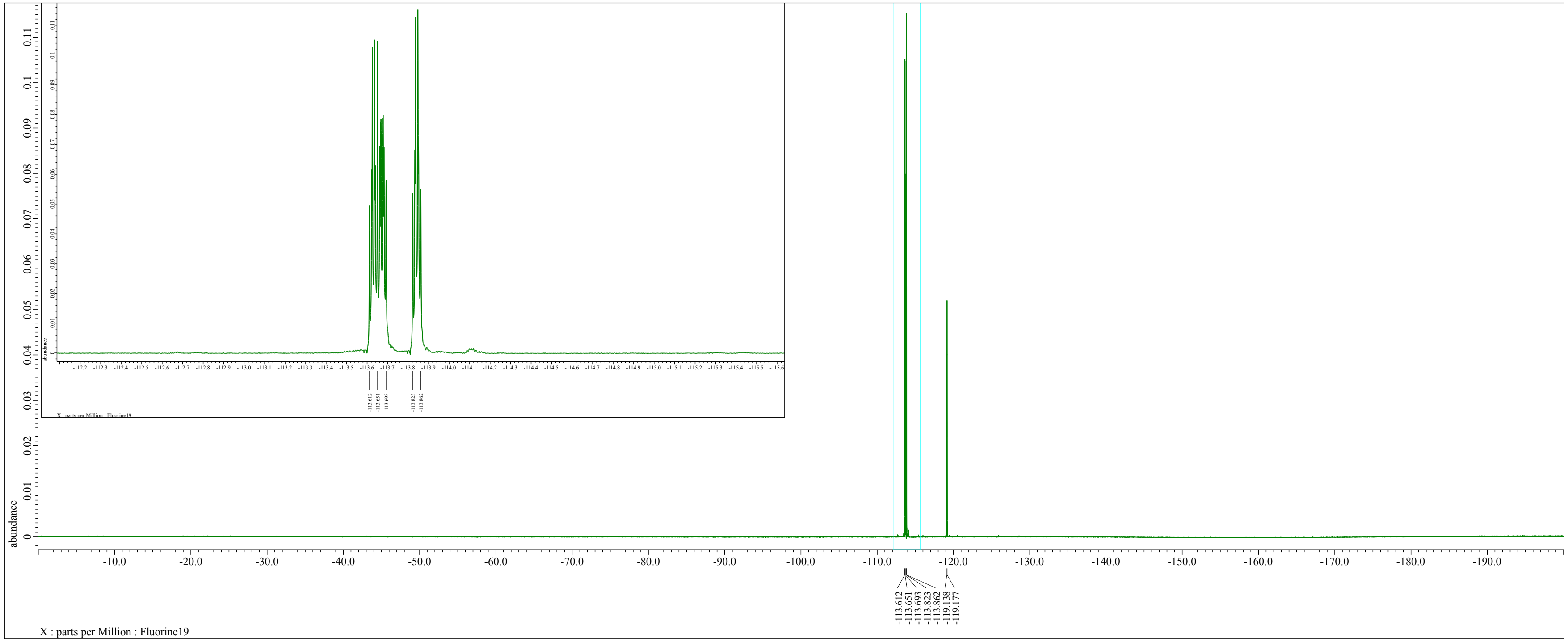

^{13}C NMR spectrum of **3fb** (151 MHz, $CDCl_3$).

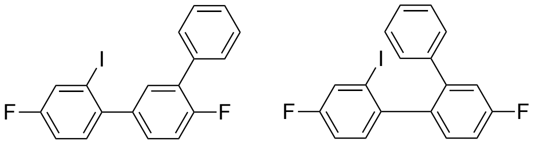

3fb
(*meta*:*ortho* = 50:50)

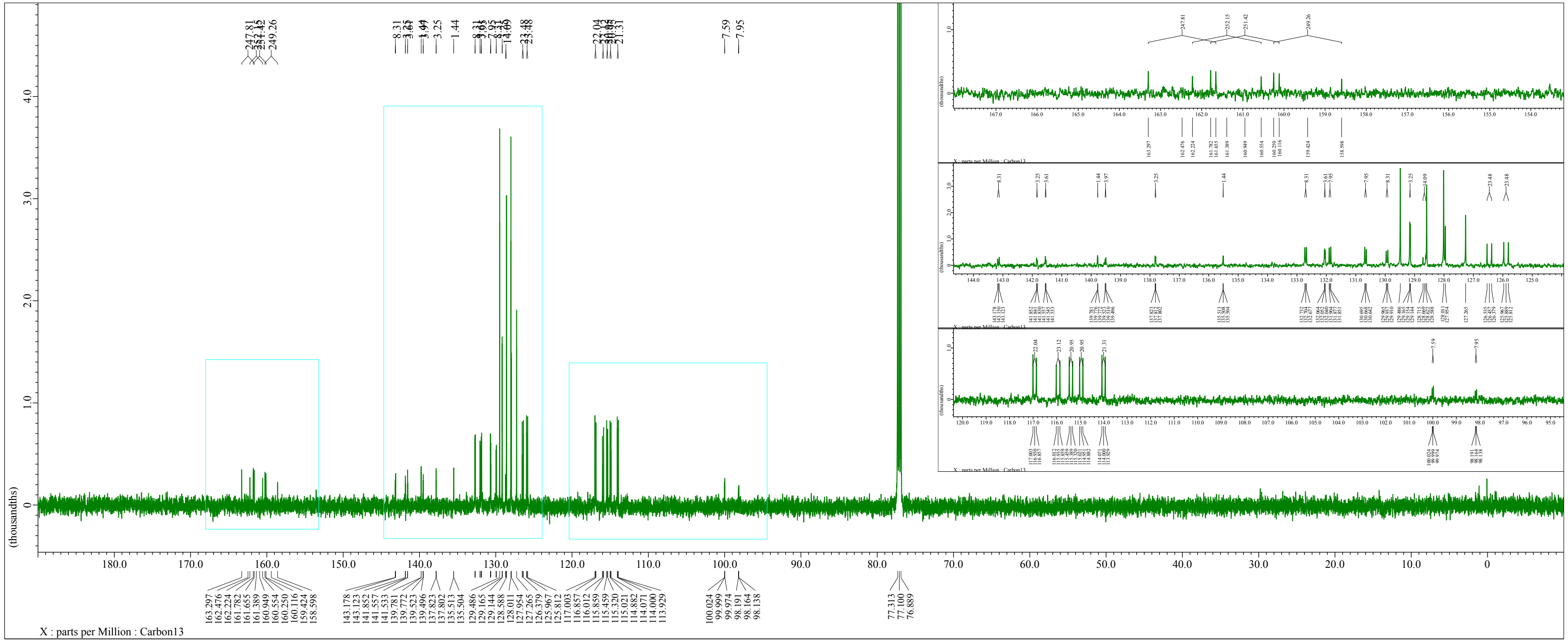

^{1}H NMR spectrum of **3gb** (600 MHz, $CDCl_3$).

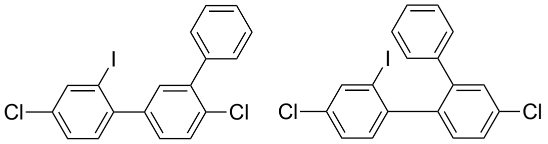

3gb
(*meta*:*ortho* = 70:30)

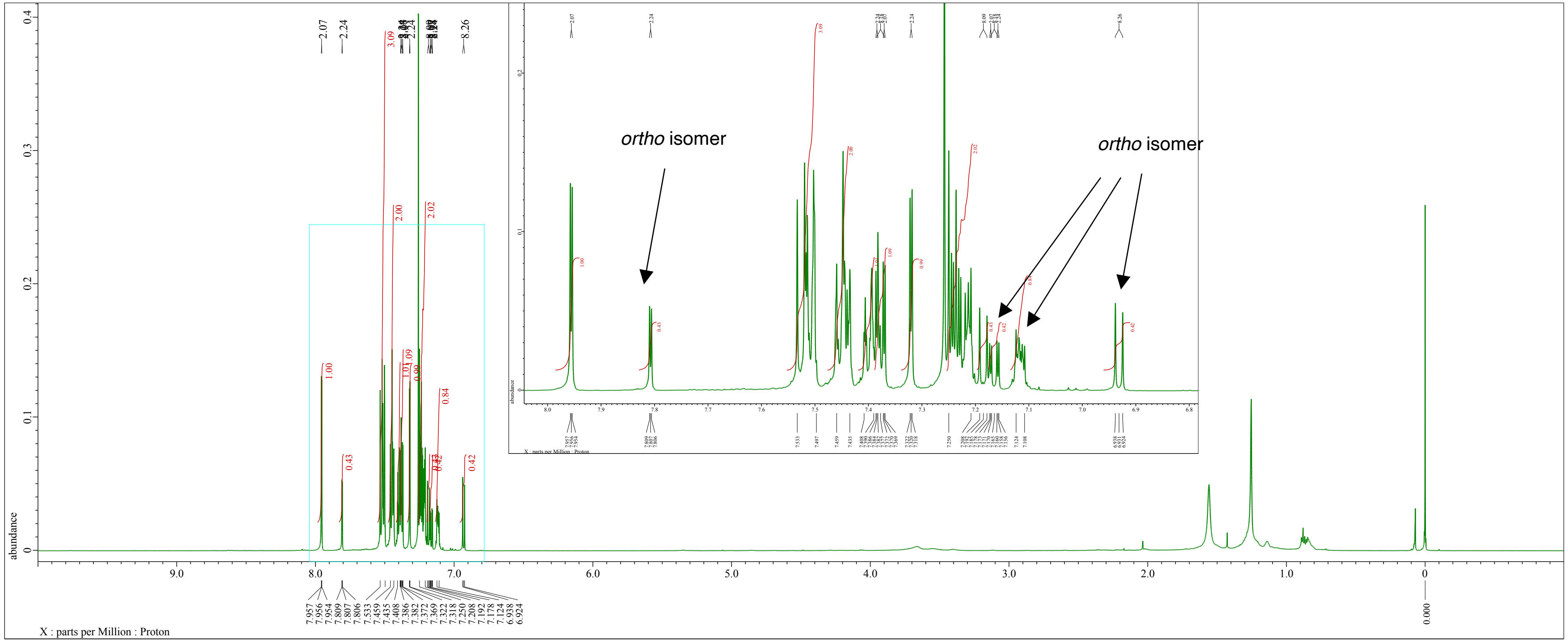

^{13}C NMR spectrum of **3gb** (151 MHz, $CDCl_3$).

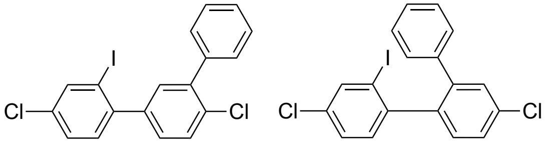

3gb
(*meta*:*ortho* = 70:30)

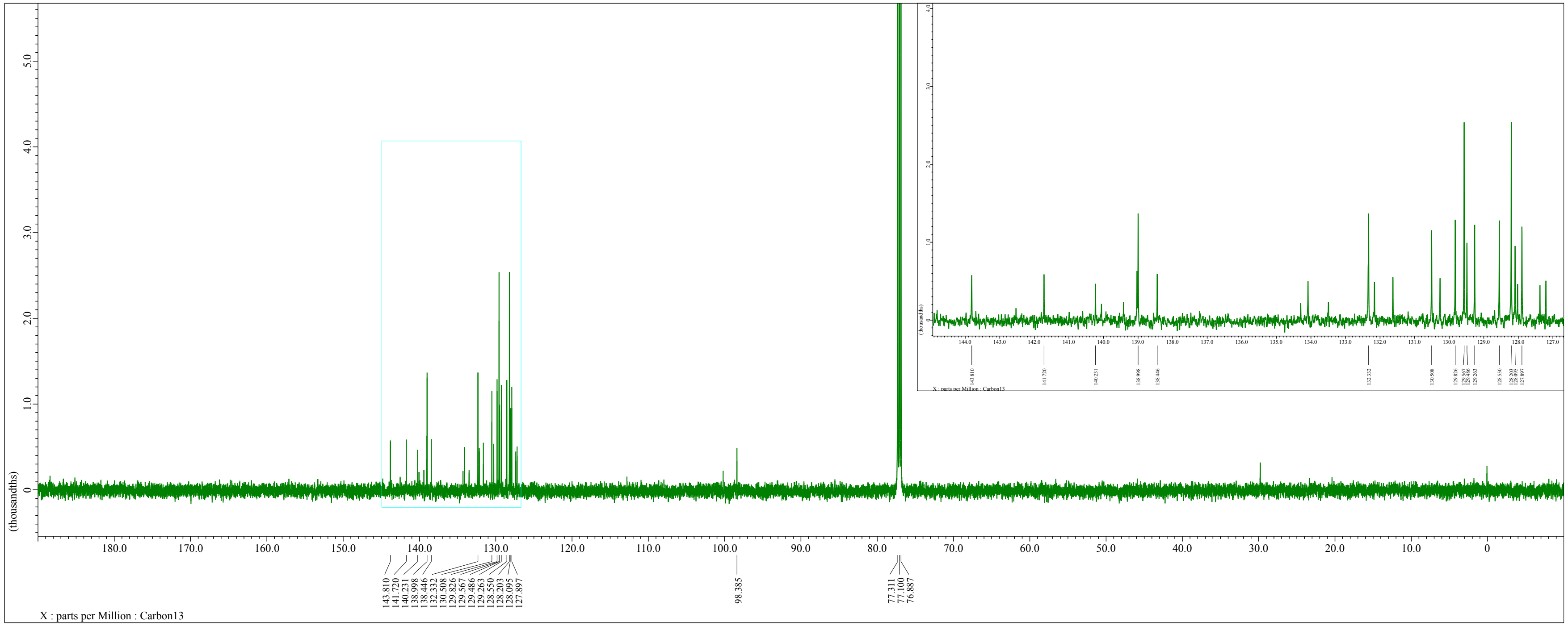

^{1}H NMR spectrum of **3hb** (400 MHz, $CDCl_3$).

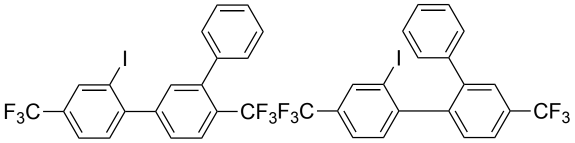

3hb
(*meta*:*ortho* = 16:84)

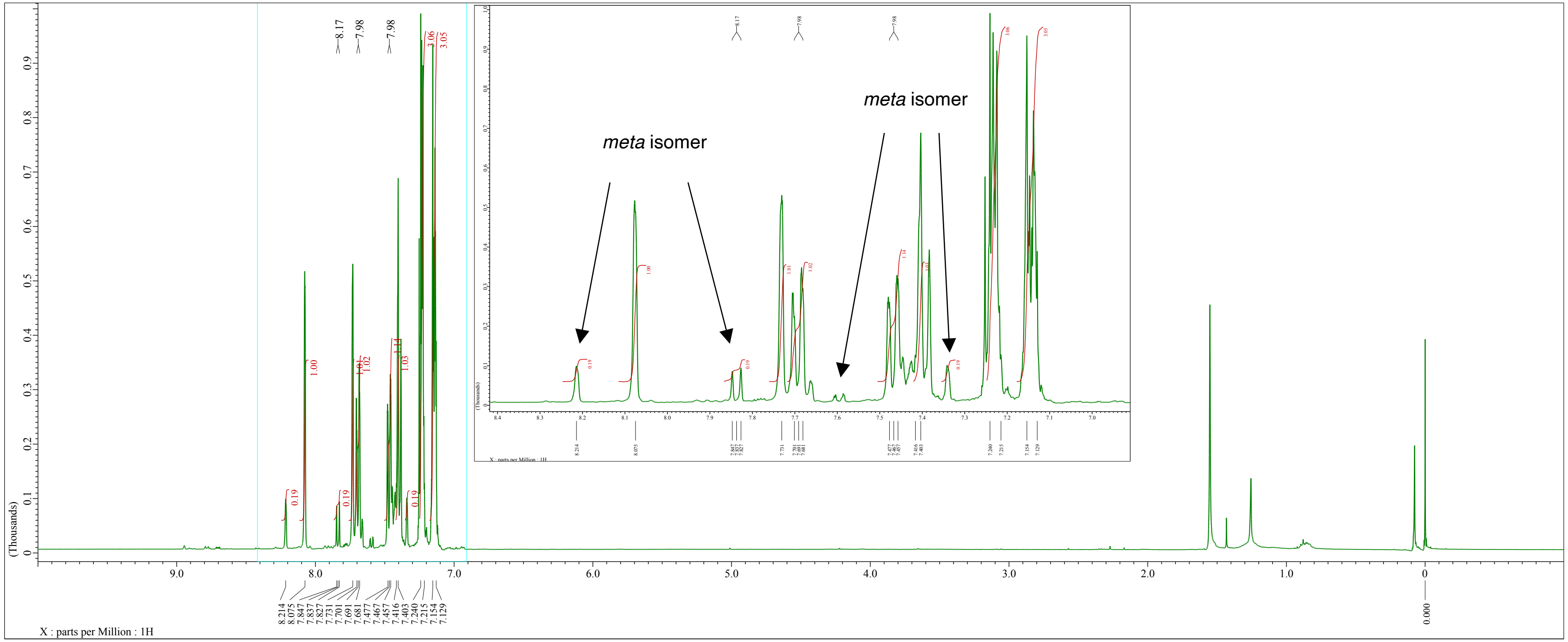

^{19}F NMR spectrum of **3hb** (376 MHz, $CDCl_3$).

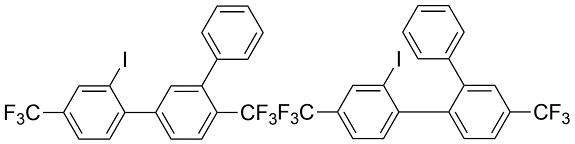

3hb
(*meta*:*ortho* = 16:84)

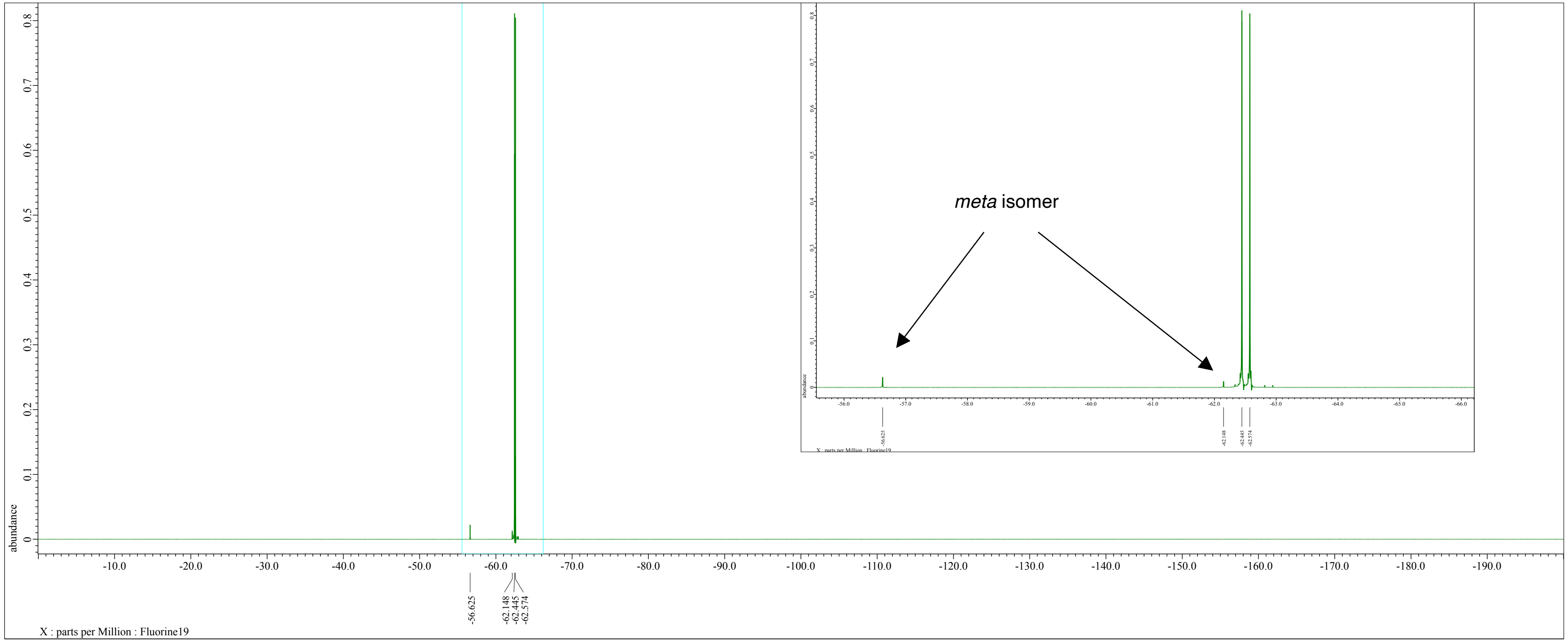

^{13}C NMR spectrum of **3hb** (151 MHz, $CDCl_3$).

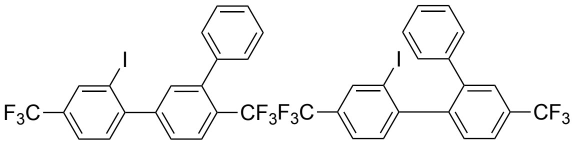

3hb
(*meta*:*ortho* = 16:84)

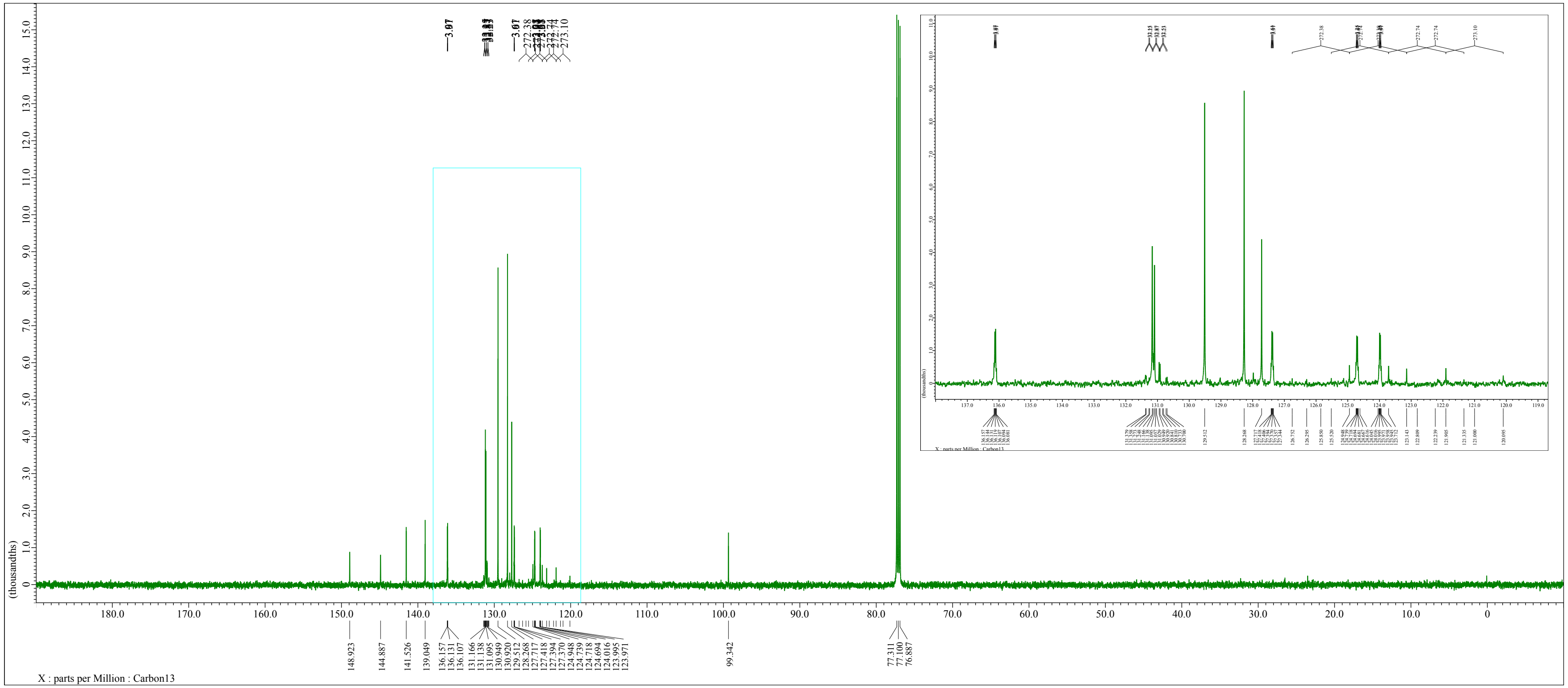

^{1}H NMR spectrum of **3ib** (600 MHz, $CDCl_3$).

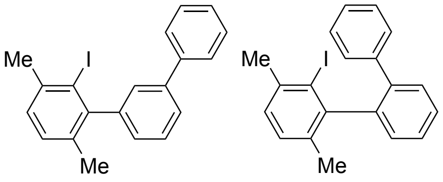

3ib
(*meta*:*ortho* = 81:19)

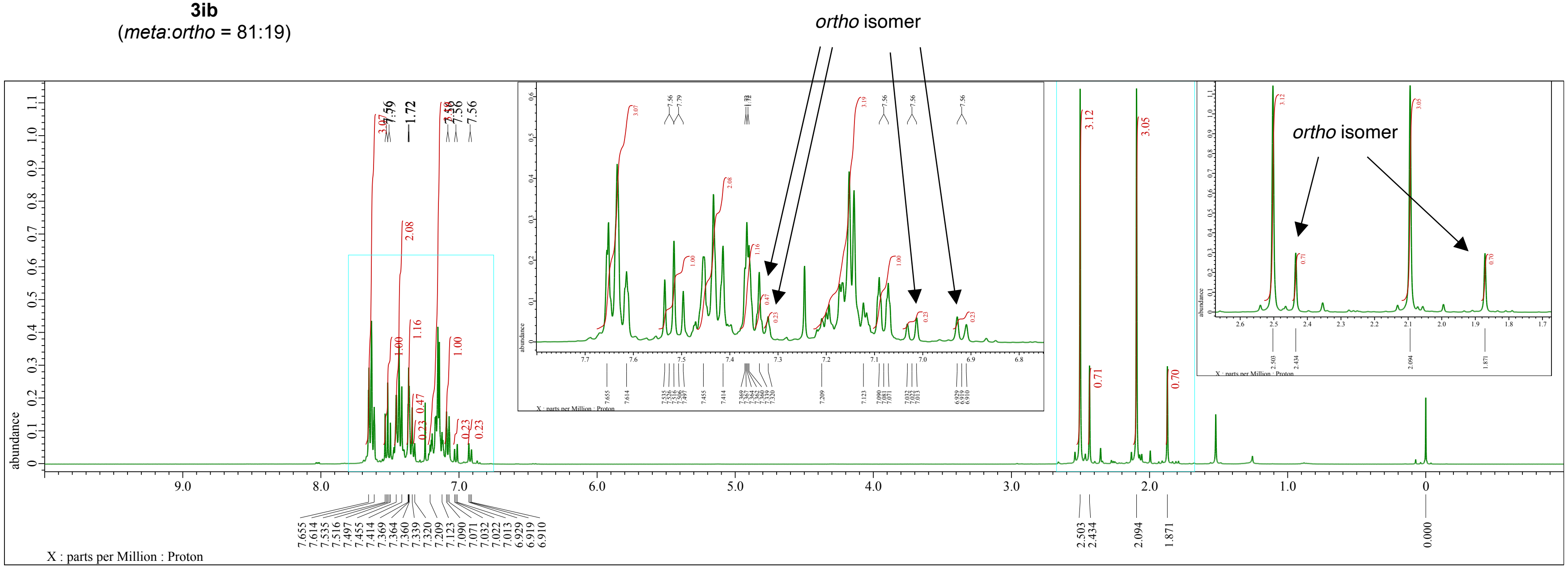

^{13}C NMR spectrum of **3ib** (151 MHz, CDCl₃).

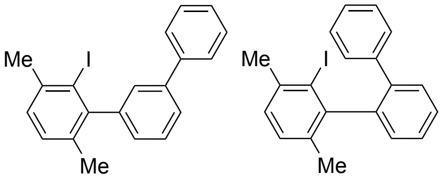

3ib
(*meta*:*ortho* = 81:19)

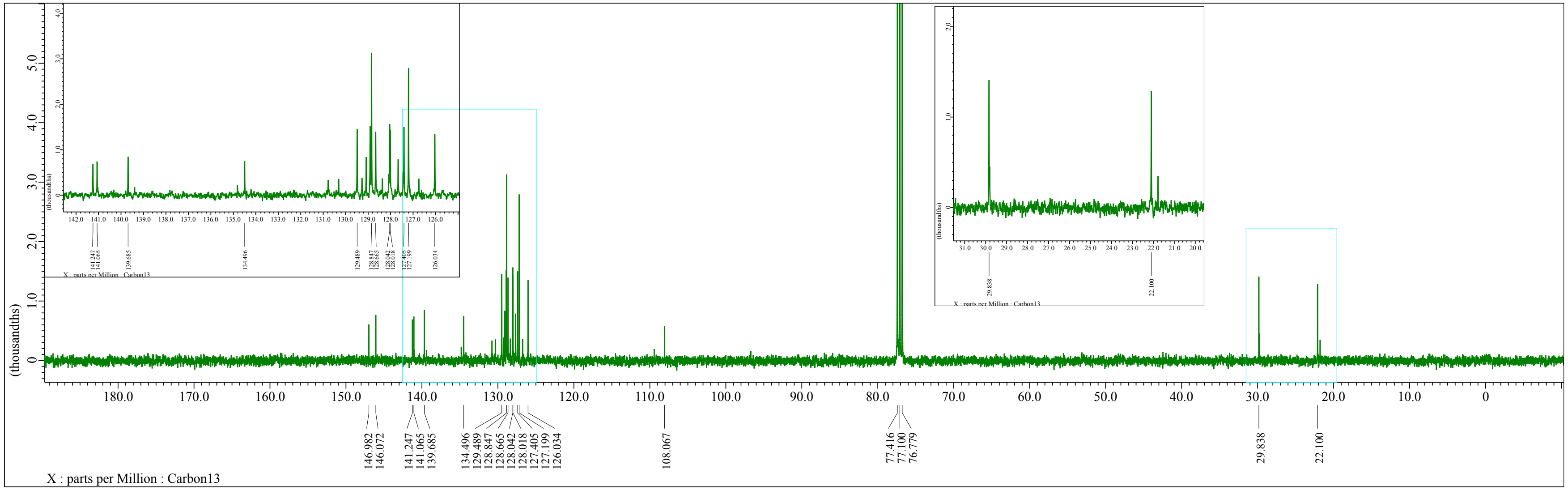

^{1}H NMR spectrum of **3jf** (400 MHz, $CDCl_3$).

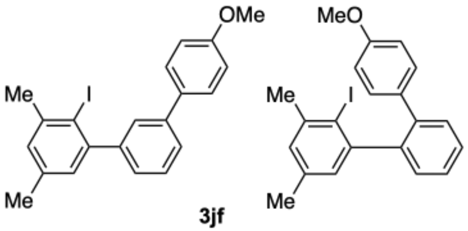

3jf
(*meta*:*ortho* = 88:12)

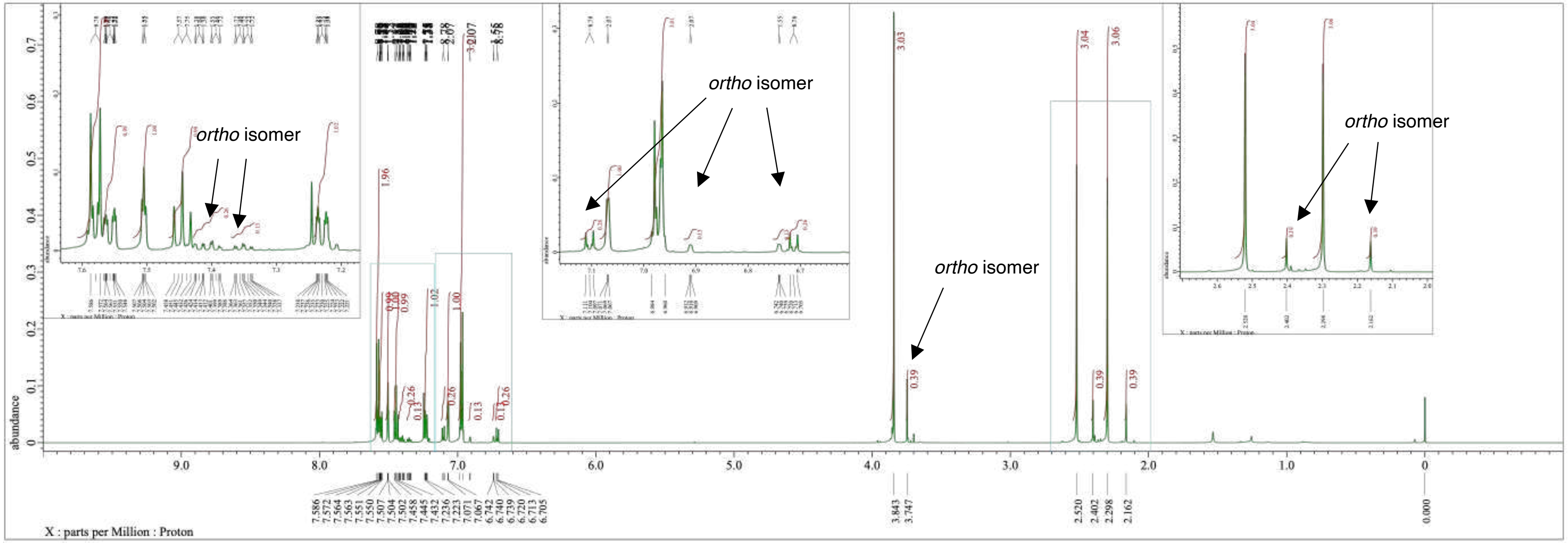

^{13}C NMR spectrum of **3jf** (151 MHz, $CDCl_3$).

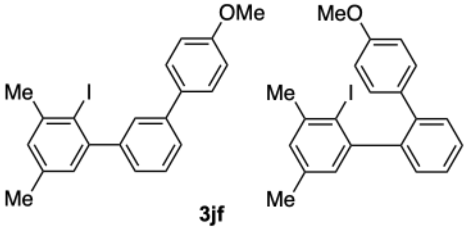

3jf
(*meta*:*ortho* = 88:12)

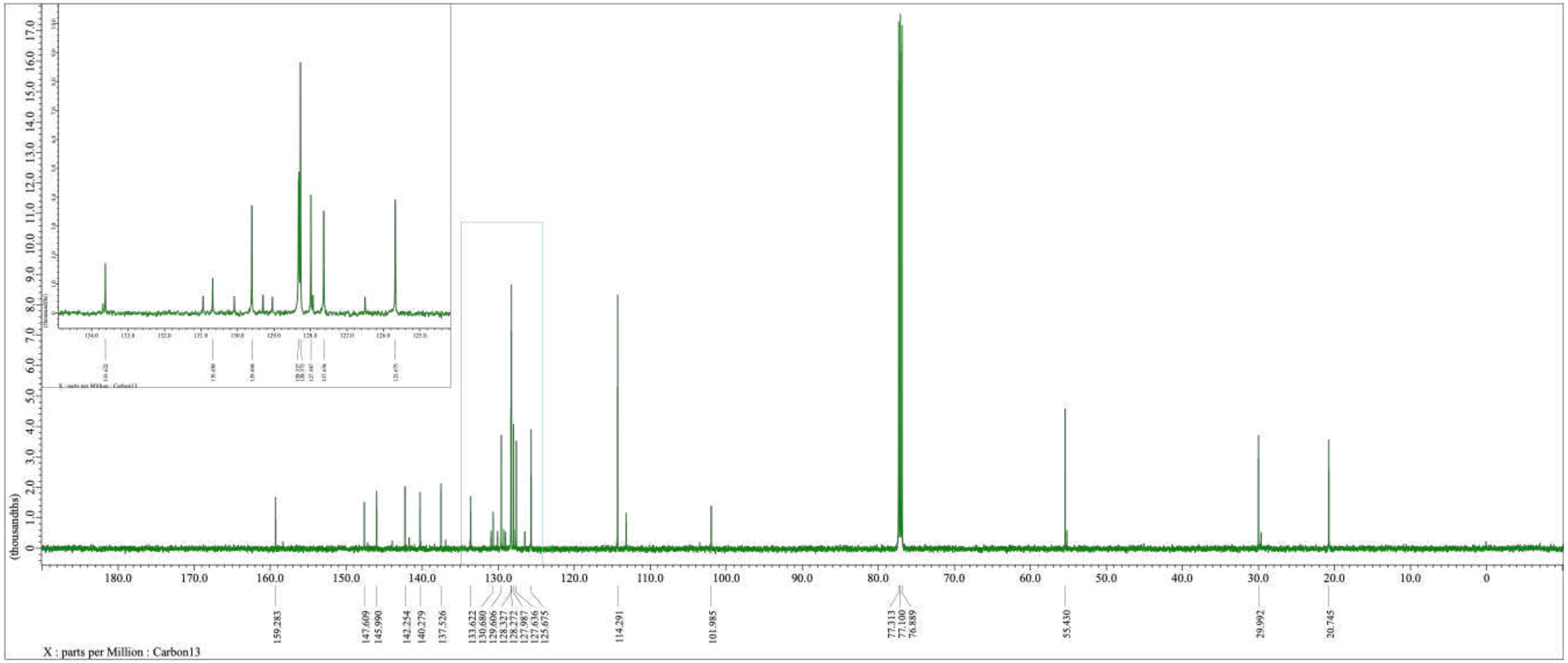

^{1}H NMR spectrum of **3kf** (400 MHz, CDCl$_3$).

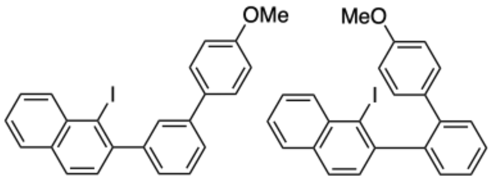

3kf
(*meta*:*ortho* = 89:11)

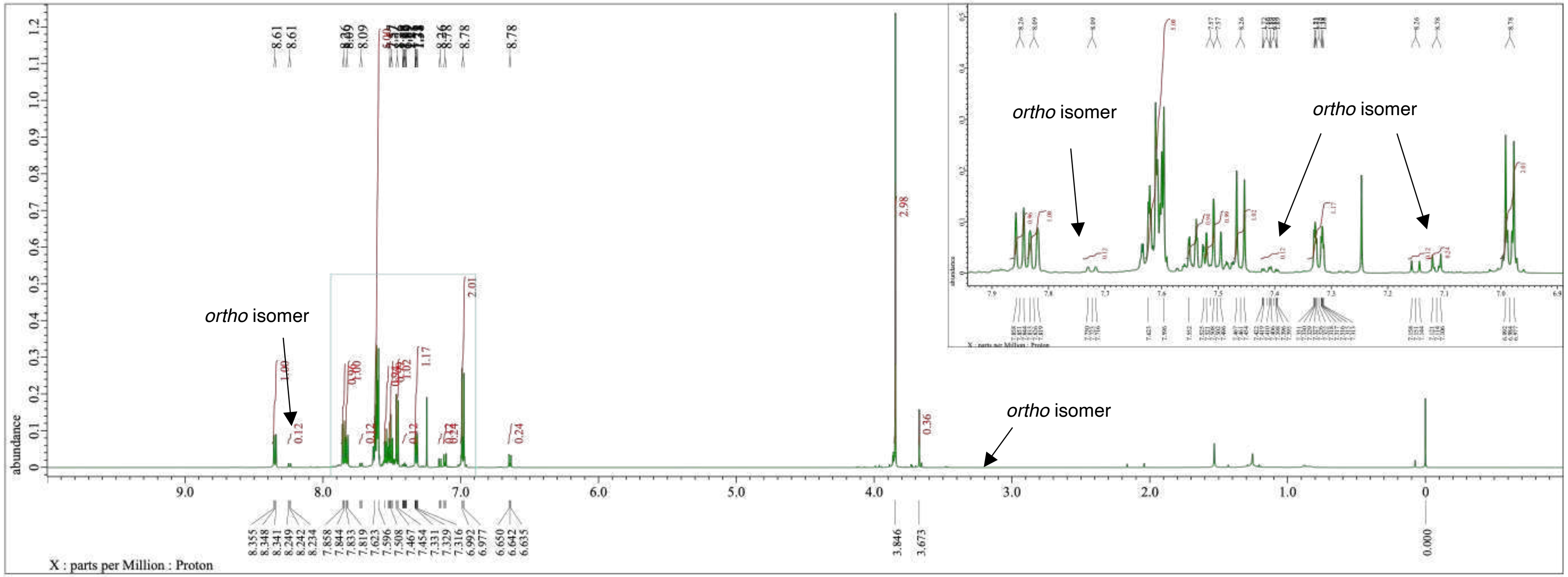

^{13}C NMR spectrum of **3kf** (151 MHz, $CDCl_3$).

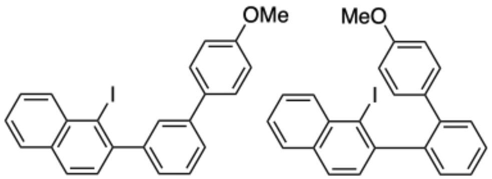

3kf
(*meta*:*ortho* = 89:11)

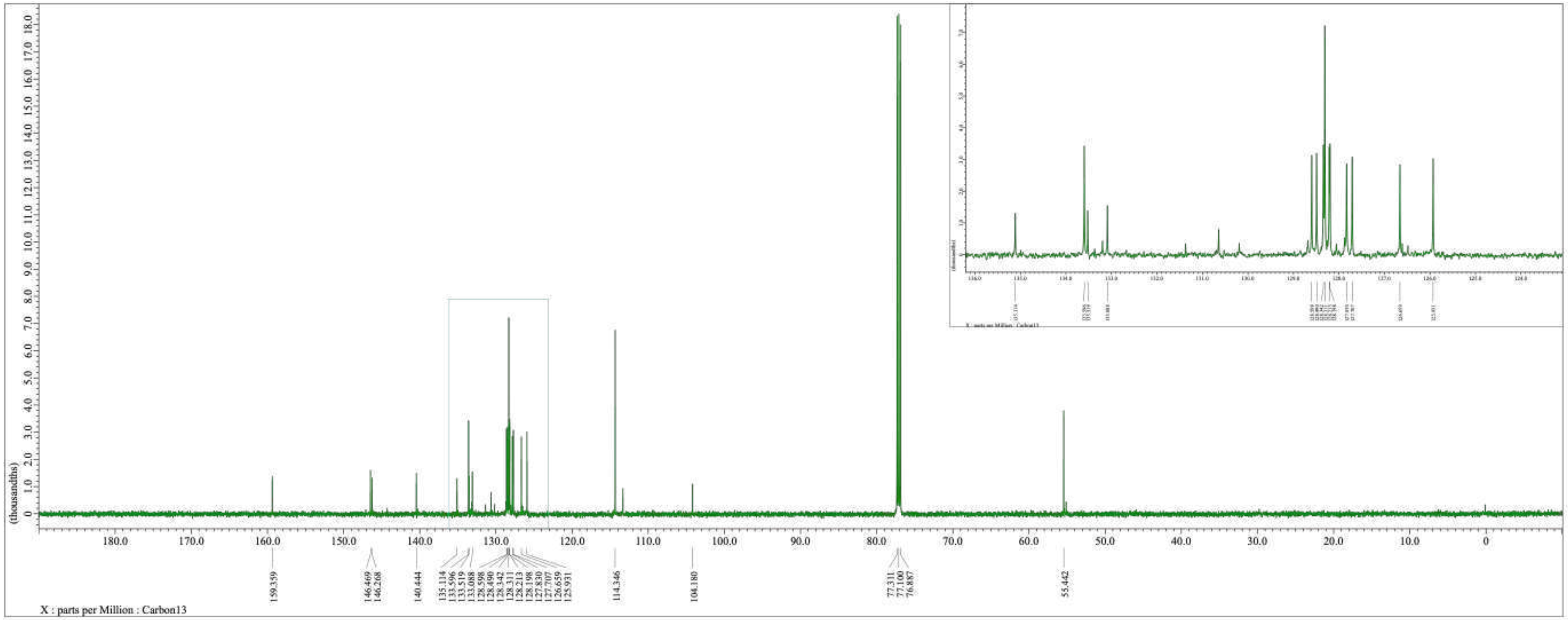

^{1}H NMR spectrum of **3lb** (400 MHz, $CDCl_3$).

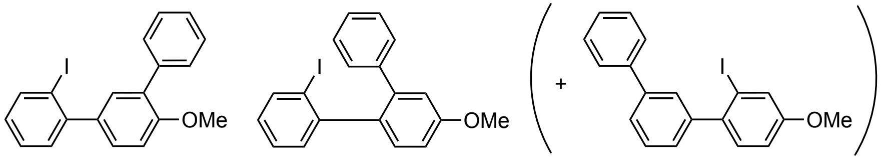

3lb **3lb’**

(*meta*-**3lb**:*ortho*-**3lb**:**3lb’** = 14:82:4)

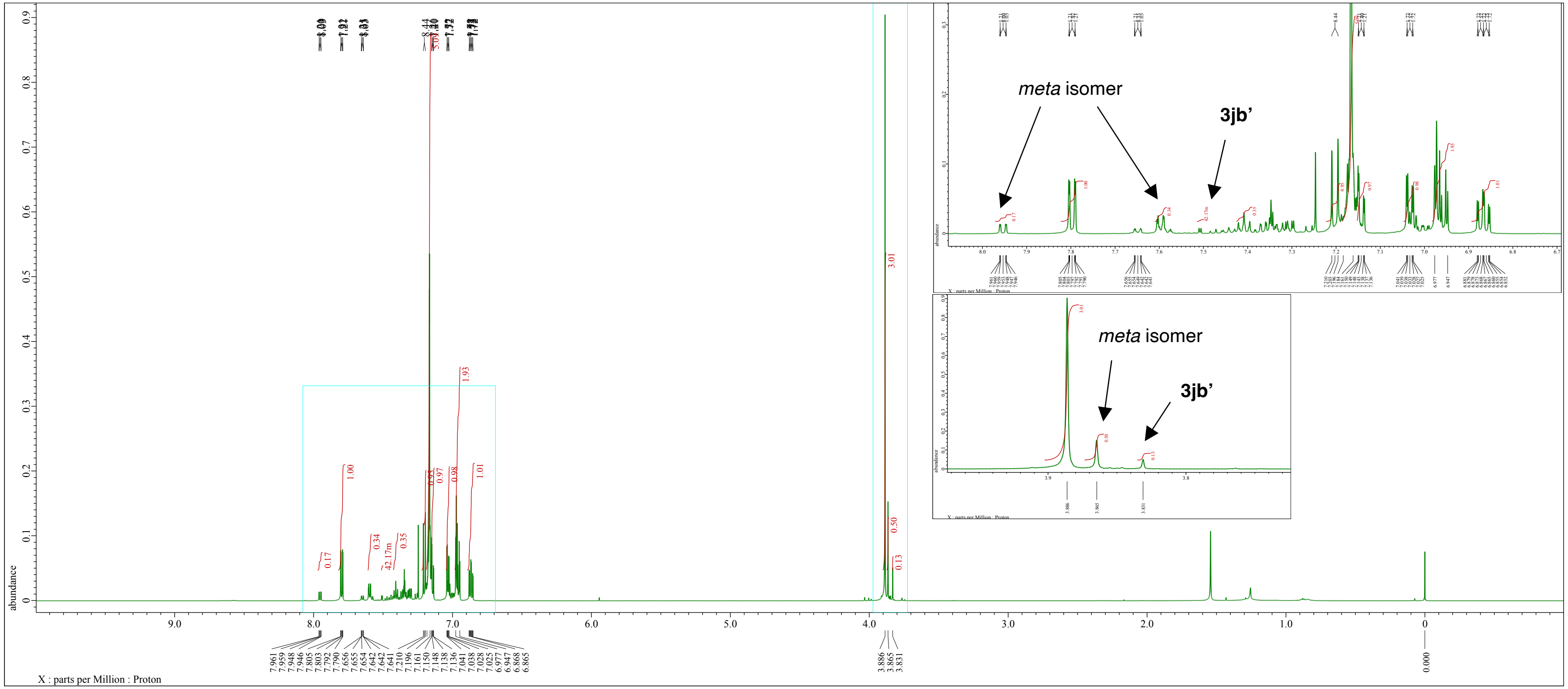

^{13}C NMR spectrum of **3lb** (151 MHz, $CDCl_3$).

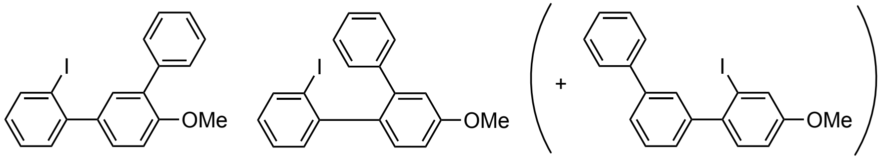

(*meta*-**3lb**:*ortho*-**3lb**:**3lb'** = 14:82:4)

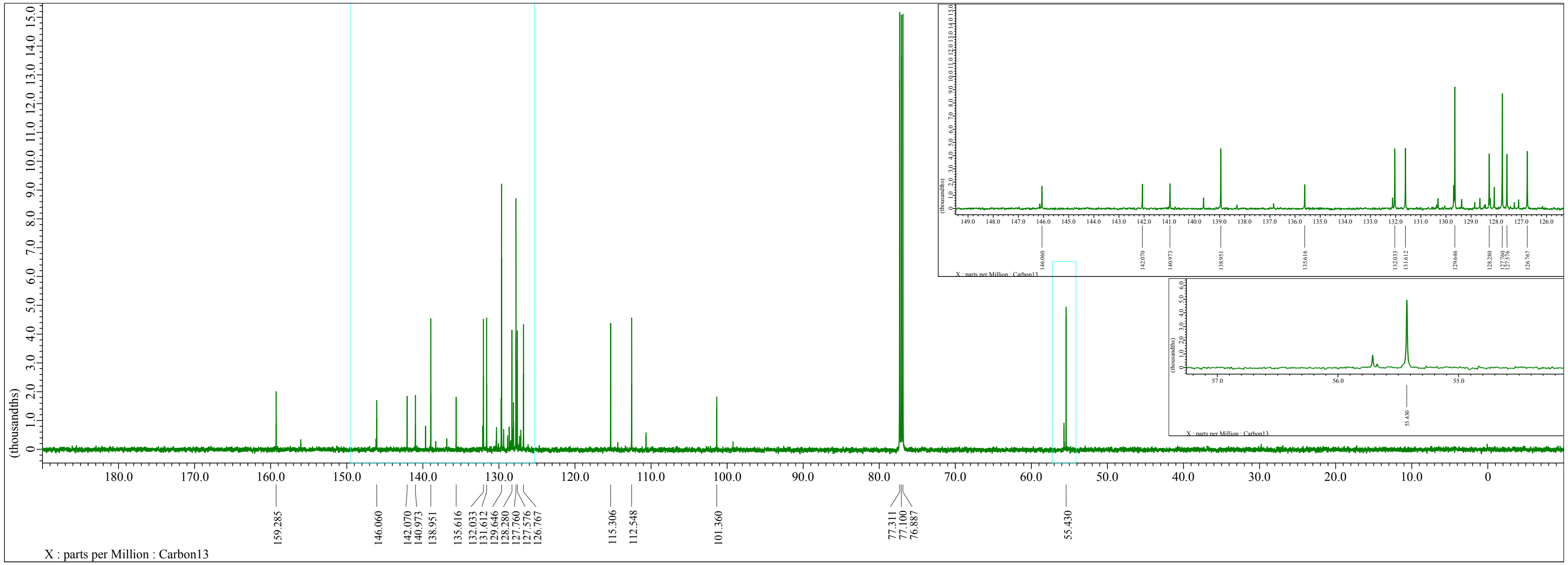

^{1}H NMR spectrum of **S3eb** (400 MHz, $CDCl_3$).

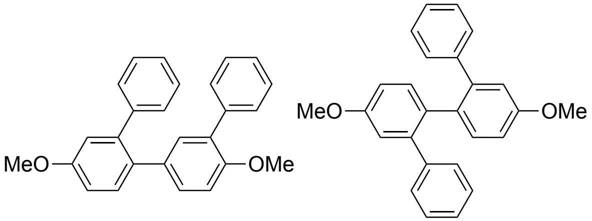

S3eb
(*meta*:*ortho* 16:84)

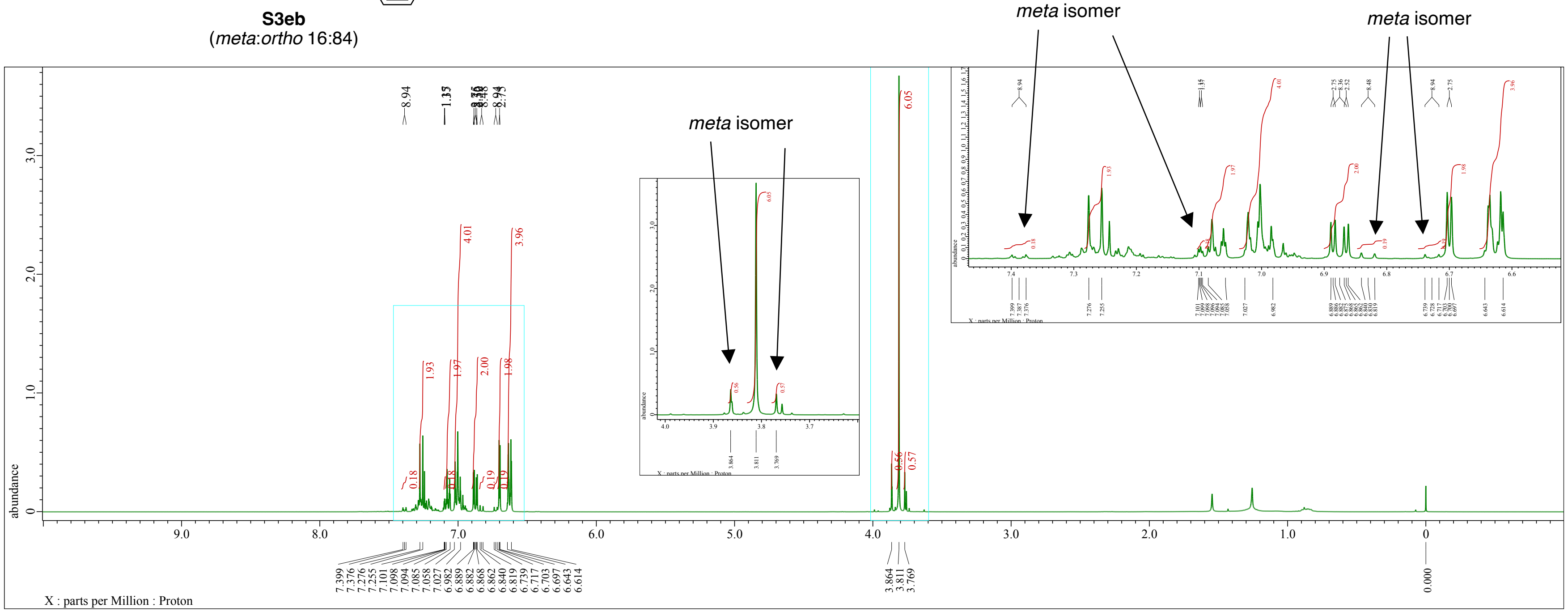

^{13}C NMR spectrum of **S3eb** (151 MHz, $CDCl_3$).

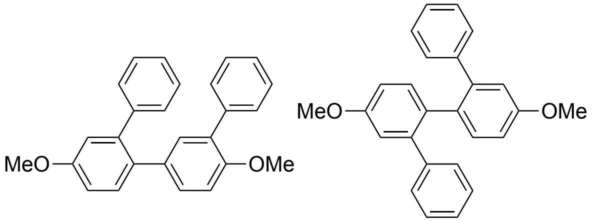

S3eb
(*meta*:*ortho* 16:84)

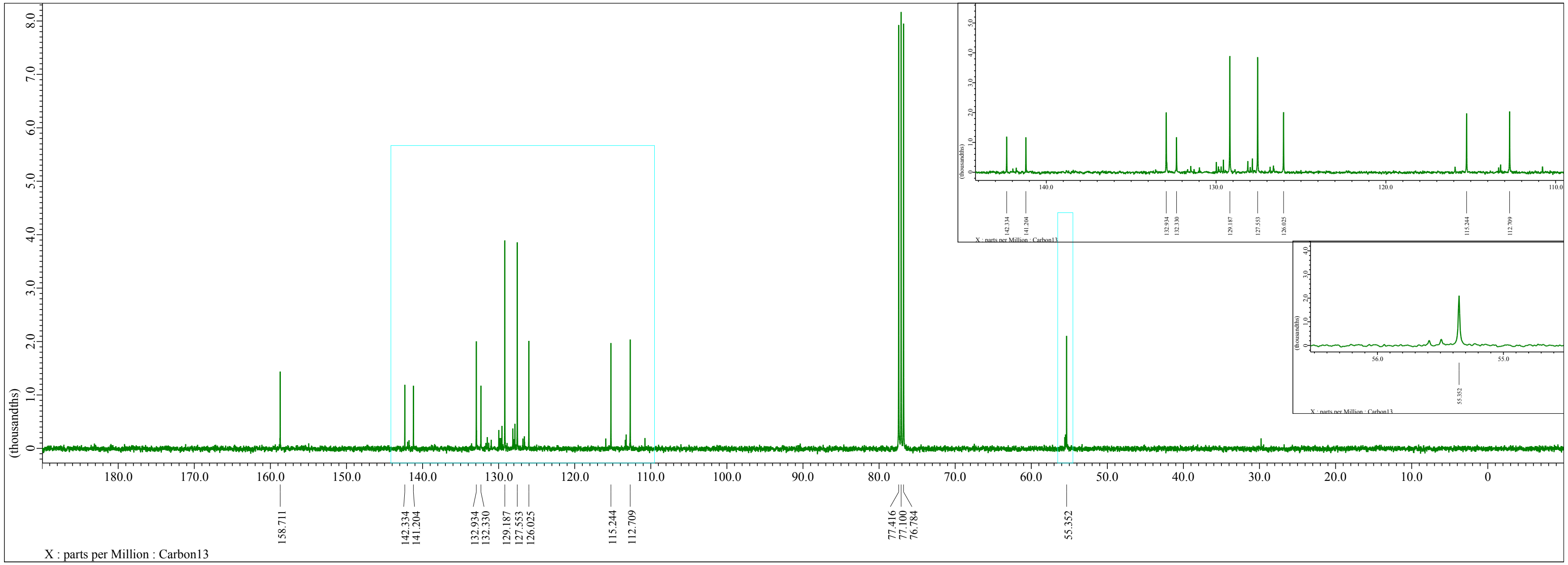

^{1}H NMR spectrum of **S3gb** (400 MHz, $CDCl_3$).

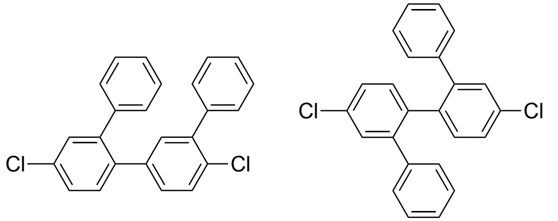

S3gb
(*meta*:*ortho* 64:36)

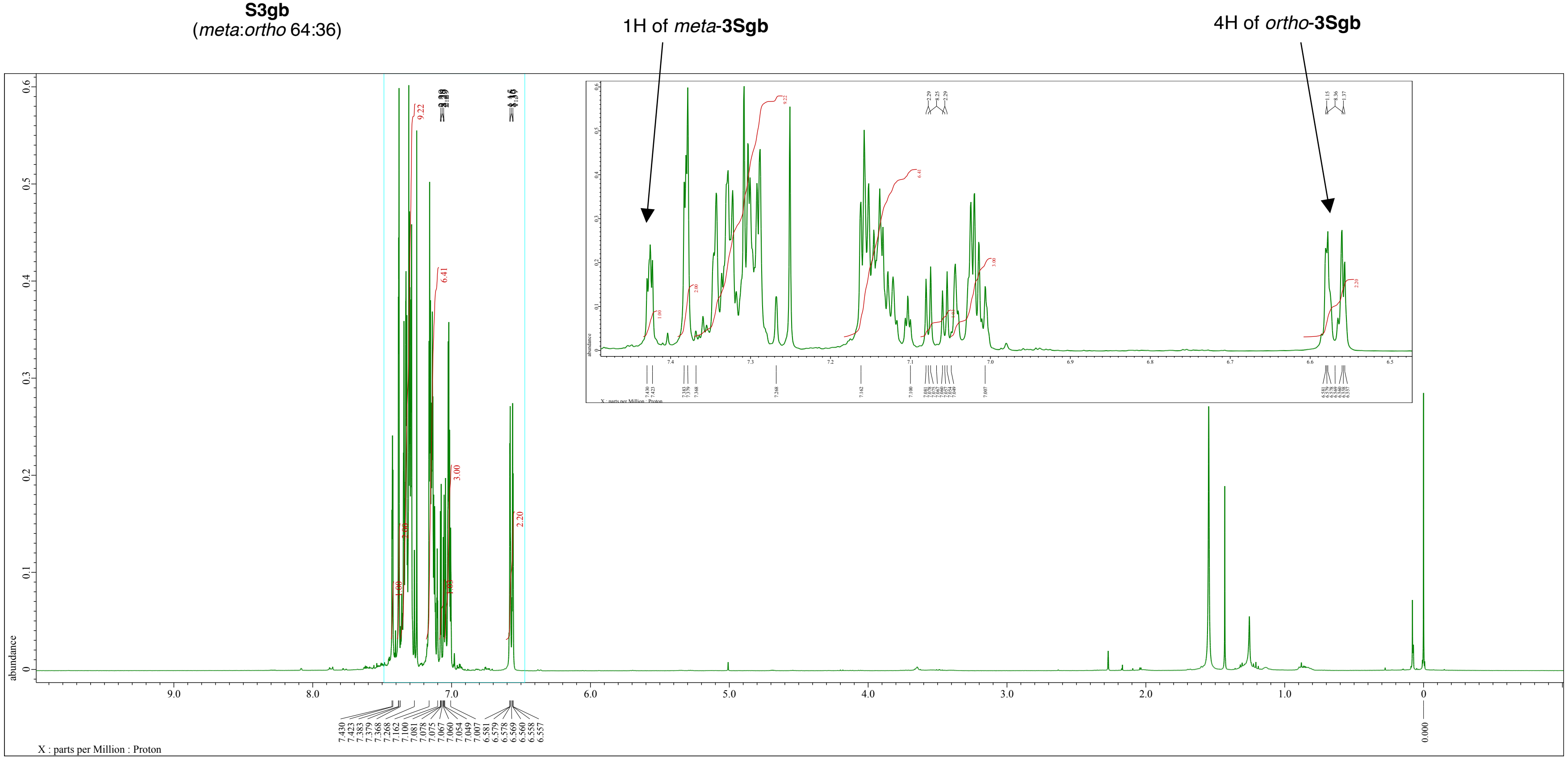

^{13}C NMR spectrum of **S3gb** (151 MHz, $CDCl_3$).

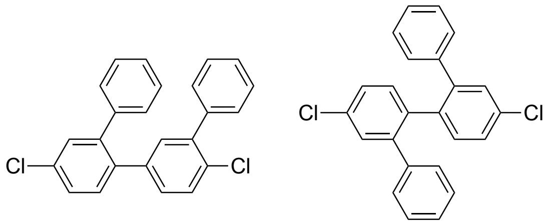

S3gb
(*meta*:*ortho* 64:36)

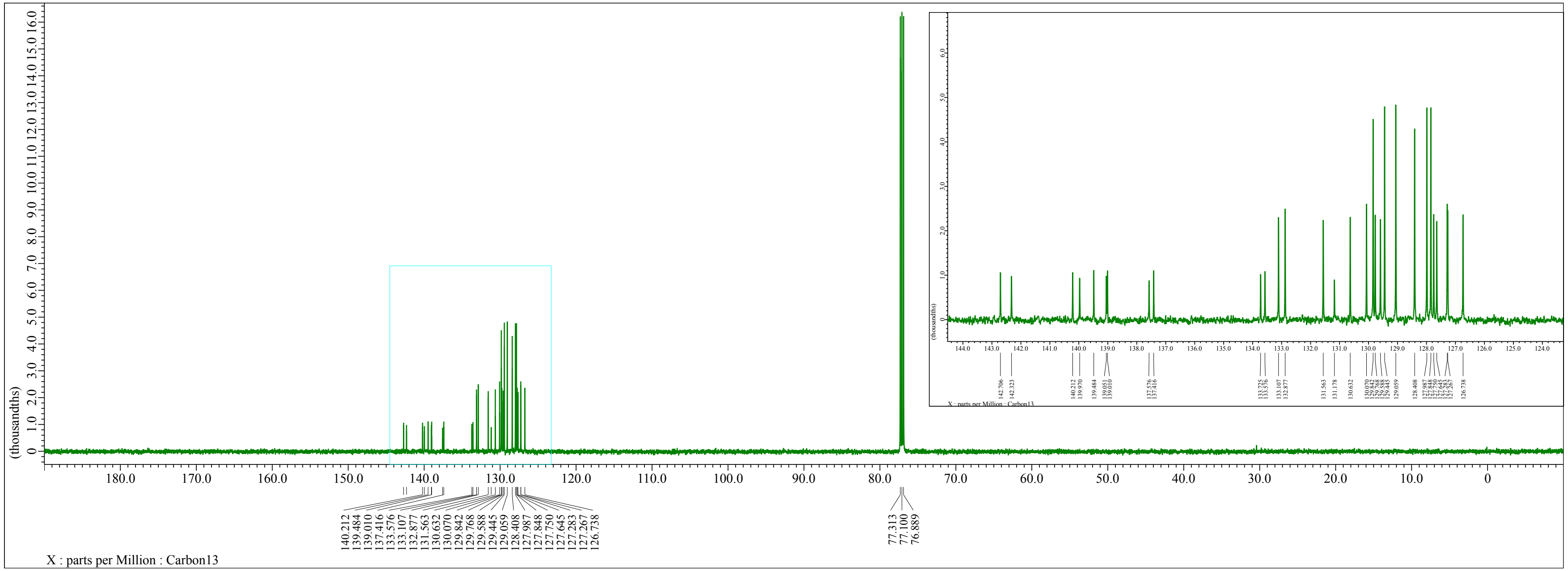

^{1}H NMR spectrum of **S3hb** (400 MHz, $CDCl_3$).

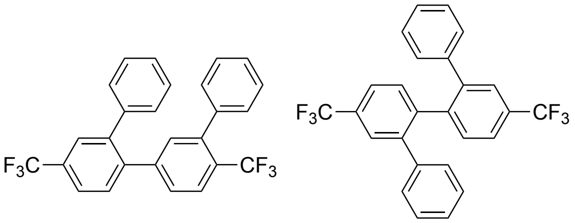

S3hb
(*meta*:*ortho* 16:84)

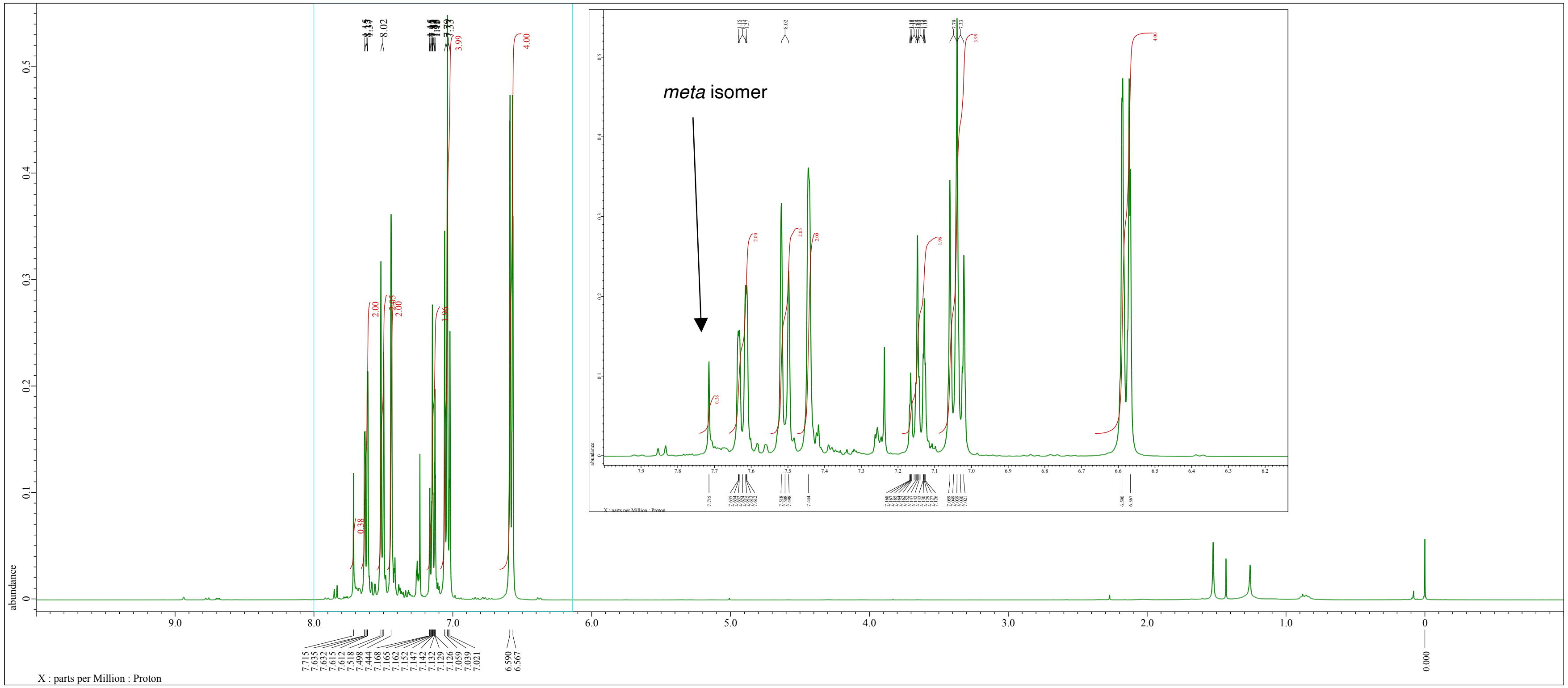

^{19}F NMR spectrum of **S3hb** (376 MHz, $CDCl_3$).

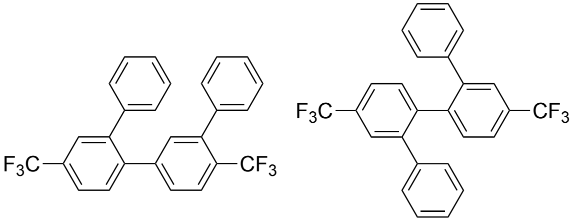

S3hb
(*meta*:*ortho* 16:84)

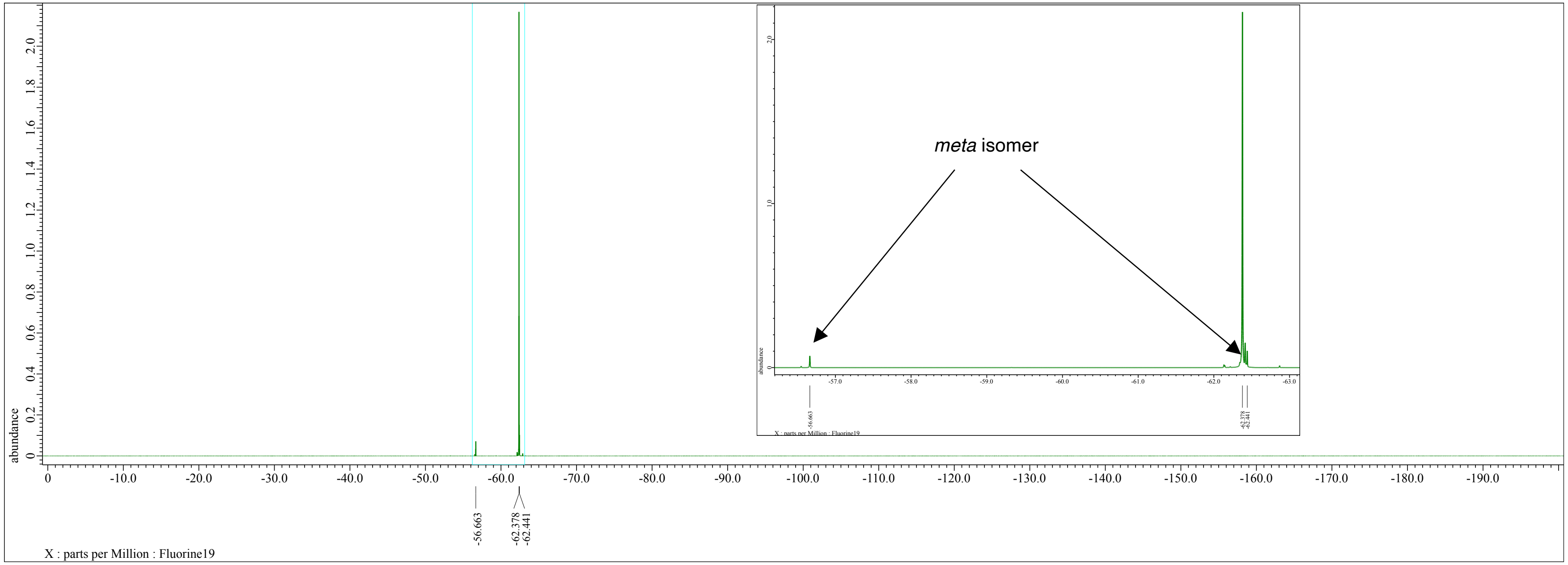

^{13}C NMR spectrum of **S3hb** (151 MHz, $CDCl_3$).

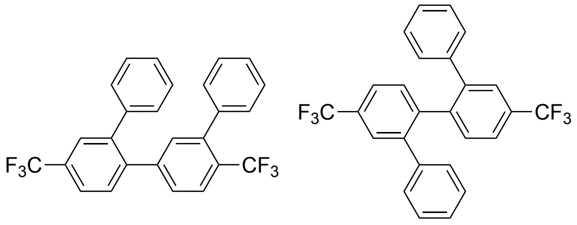

S3hb
(*meta*:*ortho* 16:84)

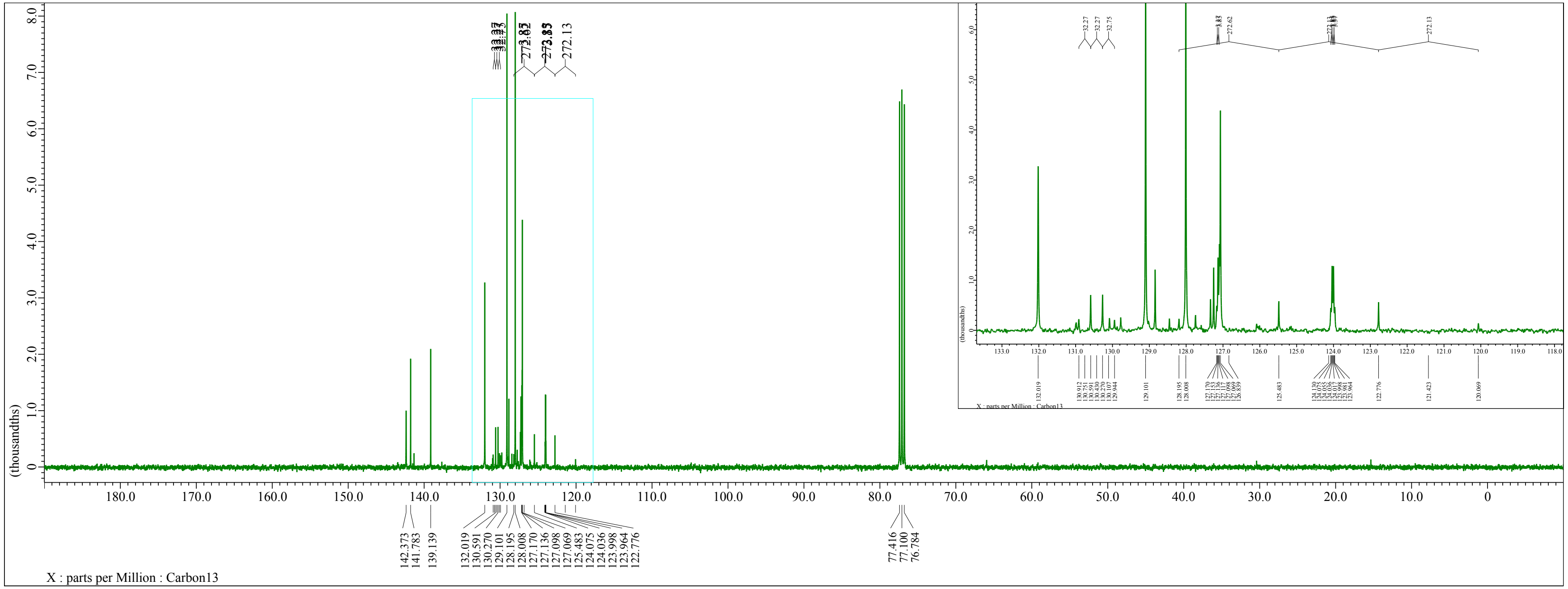

^{1}H NMR spectrum of **4** (600 MHz, $CDCl_3$).

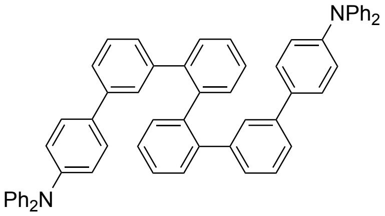

4

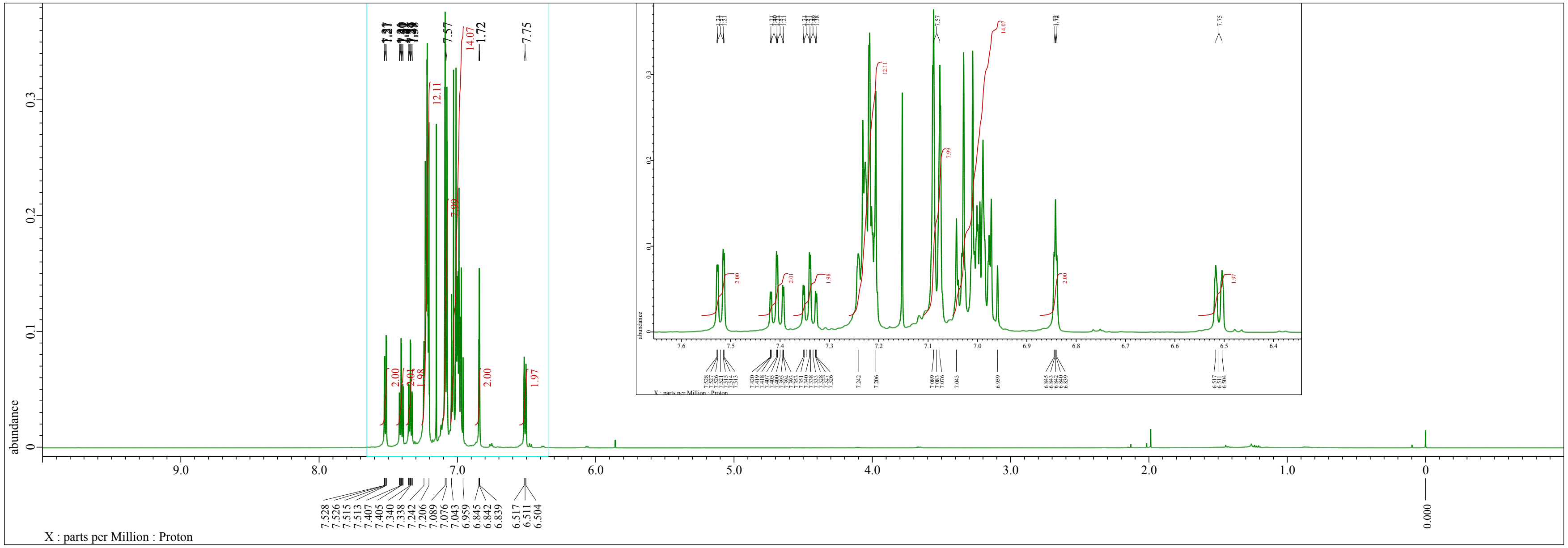

^{13}C NMR spectrum of **4** (151 MHz, $CDCl_3$).

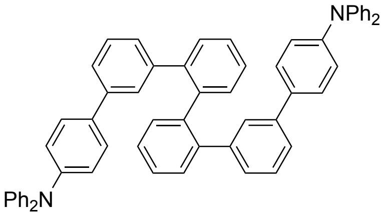

4

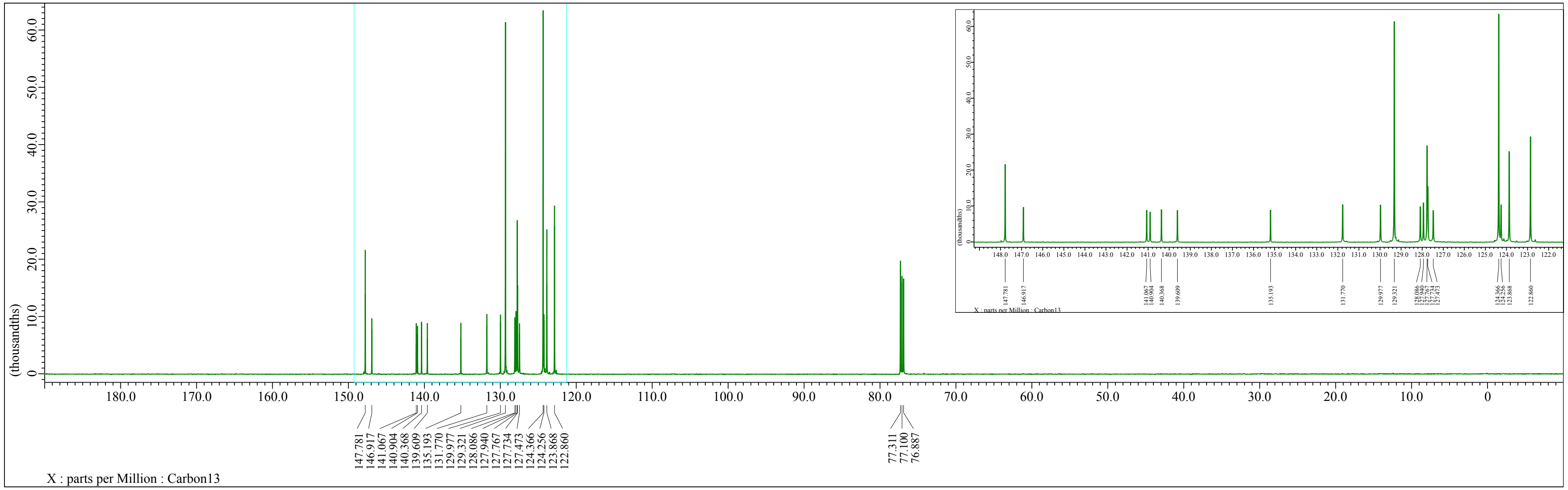

^{1}H NMR spectrum of **5** (600 MHz, $CDCl_3$).

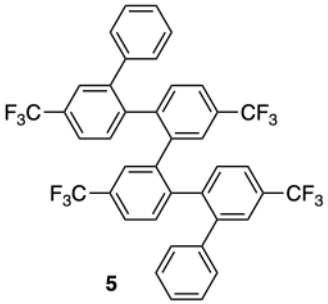

5

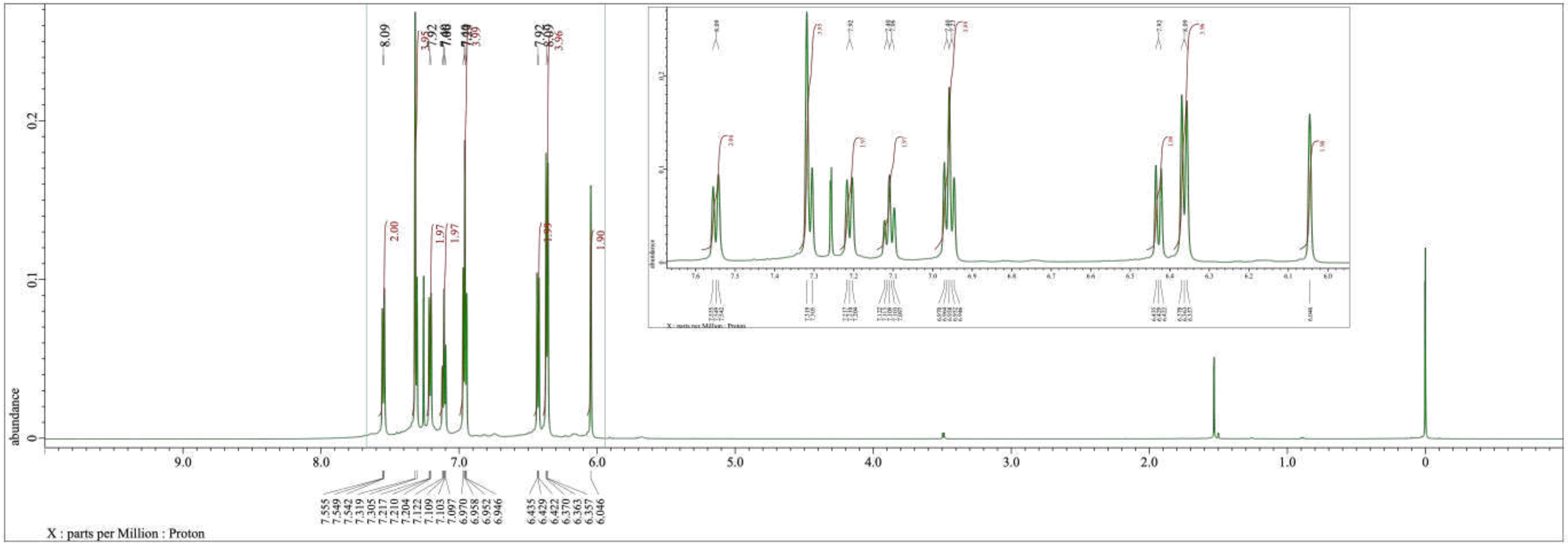

^{19}F NMR spectrum of **5** (376 MHz, $CDCl_3$).

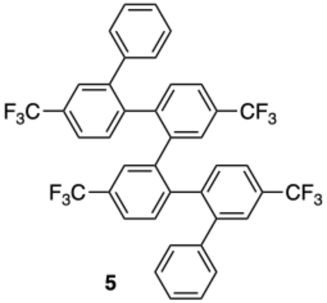

5

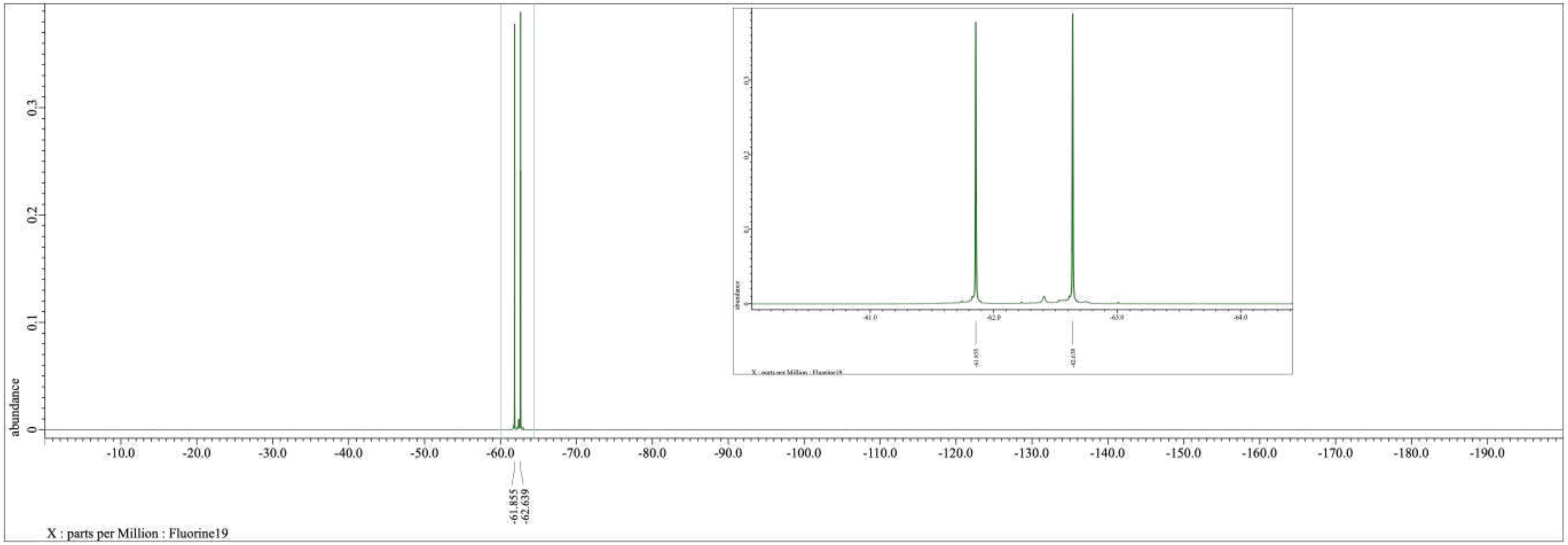

^{13}C NMR spectrum of **5** (151 MHz, $CDCl_3$).

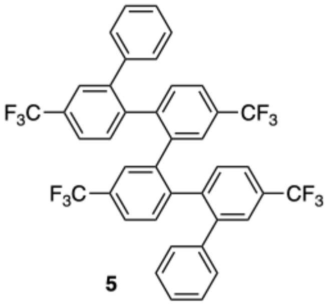

5

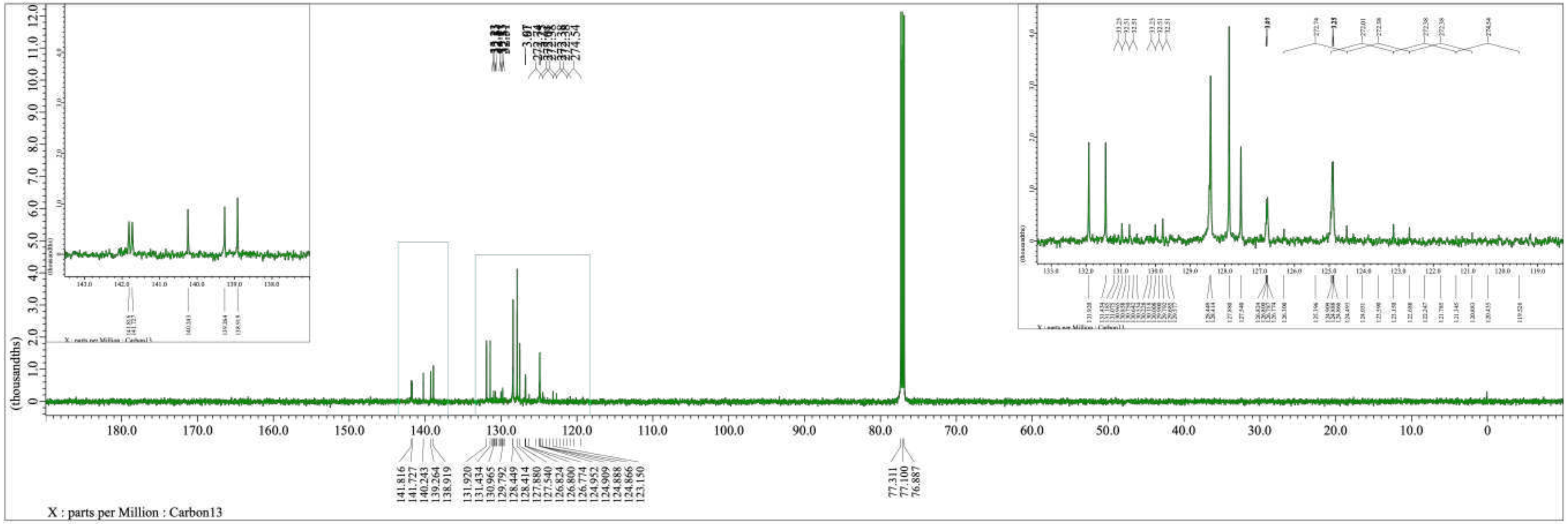

^{1}H NMR spectrum of **6** (600 MHz, $CDCl_3$).

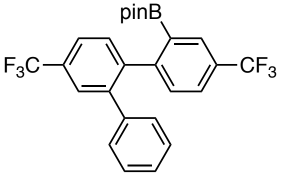

6

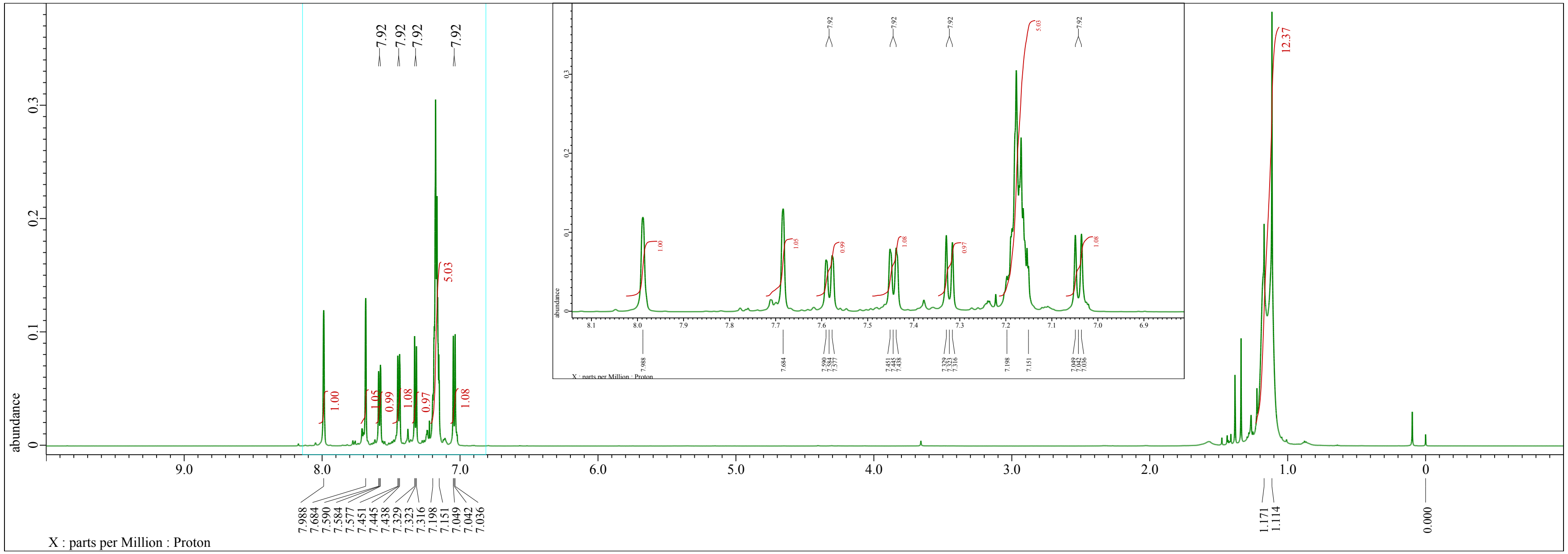

^{19}F NMR spectrum of **6** (376 MHz, $CDCl_3$).

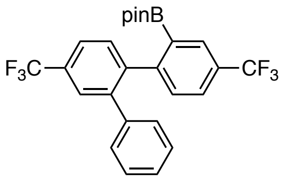

6

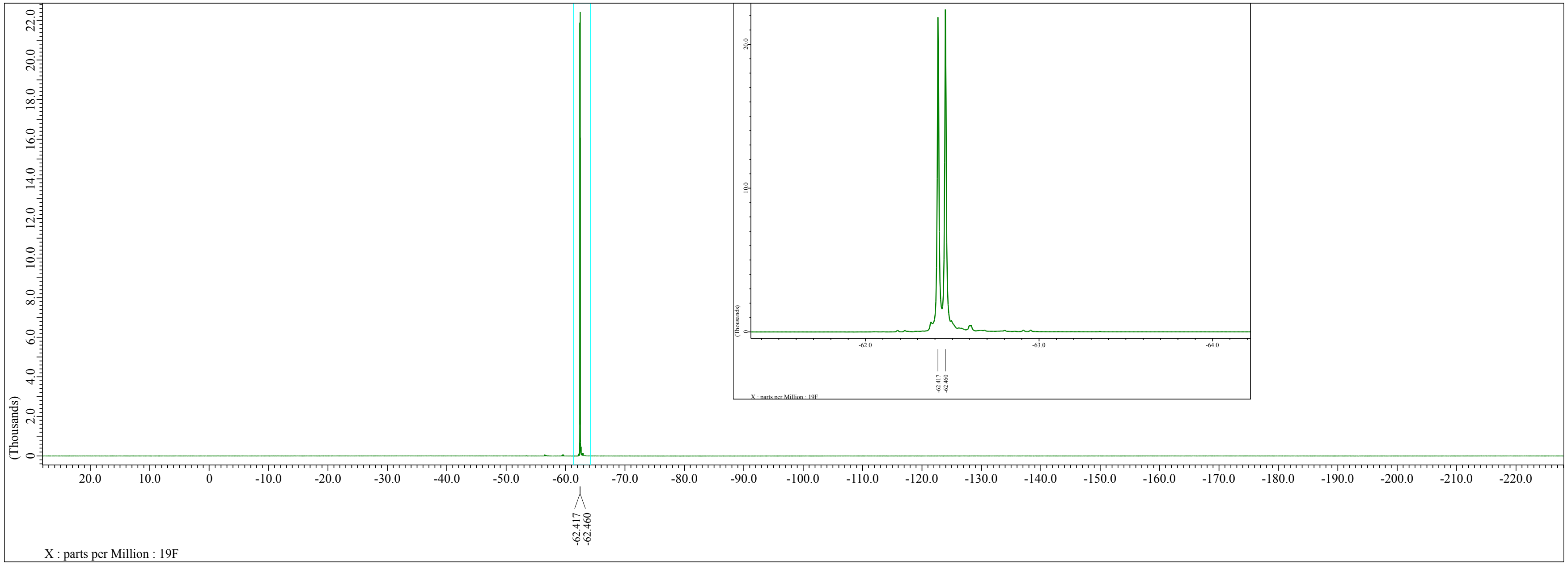

^{13}C NMR spectrum of **6** (151 MHz, $CDCl_3$).

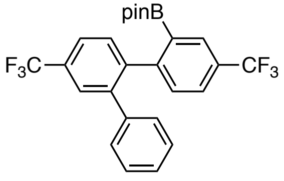

6

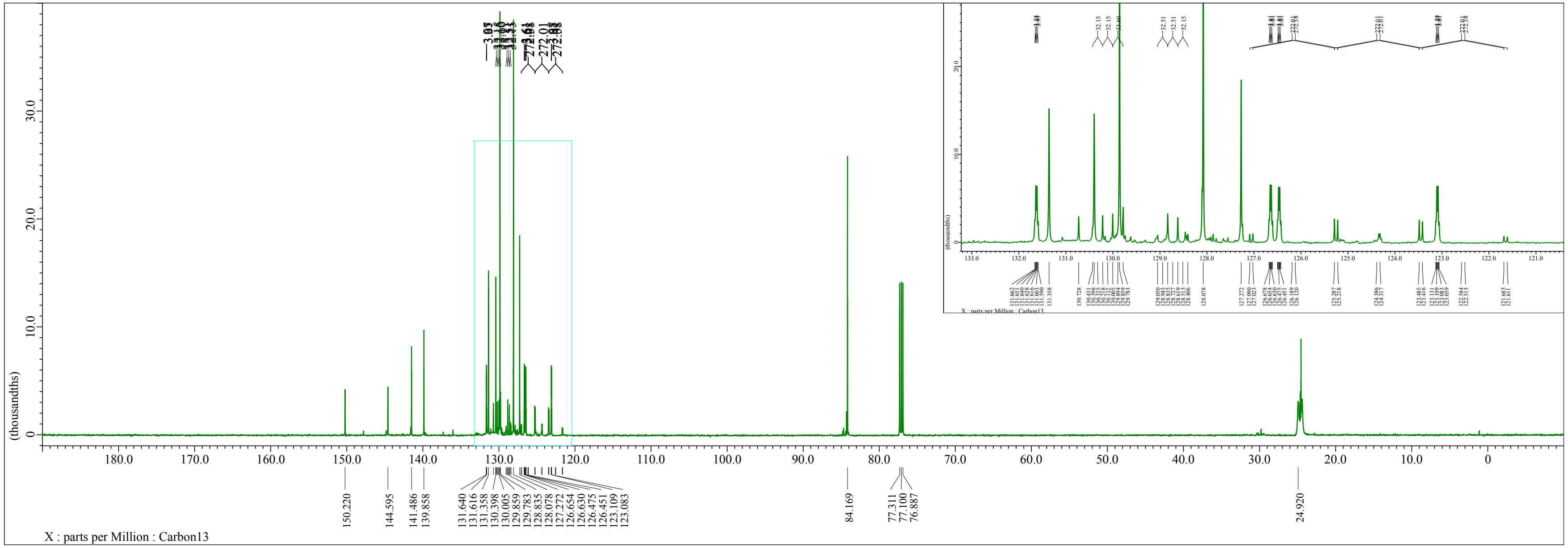

^{1}H NMR spectrum of **7** (600 MHz, $CDCl_3$).

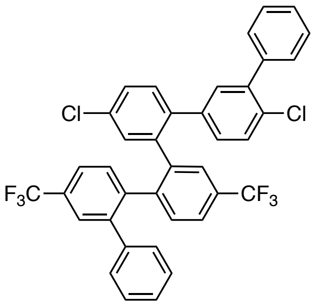

7

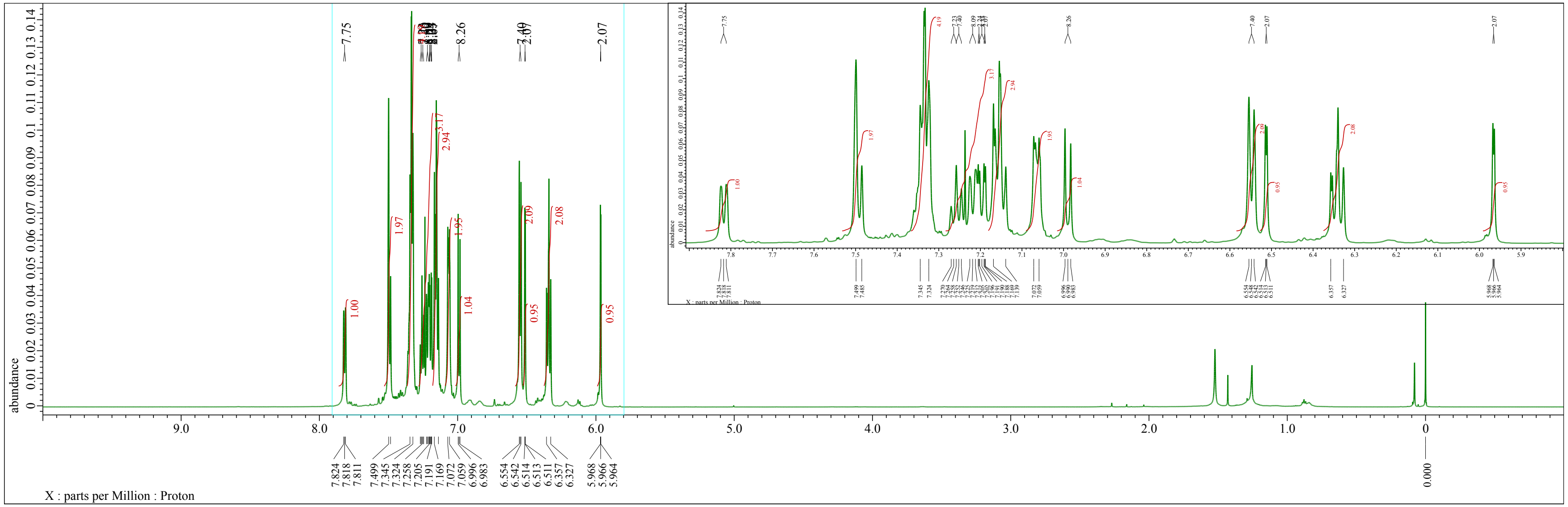

^{19}F NMR spectrum of **7** (376 MHz, $CDCl_3$).

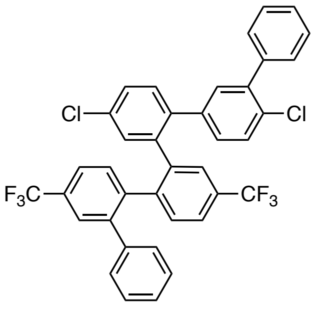

7

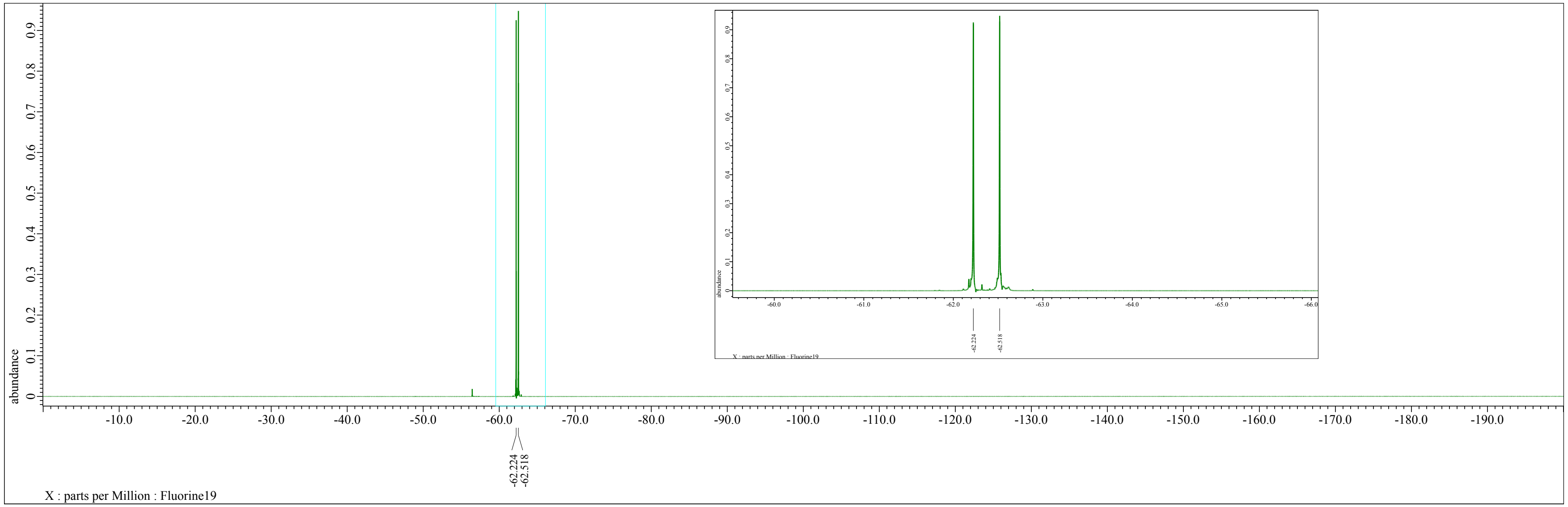

^{13}C NMR spectrum of **7** (151 MHz, $CDCl_3$).

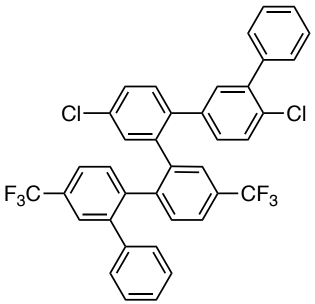

7

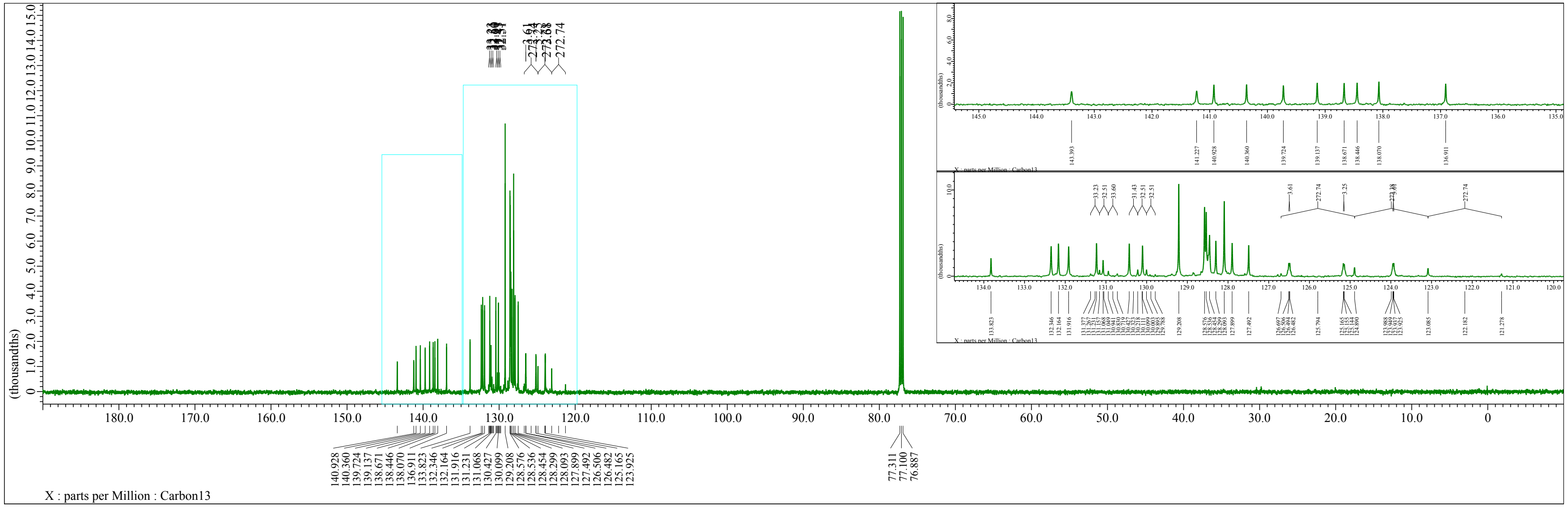

^{1}H NMR spectrum of **8** (600 MHz, DMSO-d_6).

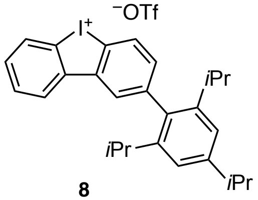

8

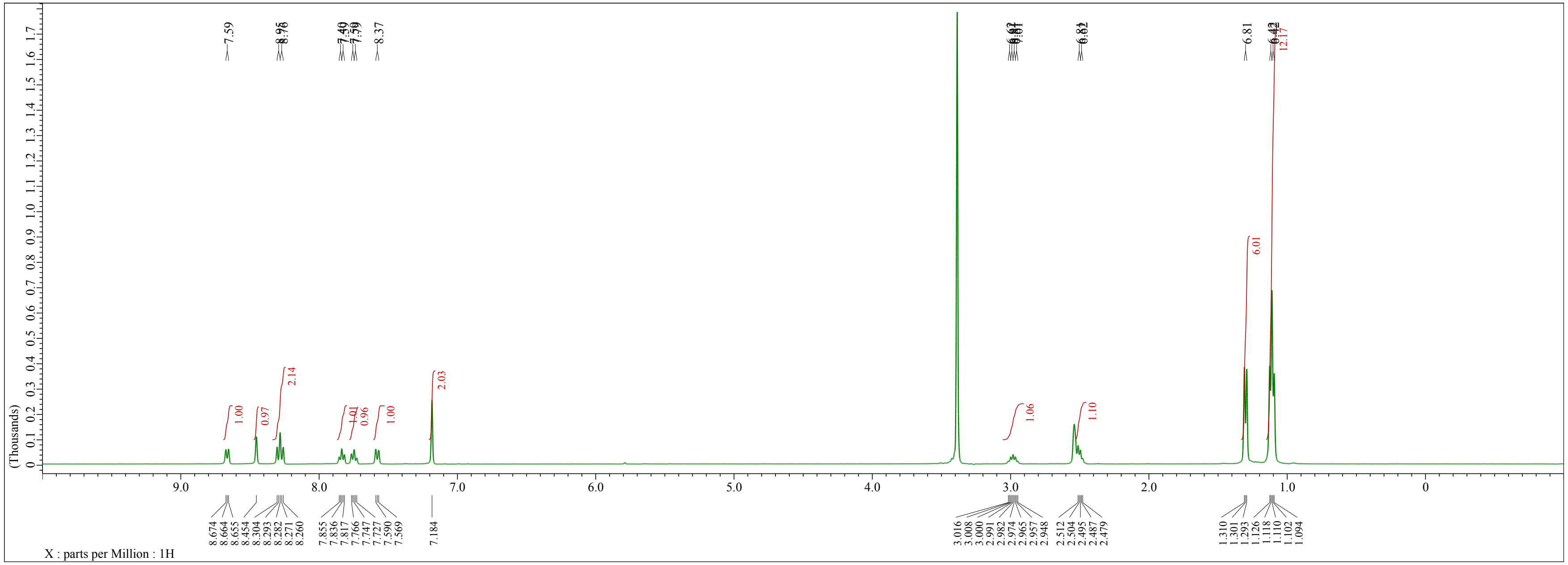

^{13}C NMR spectrum of **8** (151 MHz, DMSO-d_6).

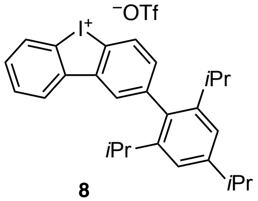

8

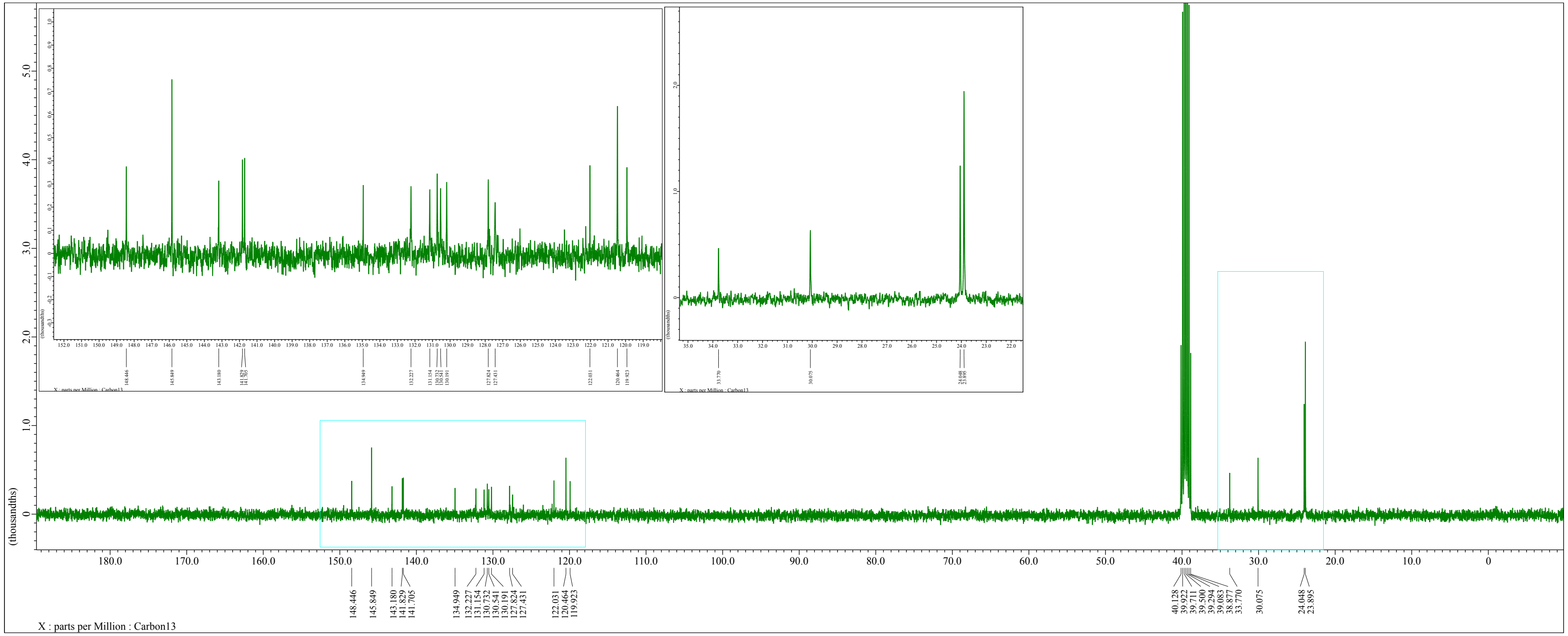

^{1}H NMR spectrum of **10** (600 MHz, $CDCl_3$).

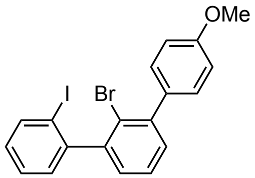

10

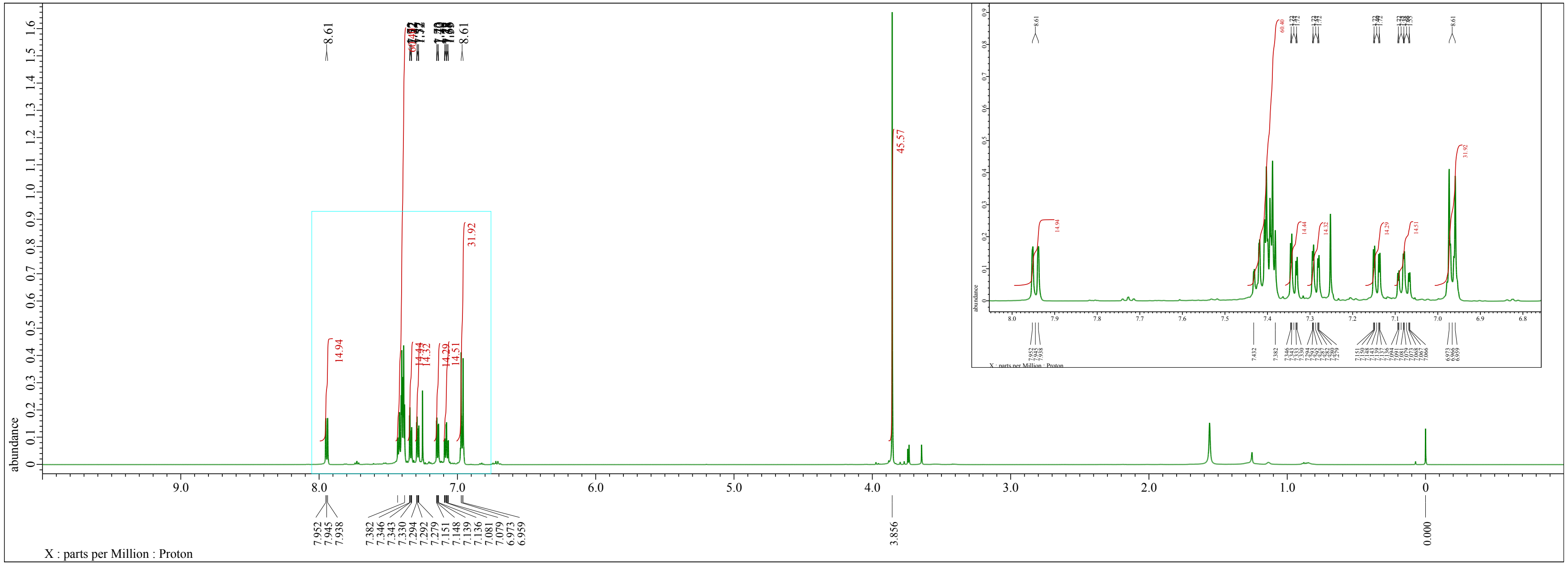

^{13}C NMR spectrum of **10** (151 MHz, $CDCl_3$).

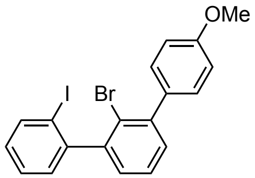

10

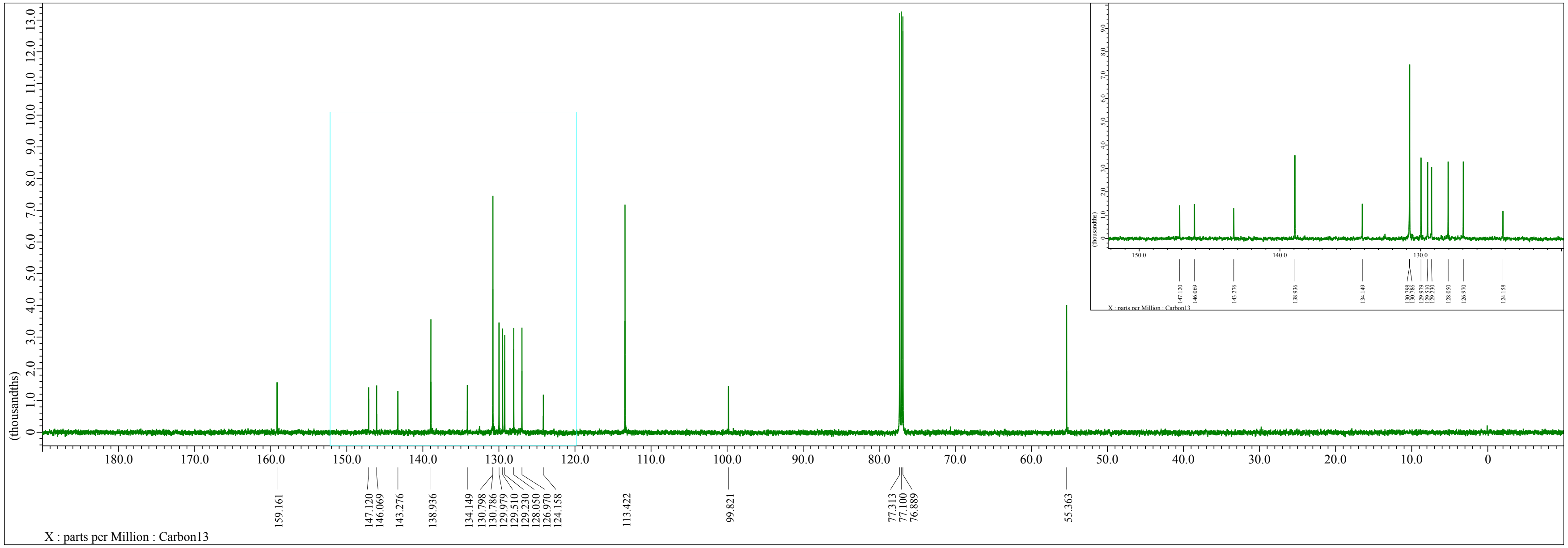

^{1}H NMR spectrum of **11** (600 MHz, $CDCl_3$).

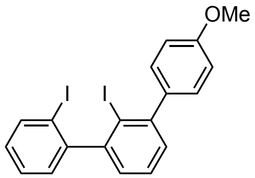

11

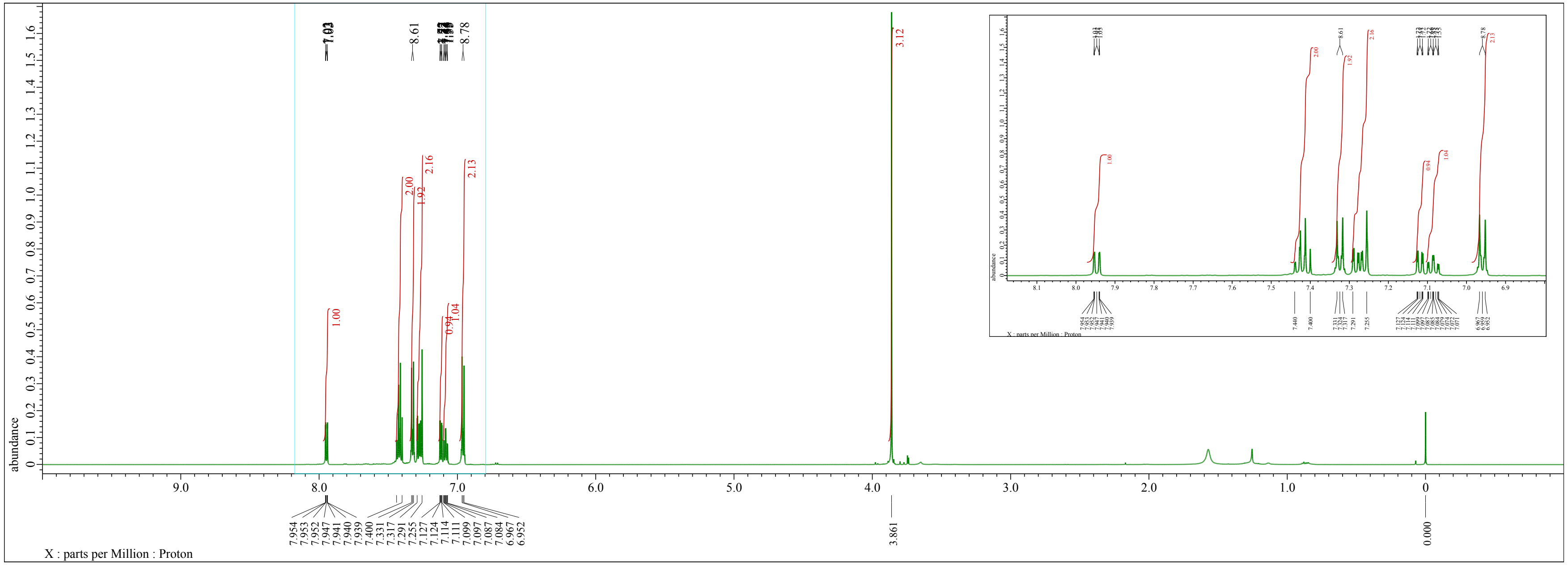

^{13}C NMR spectrum of **11** (151 MHz, $CDCl_3$).

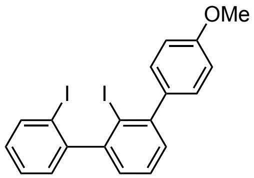

11

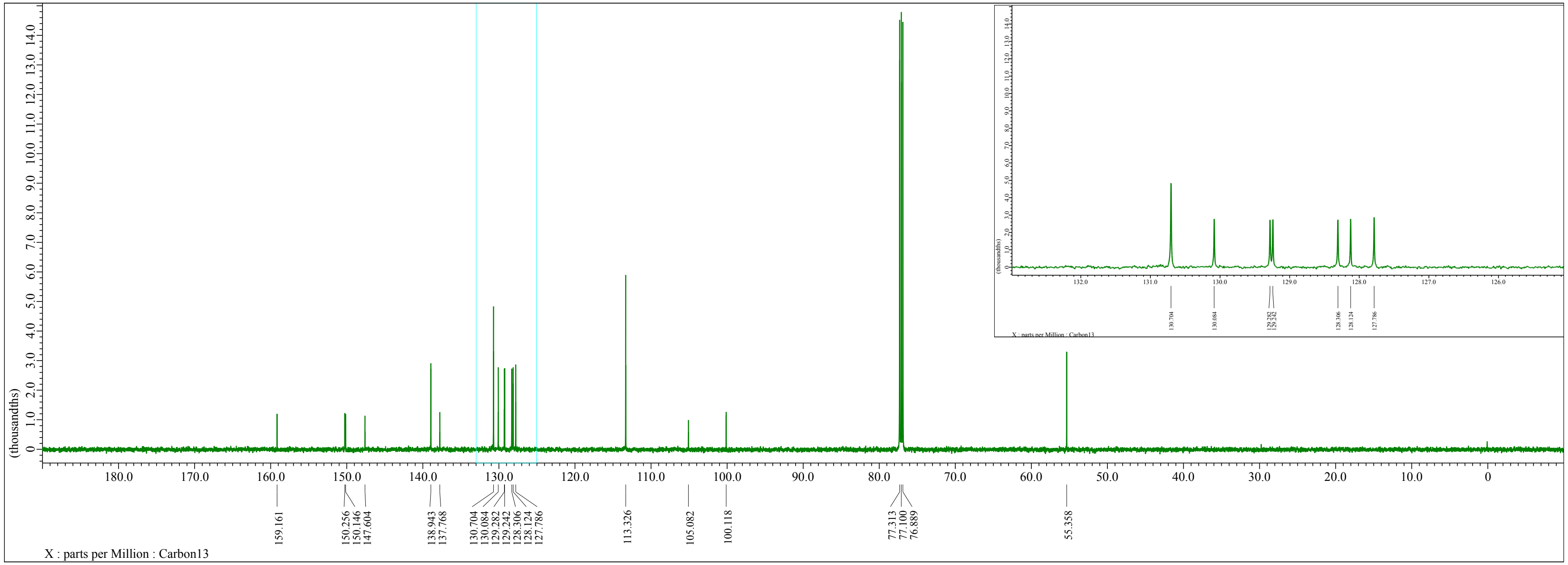

^{1}H NMR spectrum of **12** (600 MHz, $CDCl_3$).

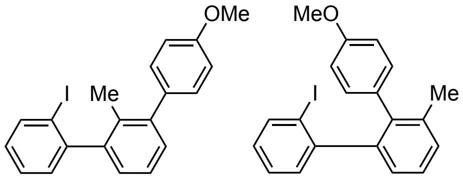

12
(*meta*:*ortho* = 94:6)

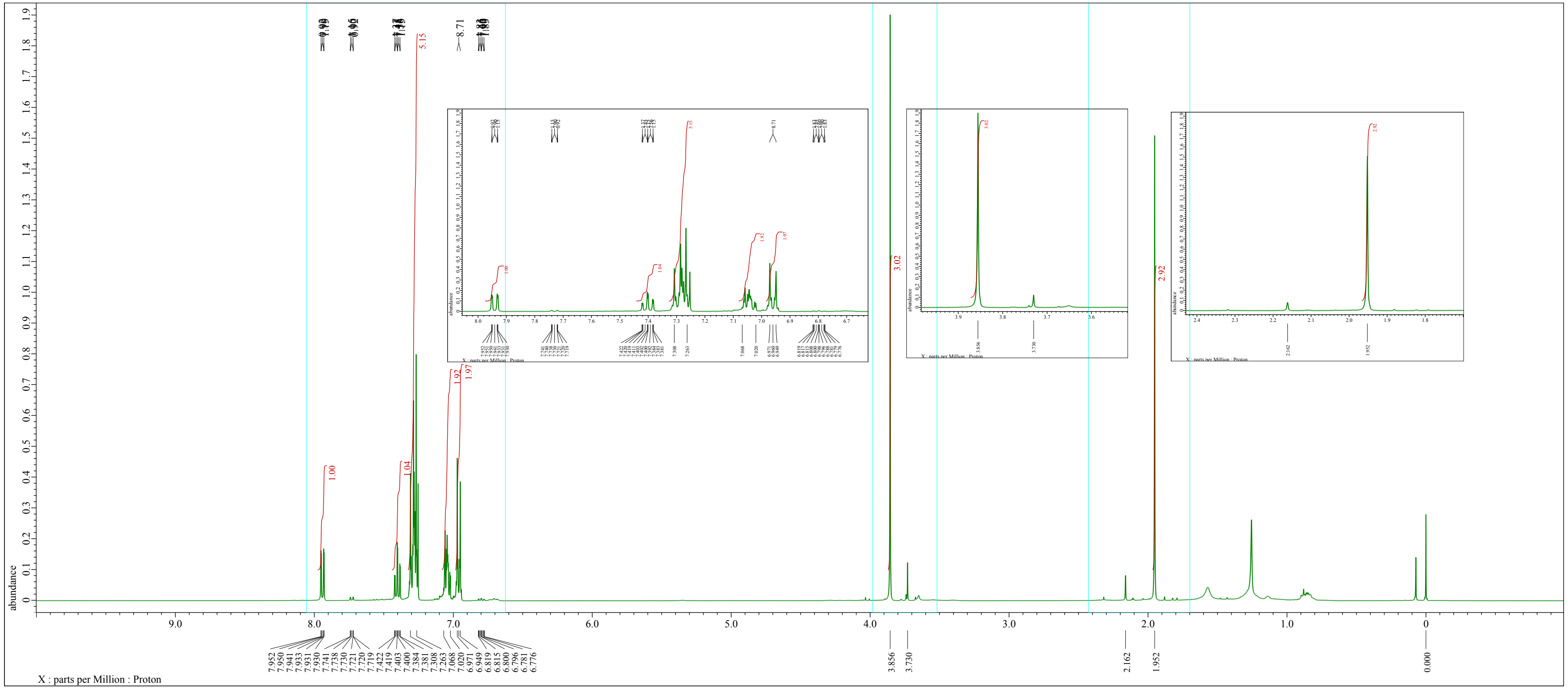

^{13}C NMR spectrum of **12** (151 MHz, CDCl$_3$).

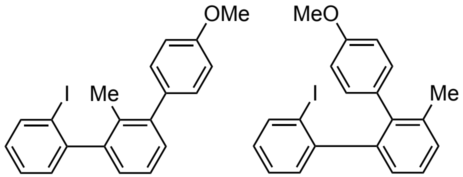

12
(*meta*:*ortho* = 94:6)

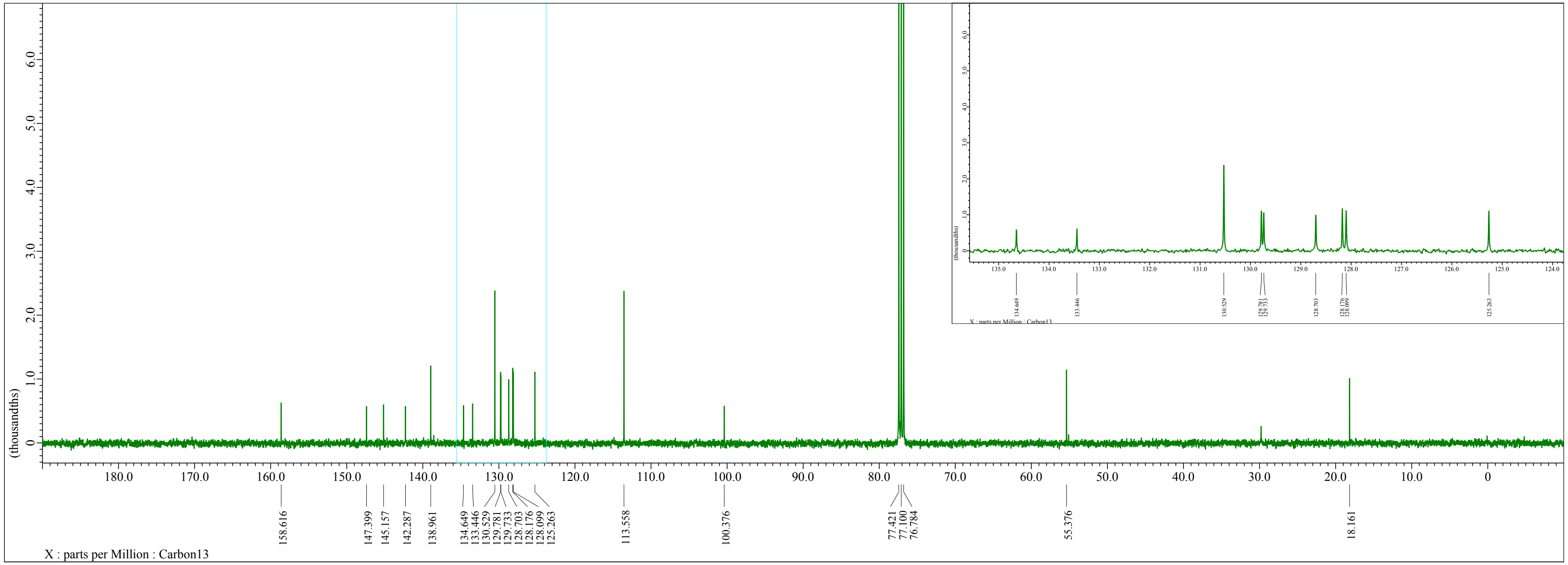

Supplement: Supplementary file 2 — Supporting File 2: anie72419‐sup‐0001‐SuppMat.pdf. [file ANIE-65-e6935149-s002.pdf]
